# Meta-Analysis of Genome-Wide Association and Gene Expression Studies Implicates Donor T Cell Function and Cytokine Pathways in Acute GvHD

**DOI:** 10.3389/fimmu.2020.00019

**Published:** 2020-02-03

**Authors:** Kati Hyvärinen, Satu Koskela, Riitta Niittyvuopio, Anne Nihtinen, Liisa Volin, Urpu Salmenniemi, Mervi Putkonen, Ismael Buño, David Gallardo, Maija Itälä-Remes, Jukka Partanen, Jarmo Ritari

**Affiliations:** ^1^Finnish Red Cross Blood Service, Helsinki, Finland; ^2^Stem Cell Transplantation Unit, Comprehensive Cancer Center, Helsinki University Hospital, Helsinki, Finland; ^3^Turku University Hospital, Turku, Finland; ^4^Department of Hematology, Genomics Unit, Hospital General Universitario Gregorio Marañón, Instituto de Investigación Sanitaria Gregorio Marañón (IiSGM), Madrid, Spain; ^5^Department of Hematology, Institut Català d'Oncologia, Institut d'Investigació Biomèdica de Girona (IDIBGI), Girona, Spain

**Keywords:** GvHD, GWAS, gene expression, meta-analysis, HSCT

## Abstract

Graft-vs.-host disease (GvHD) is a major complication after allogeneic hematopoietic stem cell transplantation that causes mortality and severe morbidity. Genetic disparities in human leukocyte antigens between the recipient and donor are known contributors to the risk of the disease. However, the overall impact of genetic component is complex, and consistent findings across different populations and studies remain sparse. To gain a comprehensive understanding of the genes responsible for GvHD, we combined genome-wide association studies (GWAS) from two distinct populations with previously published gene expression studies on GvHD in a single gene-level meta-analysis. We hypothesized that genes driving GvHD should be associated in both data modalities and therefore could be detected more readily through their combined effects in the integrated analysis rather than in separate analyses. The meta-analysis yielded a total of 51 acute GvHD-associated genes (false detection rate [FDR] <0.1). In support of our hypothesis, this number was significantly higher than that in a permutation meta-analysis involving the whole data set, as well as in separate meta-analyses on the GWAS and gene expression data sets. The genes indicated by the meta-analysis were significantly enriched in 277 Gene Ontology terms (FDR < 0.05), such as T cell function and cytokine-mediated signaling pathways, and the results highlighted several established immune mediators, such as interleukins and JAK-STAT signaling, and presented TRAF6 and TERT as potential effector candidates. Altogether, the results support the chosen methodological approach, implicate a role of gene-level variation in donors' key immunological regulators predisposing patients to acute GVHD, and present potential targets for therapeutic intervention.

## Introduction

Allogeneic hematopoietic stem cell transplantation (HSCT) is utilized as a curative treatment for life-threatening hematological malignancies. Despite improvements in survival and reduced morbidity over time, graft- vs.-host disease (GvHD) remains a major severe complication of HSCT (1). In the early stages of the treatment, donor T cell-mediated alloimmune reactions against the recipient's tissues give rise to acute GvHD (aGvHD), and consequently the majority of GvHD prophylaxis is directed at reducing aGvHD. Conditioning leads to gastrointestinal track-derived diffusion of microbial antigens, which, in turn, induces extensive immune activation and cytokine storm (2). However, the immune pathology of chronic GvHD (cGvHD) more closely resembles the symptoms of common autoimmune diseases involving dysregulation of immune tolerance and chronic tissue damage (3).

The success of HSCT treatment is strongly influenced by genetic disparities between the recipient and donor (4). The best characterized are classical human leukocyte antigen (HLA) genes encoded within the human major histocompatibility complex (MHC) on chromosome 6. Mismatching recipient and donor for HLA-molecules is a well-established risk factor for GvHD and overall survival (5, 6). However, both HLA and minor histocompatibility antigen disparities have been shown to result in the beneficial graft- vs.-leukemia effect reducing the risk of relapse (7, 8). Emphasizing the complex genetic background of the condition, the susceptibility to GvHD is also potentially altered by donor or recipient polymorphisms in genes involved in immune responses (9–11) and drug metabolism (12, 13), common deletions (14), and regulatory elements (15). Nevertheless, owing to the lack of replication of many of the results in large independent cohorts, a full understanding of relevant genetic factors remains elusive.

The first genome-wide association studies (GWASs) on GvHD were published by Sato-Otsubo et al. (16) and Bari et al. (17) in 2015. Sato-Otsubo et al. studied unrelated HSCT in a Japanese population and identified an association between HLA-DPB1 allele disparity and aGvHD. Additionally, they discovered three novel loci, including one in HLA-DP region, linked to severe aGvHD. Bari et al. identified recipient SUFU rs17114808 as a susceptibility locus for aGvHD in a cohort of US children (17). However, this result was not replicated in German pediatric and adult cohorts (18). In 2017, Goyal et al. reported three HLA-DP region loci in recipient genomes associated with severe aGvHD in a population with European-American ancestry (19). A genome-wide approach was also employed by Martin et al. (20) and Ritari et al. (21, 22), who both studied the effect of genome-wide recipient-donor mismatching. They concluded that an increase in genome-wide recipient mismatching is associated with an increased risk for GvHD.

The incomplete understanding of molecular mechanisms of GvHD-pathology and the shortage of solid biomarkers have prompted studies on GvHD-related gene expression. Donor gene expression profiling was utilized to detect “stronger alloresponders” by Baron et al. (23) in 2007, and in 2008, Buzzeo et al. (24) reported the first preliminary molecular signature of aGvHD. In 2015, Furlan et al. suggested that the recipient T cell transcriptional profile could be employed in identification of novel therapeutics, and aurora kinase A was presented as a potential target (25).

We hypothesized that combining different data sets and types could enhance the ability to detect weak but systematic common gene-level effects underlying GvHD-pathogenesis. In our present study, we explore the cooperative relationship between GWAS signals mapped into genes and published gene expression profiles in aGvHD and cGvHD. We carried out GWAS on Finnish and Spanish sibling HSCT cohorts and integrated these data with donor gene expression data sets by a meta-analysis based on gene rankings. We sought to determine whether the two data modalities lend support to each other and to understand the functional categories that may explain the outcome of HSCT in terms of GvHD pathogenesis.

## Materials and Methods

### Study Cohorts

The study consisted of three HLA-matched sibling cohorts including two populations: Finnish Cohort 1, Spanish Cohort 1, and Finnish Cohort 2. The characteristics of these study cohorts are presented in Table 1.

**Table 1:** Characteristics of the study cohorts.

	**Finnish Cohort 1^a^**	**Spanish Cohort 1^b^**	**Finnish Cohort 2^c^**	***P***
Number of recipients	262	268	174	
Number of donors	267	283	171	
Recipient age, median years (range)	48 (18–65)	50 (8–72)	51 (12-69)	0.021^d^
Donor age, median years (range)^e^	47 (11–68)	49 (4–78)	50 (11–72)	0.020^d^
Direction of transplantation, n (%)				0.674^f^
Male-male	73 (28)	87 (33)	35 (20)	
Male-female	57 (22)	62 (23)	32 (18)	
Female-female	61 (23)	51 (19)	29 (17)	
Female-male	71 (27)	66 (25)	41 (24)	
Diagnosis, *n* (%)				
Acute myeloid leukemia	73 (28)	88 (33)	59 (34)	0.312^f^
Acute lymphoblastic leukemia	39 (15)	24 (9)	21 (12)	0.112^f^
Chronic myeloid leukemia	37 (14)	13 (5)	8 (5)	<0.001^f^
Myelodysplastic syndrome	20 (8)	26 (10)	18 (10)	0.566^f^
Hodgkin's lymphoma	0 (0)	12 (5)	1 (1)	NA^g^
Non-Hodgkin's lymphoma	12 (5)	50 (19)	6 (3)	<0.001^f^
Myeloma	56 (21)	38 (14)	24 (14)	0.043^f^
Aplastic anemia	4 (2)	5 (2)	1 (1)	NA^g^
Other malignancies	21 (8)	11 (4)	37 (21)	<0.001^f^
Stem cell source, *n* (%)				<0.001^f^
Bone marrow	138 (53)	13 (5)	58 (33)	
Peripheral blood	124 (47)	254 (95)	116 (67)	
Conditioning regimen, *n* (%)				<0.001^f^
Myeloablative	199 (76)	110 (41)	132 (77)	
Reduced intensity conditioning	63 (24)	151 (57)	40 (23)	
GvDH prophylaxis, *n* (%)				NA^g^
Cyclosporine + methotraxate	0 (0)	151 (57)	76 (44)	
Cyclosporine	0 (0)	27 (10)	9 (5)	
Cyclosporine + methotraxate +steroid	193 (74)	0 (0)	68 (39)	
Cyclosporine + mycophenolate mofetil	50 (19)	31 (12)	4 (2)	
Other or missing data	18 (7)	58 (22)	17 (10)	
aGvHD grades II–IV, *n* (%)	42 (16)	94 (35)	67 (39)	<0.001^f^
aGvHD grades III–IV, *n* (%)	23 (9)	39 (15)	35 (20)	0.001 ^f^
cGvHD limited-extensive, *n* (%)	130 (54)	82 (41)	100 (58)	<0.001^f^
cGvHD extensive, *n* (%)	71 (39)	54 (32)	77 (45)	<0.001^f^

*aGvHD, acute graft- vs.-host disease; cGvHD, chronic graft- vs.-host disease; NA, not applicable*.

a *Finnish recipients underwent related donor (RD)-HSCT at Helsinki University Hospital, Comprehensive Cancer Center, Stem Cell Transplantation Unit, Finland, between 1993 and 2006*.

b *Spanish recipients underwent RD-HSCT at 13 Spanish transplant centers between 2002 and 2014*.

c *Finnish recipients underwent RD-HSCT at two Finnish centers: Helsinki University Hospital, Comprehensive Cancer Center, Stem Cell Transplantation Unit and Turku University Central Hospital between 2006 and 2016*.

d *The significance of variation between characteristics in the study cohorts was analyzed using the non-parametric Kruskal-Wallis test*.

e *Due to the missing data, approximately 20% of donor ages were imputed based on the respective recipient's age in the Spanish Cohort 1*.

f *The significance of variation between characteristics in the study cohorts was analyzed using the Pearson chi-square test*.

g *The significance of variation between characteristics in the study cohort was not analyzed due to the low frequency counts*.

Finnish Cohort 1 and Spanish Cohort 1 have been described previously in detail (11). Briefly, Finnish Cohort 1 consisted of 239 donor-recipient pairs, 23 individual recipients, and 28 individual donors with available clinical data and imputed genotype. All recipients received related donor (RD)-HSCT from 1993 through 2006 at Helsinki University Hospital, Comprehensive Cancer Center, Stem Cell Transplantation Unit, Finland. Spanish Cohort 1 was composed of 253 donor-recipient pairs, 15 individual recipients, and 30 individual donors with available clinical data and the imputed genotype. The recipients received RD-HSCT from 2002 through 2014 at 13 Spanish transplant centers. HLA-matching was performed at the HLA-A, -B, and -DRB1 loci in both cohorts.

Finnish Cohort 2 included 171 donor-recipient pairs, 3 individual recipients, and 1 individual donor with clinical data and an imputed genotype. RD-HSCT was implemented at two Finnish centers: Helsinki University Hospital, Comprehensive Cancer Center, Stem Cell Transplantation Unit and Turku University Central Hospital during the years 2006–2016. HLA-matching was performed at the HLA-A, HLA-B, HLA-C, and HLA-DRB1 loci (21).

The included clinical outcomes were grades 0, II–IV and III–IV for aGvHD, and grades 0, limited, and extensive for cGvHD. Grading was determined locally according to the European Society for Blood and Marrow Transplantation guidelines (26, 27).

The study conformed to the principles of the Declaration of Helsinki and was approved by the Finnish National Supervisory Authority for Welfare (Dnro V/74832/2017, V/3235/2019) and Health and the ethics committees of Helsinki University Central Hospital (382/13/03/01/2014, HUS/114/2018) and Turku University Central Hospital (ETMK 78/2012). Samples and data from Spanish patients included in this study were provided by the IDIBGI Biobank (Biobanc IDIBGI, B.0000872), integrated in the Spanish National Biobanks Network, and they were processed following standard operating procedures with the appropriate approval of the ethics and scientific committees.

### Genotyping and Imputation

The genotyping and imputation procedures have been previously described in detail (11). Briefly, Finnish Cohort 1 was genotyped using an Illumina Immunochip and Spanish Cohort 1 and Finnish Cohort 2 with Illumina were genotyped with an Immunoarray v2.0. Imputation of autosomal genotype data was performed using IMPUTE2 and the 1000 Genomes Phase 3 reference (28). Quality filtering for variants and samples was performed according to Anderson et al. (29), and an IMPUTE2 INFO-field measure ≥0.5 was used as the cut-off for post-imputation filtering (30). The three datasets were filtered and imputed in separate processes, and the final number of variants included in the analyses was 5041081 for Finnish Cohort 1, 5737173 for Spanish Cohort 1, and 9105726 for Finnish Cohort 2.

### Statistical Analysis of the Characteristics of the Study Cohorts

The significance of differences in recipient and donor age among the three study cohorts was analyzed using the non-parametric Kruskal–Wallis test. Pearson's chi-square test was used to evaluate the frequencies of diagnosis, gender direction of transplantation, stem cell source, conditioning regimen, and GvHD grades. Due to low frequency, the variation in Hodgkin's lymphoma and aplastic anemia diagnosis were not analyzed. The significance level of tests (alpha level) was set at 0.05.

### Population Structure Inference and Logistic Regression Analysis of the Covariates

The population structures were determined using principal component (PC) analysis (PCA). The analysis was implemented with PLINK 1.90b4.1 (www.cog-genomics.org/plink/1.9/) (31). Genotyped variants shared by all three cohorts were pruned to generate a subset of variants in approximate linkage equilibrium *(–indep-pairwise 50 5 0.5*). Dimensional reduction was executed with the command –*pca* and the top 20 PCs for each cohort were extracted separately. The combined data were used to generate a scatterplot matrix of the five initial PCAs [Supplementary Figure 1 (Supplementary Data Sheet 1)]. The plot was generated using R version 3.5.0.

Logistic regression analysis (PLINK command –*logistic*) was employed to investigate the effect of baseline covariates on the GvHD-related outcome. Binary aGvHD or cGvHD status was used as a dependent variable, and recipient gender, recipient age, direction of transplantation, and stem cell source were included as independent variables [Supplementary Table 1 (Supplementary Data Sheet 1)]. The results are presented as odds ratios (ORs) with 95% confidence intervals (CIs), and a P-value < 0.05 was considered statistically significant.

### GWAS

In the preliminary analysis, the associations between recipient and donor genotypes and GvHD clinical outcomes were examined using the PLINK 1.07 (32) 1df chi-square allelic test (command –*assoc*) and are expressed as odds ratios with 95% confidence intervals. Variants with a minor allele frequency < 0.01, Hardy-Weinberg disequilibrium *P*-value < 1 × 10^−5^, and missing call rate >0.1 were excluded from the analysis. After correction for multiple testing, a genome-wide *P*-value < 1 × 10^−7^ was considered statistically significant.

For the adjusted analyses, the association between variants and clinical outcomes was determined using PLINK 1.90b4.1 (31) logistic regression analysis (command –*logistic*). Variant filtering followed the previously described practice. For all recipients, recipient gender, recipient age, graft type, and the first three cohort specific PCs were used as covariates. For the donors, donor gender, donor age, graft type, and the first three PCs were used as covariates. The donor age data were incomplete in Finnish Cohort 1 and Spanish Cohort 1, and the missing data were imputed using the corresponding recipient's age using linear regression.

### Meta-Analyses

**Figure 1:**
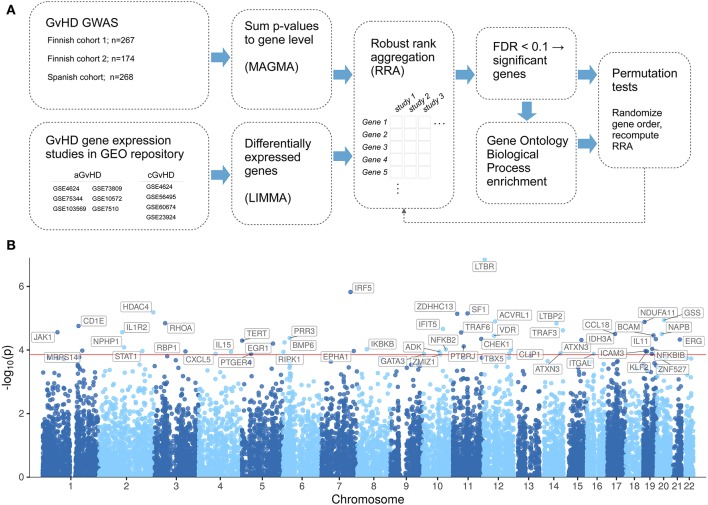
Overview of the study setup and associated genes. **(A)** Schematic diagram showing the main steps of the analysis pipeline. **(B)** Manhattan plot of donor aGvHD meta-analysis results. The meta-analysis includes the donor GWAS II–IV vs. 0 results from Finnish Cohort 1, Spanish Cohort 1, Finnish Cohort 2, and six previously published aGvHD gene expression studies. The analysis was conducted as depicted in the Method section. The red line indicates a false detection rate of <0.1.

The Figure 1A presents a schematic diagram of the main steps of the analysis pipeline. The SNP associated *P*-values of donor aGvHD grades II–IV vs. 0 and cGvHD limited-extensive vs. 0 GWASs from all three study cohorts were mapped into genes using MAGMA (33) v1.07b (https://ctg.cncr.nl/software/magma) with the default 1000 Genomes GRCh37 LD data and gene annotation data provided with MAGMA.

GvHD-related gene expression (GE) data sets (23–25, 34–39) were retrieved from the Gene Expression Omnibus data repository (https://www.ncbi.nlm.nih.gov/geo/). A summary of the selected GE studies is presented in the Supplementary Table 2 (Supplementary Data Sheet 1). Analysis of differential expression in the GE data sets was conducted using the R package limma v3.38.3 functions lmFit for fitting linear models, and eBayes to determine the empirical Bayes moderated standard errors of the models.

Using the gene lists obtained from the GWAS and GE analyses, we conducted three independent meta-analyses: (1) the GWASs together, (2) the GE studies together, and (3) the GWASs and the GE studies combined, for both the aGvHD and cGvHD data sets. These analyses were executed using the robust rank aggregation (RRA) method (40) implemented in the R package RobustRankAggreg v1.1 function aggregateRanks using exacted *P*-value calculations. Genes with a false discovery rate (FDR) < 0.1 were considered significant and included in the downstream analyses for gene ontology (GO) biological processes (BP) enrichment. GO:BP enrichment was performed using the R package clusterProfiler (41) v3.10.1 function enrichGO with the full list of analyzed genes as the background set. GO terms with an FDR < 0.05 were considered statistically significant.

To control for subtle biases that could potentially cause inflated numbers of false positives, we conducted permutation tests on the gene lists included in the RRA meta-analysis. The gene list permutation was repeated 100 times, and for each iteration, the number of genes with an FDR < 0.1 was calculated. These values were then compared with the numbers of significant genes from the RRA conducted on the original gene lists to estimate whether the observed number of significant genes could be obtained by chance alone.

A similar permutation test was conducted for the GO:BP enrichment analysis, whereby the enrichment at an FDR < 0.05 was re-calculated for each iteration of the permutated RRA for genes with an FDR < 0.1. Each enriched gene combination was accepted only once to limit the number of redundant GO terms.

The clustering and visualization of the GO enrichment results were performed using the REVIGO (42) Web server (http://revigo.irb.hr/).

### Visualization of Colocalization of GWAS and eQTL Events

The visualization of colocalization of GWAS and expression quantitative trait loci (eQTL) events was performed using the R package LocusCompareR (43). The GWAS variants of aGvHD-associated genes were selected using a ± 1Mb range from the gene boundaries. The corresponding blood *cis*-eQTL SNPs were extracted from the eQTLGen Consortium database (http://www.eqtlgen.org) (44). This database incorporates 31,684 individuals and 37 datasets, resulting in 16,989 *cis*-eQTL genes. The generated plots visualized the distributions of *cis*-eQTL and GWAS signals and the linkage disequilibrium to the selected variant.

### Gene Expression Directions

To visualize the direction and distribution of expression of the significant RRA genes, the gene-wise expression values were extracted from each included GE study. For each study, the expression values for the cases were divided by the mean value of the controls and thereafter scaled by subtracting the mean and dividing by the standard deviation. The processed values were then pooled for each gene from all of the GE studies and plotted as boxplots.

### Data and Code Availability Statement

The limitations of ethical permits restrict the public distribution of personal data including individual genetic data. The code for the meta-analyses is publicly available in GitHub (https://github.com/FRCBS/GvHD_meta).

### Supplemental Information

The supplementary information includes Supplementary Data Sheets 1–4.

## Results

### Characteristics of the HSCT Study Cohorts and the GvHD-Related Risk Factors

Table 1 summarizes the key characteristics of the HSCT study cohorts. The three cohorts differed substantially in many aspects. The distributions of recipient and donor ages and the frequencies of diagnoses, stem cell sources, conditioning regimens, and GvHD outcomes significantly varied among the cohorts. Additionally, the GvHD prophylaxis regimens varied markedly. The association between the common GvHD-related risk factors and the disease outcomes diverged as depicted in Supplementary Table 1 (Supplementary Data Sheet 1). Increasing recipient age was significantly associated with all GvHD outcomes in Finnish Cohort 1 (all *P*-values ≤ 0.036). However, the odds ratios were low (from 1.035 to 1.087) and age had no effect in the other two study cohorts. Female gender was associated with beneficial cGvHD outcomes in Spanish Cohort 1 (*P*-values = 0.001). Using the bone marrow as a stem cell source reduced the risk for both aGvHD and cGvHD in Finnish Cohort 2 (*P*-values ≤ 0.045, except for 0.065 in the cGvHD limited-extensive group) and for cGvHD in Finnish Cohort 1 (*P*-values ≤ 0.007).

### GWASs of the HSCT Study Cohorts

In the unadjusted preliminary GWAS in Finnish Cohort 1, we found severe aGvHD-associated loci in the MHC region in the recipient genotype at a genome-wide significance level of *P* < 5 x 10^−8^ and in the donor genotype at a suggestive significance level of *P* < 5 × 10^−5^ [Supplementary Figure 2 (Supplementary Data Sheet 1), panel A for recipients and panel C for donors]. These results were not replicated in the two other HSCT study cohorts, and none of the variants reached a genome-wide significance level (data not shown). Additionally, all genome-wide significance was abolished in Finnish Cohort 1 after adjusting the analyses by recipient age, recipient gender, stem cell source, and the top three PCs [Supplementary Figure 2 (Supplementary Data Sheet 1), panel B for recipients and D for donors].

### Meta-Analyses

**Figure 2:**
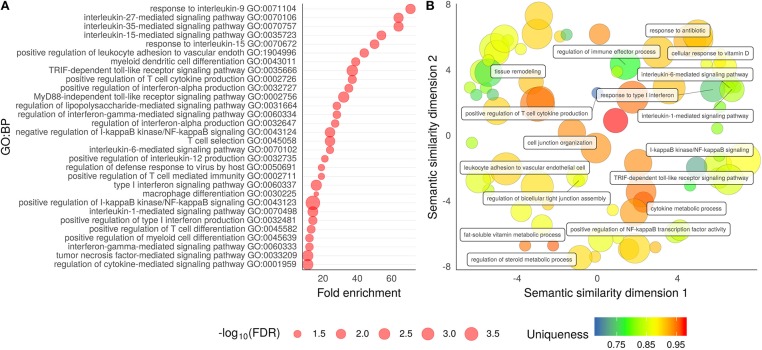
Visualization of enrichment and sematic similarity of the aGvHD-associated gene ontologies. The aGvHD-associated genes discovered in the meta-analysis were analyzed for enriched Gene Ontology Biological Process (GO:BP) categories. **(A)** T cell activation and cytokine response-focused GO:BP categories. The X-axis shows the enrichment level, and the size of the circle depicts the false detection rate (FDR). **(B)** Sematic similarity analysis of all the GO:BP categories. The bubbles represent the individual GO:BP categories, and more related terms are closer in the plot. The uniqueness from the total mean is shown by a color scale, with blue indicating a less unique and red indicating a more unique category. The size of the circle indicates the number of detected genes within the underlying GO term. The labels present some of the cluster representatives.

All data sets included in the RRA meta-analyses are presented in Table 2, and more detailed information on the GE studies performed is listed in Supplementary Table 2 (Supplementary Data Sheet 1). The combined meta-analysis of cGvHD-related data sets revealed only cysteine protease legumain (LGMN) as associated with cGvHD at the FDR < 0.1 level. The analysis of aGvHD-related data sets revealed 51 aGvHD-associated genes at the FDR < 0.1 level, including lymphotoxin beta receptor (LTBR), Janus kinase 1 (JAK1), tumor necrosis factor (TNF) receptor associated factor 6 (TRAF6), signal transducer and activator of transcription 1 (STAT1), vitamin D receptor (VDR), interleukin (IL) 11, IL15, and IL1 receptor 2 (IL1R2) [Figure 1; Supplementary Table 3 (Supplementary Data Sheet 1)]. The GO enrichment analysis of these genes detected 277 aGvHD-associated BPs at the FDR < 0.05 level [Supplementary Table 4 (Supplementary Data Sheet 1)]. The majority of associated GO:BP categories were strongly linked to immune responses and regulation, highlighting T cell function and cytokine-mediated signaling pathways. The top 30 of these detailed GO:BP categories are presented in Figure 2A. The degree of relatedness of all ontological categories defined by their annotation, the sematic similarity, is shown in Figure 2B.

**Table 2:** The data sets included in the aGvHD and cGvHD meta-analyses.

**Meta-analysis**	**GWAS**	**Gene expression study^a^(GEO number)**
aGvHD	Finnish Cohort 1 donors aGvHD II–IV vs. 0 Spanish Cohort 1 donors aGvHD II–IV vs. 0 Finnish Cohort 2 donors aGvHD II–IV vs. 0	GSE4624 GSE75344 GSE103569 GSE73809 GSE10572 GSE7510
cGvHD	Finnish Cohort 1 donors cGvHD limited-extensive vs. 0 Spanish Cohort 1 donors cGvHD limited-extensive vs. 0 Finnish Cohort 2 donors cGvHD limited-extensive vs. 0	GSE4624 GSE56495 GSE60674 GSE23924

*aGvHD, acute graft- vs.-host disease; cGvHD, chronic graft- vs.-host disease; GEO, gene expression omnibus; GWAS, genome-wide association study*.

a *https://www.ncbi.nlm.nih.gov/ge*.

**Figure 3:**
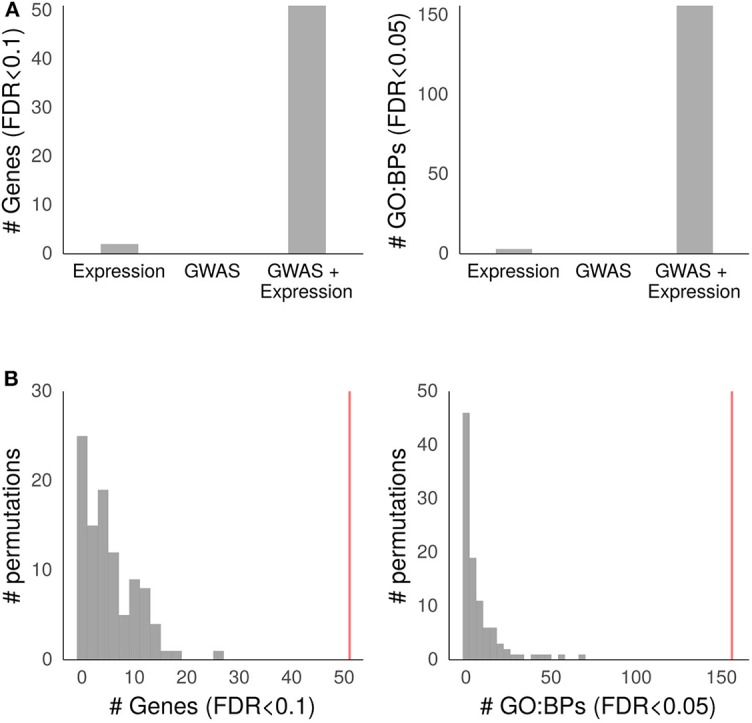
Validation of the aGvHD meta-analysis. **(A)** Results of meta-analyses on the GWAS and GE studies separately and the meta-analysis on the GWAS and GE data together. The left side panel shows the number of associated genes at a false detection rate (FDR) level of <0.1, and the right side panel shows the number of Gene Ontology biological process (GO:BP) categories at an FDR < 0.05. **(B)** Results of 100 meta-analyses with permuted gene order. The left side panel shows the numbers of aGvHD-associated genes at an FDR level of <0.1, and the right side panel shows the numbers of GO:BP categories at an FDR level of <0.05. The vertical red line depicts the corresponding values from the original meta-analysis. The meta-analyses include the donor GWAS II–IV vs. 0 results from Finnish Cohort 1, Spanish Cohort 1, Finnish Cohort 2, and six previously published aGvHD gene expression (GE) studies. The analyses were conducted as described in the Methods section.

To confirm the validity of the combined meta-analysis including both the GWASs and the GE studies, we performed a meta-analysis of the GWASs and the GE studies separately. Figure 3A presents the comparison of aGvHD-associated genes and unique GO:BP gene combinations in different analysis settings. The meta-analysis of the three GWASs did not result in any associated genes at the FDR < 0.1 level and, consequently, no GO:BPs at the FDR < 0.05 level. We found two genes and three GO:BPs associated with aGvHD in the separate GE meta-analyses, but these numbers were substantially lower than those in the combined meta-analysis (51 genes and 156 unique GO:BP gene combinations).

The mean number of significant (FDR < 0.1) permuted RRA genes was 5.69 with an SD of 0.48, which corresponded with the expected number of 5.1 based on an FDR < 0.1 in the true data. In the GO enrichment analysis of permuted data, the mean number of significant (FDR < 0.05) unique GO terms was 7.95 with an SD of 1.28, which was also close to the expected value of 7.8 obtained from the true data at an FDR < 0.05. The number of significant genes or GO terms did not reach the value obtained with the true data in any of the permutation iterations. Figure 3B depicts the results of the permutation tests.

### Colocalization of GWAS and eQTL Events and the Combined Effect Directions of Gene Expression Studies

**Figure 4:**
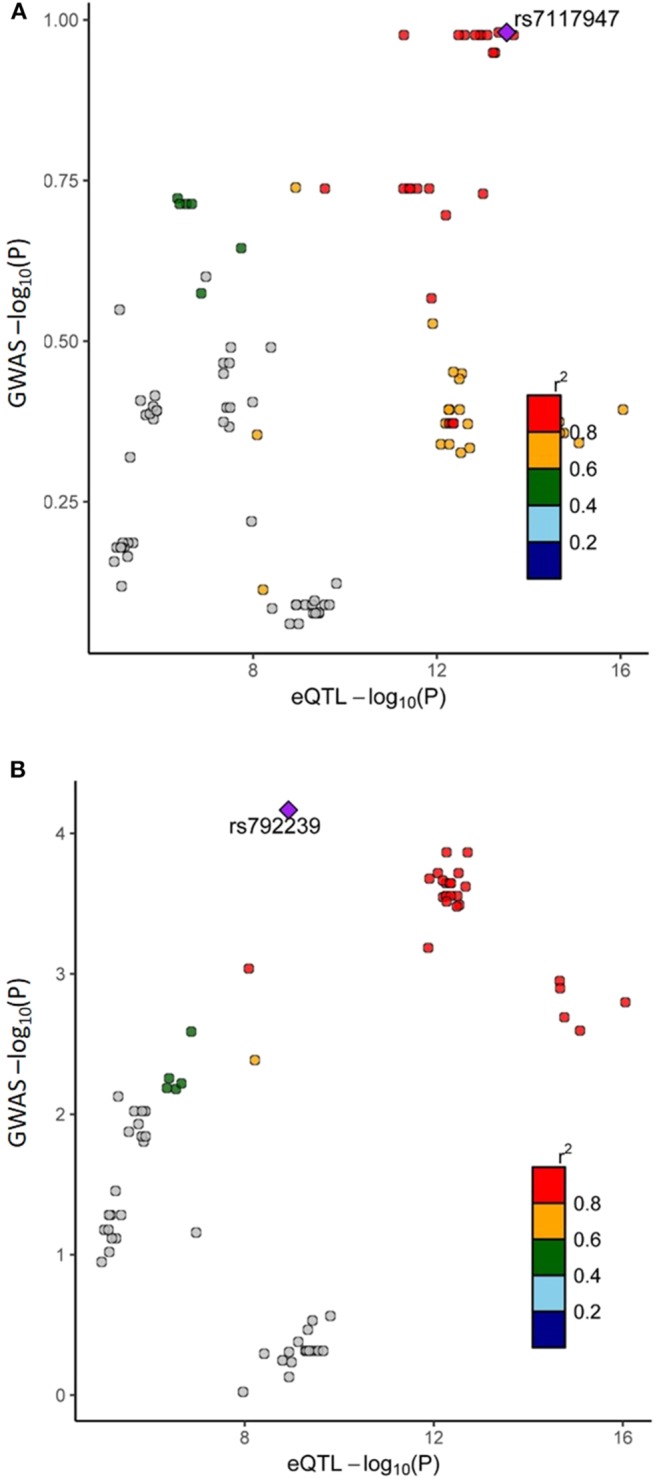
Colocalization of aGvHD GWAS and eQTL events of TRAF6 in Finnish Cohort 1 and Spanish Cohort 1. Visualization of colocalization events was performed using LocusCompareR as described in the Methods section. The *cis*-eQTL P-values of the TRAF6 locus were extracted from the eQTLGen Consortium database (http://www.eqtlgen.org) and the aGvHD-related P-values were obtained from the donor GWAS II–IV vs. 0 results. The dots in the scatter plots are colored according to their linkage disequilibrium to the colocalization lead variant. **(A)** Shows the colocalization of the eQTL and GWAS distributions of TRAF6 in Finnish Cohort 1, and **(B)** shows these distributions in Spanish Cohort 1.

**Figure 5:**
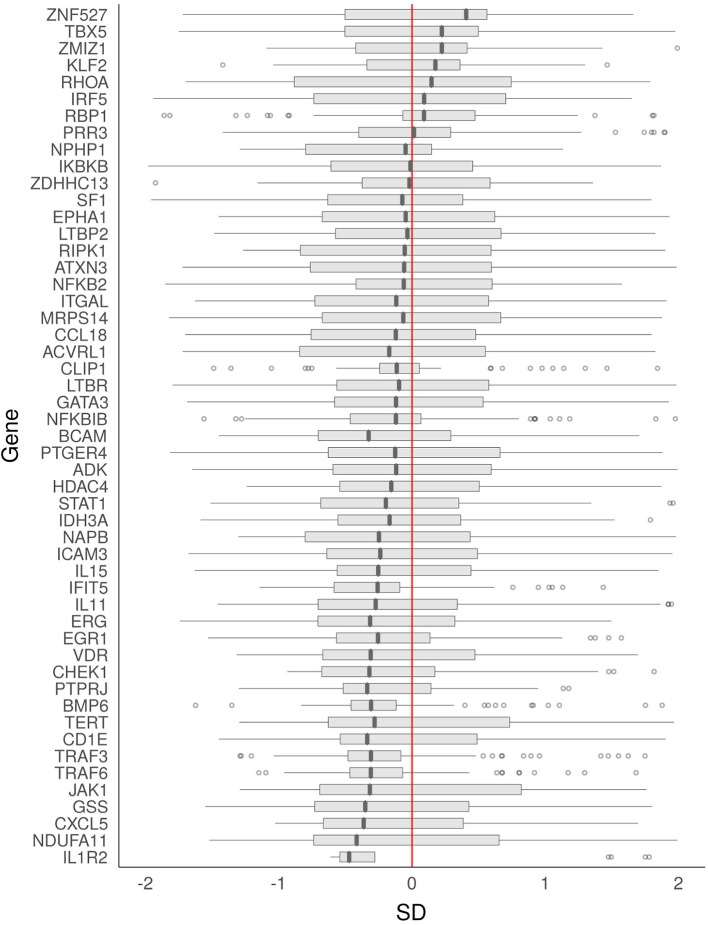
Direction and distribution of expression of aGvHD-associated genes. The effect directions were derived from all aGvHD-linked gene expression studies as described in the Methods section. The box plots represent the interquartile range (IQR) with the median, and the whiskers represent a maximum IQR of 1.5. The X-axis depicts the normalized distribution of gene expression values of cases relative to the mean of controls. SD, standard deviation.

The colocalization of GWAS and *cis*-eQTL events of the 51 aGvHD-associated genes were visualized using LocusCompareR and the results for Finnish Cohort 1, Spanish Cohort 1, and Finnish Cohort 2 are presented in Supplementary Data Sheets 2–4, respectively. The majority of events showed no direct positive colocalization signal. However, the *cis*-eQTL events of TRAF6 and the corresponding variants of aGvHD GWASs were colocalized in Finnish Cohort 1 (Figure 4A). This observation was supported by Spanish Cohort 1, although the lead variants were independent (Figure 4B). We formulated combined effect directions of gene expression for the 51 aGvHD-associated genes from the aGvHD GE data sets (Figure 5). The expression levels of TRAF6, TRAF3, IL1R2, interferon induced protein with tetratricopeptide repeats 5, and bone morphogenetic protein 6 seemed to be reduced among the aGvHD patients compared to the controls.

### Data and Code Availability

The limitations of ethical permits restrict the public distribution of personal data including individual genetic data. The code for the meta-analyses is publicly available in GitHub (https://github.com/FRCBS/GvHD_meta).

## Discussion

In this study, we performed a novel gene-level meta-analysis on GvHD by integrating GWAS data and gene expression results from different populations and study settings. We discovered pathways associated with aGvHD pathogenesis, implicating immunological responses in processes such as T cell function, cytokines, JAK-STAT signaling, and regulation of the TRAF6 gene.

The risk for GvHD has mainly been investigated during past decades by concentrating on the MHC region and specific candidate genes (10, 11), and only in recent years has the use of genome-wide approaches emerged in the field (16, 17, 19–22). These studies have shown the importance of genetic component in HSCT complications, but the results remain diverse, and their replication is incomplete. Even though individual gene expression studies can yield several differentially expressed genes, the dynamic nature of gene expression and heterogeneity in the treatment settings and genetic and environmental backgrounds of the subjects may make generalization from a single study difficult. Additionally, interpreting the functionality and downstream effects of disease-associated GWAS variants has generally been problematic because the majority of detected polymorphisms fall within non-coding regions of the genome. Our results highlight the strength of extensive meta-analysis; neither the independent adjusted GWASs nor the separate meta-analyses of gene expression or GWAS yielded statistically significant results alone. In contrast, the full data combination produced biologically meaningful genes and biological processes that were confirmed by permutation analysis. Thus, these results may indicate genes that have an impact on GvHD regardless of the various differences between the cohorts.

In the present study, the 51 aGvHD-associated genes included several prominent immunological effectors, supporting their role in GvHD pathogenesis. Many of these genes have been linked with GvHD in previous studies [Supplementary Table 5 (Supplementary Data Sheet 1)]. Connection with aGvHD has been demonstrated for telomerase reverse transcriptase (TERT), IL11, IL15, Kruppel like factor 2 (KLF2), STAT1, interferon regulatory factor 5 (IRF5), histone deacetylase 4 (HDAC4), JAK1, receptor interacting serine/threonine kinase 1 (RIPK1), TRAF3, and TRAF6 (45–58). As most of these genes have been studied mainly in murine models, their detection by our meta-analysis supports the role of these immune effectors in humans, and therefore provides candidates for potential drug targets. For instance, HDAC inhibitor Vorinostat has been shown to be promising in phase I/II trials (28784598).

Many of the implicated genes are parts of key immunoregulatory pathways such as JAK-STAT, TNF, and nuclear factor kappa B (NFκB). JAK1, an essential kinase for cytokine receptor signaling molecules, and STAT1, an activating transcription factor in response to pathogens, were involved in 33% of the significantly enriched biological processes. Importantly, JAK-STAT signaling assembles a connective molecular mechanism for multiple observations involving IL11, IL15 and other cytokines and links these results to the aGvHD-predisposing cytokine storm. Currently, the JAK1/2 inhibitor ruxolitinib is considered a potent salvage therapy for corticosteroid-refractory GvDH (59) and is being studied in a prospective randomized phase 2 trial (NCT02396628). The role of TNF signaling was also evident in the results; the herpesvirus entry mediator (HVEM), a member of the TNF receptor superfamily, is expressed on T-cells, and soluble LTBR protein has been shown to compete with HVEM receptor to inhibit T-cell activity and prevent GvHD in murine models. The RIPK1 receptor interacting serine/threonine kinase 1 is a downstream effector of TNFR2 (58), the activation of which has been shown to protect from aGvHD in a murine model and act concordantly in human cells *in vitro* (57). TNF down-stream signaling and transcription are regulated by TRAFs (50, 60). Three genes identified by the present study are closely involved with the NFκB pathway, a central regulator of cytokine production: NFκB inhibitor β, inhibitor of NFκB2 kinase subunit β, and NFκB2. Even though NFκB inhibition has not been successful in human trials, the important role of this pathway in immunological functions may warrant further studies in members of its signaling network.

The biologically active form of vitamin D, 1,25-dihydxoyvitamin D3, has been reported to exert beneficial immunosuppressive modulation via binding to the nuclear VDR (61). In an HSCT setting, vitamin D deficiency is considered a common complaint that may affect the treatment outcome (62). Genetic polymorphisms of the VDR have been shown to alter the immunomodulatory effect of vitamin D (63). Consistent with these observations, our results suggest a role for VDR in aGvHD-pathogenesis and present 24 VDR-associated biological processes. A possible mechanism could involve a positive effect of vitamin D on telomere length of blood cells directly or via increasing sex hormone levels, supporting proliferative capacity, reconstitution, and long-term immunological functionality of the graft (64–66). This hypothesis is supported by our results identifying TERT as significantly decreased in severe aGvHD, and enrichment of GOs involving VDR in hormone metabolism.

eQTL databases may be utilized to detect downstream effects of variants on gene expression. However, due to the abundance of *cis*-eQTL data, generating causal hypotheses for functional mechanisms of variants has been strongly affected by the high false-positive rate (43). To overcome this limitation, the colocalization analysis visualizes the distributions of eQTL and GWAS signals instead of focusing solely on the lead variants. Our results suggest colocalization of *cis*-eQTL events of TRAF6 and the corresponding variants of aGvHD in GWASs, indicating potential functional importance. TRAF6 has been identified as an important nuclear factor κB activator and an essential mediator for the GvHD-suppressing ability of thymic-derived regulatory T cells (49). Our results showing reduced TRAF6 expression among aGvHD patients are in consistent with this result. In contrast, high levels of TRAF6 have been associated with increased GvHD severity in mice (50). TRAF6 is regulated by miR-146a (67) and together with TRAF3, which was also implicated by our analysis, is a downstream effector of TLR molecules (47) involved in innate immunity-driven pathogenesis of GvHD (48).

There were several limitations of the study. The HSCT study cohorts were treated and collected over a long period of time in different centers, rendering treatment protocols heterogeneous. The numbers of cases were low in the separate GWASs and the case-control balance was therefore hardly optimal. Moreover, the gene expression studies were very heterogeneous in their study designs and sample collection procedures, and the overall number of samples was relatively low. This is to some extent mitigated by the employed meta-analysis method which was designed for noisy and heterogeneous data (40). Our approach was also not appropriate for analyzing the complex relationship between genetic variants implicated by GWAS and expression of the corresponding genes. To determine the effect directions of gene expression for GWASs, a transcriptome-wide association study method (68) would be required, but this could not be pursued here due to lack of appropriate reference material. This approach could be improved in future studies by using larger gene expression data set with more shared genetic variants. While the data we used were from Caucasian populations, it would be important to extend these analyses into other populations. Single nucleotide polymorphisms may vary among the populations which may impact the gene-level summary of the GWASs or regulation of gene expression.

We obtained no results for cGvHD, which may be due to the smaller number of available studies or lack of strong genetic component in this condition. We recently reported that the severity of cGvHD is associated with the overall immunogenetic differences between HSCT pairs (22); hence, its genetic background may differ from that of aGvHD.

We conclude that the meta-analysis of varied data types and populations identified common gene-level effects underlying the development of acute GvHD. As our approach was able to discover several previously known genes and immunologically relevant functional categories, it may be applied in different setting and to other data types to help identify novel genes and pathways.

## Data Availability Statement

The datasets generated for this study will not be made publicly available because the limitations of ethical permits restrict the public distribution of personal data including individual genetic data.

## Ethics Statement

The studies involving human participants were reviewed and approved by the Finnish National Supervisory Authority for Welfare (Dnro V/74832/2017, V/3235/2019) and Health and the ethics committees of Helsinki University Central Hospital (382/13/03/01/2014, HUS/114/2018) and Turku University Central Hospital (ETMK 78/2012). Samples and data from Spanish patients included in this study were provided by the IDIBGI Biobank (Biobanc IDIBGI, B.0000872), integrated in the Spanish National Biobanks Network, and they were processed following standard operating procedures with the appropriate approval of the ethics and scientific committees. Written informed consent from the participants' legal guardian/next of kin was not required to participate in this study in accordance with the national legislation and the institutional requirements.

## Author Contributions

KH, SK, JP, and JR designed the study. LV, RN, and AN evaluated the clinical end points of Finnish Cohort 1, and MI-R, US, and MP evaluated Finnish Cohort 2. DG and IB provided the DNA samples and clinical data for Spanish Cohort 1. SK managed all DNA samples, verified HLA assignations and performed the preliminary GWAS of Finnish Cohort 1. KH performed the genomic imputation, PCA, GWASs, and logistic regression analyses. JR performed the meta-analyses, detection of gene expression effect directions, and visualization of co-localization. KH and JR interpreted the results and drafted the manuscript. All authors critically revised the final version of the manuscript and approved its submission for the publication.

### Conflict of Interest

The authors declare that the research was conducted in the absence of any commercial or financial relationships that could be construed as a potential conflict of interest.
