# Supplementary material for: Meta-Analysis of Genome-Wide Association and Gene Expression Studies Implicates Donor T Cell Function and Cytokine Pathways in Acute GvHD
Source: Front Immunol. 2020 Feb 3;11:19. doi: 10.3389/fimmu.2020.00019 (PMC7008714; doi:10.3389/fimmu.2020.00019)
Supplement: Supplementary file 1 [file Data_Sheet_1.docx]

SUPPLEMENTARY FILE 1

Meta-analysis of GWAS and gene expression studies implicates donor T cell function and cytokine pathways in acute GvHD

Short title: GvHD meta-analysis

Kati Hyvärinen^1^, Satu Koskela^1^, Riitta Niittyvuopio^2^, Anne Nihtinen^2^, Liisa Volin^2^, Urpu Salmenniemi^3^, Mervi Putkonen^3^, Ismael Buño^4^, David Gallardo^5^, Maija Itälä-Remes^3^, Jukka Partanen^1^, Jarmo Ritari^1^

1. Finnish Red Cross Blood Service, Helsinki, Finland; 2. Helsinki University Hospital, Comprehensive Cancer Center, Stem Cell Transplantation Unit, Helsinki, Finland; 3. Turku University Hospital, Turku, Finland; 4. Department of Hematology, Genomics Unit, Hosp. G.U. Gregorio Marañón, IiSGM, Madrid, Spain; 5. Department of Hematology, Institut Català d'Oncologia, IDIBGi, Girona, Spain.

Supplemetary Tables 5

Supplementary Figures 2

References 11

**Supplementary Table 1. Association of the common risk factors with GvHD outcomes**

| Predictor | Dependent variable^a^ | Finnish Cohort 1 | | Spanish Cohort 1 | | Finnish Cohort 2 | |
| --- | --- | --- | --- | --- | --- | --- | --- |
|  |  | Odds ratio  (95% CI) ^b^ | P-value^b^ | Odds ratio  (95% CI) ^b^ | P-value^b^ | Odds ratio  (95% CI) ^b^ | P-value^b^ |
| Recipient age | aGvHD 2–4 | 1.065  (1.015–1.099) | 0.007 | 1.003  (0.984–1.022) | 0.760 | 1.017  (0.990–1.044) | 0.216 |
|  | aGvHD 3–4 | 1.087  (1.023–1.154) | 0.007 | 1.029  (0.999–1.060) | 0.059 | 1.006  (0.974–1.038) | 0.730 |
|  | cGvHD limited–extensive | 1.046  (1.018–1.075) | 0.001 | 1.009  (0.988–1.032) | 0.400 | 0.990  (0.964–1.016) | 0.452 |
|  | cGvHD extensive | 1.035  (1.002–1.068) | 0.036 | 1.020  (0.992–1.048) | 0.169 | 0.986  (0.958–1.016) | 0.362 |
| Recipient gender^c^ | aGvHD 2–4 | 0.731  (0.364–1.466) | 0.378 | 0.743  (0.440–1.256) | 0.267 | 1.670  (0.859–3.247) | 0.131 |
|  | aGvHD 3–4 | 0.595  (0.239–1.478) | 0.263 | 0.741  (0.354–1.551) | 0.426 | 1.390  (0.619–3.122) | 0.425 |
|  | cGvHD limited–extensive | 0.762  (0.442–1.313) | 0.328 | 0.363  (0.198–0.666) | 0.001 | 1.078  (0.560–2.076) | 0.823 |
|  | cGvHD extensive | 0.693  (0.362–1.324) | 0.267 | 0.269  (0.128–0.566) | 0.001 | 1.072  (0.529–2.173) | 0.846 |
| Stem cell source^d^ | aGvHD 2–4 | 1.673  (0.789–3.548) | 0.180 | 0.335  (0.071–1.574) | 0.166 | 0.400  (0.191–0.839) | 0.015 |
|  | aGvHD 3–4 | 1.106  (0.413–2.962) | 0.841 | 0.554  (0.067–4.616) | 0.585 | 0.383  (0.150–0.150) | 0.045 |
|  | cGvHD limited–extensive | 0.460  (0.261–0.809) | 0.007 | 0.622  (0.174–2.216) | 0.464 | 0.525  (0.266–1.040) | 0.065 |
|  | cGvHD extensive | 0.298  (0.154–0.576) | <0.001 | NA^e^ | 0.999 | 0.339  (0.156–0.737) | 0.006 |

aGvHD, acute graft-vs-host disease; cGvDH, chronic graft-versus-host disease; CI, confidence interval; NA, not applicable.

^a^ Graft-vs-host disease status with gradus.

^b^ Logistic regression analysis using recipient age, recipient gender, direction of transplantation, and stem cell source as covariates.

^c^ Female vs male (the reference group).

^d^ Bone marrow vs peripheral blood (the reference group).

^e^ The number of events is too low.

^b^ Logistic regression analysis using recipient age, recipient gender, stem cell source, and number of missing variants as covariates.

**Supplementary table 2. The characteristics of the gene expression studies used in meta-analysis.**

| **Refererence** | **Year** | **GEO Accession^a^** | **Disease outcome** | **Total N (affected)** | **Gene expression data, cell types** | **Donor type** | **Gene Expression Array** |
| --- | --- | --- | --- | --- | --- | --- | --- |
| Baron et al. (1) | 2007 | GSE4624 | aGvHD I-IV | CD4+ 43 (17)  CD8+ 41 (16) | Donor pre-grafts:   1. CD4^+^  T cells 2. CD8^+^ T cells | MRD | H19K |
| Glauzy et al.(2) | 2016 | GSE75344 | aGvHD II-III | 9 (5) | HSCT patients:   1. CD34^+^lin^-^CD10^+^CD24^-^ cells 2. CD34^+^lin^-^CD10^-^ cells | MRD, MUD | GeneChip Human Genome U133plus 2.0 |
| Lupsa et al.(3) | 2018 | GSE103569 | Cutaneous and/or GI aGvHD | 20 (15) | HSCT patients:   1. CD8β^+^ CLA^+^ T cells 2. CD8β^+^ ITGβ7^+^ T cells 3. CD8β^+^ CLA^–^ ITGβ7^–^ T cells | Sibling, haplo, MUD | Agilent-026652 Whole Human Genome Microarray 4x44K v2 |
| Furlan et al.(4) | 2015 | GSE73809 | aGvHD II–IV | 24 (11) | HSCT patients:   1. Cells positive for either CD4 or CD8 | MSD, MMUD | Affymetrix Human Transcriptome Array 2.0 |
| Takahashi et al.(5) | 2008 | GSE10572 | aGvHD I–II | aGvHD phase 8 (8)  Recovery phase 8 (8) | CBT patients:   1. CD8+ cells 2. CD56+ cells 3. CD4+ cells 4. CD14+ cells | URD | GPL6487 for IMShuman0.6Kv.1 and GPL6488 for IMShuman0.6Kv.2 |
| Buzzeo et al.(6) | 2008 | GSE7510 | aGvHD | 8 (4) | HSCT patients:   1. PB leukocytes | Related, MUD | Affymetrix GeneChip® Human Genome U133 Plus 2.0 |
| Baron et al.(1) | 2007 | GSE4624 | Extensive cGvHD | CD4+ 43 (28)  CD8+ 41 (26) | Donor pre-grafts:   1. CD4^+^ cells 2. CD8^+^ T cells | MRD | H19K |
| Munagala/Miller et al.(7) | 2013 | GSE56495 | cGvHD | 31(23) | HSCT patients and healthy ctrls baseline results:   1. Whole blood | ? | Illumina HumanHT-12 V4.0 expression beadchip |
| Hakim et al.(8) | 2016 | GSE60674 | Mild + extensive  cGvHD | 36 (26) | HSCT patients and healthy ctrls:   1. Monocytes: CD3- CD4dim cells, >95% CD14+ | Related, unrelated, haplo | Affymetrix Human Gene 1.0 ST Array |
| Kohrt et al.(9) | 2013 | GSE23924 | cGvHD | 63 (35) | HSCT patients:   1. PBMC | Sibling, unrelated | Agilent-014850 Whole Human Genome Microarray 4x44K G4112F |

aGvHD, acute graft-versus-host disease; CBT, cord blood transplantation; cGvHD, chronic graft-versus-host disease; GEO, Gene Expression Omnibus; GI, gastro-intestinal; MMRD, mismatched related donor; MRD, matched related donor; MSD, matched sibling donor; MUD, matched unrelated donor; PB, peripheral blood; PBMC, peripheral blood monocytes

^a^ <https://www.ncbi.nlm.nih.gov/geo/>

**Supplementary Table 3. The genes associating with aGVHD in meta-analysis.**

| **Gene^a^** | **CHR** | **P-value^b^** | **FDR^b^** |
| --- | --- | --- | --- |
| LTBR, lymphotoxin beta receptor | 12 | 1.405E-07 | 0.005 |
| IRF5, interferon induced protein with tetratricopeptide repeats 5 | 7 | 1.503E-06 | 0.027 |
| HDAC4, histone deacetylase 4 | 2 | 6.551E-06 | 0.053 |
| SF1, splicing factor 1 | 11 | 7.065E-06 | 0.053 |
| ZDHHC13, zinc finger DHHC-type containing 13 | 11 | 7.262E-06 | 0.053 |
| GSS, glutathione synthetase | 20 | 1.150E-05 | 0.053 |
| ACVRL1, activin A receptor like type 1 | 12 | 1.260E-05 | 0.053 |
| NDUFA11, NADH:ubiquinone oxidoreductase subunit A11 | 19 | 1.310E-05 | 0.053 |
| RHOA, ras homolog family member A | 3 | 1.434E-05 | 0.053 |
| LTBP2, latent transforming growth factor beta binding protein 2 | 14 | 1.452E-05 | 0.053 |
| CD1E, CD1e molecule | 1 | 1.757E-05 | 0.058 |
| IFIT5, interferon induced protein with tetratricopeptide repeats 5 | 10 | 2.174E-05 | 0.064 |
| TRAF3, TNF receptor associated factor 3 | 14 | 2.412E-05 | 0.064 |
| IL1R2, interleukin 1 receptor type 2 | 2 | 2.761E-05 | 0.064 |
| JAK1, Janus kinase 1 | 1 | 2.772E-05 | 0.064 |
| TRAF6, TNF receptor associated factor 6 | 11 | 2.811E-05 | 0.064 |
| NAPB, NSF attachment protein beta | 20 | 3.135E-05 | 0.064 |
| CCL18, C-C motif chemokine ligand 18 | 17 | 3.139E-05 | 0.064 |
| BCAM, basal cell adhesion molecule | 19 | 3.484E-05 | 0.065 |
| VDR, vitamin D receptor | 12 | 3.555E-05 | 0.065 |
| PRR3, proline rich 3 | 6 | 4.225E-05 | 0.071 |
| CHEK1, checkpoint kinase 1 | 11 | 4.486E-05 | 0.071 |
| IL11, interleukin 11 | 19 | 4.548E-05 | 0.071 |
| ERG, ETS transcription factor ERG | 21 | 4.682E-05 | 0.071 |
| IDH3A, isocitrate dehydrogenase (NAD(+)) 3 alpha | 15 | 4.836E-05 | 0.071 |
| TERT, telomerase reverse transcriptase | 5 | 5.072E-05 | 0.071 |
| BMP6, bone morphogenetic protein 6 | 6 | 5.758E-05 | 0.078 |
| EGR1, early growth response 1 | 5 | 6.269E-05 | 0.082 |
| PTPRJ, protein tyrosine phosphatase receptor type J | 11 | 7.702E-05 | 0.097 |
| NPHP1, nephrocystin 1 | 2 | 8.238E-05 | 0.097 |
| NFKBIB, NFKB inhibitor beta | 19 | 9.232E-05 | 0.097 |
| IKBKB, inhibitor of nuclear factor kappa B kinase subunit beta | 8 | 9.476E-05 | 0.097 |
| NFKB2, nuclear factor kappa B subunit 2 | 10 | 9.590E-05 | 0.097 |
| CLIP1, CAP-Gly domain containing linker protein 1 | 12 | 9.860E-05 | 0.097 |
| MRPS14, mitochondrial ribosomal protein S14 | 1 | 1.048E-04 | 0.097 |
| ICAM3, intercellular adhesion molecule 3 | 19 | 1.076E-04 | 0.097 |
| EPHA1, EPH receptor A1 | 7 | 1.077E-04 | 0.097 |
| STAT1, signal transducer and activator of transcription 1 | 2 | 1.082E-04 | 0.097 |
| KLF2, Kruppel like factor 2 | 19 | 1.108E-04 | 0.097 |
| RBP1, retinol binding protein 1 | 3 | 1.110E-04 | 0.097 |
| ADK, adenosine kinase | 10 | 1.131E-04 | 0.097 |
| IL15, interleukin 15 | 4 | 1.144E-04 | 0.097 |
| RIPK1, receptor interacting serine/threonine kinase 1 | 6 | 1.150E-04 | 0.097 |
| ATXN3, ataxin 3 | 14 | 1.284E-04 | 0.098 |
| TBX5, T-box transcription factor 5 | 12 | 1.302E-04 | 0.098 |
| CXCL5, C-X-C motif chemokine ligand 5 | 4 | 1.320E-04 | 0.098 |
| ITGAL, integrin subunit alpha L | 16 | 1.324E-04 | 0.098 |
| ZNF527, zinc finger protein 527 | 19 | 1.327E-04 | 0.098 |
| PTGER4, prostaglandin E receptor 4 | 15 | 1.328E-04 | 0.098 |
| GATA3, GATA binding protein 3 | 10 | 1.349E-04 | 0.098 |
| ZMIZ1, zinc finger MIZ-type containing 1 | 10 | 1.392E-04 | 0.100 |

CHR, chromosome; FDR, false detection rate.

^a^ NCBI gene annotation database <https://www.ncbi.nlm.nih.gov/gene>

^b^ Robust rank aggregation meta-analysis(10)

**Supplementary Table 4. The gene ontologies associating with aGVHD.**

| **Gene Ontolody^a^** | **Description^a^** | **GeneRatio^a^** | **BgRatio^a^** | **P-value^a^** | **Adjusted P-value^a^** | **FDR^a^** | **geneID^a^** | **Gene count^a^** | **GeneBgRatio^a^** |
| --- | --- | --- | --- | --- | --- | --- | --- | --- | --- |
| GO:0002521 | leukocyte differentiation | 2.45E-01 | 2.80E-02 | 7.36E-09 | 1.48E-05 | 9.76E-06 | LTBR/HDAC4/RHOA/TRAF6/IL11/EGR1/PTPRJ/IL15/RIPK1/PTGER4/GATA3/ZMIZ1 | 12 | 9 |
| GO:0034612 | response to tumor necrosis factor | 1.84E-01 | 1.63E-02 | 8.27E-08 | 8.29E-05 | 5.49E-05 | LTBR/HDAC4/GSS/TRAF3/IKBKB/STAT1/KLF2/RIPK1/GATA3 | 9 | 11 |
| GO:0043122 | regulation of I-kappaB kinase/NF-kappaB signaling | 1.63E-01 | 1.28E-02 | 1.85E-07 | 1.15E-04 | 7.59E-05 | LTBR/ZDHHC13/RHOA/IFIT5/TRAF6/IKBKB/STAT1/RIPK1 | 8 | 13 |
| GO:0033002 | muscle cell proliferation | 1.63E-01 | 1.36E-02 | 2.86E-07 | 1.15E-04 | 7.59E-05 | HDAC4/SF1/TRAF6/TERT/STAT1/IL15/RIPK1/TBX5 | 8 | 12 |
| GO:0030098 | lymphocyte differentiation | 1.84E-01 | 1.88E-02 | 2.76E-07 | 1.15E-04 | 7.59E-05 | HDAC4/RHOA/IL11/EGR1/PTPRJ/IL15/PTGER4/GATA3/ZMIZ1 | 9 | 10 |
| GO:0001819 | positive regulation of cytokine production | 2.04E-01 | 2.56E-02 | 3.67E-07 | 1.23E-04 | 8.10E-05 | IRF5/TRAF6/EGR1/PTPRJ/NFKB2/STAT1/IL15/RIPK1/PTGER4/GATA3 | 10 | 8 |
| GO:0043123 | positive regulation of I-kappaB kinase/NF-kappaB signaling | 1.43E-01 | 9.94E-03 | 5.12E-07 | 1.28E-04 | 8.49E-05 | LTBR/ZDHHC13/RHOA/IFIT5/TRAF6/IKBKB/RIPK1 | 7 | 14 |
| GO:0007249 | I-kappaB kinase/NF-kappaB signaling | 1.63E-01 | 1.45E-02 | 4.72E-07 | 1.28E-04 | 8.49E-05 | LTBR/ZDHHC13/RHOA/IFIT5/TRAF6/IKBKB/STAT1/RIPK1 | 8 | 11 |
| GO:1903708 | positive regulation of hemopoiesis | 1.43E-01 | 1.05E-02 | 7.49E-07 | 1.50E-04 | 9.94E-05 | RHOA/TRAF6/STAT1/IL15/RIPK1/GATA3/ZMIZ1 | 7 | 14 |
| GO:0071356 | cellular response to tumor necrosis factor | 1.63E-01 | 1.53E-02 | 6.92E-07 | 1.50E-04 | 9.94E-05 | LTBR/HDAC4/TRAF3/IKBKB/STAT1/KLF2/RIPK1/GATA3 | 8 | 11 |
| GO:0009612 | response to mechanical stimulus | 1.43E-01 | 1.15E-02 | 1.36E-06 | 2.49E-04 | 1.64E-04 | LTBR/HDAC4/RHOA/CHEK1/BMP6/STAT1/PTGER4 | 7 | 12 |
| GO:0071260 | cellular response to mechanical stimulus | 1.02E-01 | 4.39E-03 | 2.34E-06 | 3.91E-04 | 2.59E-04 | LTBR/HDAC4/CHEK1/BMP6/PTGER4 | 5 | 23 |
| GO:1902107 | positive regulation of leukocyte differentiation | 1.22E-01 | 8.26E-03 | 2.99E-06 | 4.61E-04 | 3.05E-04 | RHOA/TRAF6/IL15/RIPK1/GATA3/ZMIZ1 | 6 | 15 |
| GO:0032350 | regulation of hormone metabolic process | 8.16E-02 | 2.20E-03 | 3.90E-06 | 5.48E-04 | 3.62E-04 | VDR/BMP6/EGR1/GATA3 | 4 | 37 |
| GO:0030856 | regulation of epithelial cell differentiation | 1.22E-01 | 8.72E-03 | 4.10E-06 | 5.48E-04 | 3.62E-04 | ACVRL1/VDR/BMP6/IKBKB/STAT1/GATA3 | 6 | 14 |
| GO:1901888 | regulation of cell junction assembly | 1.02E-01 | 5.20E-03 | 5.41E-06 | 6.78E-04 | 4.48E-04 | ACVRL1/RHOA/PTPRJ/NPHP1/IKBKB | 5 | 20 |
| GO:0048660 | regulation of smooth muscle cell proliferation | 1.22E-01 | 9.65E-03 | 7.31E-06 | 8.63E-04 | 5.71E-04 | HDAC4/SF1/TRAF6/TERT/STAT1/IL15 | 6 | 13 |
| GO:0048659 | smooth muscle cell proliferation | 1.22E-01 | 9.76E-03 | 7.83E-06 | 8.72E-04 | 5.77E-04 | HDAC4/SF1/TRAF6/TERT/STAT1/IL15 | 6 | 13 |
| GO:0032352 | positive regulation of hormone metabolic process | 6.12E-02 | 8.67E-04 | 9.47E-06 | 1.00E-03 | 6.61E-04 | BMP6/EGR1/GATA3 | 3 | 71 |
| GO:0070555 | response to interleukin-1 | 1.22E-01 | 1.06E-02 | 1.23E-05 | 1.24E-03 | 8.18E-04 | HDAC4/IL1R2/TRAF6/EGR1/IKBKB/KLF2 | 6 | 12 |
| GO:1901342 | regulation of vasculature development | 1.63E-01 | 2.36E-02 | 1.72E-05 | 1.63E-03 | 1.08E-03 | ACVRL1/RHOA/JAK1/TERT/EGR1/EPHA1/STAT1/KLF2 | 8 | 7 |
| GO:0030099 | myeloid cell differentiation | 1.63E-01 | 2.37E-02 | 1.78E-05 | 1.63E-03 | 1.08E-03 | LTBR/TRAF6/IL11/STAT1/KLF2/IL15/RIPK1/GATA3 | 8 | 7 |
| GO:0002237 | response to molecule of bacterial origin | 1.43E-01 | 1.77E-02 | 2.34E-05 | 1.96E-03 | 1.29E-03 | IRF5/RHOA/TRAF6/BMP6/NFKBIB/NFKB2/PTGER4 | 7 | 8 |
| GO:0071214 | cellular response to abiotic stimulus | 1.43E-01 | 1.79E-02 | 2.44E-05 | 1.96E-03 | 1.29E-03 | LTBR/HDAC4/CHEK1/BMP6/EGR1/PTGER4/GATA3 | 7 | 8 |
| GO:0104004 | cellular response to environmental stimulus | 1.43E-01 | 1.79E-02 | 2.44E-05 | 1.96E-03 | 1.29E-03 | LTBR/HDAC4/CHEK1/BMP6/EGR1/PTGER4/GATA3 | 7 | 8 |
| GO:0072111 | cell proliferation involved in kidney development | 6.12E-02 | 1.21E-03 | 2.74E-05 | 2.01E-03 | 1.33E-03 | EGR1/STAT1/GATA3 | 3 | 50 |
| GO:0007160 | cell-matrix adhesion | 1.22E-01 | 1.22E-02 | 2.84E-05 | 2.01E-03 | 1.33E-03 | ACVRL1/RHOA/BCAM/PTPRJ/EPHA1/ITGAL | 6 | 10 |
| GO:0071496 | cellular response to external stimulus | 1.43E-01 | 1.84E-02 | 2.93E-05 | 2.01E-03 | 1.33E-03 | LTBR/HDAC4/VDR/CHEK1/BMP6/IL15/PTGER4 | 7 | 8 |
| GO:0009615 | response to virus | 1.43E-01 | 1.85E-02 | 3.05E-05 | 2.01E-03 | 1.33E-03 | IRF5/IFIT5/TRAF3/IKBKB/STAT1/IL15/GATA3 | 7 | 8 |
| GO:0031589 | cell-substrate adhesion | 1.43E-01 | 1.85E-02 | 3.05E-05 | 2.01E-03 | 1.33E-03 | ACVRL1/RHOA/BCAM/PTPRJ/EPHA1/ATXN3/ITGAL | 7 | 8 |
| GO:0042110 | T cell activation | 1.63E-01 | 2.56E-02 | 3.10E-05 | 2.01E-03 | 1.33E-03 | RHOA/TRAF6/EGR1/IL15/ITGAL/PTGER4/GATA3/ZMIZ1 | 8 | 6 |
| GO:0046677 | response to antibiotic | 1.43E-01 | 1.87E-02 | 3.30E-05 | 2.07E-03 | 1.37E-03 | RHOA/JAK1/EGR1/STAT1/KLF2/RIPK1/GATA3 | 7 | 8 |
| GO:0002831 | regulation of response to biotic stimulus | 1.02E-01 | 7.97E-03 | 4.31E-05 | 2.55E-03 | 1.69E-03 | TRAF3/TRAF6/BMP6/STAT1/IL15 | 5 | 13 |
| GO:2000146 | negative regulation of cell motility | 1.43E-01 | 1.95E-02 | 4.32E-05 | 2.55E-03 | 1.69E-03 | ACVRL1/RHOA/PTPRJ/EPHA1/TBX5/PTGER4/GATA3 | 7 | 7 |
| GO:0030217 | T cell differentiation | 1.22E-01 | 1.35E-02 | 4.93E-05 | 2.82E-03 | 1.87E-03 | RHOA/EGR1/IL15/PTGER4/GATA3/ZMIZ1 | 6 | 9 |
| GO:0046885 | regulation of hormone biosynthetic process | 6.12E-02 | 1.56E-03 | 5.95E-05 | 3.25E-03 | 2.15E-03 | VDR/BMP6/EGR1 | 3 | 39 |
| GO:0051271 | negative regulation of cellular component movement | 1.43E-01 | 2.06E-02 | 6.00E-05 | 3.25E-03 | 2.15E-03 | ACVRL1/RHOA/PTPRJ/EPHA1/TBX5/PTGER4/GATA3 | 7 | 7 |
| GO:0035666 | TRIF-dependent toll-like receptor signaling pathway | 6.12E-02 | 1.68E-03 | 7.40E-05 | 3.71E-03 | 2.45E-03 | TRAF3/IKBKB/RIPK1 | 3 | 37 |
| GO:0045765 | regulation of angiogenesis | 1.43E-01 | 2.11E-02 | 7.14E-05 | 3.71E-03 | 2.45E-03 | ACVRL1/RHOA/JAK1/TERT/EPHA1/STAT1/KLF2 | 7 | 7 |
| GO:0040013 | negative regulation of locomotion | 1.43E-01 | 2.13E-02 | 7.39E-05 | 3.71E-03 | 2.45E-03 | ACVRL1/RHOA/PTPRJ/EPHA1/TBX5/PTGER4/GATA3 | 7 | 7 |
| GO:0071347 | cellular response to interleukin-1 | 1.02E-01 | 9.01E-03 | 7.72E-05 | 3.78E-03 | 2.50E-03 | IL1R2/TRAF6/EGR1/IKBKB/KLF2 | 5 | 11 |
| GO:0016579 | protein deubiquitination | 1.22E-01 | 1.48E-02 | 8.10E-05 | 3.87E-03 | 2.56E-03 | RHOA/TRAF3/TRAF6/RIPK1/ATXN3/GATA3 | 6 | 8 |
| GO:0033209 | tumor necrosis factor-mediated signaling pathway | 1.02E-01 | 9.53E-03 | 1.01E-04 | 4.55E-03 | 3.01E-03 | LTBR/TRAF3/IKBKB/STAT1/RIPK1 | 5 | 11 |
| GO:1902105 | regulation of leukocyte differentiation | 1.22E-01 | 1.54E-02 | 1.02E-04 | 4.55E-03 | 3.01E-03 | RHOA/TRAF6/IL15/RIPK1/GATA3/ZMIZ1 | 6 | 8 |
| GO:0045785 | positive regulation of cell adhesion | 1.43E-01 | 2.23E-02 | 9.96E-05 | 4.55E-03 | 3.01E-03 | RHOA/TRAF6/PTPRJ/EPHA1/IL15/GATA3/ZMIZ1 | 7 | 6 |
| GO:0002756 | MyD88-independent toll-like receptor signaling pathway | 6.12E-02 | 1.91E-03 | 1.10E-04 | 4.58E-03 | 3.03E-03 | TRAF3/IKBKB/RIPK1 | 3 | 32 |
| GO:0001959 | regulation of cytokine-mediated signaling pathway | 1.02E-01 | 9.71E-03 | 1.10E-04 | 4.58E-03 | 3.03E-03 | IL1R2/JAK1/IKBKB/STAT1/RIPK1 | 5 | 11 |
| GO:0070646 | protein modification by small protein removal | 1.22E-01 | 1.55E-02 | 1.06E-04 | 4.58E-03 | 3.03E-03 | RHOA/TRAF3/TRAF6/RIPK1/ATXN3/GATA3 | 6 | 8 |
| GO:0060337 | type I interferon signaling pathway | 8.16E-02 | 5.26E-03 | 1.26E-04 | 4.97E-03 | 3.29E-03 | IRF5/JAK1/EGR1/STAT1 | 4 | 16 |
| GO:0071357 | cellular response to type I interferon | 8.16E-02 | 5.26E-03 | 1.26E-04 | 4.97E-03 | 3.29E-03 | IRF5/JAK1/EGR1/STAT1 | 4 | 16 |
| GO:0031098 | stress-activated protein kinase signaling cascade | 1.22E-01 | 1.59E-02 | 1.22E-04 | 4.97E-03 | 3.29E-03 | LTBR/RHOA/TRAF6/IKBKB/RIPK1/PTGER4 | 6 | 8 |
| GO:0045621 | positive regulation of lymphocyte differentiation | 8.16E-02 | 5.37E-03 | 1.38E-04 | 5.20E-03 | 3.44E-03 | RHOA/IL15/GATA3/ZMIZ1 | 4 | 15 |
| GO:0043900 | regulation of multi-organism process | 1.43E-01 | 2.35E-02 | 1.36E-04 | 5.20E-03 | 3.44E-03 | HDAC4/IFIT5/TRAF3/TRAF6/BMP6/STAT1/IL15 | 7 | 6 |
| GO:0034340 | response to type I interferon | 8.16E-02 | 5.49E-03 | 1.49E-04 | 5.44E-03 | 3.60E-03 | IRF5/JAK1/EGR1/STAT1 | 4 | 15 |
| GO:0050810 | regulation of steroid biosynthetic process | 8.16E-02 | 5.49E-03 | 1.49E-04 | 5.44E-03 | 3.60E-03 | SF1/VDR/BMP6/EGR1 | 4 | 15 |
| GO:0060759 | regulation of response to cytokine stimulus | 1.02E-01 | 1.05E-02 | 1.55E-04 | 5.57E-03 | 3.68E-03 | IL1R2/JAK1/IKBKB/STAT1/RIPK1 | 5 | 10 |
| GO:0032496 | response to lipopolysaccharide | 1.22E-01 | 1.69E-02 | 1.70E-04 | 5.97E-03 | 3.95E-03 | RHOA/TRAF6/BMP6/NFKBIB/NFKB2/PTGER4 | 6 | 7 |
| GO:0048661 | positive regulation of smooth muscle cell proliferation | 8.16E-02 | 5.72E-03 | 1.75E-04 | 6.05E-03 | 4.01E-03 | HDAC4/TRAF6/TERT/STAT1 | 4 | 14 |
| GO:0070498 | interleukin-1-mediated signaling pathway | 8.16E-02 | 5.84E-03 | 1.89E-04 | 6.43E-03 | 4.25E-03 | IL1R2/TRAF6/EGR1/IKBKB | 4 | 14 |
| GO:0045601 | regulation of endothelial cell differentiation | 6.12E-02 | 2.37E-03 | 2.11E-04 | 6.94E-03 | 4.59E-03 | ACVRL1/BMP6/IKBKB | 3 | 26 |
| GO:0045088 | regulation of innate immune response | 1.43E-01 | 2.52E-02 | 2.11E-04 | 6.94E-03 | 4.59E-03 | TRAF3/JAK1/TRAF6/IKBKB/ICAM3/STAT1/RIPK1 | 7 | 6 |
| GO:0050870 | positive regulation of T cell activation | 1.02E-01 | 1.14E-02 | 2.36E-04 | 7.63E-03 | 5.05E-03 | RHOA/TRAF6/IL15/GATA3/ZMIZ1 | 5 | 9 |
| GO:0010810 | regulation of cell-substrate adhesion | 1.02E-01 | 1.15E-02 | 2.42E-04 | 7.69E-03 | 5.09E-03 | ACVRL1/RHOA/PTPRJ/EPHA1/ATXN3 | 5 | 9 |
| GO:0007568 | aging | 1.22E-01 | 1.81E-02 | 2.47E-04 | 7.72E-03 | 5.11E-03 | GSS/CHEK1/TERT/NFKB2/IL15/ZMIZ1 | 6 | 7 |
| GO:0002573 | myeloid leukocyte differentiation | 1.02E-01 | 1.16E-02 | 2.53E-04 | 7.81E-03 | 5.16E-03 | LTBR/TRAF6/IL15/RIPK1/GATA3 | 5 | 9 |
| GO:0043124 | negative regulation of I-kappaB kinase/NF-kappaB signaling | 6.12E-02 | 2.60E-03 | 2.78E-04 | 8.33E-03 | 5.51E-03 | RHOA/STAT1/RIPK1 | 3 | 24 |
| GO:0045058 | T cell selection | 6.12E-02 | 2.60E-03 | 2.78E-04 | 8.33E-03 | 5.51E-03 | RHOA/IL15/GATA3 | 3 | 24 |
| GO:0030336 | negative regulation of cell migration | 1.22E-01 | 1.87E-02 | 2.87E-04 | 8.46E-03 | 5.60E-03 | ACVRL1/RHOA/PTPRJ/EPHA1/TBX5/PTGER4 | 6 | 7 |
| GO:0007159 | leukocyte cell-cell adhesion | 1.22E-01 | 1.89E-02 | 3.06E-04 | 8.90E-03 | 5.89E-03 | RHOA/TRAF6/IL15/ITGAL/GATA3/ZMIZ1 | 6 | 6 |
| GO:0001952 | regulation of cell-matrix adhesion | 8.16E-02 | 6.64E-03 | 3.11E-04 | 8.91E-03 | 5.90E-03 | ACVRL1/RHOA/PTPRJ/EPHA1 | 4 | 12 |
| GO:1903706 | regulation of hemopoiesis | 1.43E-01 | 2.69E-02 | 3.17E-04 | 8.94E-03 | 5.91E-03 | RHOA/TRAF6/STAT1/IL15/RIPK1/GATA3/ZMIZ1 | 7 | 5 |
| GO:0042445 | hormone metabolic process | 1.02E-01 | 1.22E-02 | 3.24E-04 | 8.96E-03 | 5.93E-03 | VDR/BMP6/EGR1/RBP1/GATA3 | 5 | 8 |
| GO:0034329 | cell junction assembly | 1.02E-01 | 1.23E-02 | 3.31E-04 | 8.96E-03 | 5.93E-03 | ACVRL1/RHOA/PTPRJ/NPHP1/IKBKB | 5 | 8 |
| GO:1903039 | positive regulation of leukocyte cell-cell adhesion | 1.02E-01 | 1.23E-02 | 3.31E-04 | 8.96E-03 | 5.93E-03 | RHOA/TRAF6/IL15/GATA3/ZMIZ1 | 5 | 8 |
| GO:0071104 | response to interleukin-9 | 4.08E-02 | 5.78E-04 | 3.48E-04 | 9.19E-03 | 6.08E-03 | JAK1/STAT1 | 2 | 71 |
| GO:0072203 | cell proliferation involved in metanephros development | 4.08E-02 | 5.78E-04 | 3.48E-04 | 9.19E-03 | 6.08E-03 | EGR1/STAT1 | 2 | 71 |
| GO:0046886 | positive regulation of hormone biosynthetic process | 4.08E-02 | 6.36E-04 | 4.25E-04 | 1.01E-02 | 6.67E-03 | BMP6/EGR1 | 2 | 64 |
| GO:0070106 | interleukin-27-mediated signaling pathway | 4.08E-02 | 6.36E-04 | 4.25E-04 | 1.01E-02 | 6.67E-03 | JAK1/STAT1 | 2 | 64 |
| GO:0070757 | interleukin-35-mediated signaling pathway | 4.08E-02 | 6.36E-04 | 4.25E-04 | 1.01E-02 | 6.67E-03 | JAK1/STAT1 | 2 | 64 |
| GO:2000615 | regulation of histone H3-K9 acetylation | 4.08E-02 | 6.36E-04 | 4.25E-04 | 1.01E-02 | 6.67E-03 | CHEK1/GATA3 | 2 | 64 |
| GO:0019218 | regulation of steroid metabolic process | 8.16E-02 | 7.11E-03 | 4.02E-04 | 1.01E-02 | 6.67E-03 | SF1/VDR/BMP6/EGR1 | 4 | 11 |
| GO:0032479 | regulation of type I interferon production | 8.16E-02 | 7.11E-03 | 4.02E-04 | 1.01E-02 | 6.67E-03 | IRF5/TRAF3/NFKB2/STAT1 | 4 | 11 |
| GO:0003158 | endothelium development | 8.16E-02 | 7.16E-03 | 4.14E-04 | 1.01E-02 | 6.67E-03 | ACVRL1/RHOA/BMP6/IKBKB | 4 | 11 |
| GO:0032606 | type I interferon production | 8.16E-02 | 7.22E-03 | 4.27E-04 | 1.01E-02 | 6.67E-03 | IRF5/TRAF3/NFKB2/STAT1 | 4 | 11 |
| GO:1904018 | positive regulation of vasculature development | 1.02E-01 | 1.30E-02 | 4.25E-04 | 1.01E-02 | 6.67E-03 | ACVRL1/JAK1/TERT/EGR1/EPHA1 | 5 | 8 |
| GO:0051607 | defense response to virus | 1.02E-01 | 1.32E-02 | 4.61E-04 | 1.07E-02 | 7.10E-03 | IRF5/IFIT5/TRAF3/STAT1/IL15 | 5 | 8 |
| GO:0046631 | alpha-beta T cell activation | 8.16E-02 | 7.68E-03 | 5.40E-04 | 1.24E-02 | 8.23E-03 | RHOA/IL15/PTGER4/GATA3 | 4 | 11 |
| GO:0090183 | regulation of kidney development | 6.12E-02 | 3.29E-03 | 5.60E-04 | 1.28E-02 | 8.45E-03 | EGR1/STAT1/GATA3 | 3 | 19 |
| GO:0035723 | interleukin-15-mediated signaling pathway | 4.08E-02 | 7.51E-04 | 6.00E-04 | 1.31E-02 | 8.66E-03 | JAK1/IL15 | 2 | 54 |
| GO:0043970 | histone H3-K9 acetylation | 4.08E-02 | 7.51E-04 | 6.00E-04 | 1.31E-02 | 8.66E-03 | CHEK1/GATA3 | 2 | 54 |
| GO:0071350 | cellular response to interleukin-15 | 4.08E-02 | 7.51E-04 | 6.00E-04 | 1.31E-02 | 8.66E-03 | JAK1/IL15 | 2 | 54 |
| GO:0072182 | regulation of nephron tubule epithelial cell differentiation | 4.08E-02 | 7.51E-04 | 6.00E-04 | 1.31E-02 | 8.66E-03 | STAT1/GATA3 | 2 | 54 |
| GO:0045580 | regulation of T cell differentiation | 8.16E-02 | 8.03E-03 | 6.38E-04 | 1.37E-02 | 9.09E-03 | RHOA/IL15/GATA3/ZMIZ1 | 4 | 10 |
| GO:0070672 | response to interleukin-15 | 4.08E-02 | 8.09E-04 | 6.99E-04 | 1.40E-02 | 9.27E-03 | JAK1/IL15 | 2 | 50 |
| GO:0090399 | replicative senescence | 4.08E-02 | 8.09E-04 | 6.99E-04 | 1.40E-02 | 9.27E-03 | CHEK1/TERT | 2 | 50 |
| GO:1901722 | regulation of cell proliferation involved in kidney development | 4.08E-02 | 8.09E-04 | 6.99E-04 | 1.40E-02 | 9.27E-03 | EGR1/GATA3 | 2 | 50 |
| GO:0051893 | regulation of focal adhesion assembly | 6.12E-02 | 3.52E-03 | 6.84E-04 | 1.40E-02 | 9.27E-03 | ACVRL1/RHOA/PTPRJ | 3 | 17 |
| GO:0090109 | regulation of cell-substrate junction assembly | 6.12E-02 | 3.52E-03 | 6.84E-04 | 1.40E-02 | 9.27E-03 | ACVRL1/RHOA/PTPRJ | 3 | 17 |
| GO:2000027 | regulation of organ morphogenesis | 1.02E-01 | 1.44E-02 | 6.74E-04 | 1.40E-02 | 9.27E-03 | RHOA/VDR/STAT1/TBX5/GATA3 | 5 | 7 |
| GO:0022409 | positive regulation of cell-cell adhesion | 1.02E-01 | 1.44E-02 | 6.86E-04 | 1.40E-02 | 9.27E-03 | RHOA/TRAF6/IL15/GATA3/ZMIZ1 | 5 | 7 |
| GO:0043297 | apical junction assembly | 6.12E-02 | 3.58E-03 | 7.17E-04 | 1.42E-02 | 9.38E-03 | RHOA/NPHP1/IKBKB | 3 | 17 |
| GO:0071772 | response to BMP | 8.16E-02 | 8.32E-03 | 7.28E-04 | 1.42E-02 | 9.38E-03 | ACVRL1/BMP6/EGR1/GATA3 | 4 | 10 |
| GO:0071773 | cellular response to BMP stimulus | 8.16E-02 | 8.32E-03 | 7.28E-04 | 1.42E-02 | 9.38E-03 | ACVRL1/BMP6/EGR1/GATA3 | 4 | 10 |
| GO:0002224 | toll-like receptor signaling pathway | 8.16E-02 | 8.38E-03 | 7.47E-04 | 1.43E-02 | 9.47E-03 | TRAF3/TRAF6/IKBKB/RIPK1 | 4 | 10 |
| GO:0051091 | positive regulation of DNA binding transcription factor activity | 1.02E-01 | 1.47E-02 | 7.50E-04 | 1.43E-02 | 9.47E-03 | HDAC4/TRAF6/IKBKB/NFKB2/RIPK1 | 5 | 7 |
| GO:0051251 | positive regulation of lymphocyte activation | 1.02E-01 | 1.48E-02 | 7.77E-04 | 1.47E-02 | 9.72E-03 | RHOA/TRAF6/IL15/GATA3/ZMIZ1 | 5 | 7 |
| GO:0010566 | regulation of ketone biosynthetic process | 4.08E-02 | 8.67E-04 | 8.05E-04 | 1.49E-02 | 9.89E-03 | BMP6/EGR1 | 2 | 47 |
| GO:0072160 | nephron tubule epithelial cell differentiation | 4.08E-02 | 8.67E-04 | 8.05E-04 | 1.49E-02 | 9.89E-03 | STAT1/GATA3 | 2 | 47 |
| GO:0030858 | positive regulation of epithelial cell differentiation | 6.12E-02 | 3.76E-03 | 8.23E-04 | 1.51E-02 | 9.97E-03 | ACVRL1/VDR/BMP6 | 3 | 16 |
| GO:0051092 | positive regulation of NF-kappaB transcription factor activity | 8.16E-02 | 8.61E-03 | 8.27E-04 | 1.51E-02 | 9.97E-03 | TRAF6/IKBKB/NFKB2/RIPK1 | 4 | 9 |
| GO:0021700 | developmental maturation | 1.02E-01 | 1.51E-02 | 8.47E-04 | 1.53E-02 | 1.01E-02 | ACVRL1/RHOA/KLF2/IL15/GATA3 | 5 | 7 |
| GO:1903391 | regulation of adherens junction organization | 6.12E-02 | 3.87E-03 | 8.99E-04 | 1.61E-02 | 1.07E-02 | ACVRL1/RHOA/PTPRJ | 3 | 16 |
| GO:1904996 | positive regulation of leukocyte adhesion to vascular endothelial cell | 4.08E-02 | 9.24E-04 | 9.19E-04 | 1.63E-02 | 1.08E-02 | RHOA/TRAF6 | 2 | 44 |
| GO:0071695 | anatomical structure maturation | 8.16E-02 | 8.96E-03 | 9.58E-04 | 1.67E-02 | 1.11E-02 | ACVRL1/RHOA/KLF2/GATA3 | 4 | 9 |
| GO:0051403 | stress-activated MAPK cascade | 1.02E-01 | 1.55E-02 | 9.53E-04 | 1.67E-02 | 1.11E-02 | LTBR/TRAF6/IKBKB/RIPK1/PTGER4 | 5 | 7 |
| GO:0090185 | negative regulation of kidney development | 4.08E-02 | 9.82E-04 | 1.04E-03 | 1.77E-02 | 1.17E-02 | STAT1/GATA3 | 2 | 42 |
| GO:1905331 | negative regulation of morphogenesis of an epithelium | 4.08E-02 | 9.82E-04 | 1.04E-03 | 1.77E-02 | 1.17E-02 | STAT1/GATA3 | 2 | 42 |
| GO:0050680 | negative regulation of epithelial cell proliferation | 8.16E-02 | 9.13E-03 | 1.03E-03 | 1.77E-02 | 1.17E-02 | ACVRL1/VDR/STAT1/GATA3 | 4 | 9 |
| GO:0034330 | cell junction organization | 1.02E-01 | 1.59E-02 | 1.05E-03 | 1.77E-02 | 1.17E-02 | ACVRL1/RHOA/PTPRJ/NPHP1/IKBKB | 5 | 6 |
| GO:0050688 | regulation of defense response to virus | 6.12E-02 | 4.16E-03 | 1.11E-03 | 1.84E-02 | 1.22E-02 | TRAF3/STAT1/IL15 | 3 | 15 |
| GO:0002697 | regulation of immune effector process | 1.22E-01 | 2.42E-02 | 1.11E-03 | 1.84E-02 | 1.22E-02 | TRAF3/TRAF6/PTPRJ/STAT1/IL15/GATA3 | 6 | 5 |
| GO:0043011 | myeloid dendritic cell differentiation | 4.08E-02 | 1.04E-03 | 1.17E-03 | 1.89E-02 | 1.25E-02 | LTBR/TRAF6 | 2 | 39 |
| GO:0070932 | histone H3 deacetylation | 4.08E-02 | 1.04E-03 | 1.17E-03 | 1.89E-02 | 1.25E-02 | HDAC4/ATXN3 | 2 | 39 |
| GO:0032481 | positive regulation of type I interferon production | 6.12E-02 | 4.28E-03 | 1.20E-03 | 1.89E-02 | 1.25E-02 | IRF5/NFKB2/STAT1 | 3 | 14 |
| GO:0045619 | regulation of lymphocyte differentiation | 8.16E-02 | 9.48E-03 | 1.18E-03 | 1.89E-02 | 1.25E-02 | RHOA/IL15/GATA3/ZMIZ1 | 4 | 9 |
| GO:0048771 | tissue remodeling | 8.16E-02 | 9.48E-03 | 1.18E-03 | 1.89E-02 | 1.25E-02 | ACVRL1/TRAF6/VDR/IL15 | 4 | 9 |
| GO:0051090 | regulation of DNA binding transcription factor activity | 1.22E-01 | 2.45E-02 | 1.20E-03 | 1.89E-02 | 1.25E-02 | HDAC4/TRAF3/TRAF6/IKBKB/NFKB2/RIPK1 | 6 | 5 |
| GO:0002726 | positive regulation of T cell cytokine production | 4.08E-02 | 1.10E-03 | 1.30E-03 | 1.96E-02 | 1.30E-02 | TRAF6/GATA3 | 2 | 37 |
| GO:0003159 | morphogenesis of an endothelium | 4.08E-02 | 1.10E-03 | 1.30E-03 | 1.96E-02 | 1.30E-02 | ACVRL1/RHOA | 2 | 37 |
| GO:0032332 | positive regulation of chondrocyte differentiation | 4.08E-02 | 1.10E-03 | 1.30E-03 | 1.96E-02 | 1.30E-02 | ACVRL1/BMP6 | 2 | 37 |
| GO:0061154 | endothelial tube morphogenesis | 4.08E-02 | 1.10E-03 | 1.30E-03 | 1.96E-02 | 1.30E-02 | ACVRL1/RHOA | 2 | 37 |
| GO:2000696 | regulation of epithelial cell differentiation involved in kidney development | 4.08E-02 | 1.10E-03 | 1.30E-03 | 1.96E-02 | 1.30E-02 | STAT1/GATA3 | 2 | 37 |
| GO:0002040 | sprouting angiogenesis | 8.16E-02 | 9.65E-03 | 1.26E-03 | 1.96E-02 | 1.30E-02 | ACVRL1/RHOA/JAK1/KLF2 | 4 | 8 |
| GO:0032727 | positive regulation of interferon-alpha production | 4.08E-02 | 1.16E-03 | 1.44E-03 | 2.09E-02 | 1.38E-02 | IRF5/STAT1 | 2 | 35 |
| GO:0060044 | negative regulation of cardiac muscle cell proliferation | 4.08E-02 | 1.16E-03 | 1.44E-03 | 2.09E-02 | 1.38E-02 | RIPK1/TBX5 | 2 | 35 |
| GO:0060231 | mesenchymal to epithelial transition | 4.08E-02 | 1.16E-03 | 1.44E-03 | 2.09E-02 | 1.38E-02 | STAT1/GATA3 | 2 | 35 |
| GO:0050886 | endocrine process | 6.12E-02 | 4.56E-03 | 1.45E-03 | 2.09E-02 | 1.38E-02 | RHOA/BMP6/GATA3 | 3 | 13 |
| GO:1903037 | regulation of leukocyte cell-cell adhesion | 1.02E-01 | 1.70E-02 | 1.41E-03 | 2.09E-02 | 1.38E-02 | RHOA/TRAF6/IL15/GATA3/ZMIZ1 | 5 | 6 |
| GO:0002758 | innate immune response-activating signal transduction | 1.02E-01 | 1.70E-02 | 1.44E-03 | 2.09E-02 | 1.38E-02 | TRAF3/TRAF6/IKBKB/ICAM3/RIPK1 | 5 | 6 |
| GO:0071222 | cellular response to lipopolysaccharide | 8.16E-02 | 1.01E-02 | 1.47E-03 | 2.10E-02 | 1.39E-02 | RHOA/TRAF6/BMP6/NFKBIB | 4 | 8 |
| GO:0050852 | T cell receptor signaling pathway | 8.16E-02 | 1.01E-02 | 1.50E-03 | 2.13E-02 | 1.41E-02 | TRAF6/PTPRJ/IKBKB/GATA3 | 4 | 8 |
| GO:0007045 | cell-substrate adherens junction assembly | 6.12E-02 | 4.68E-03 | 1.56E-03 | 2.18E-02 | 1.44E-02 | ACVRL1/RHOA/PTPRJ | 3 | 13 |
| GO:0048041 | focal adhesion assembly | 6.12E-02 | 4.68E-03 | 1.56E-03 | 2.18E-02 | 1.44E-02 | ACVRL1/RHOA/PTPRJ | 3 | 13 |
| GO:0033233 | regulation of protein sumoylation | 4.08E-02 | 1.21E-03 | 1.59E-03 | 2.20E-02 | 1.46E-02 | HDAC4/EGR1 | 2 | 34 |
| GO:0070920 | regulation of production of small RNA involved in gene silencing by RNA | 4.08E-02 | 1.21E-03 | 1.59E-03 | 2.20E-02 | 1.46E-02 | TERT/RIPK1 | 2 | 34 |
| GO:0002696 | positive regulation of leukocyte activation | 1.02E-01 | 1.76E-02 | 1.64E-03 | 2.25E-02 | 1.49E-02 | RHOA/TRAF6/IL15/GATA3/ZMIZ1 | 5 | 6 |
| GO:0045582 | positive regulation of T cell differentiation | 6.12E-02 | 4.80E-03 | 1.67E-03 | 2.26E-02 | 1.50E-02 | RHOA/GATA3/ZMIZ1 | 3 | 13 |
| GO:0051492 | regulation of stress fiber assembly | 6.12E-02 | 4.80E-03 | 1.67E-03 | 2.26E-02 | 1.50E-02 | RHOA/EPHA1/PTGER4 | 3 | 13 |
| GO:0071219 | cellular response to molecule of bacterial origin | 8.16E-02 | 1.05E-02 | 1.70E-03 | 2.29E-02 | 1.51E-02 | RHOA/TRAF6/BMP6/NFKBIB | 4 | 8 |
| GO:0050863 | regulation of T cell activation | 1.02E-01 | 1.77E-02 | 1.71E-03 | 2.29E-02 | 1.51E-02 | RHOA/TRAF6/IL15/GATA3/ZMIZ1 | 5 | 6 |
| GO:0048799 | animal organ maturation | 4.08E-02 | 1.27E-03 | 1.75E-03 | 2.29E-02 | 1.52E-02 | RHOA/GATA3 | 2 | 32 |
| GO:2000810 | regulation of bicellular tight junction assembly | 4.08E-02 | 1.27E-03 | 1.75E-03 | 2.29E-02 | 1.52E-02 | NPHP1/IKBKB | 2 | 32 |
| GO:0042446 | hormone biosynthetic process | 6.12E-02 | 4.85E-03 | 1.73E-03 | 2.29E-02 | 1.52E-02 | VDR/BMP6/EGR1 | 3 | 13 |
| GO:1901214 | regulation of neuron death | 1.02E-01 | 1.80E-02 | 1.81E-03 | 2.36E-02 | 1.56E-02 | HDAC4/RHOA/TERT/EGR1/GATA3 | 5 | 6 |
| GO:0060562 | epithelial tube morphogenesis | 1.02E-01 | 1.80E-02 | 1.84E-03 | 2.38E-02 | 1.57E-02 | ACVRL1/RHOA/TRAF6/VDR/GATA3 | 5 | 6 |
| GO:0045603 | positive regulation of endothelial cell differentiation | 4.08E-02 | 1.33E-03 | 1.91E-03 | 2.40E-02 | 1.59E-02 | ACVRL1/BMP6 | 2 | 31 |
| GO:0071305 | cellular response to vitamin D | 4.08E-02 | 1.33E-03 | 1.91E-03 | 2.40E-02 | 1.59E-02 | VDR/IL15 | 2 | 31 |
| GO:0072215 | regulation of metanephros development | 4.08E-02 | 1.33E-03 | 1.91E-03 | 2.40E-02 | 1.59E-02 | EGR1/STAT1 | 2 | 31 |
| GO:2000377 | regulation of reactive oxygen species metabolic process | 8.16E-02 | 1.07E-02 | 1.88E-03 | 2.40E-02 | 1.59E-02 | HDAC4/RHOA/KLF2/RIPK1 | 4 | 8 |
| GO:0002218 | activation of innate immune response | 1.02E-01 | 1.82E-02 | 1.91E-03 | 2.40E-02 | 1.59E-02 | TRAF3/TRAF6/IKBKB/ICAM3/RIPK1 | 5 | 6 |
| GO:0002286 | T cell activation involved in immune response | 6.12E-02 | 5.08E-03 | 1.98E-03 | 2.45E-02 | 1.62E-02 | ITGAL/PTGER4/GATA3 | 3 | 12 |
| GO:0050867 | positive regulation of cell activation | 1.02E-01 | 1.83E-02 | 1.97E-03 | 2.45E-02 | 1.62E-02 | RHOA/TRAF6/IL15/GATA3/ZMIZ1 | 5 | 6 |
| GO:0006694 | steroid biosynthetic process | 8.16E-02 | 1.10E-02 | 2.03E-03 | 2.49E-02 | 1.65E-02 | SF1/VDR/BMP6/EGR1 | 4 | 7 |
| GO:0007178 | transmembrane receptor protein serine/threonine kinase signaling pathway | 1.02E-01 | 1.85E-02 | 2.05E-03 | 2.51E-02 | 1.66E-02 | ACVRL1/RHOA/LTBP2/BMP6/EGR1 | 5 | 6 |
| GO:1901889 | negative regulation of cell junction assembly | 4.08E-02 | 1.39E-03 | 2.08E-03 | 2.53E-02 | 1.67E-02 | ACVRL1/IKBKB | 2 | 29 |
| GO:0034333 | adherens junction assembly | 6.12E-02 | 5.20E-03 | 2.11E-03 | 2.53E-02 | 1.67E-02 | ACVRL1/RHOA/PTPRJ | 3 | 12 |
| GO:0045639 | positive regulation of myeloid cell differentiation | 6.12E-02 | 5.20E-03 | 2.11E-03 | 2.53E-02 | 1.67E-02 | TRAF6/STAT1/RIPK1 | 3 | 12 |
| GO:0001656 | metanephros development | 6.12E-02 | 5.26E-03 | 2.17E-03 | 2.56E-02 | 1.70E-02 | EGR1/STAT1/GATA3 | 3 | 12 |
| GO:0060333 | interferon-gamma-mediated signaling pathway | 6.12E-02 | 5.26E-03 | 2.17E-03 | 2.56E-02 | 1.70E-02 | IRF5/JAK1/STAT1 | 3 | 12 |
| GO:0002221 | pattern recognition receptor signaling pathway | 8.16E-02 | 1.12E-02 | 2.19E-03 | 2.56E-02 | 1.70E-02 | TRAF3/TRAF6/IKBKB/RIPK1 | 4 | 7 |
| GO:0001501 | skeletal system development | 1.22E-01 | 2.76E-02 | 2.18E-03 | 2.56E-02 | 1.70E-02 | HDAC4/ACVRL1/RHOA/VDR/BMP6/PTGER4 | 6 | 4 |
| GO:0031664 | regulation of lipopolysaccharide-mediated signaling pathway | 4.08E-02 | 1.44E-03 | 2.26E-03 | 2.57E-02 | 1.70E-02 | TRAF6/BMP6 | 2 | 28 |
| GO:0060330 | regulation of response to interferon-gamma | 4.08E-02 | 1.44E-03 | 2.26E-03 | 2.57E-02 | 1.70E-02 | JAK1/STAT1 | 2 | 28 |
| GO:0060334 | regulation of interferon-gamma-mediated signaling pathway | 4.08E-02 | 1.44E-03 | 2.26E-03 | 2.57E-02 | 1.70E-02 | JAK1/STAT1 | 2 | 28 |
| GO:0046890 | regulation of lipid biosynthetic process | 8.16E-02 | 1.13E-02 | 2.23E-03 | 2.57E-02 | 1.70E-02 | SF1/VDR/BMP6/EGR1 | 4 | 7 |
| GO:0001101 | response to acid chemical | 1.02E-01 | 1.88E-02 | 2.22E-03 | 2.57E-02 | 1.70E-02 | GSS/RHOA/BMP6/EGR1/KLF2 | 5 | 5 |
| GO:1901216 | positive regulation of neuron death | 6.12E-02 | 5.37E-03 | 2.31E-03 | 2.61E-02 | 1.72E-02 | HDAC4/RHOA/EGR1 | 3 | 11 |
| GO:0045766 | positive regulation of angiogenesis | 8.16E-02 | 1.14E-02 | 2.31E-03 | 2.61E-02 | 1.72E-02 | ACVRL1/JAK1/TERT/EPHA1 | 4 | 7 |
| GO:0007254 | JNK cascade | 8.16E-02 | 1.14E-02 | 2.35E-03 | 2.64E-02 | 1.74E-02 | LTBR/TRAF6/RIPK1/PTGER4 | 4 | 7 |
| GO:0032231 | regulation of actin filament bundle assembly | 6.12E-02 | 5.43E-03 | 2.39E-03 | 2.64E-02 | 1.75E-02 | RHOA/EPHA1/PTGER4 | 3 | 11 |
| GO:0110020 | regulation of actomyosin structure organization | 6.12E-02 | 5.43E-03 | 2.39E-03 | 2.64E-02 | 1.75E-02 | RHOA/EPHA1/PTGER4 | 3 | 11 |
| GO:0006700 | C21-steroid hormone biosynthetic process | 4.08E-02 | 1.50E-03 | 2.44E-03 | 2.65E-02 | 1.75E-02 | BMP6/EGR1 | 2 | 27 |
| GO:0032647 | regulation of interferon-alpha production | 4.08E-02 | 1.50E-03 | 2.44E-03 | 2.65E-02 | 1.75E-02 | IRF5/STAT1 | 2 | 27 |
| GO:1903579 | negative regulation of ATP metabolic process | 4.08E-02 | 1.50E-03 | 2.44E-03 | 2.65E-02 | 1.75E-02 | HDAC4/RHOA | 2 | 27 |
| GO:2000679 | positive regulation of transcription regulatory region DNA binding | 4.08E-02 | 1.50E-03 | 2.44E-03 | 2.65E-02 | 1.75E-02 | TRAF6/GATA3 | 2 | 27 |
| GO:0007044 | cell-substrate junction assembly | 6.12E-02 | 5.55E-03 | 2.53E-03 | 2.71E-02 | 1.79E-02 | ACVRL1/RHOA/PTPRJ | 3 | 11 |
| GO:1903426 | regulation of reactive oxygen species biosynthetic process | 6.12E-02 | 5.55E-03 | 2.53E-03 | 2.71E-02 | 1.79E-02 | HDAC4/RHOA/KLF2 | 3 | 11 |
| GO:0009636 | response to toxic substance | 1.22E-01 | 2.84E-02 | 2.54E-03 | 2.71E-02 | 1.79E-02 | RHOA/EGR1/STAT1/KLF2/RIPK1/GATA3 | 6 | 4 |
| GO:0007043 | cell-cell junction assembly | 6.12E-02 | 5.60E-03 | 2.61E-03 | 2.74E-02 | 1.81E-02 | RHOA/NPHP1/IKBKB | 3 | 11 |
| GO:0030038 | contractile actin filament bundle assembly | 6.12E-02 | 5.60E-03 | 2.61E-03 | 2.74E-02 | 1.81E-02 | RHOA/EPHA1/PTGER4 | 3 | 11 |
| GO:0043149 | stress fiber assembly | 6.12E-02 | 5.60E-03 | 2.61E-03 | 2.74E-02 | 1.81E-02 | RHOA/EPHA1/PTGER4 | 3 | 11 |
| GO:0071216 | cellular response to biotic stimulus | 8.16E-02 | 1.18E-02 | 2.62E-03 | 2.74E-02 | 1.81E-02 | RHOA/TRAF6/BMP6/NFKBIB | 4 | 7 |
| GO:2000379 | positive regulation of reactive oxygen species metabolic process | 6.12E-02 | 5.66E-03 | 2.69E-03 | 2.79E-02 | 1.85E-02 | HDAC4/KLF2/RIPK1 | 3 | 11 |
| GO:0010817 | regulation of hormone levels | 1.22E-01 | 2.88E-02 | 2.72E-03 | 2.82E-02 | 1.86E-02 | VDR/IL11/BMP6/EGR1/RBP1/GATA3 | 6 | 4 |
| GO:0001773 | myeloid dendritic cell activation | 4.08E-02 | 1.62E-03 | 2.83E-03 | 2.82E-02 | 1.86E-02 | LTBR/TRAF6 | 2 | 25 |
| GO:0032607 | interferon-alpha production | 4.08E-02 | 1.62E-03 | 2.83E-03 | 2.82E-02 | 1.86E-02 | IRF5/STAT1 | 2 | 25 |
| GO:1900543 | negative regulation of purine nucleotide metabolic process | 4.08E-02 | 1.62E-03 | 2.83E-03 | 2.82E-02 | 1.86E-02 | HDAC4/RHOA | 2 | 25 |
| GO:1904994 | regulation of leukocyte adhesion to vascular endothelial cell | 4.08E-02 | 1.62E-03 | 2.83E-03 | 2.82E-02 | 1.86E-02 | RHOA/TRAF6 | 2 | 25 |
| GO:0046632 | alpha-beta T cell differentiation | 6.12E-02 | 5.72E-03 | 2.76E-03 | 2.82E-02 | 1.86E-02 | RHOA/PTGER4/GATA3 | 3 | 11 |
| GO:0018108 | peptidyl-tyrosine phosphorylation | 1.02E-01 | 1.99E-02 | 2.81E-03 | 2.82E-02 | 1.86E-02 | JAK1/IL11/PTPRJ/EPHA1/IL15 | 5 | 5 |
| GO:0070997 | neuron death | 1.02E-01 | 1.99E-02 | 2.84E-03 | 2.82E-02 | 1.86E-02 | HDAC4/RHOA/TERT/EGR1/GATA3 | 5 | 5 |
| GO:0044089 | positive regulation of cellular component biogenesis | 1.22E-01 | 2.89E-02 | 2.75E-03 | 2.82E-02 | 1.86E-02 | HDAC4/RHOA/PTPRJ/NPHP1/CLIP1/EPHA1 | 6 | 4 |
| GO:0043523 | regulation of neuron apoptotic process | 8.16E-02 | 1.21E-02 | 2.86E-03 | 2.83E-02 | 1.87E-02 | HDAC4/RHOA/TERT/GATA3 | 4 | 7 |
| GO:0018212 | peptidyl-tyrosine modification | 1.02E-01 | 2.00E-02 | 2.91E-03 | 2.86E-02 | 1.89E-02 | JAK1/IL11/PTPRJ/EPHA1/IL15 | 5 | 5 |
| GO:0045980 | negative regulation of nucleotide metabolic process | 4.08E-02 | 1.68E-03 | 3.04E-03 | 2.95E-02 | 1.95E-02 | HDAC4/RHOA | 2 | 24 |
| GO:0070102 | interleukin-6-mediated signaling pathway | 4.08E-02 | 1.68E-03 | 3.04E-03 | 2.95E-02 | 1.95E-02 | JAK1/STAT1 | 2 | 24 |
| GO:0010862 | positive regulation of pathway-restricted SMAD protein phosphorylation | 4.08E-02 | 1.73E-03 | 3.25E-03 | 3.13E-02 | 2.07E-02 | ACVRL1/BMP6 | 2 | 24 |
| GO:0061036 | positive regulation of cartilage development | 4.08E-02 | 1.73E-03 | 3.25E-03 | 3.13E-02 | 2.07E-02 | ACVRL1/BMP6 | 2 | 24 |
| GO:0045446 | endothelial cell differentiation | 6.12E-02 | 6.07E-03 | 3.26E-03 | 3.13E-02 | 2.07E-02 | ACVRL1/BMP6/IKBKB | 3 | 10 |
| GO:0050851 | antigen receptor-mediated signaling pathway | 8.16E-02 | 1.25E-02 | 3.28E-03 | 3.13E-02 | 2.07E-02 | TRAF6/PTPRJ/IKBKB/GATA3 | 4 | 7 |
| GO:0002724 | regulation of T cell cytokine production | 4.08E-02 | 1.79E-03 | 3.46E-03 | 3.22E-02 | 2.13E-02 | TRAF6/GATA3 | 2 | 23 |
| GO:0044319 | wound healing, spreading of cells | 4.08E-02 | 1.79E-03 | 3.46E-03 | 3.22E-02 | 2.13E-02 | ACVRL1/RHOA | 2 | 23 |
| GO:0055022 | negative regulation of cardiac muscle tissue growth | 4.08E-02 | 1.79E-03 | 3.46E-03 | 3.22E-02 | 2.13E-02 | RIPK1/TBX5 | 2 | 23 |
| GO:0061117 | negative regulation of heart growth | 4.08E-02 | 1.79E-03 | 3.46E-03 | 3.22E-02 | 2.13E-02 | RIPK1/TBX5 | 2 | 23 |
| GO:0090505 | epiboly involved in wound healing | 4.08E-02 | 1.79E-03 | 3.46E-03 | 3.22E-02 | 2.13E-02 | ACVRL1/RHOA | 2 | 23 |
| GO:0050678 | regulation of epithelial cell proliferation | 1.02E-01 | 2.09E-02 | 3.45E-03 | 3.22E-02 | 2.13E-02 | ACVRL1/VDR/BMP6/STAT1/GATA3 | 5 | 5 |
| GO:0043618 | regulation of transcription from RNA polymerase II promoter in response to stress | 6.12E-02 | 6.24E-03 | 3.53E-03 | 3.27E-02 | 2.16E-02 | CHEK1/EGR1/KLF2 | 3 | 10 |
| GO:0071295 | cellular response to vitamin | 4.08E-02 | 1.85E-03 | 3.69E-03 | 3.33E-02 | 2.20E-02 | VDR/IL15 | 2 | 22 |
| GO:0090504 | epiboly | 4.08E-02 | 1.85E-03 | 3.69E-03 | 3.33E-02 | 2.20E-02 | ACVRL1/RHOA | 2 | 22 |
| GO:1901099 | negative regulation of signal transduction in absence of ligand | 4.08E-02 | 1.85E-03 | 3.69E-03 | 3.33E-02 | 2.20E-02 | TERT/RIPK1 | 2 | 22 |
| GO:2001240 | negative regulation of extrinsic apoptotic signaling pathway in absence of ligand | 4.08E-02 | 1.85E-03 | 3.69E-03 | 3.33E-02 | 2.20E-02 | TERT/RIPK1 | 2 | 22 |
| GO:0042035 | regulation of cytokine biosynthetic process | 6.12E-02 | 6.30E-03 | 3.63E-03 | 3.33E-02 | 2.20E-02 | TRAF6/EGR1/GATA3 | 3 | 10 |
| GO:0014706 | striated muscle tissue development | 1.02E-01 | 2.12E-02 | 3.70E-03 | 3.33E-02 | 2.20E-02 | HDAC4/RHOA/EGR1/RIPK1/TBX5 | 5 | 5 |
| GO:0045089 | positive regulation of innate immune response | 1.02E-01 | 2.13E-02 | 3.79E-03 | 3.39E-02 | 2.24E-02 | TRAF3/TRAF6/IKBKB/ICAM3/RIPK1 | 5 | 5 |
| GO:1901890 | positive regulation of cell junction assembly | 4.08E-02 | 1.91E-03 | 3.92E-03 | 3.45E-02 | 2.28E-02 | PTPRJ/NPHP1 | 2 | 21 |
| GO:2000352 | negative regulation of endothelial cell apoptotic process | 4.08E-02 | 1.91E-03 | 3.92E-03 | 3.45E-02 | 2.28E-02 | TERT/GATA3 | 2 | 21 |
| GO:0030218 | erythrocyte differentiation | 6.12E-02 | 6.47E-03 | 3.91E-03 | 3.45E-02 | 2.28E-02 | STAT1/KLF2/GATA3 | 3 | 9 |
| GO:0002429 | immune response-activating cell surface receptor signaling pathway | 1.02E-01 | 2.14E-02 | 3.87E-03 | 3.45E-02 | 2.28E-02 | TRAF6/PTPRJ/IKBKB/ICAM3/GATA3 | 5 | 5 |
| GO:0002223 | stimulatory C-type lectin receptor signaling pathway | 6.12E-02 | 6.53E-03 | 4.01E-03 | 3.47E-02 | 2.29E-02 | TRAF6/IKBKB/ICAM3 | 3 | 9 |
| GO:0007569 | cell aging | 6.12E-02 | 6.53E-03 | 4.01E-03 | 3.47E-02 | 2.29E-02 | CHEK1/TERT/ZMIZ1 | 3 | 9 |
| GO:0030183 | B cell differentiation | 6.12E-02 | 6.53E-03 | 4.01E-03 | 3.47E-02 | 2.29E-02 | HDAC4/IL11/PTPRJ | 3 | 9 |
| GO:0043620 | regulation of DNA-templated transcription in response to stress | 6.12E-02 | 6.53E-03 | 4.01E-03 | 3.47E-02 | 2.29E-02 | CHEK1/EGR1/KLF2 | 3 | 9 |
| GO:0032735 | positive regulation of interleukin-12 production | 4.08E-02 | 1.96E-03 | 4.16E-03 | 3.58E-02 | 2.37E-02 | IRF5/TRAF6 | 2 | 21 |
| GO:0002220 | innate immune response activating cell surface receptor signaling pathway | 6.12E-02 | 6.70E-03 | 4.32E-03 | 3.70E-02 | 2.45E-02 | TRAF6/IKBKB/ICAM3 | 3 | 9 |
| GO:0110111 | NA | 4.08E-02 | 2.02E-03 | 4.40E-03 | 3.73E-02 | 2.47E-02 | STAT1/GATA3 | 2 | 20 |
| GO:0034754 | cellular hormone metabolic process | 6.12E-02 | 6.76E-03 | 4.42E-03 | 3.73E-02 | 2.47E-02 | BMP6/EGR1/RBP1 | 3 | 9 |
| GO:1903409 | reactive oxygen species biosynthetic process | 6.12E-02 | 6.76E-03 | 4.42E-03 | 3.73E-02 | 2.47E-02 | HDAC4/RHOA/KLF2 | 3 | 9 |
| GO:0060537 | muscle tissue development | 1.02E-01 | 2.21E-02 | 4.38E-03 | 3.73E-02 | 2.47E-02 | HDAC4/RHOA/EGR1/RIPK1/TBX5 | 5 | 5 |
| GO:0051402 | neuron apoptotic process | 8.16E-02 | 1.37E-02 | 4.48E-03 | 3.76E-02 | 2.49E-02 | HDAC4/RHOA/TERT/GATA3 | 4 | 6 |
| GO:0001503 | ossification | 1.02E-01 | 2.23E-02 | 4.58E-03 | 3.83E-02 | 2.53E-02 | HDAC4/RHOA/TRAF6/BMP6/PTGER4 | 5 | 5 |
| GO:0042089 | cytokine biosynthetic process | 6.12E-02 | 6.88E-03 | 4.64E-03 | 3.86E-02 | 2.55E-02 | TRAF6/EGR1/GATA3 | 3 | 9 |
| GO:0022407 | regulation of cell-cell adhesion | 1.02E-01 | 2.24E-02 | 4.68E-03 | 3.88E-02 | 2.57E-02 | RHOA/TRAF6/IL15/GATA3/ZMIZ1 | 5 | 5 |
| GO:0034101 | erythrocyte homeostasis | 6.12E-02 | 6.93E-03 | 4.75E-03 | 3.90E-02 | 2.58E-02 | STAT1/KLF2/GATA3 | 3 | 9 |
| GO:0042107 | cytokine metabolic process | 6.12E-02 | 6.93E-03 | 4.75E-03 | 3.90E-02 | 2.58E-02 | TRAF6/EGR1/GATA3 | 3 | 9 |
| GO:0045926 | negative regulation of growth | 8.16E-02 | 1.40E-02 | 4.83E-03 | 3.95E-02 | 2.61E-02 | ACVRL1/PTPRJ/RIPK1/TBX5 | 4 | 6 |
| GO:0008207 | C21-steroid hormone metabolic process | 4.08E-02 | 2.14E-03 | 4.91E-03 | 3.95E-02 | 2.61E-02 | BMP6/EGR1 | 2 | 19 |
| GO:0034105 | positive regulation of tissue remodeling | 4.08E-02 | 2.14E-03 | 4.91E-03 | 3.95E-02 | 2.61E-02 | VDR/IL15 | 2 | 19 |
| GO:0042181 | ketone biosynthetic process | 4.08E-02 | 2.14E-03 | 4.91E-03 | 3.95E-02 | 2.61E-02 | BMP6/EGR1 | 2 | 19 |
| GO:0050691 | regulation of defense response to virus by host | 4.08E-02 | 2.14E-03 | 4.91E-03 | 3.95E-02 | 2.61E-02 | STAT1/IL15 | 2 | 19 |
| GO:0046330 | positive regulation of JNK cascade | 6.12E-02 | 7.05E-03 | 4.97E-03 | 3.99E-02 | 2.64E-02 | LTBR/TRAF6/RIPK1 | 3 | 9 |
| GO:0007517 | muscle organ development | 1.02E-01 | 2.28E-02 | 4.99E-03 | 3.99E-02 | 2.64E-02 | HDAC4/RHOA/EGR1/RIPK1/TBX5 | 5 | 4 |
| GO:0003007 | heart morphogenesis | 8.16E-02 | 1.42E-02 | 5.11E-03 | 4.07E-02 | 2.69E-02 | ACVRL1/TBX5/GATA3/ZMIZ1 | 4 | 6 |
| GO:0002711 | positive regulation of T cell mediated immunity | 4.08E-02 | 2.20E-03 | 5.17E-03 | 4.08E-02 | 2.70E-02 | TRAF6/GATA3 | 2 | 19 |
| GO:0033280 | response to vitamin D | 4.08E-02 | 2.20E-03 | 5.17E-03 | 4.08E-02 | 2.70E-02 | VDR/IL15 | 2 | 19 |
| GO:0051249 | regulation of lymphocyte activation | 1.02E-01 | 2.31E-02 | 5.32E-03 | 4.18E-02 | 2.77E-02 | RHOA/TRAF6/IL15/GATA3/ZMIZ1 | 5 | 4 |
| GO:0046621 | negative regulation of organ growth | 4.08E-02 | 2.25E-03 | 5.44E-03 | 4.25E-02 | 2.81E-02 | RIPK1/TBX5 | 2 | 18 |
| GO:0045216 | cell-cell junction organization | 6.12E-02 | 7.28E-03 | 5.44E-03 | 4.25E-02 | 2.81E-02 | RHOA/NPHP1/IKBKB | 3 | 8 |
| GO:0002768 | immune response-regulating cell surface receptor signaling pathway | 1.02E-01 | 2.34E-02 | 5.60E-03 | 4.36E-02 | 2.88E-02 | TRAF6/PTPRJ/IKBKB/ICAM3/GATA3 | 5 | 4 |
| GO:0002369 | T cell cytokine production | 4.08E-02 | 2.31E-03 | 5.72E-03 | 4.41E-02 | 2.92E-02 | TRAF6/GATA3 | 2 | 18 |
| GO:1902893 | regulation of pri-miRNA transcription from RNA polymerase II promoter | 4.08E-02 | 2.31E-03 | 5.72E-03 | 4.41E-02 | 2.92E-02 | HDAC4/TERT | 2 | 18 |
| GO:1903670 | regulation of sprouting angiogenesis | 6.12E-02 | 7.45E-03 | 5.80E-03 | 4.46E-02 | 2.95E-02 | RHOA/JAK1/KLF2 | 3 | 8 |
| GO:0072210 | metanephric nephron development | 4.08E-02 | 2.37E-03 | 6.00E-03 | 4.57E-02 | 3.03E-02 | EGR1/STAT1 | 2 | 17 |
| GO:0090184 | positive regulation of kidney development | 4.08E-02 | 2.37E-03 | 6.00E-03 | 4.57E-02 | 3.03E-02 | EGR1/GATA3 | 2 | 17 |
| GO:0030509 | BMP signaling pathway | 6.12E-02 | 7.57E-03 | 6.06E-03 | 4.60E-02 | 3.04E-02 | ACVRL1/BMP6/EGR1 | 3 | 8 |
| GO:0055026 | negative regulation of cardiac muscle tissue development | 4.08E-02 | 2.43E-03 | 6.29E-03 | 4.74E-02 | 3.14E-02 | RIPK1/TBX5 | 2 | 17 |
| GO:0072593 | reactive oxygen species metabolic process | 8.16E-02 | 1.51E-02 | 6.29E-03 | 4.74E-02 | 3.14E-02 | HDAC4/RHOA/KLF2/RIPK1 | 4 | 5 |
| GO:0050673 | epithelial cell proliferation | 1.02E-01 | 2.41E-02 | 6.33E-03 | 4.75E-02 | 3.14E-02 | ACVRL1/VDR/BMP6/STAT1/GATA3 | 5 | 4 |
| GO:0034332 | adherens junction organization | 6.12E-02 | 7.74E-03 | 6.45E-03 | 4.82E-02 | 3.19E-02 | ACVRL1/RHOA/PTPRJ | 3 | 8 |
| GO:0006775 | fat-soluble vitamin metabolic process | 4.08E-02 | 2.48E-03 | 6.58E-03 | 4.87E-02 | 3.22E-02 | VDR/RBP1 | 2 | 16 |
| GO:0097028 | dendritic cell differentiation | 4.08E-02 | 2.48E-03 | 6.58E-03 | 4.87E-02 | 3.22E-02 | LTBR/TRAF6 | 2 | 16 |
| GO:2001239 | regulation of extrinsic apoptotic signaling pathway in absence of ligand | 4.08E-02 | 2.48E-03 | 6.58E-03 | 4.87E-02 | 3.22E-02 | TERT/RIPK1 | 2 | 16 |
| GO:0051017 | actin filament bundle assembly | 6.12E-02 | 7.86E-03 | 6.72E-03 | 4.95E-02 | 3.27E-02 | RHOA/EPHA1/PTGER4 | 3 | 8 |
| GO:0030225 | macrophage differentiation | 4.08E-02 | 2.54E-03 | 6.89E-03 | 4.98E-02 | 3.30E-02 | IL15/RIPK1 | 2 | 16 |
| GO:0035850 | epithelial cell differentiation involved in kidney development | 4.08E-02 | 2.54E-03 | 6.89E-03 | 4.98E-02 | 3.30E-02 | STAT1/GATA3 | 2 | 16 |
| GO:0060393 | regulation of pathway-restricted SMAD protein phosphorylation | 4.08E-02 | 2.54E-03 | 6.89E-03 | 4.98E-02 | 3.30E-02 | ACVRL1/BMP6 | 2 | 16 |
| GO:0061756 | leukocyte adhesion to vascular endothelial cell | 4.08E-02 | 2.54E-03 | 6.89E-03 | 4.98E-02 | 3.30E-02 | RHOA/TRAF6 | 2 | 16 |
| GO:0061572 | actin filament bundle organization | 6.12E-02 | 7.92E-03 | 6.85E-03 | 4.98E-02 | 3.30E-02 | RHOA/EPHA1/PTGER4 | 3 | 8 |

FDR, false detection rate; GO, gene ontology.

^a^ The Gene Ontology:Biological Process enrichment was carried out using the R package clusterProfiler(11) v3.10.1 function enrichGO with the full list of analyzed genes as the background set.

**Supplementary Table 5. Links of the 51 genes with GvHD in previous studies.**

| **Gene^a^** | **GvHD associations (reference)** | **Possible mechanism** | **GvHD** | **Species** |
| --- | --- | --- | --- | --- |
| ACVRL1, activin A receptor like type 1 | NA |  |  |  |
| ADK, adenosine kinase | NA |  |  |  |
| ATXN3, ataxin 3 | NA |  |  |  |
| BCAM, basal cell adhesion molecule | NA |  |  |  |
| BMP6, bone morphogenetic protein 6 | NA |  |  |  |
| CCL18, C-C motif chemokine ligand 18 | (12) |  | GvHD-associated dry eye | Human |
| CD1E, CD1e molecule | NA |  |  |  |
| CHEK1, checkpoint kinase 1 | NA |  |  |  |
| CLIP1, CAP-Gly domain containing linker protein 1 | NA |  |  |  |
| CXCL5, C-X-C motif chemokine ligand 5 | (13) | Suppression of Th 1 and Th 17 responses | GvHD | Human |
| EGR1, early growth response 1 | NA |  |  |  |
| EPHA1, EPH receptor A1 | NA |  |  |  |
| ERG, ETS transcription factor ERG | NA |  |  |  |
| GATA3, GATA binding protein 3 | (14) | TGF-β generation and in vivo expansion of Tregs | GvHD | Murine |
| GSS, glutathione synthetase | NA |  |  |  |
| HDAC4, histone deacetylase 4 | (15,16) | TNF-α, IL-1, and IFN-γ inhibition | aGvHD | Human + murine |
| ICAM3, intercellular adhesion molecule 3 | NA |  |  |  |
| IDH3A, isocitrate dehydrogenase (NAD(+)) 3 alpha | NA |  |  |  |
| IFIT5, interferon induced protein with tetratricopeptide repeats 5 | NA |  |  |  |
| IKBKB, inhibitor of nuclear factor kappa B kinase subunit beta | (17) | NF kappa B signaling/regulation | GvHD | Murine |
| IL11, interleukin 11 | (18) | T cell polarization | aGvHD | Murine |
|  | (19) | Inhibition of CD4-mediated GvHD | GvHD | Murine |
| IL15, interleukin 15 | (20) | T-cell function | aGvHD | Murine |
| IL1R2, interleukin 1 receptor type 2 | (21) |  | cGvHD | Human |
| IRF5, interferon induced protein with tetratricopeptide repeats 5 | (22) | miR-146a regulation | aGvHD | Human |
| ITGAL, integrin subunit alpha L | NA |  |  |  |
| JAK1, Janus kinase 1 | (23) | Inhibition of JAK 1/2 signaling | aGvHD | Murine |
|  | (24) | Inhibition of JAK 1/2 signaling | GvHD | Murine |
|  | (25) | Inhibition of JAK 1/2 signaling | aGvHD | Murine |
|  | (26) | Inhibition of JAK 1/2 signaling | Steroid-refractory aGvHD and cGvHD | Human |
|  |  |  |  |  |
| KLF2, Kruppel like factor 2 | (27) | Treg migration | aGvHD | Murine |
| LTBP2, latent transforming growth factor beta binding protein 2 | NA |  |  |  |
| LTBR, lymphotoxin beta receptor | (28) | Blockade of germinal center formation | cGvHD | Murine |
|  | (29) | Blockade of the LIGHT-HVEM pathway | GvHD | Murine |
|  | (30) | Inhibition of LIGHT | GvHD | Murine |
|  | (31) | T cell inhibition | GvHD | Murine |
| MRPS14, mitochondrial ribosomal protein S14 | NA |  |  |  |
| NAPB, NSF attachment protein beta | NA |  |  |  |
| NDUFA11, NADH:ubiquinone oxidoreductase subunit A11 | NA |  |  |  |
| NFKB2, nuclear factor kappa B subunit 2 | (17) | NF kappa B signaling/regulation | GvHD | Murine |
| NFKBIB, NFKB inhibitor beta | (17) | NF kappa B signaling/regulation | GvHD | Murine |
| NPHP1, nephrocystin 1 | NA |  |  |  |
| PRR3, proline rich 3 | NA |  |  |  |
| PTGER4, prostaglandin E receptor 4 | NA |  |  |  |
| PTPRJ, protein tyrosine phosphatase receptor type J | NA |  |  |  |
| RBP1, retinol binding protein 1 | (32) | HSP47 conjugation | cGvHD | Murine |
| RHOA, ras homolog family member A | (33) | Rho kinase inhibition | Intestinal GvHD | Murine |
| RIPK1, receptor interacting serine/threonine kinase 1 | (34,35) | TNFR2 activation | aGvHD | Murine |
| SF1, splicing factor 1 | NA |  |  |  |
| STAT1, signal transducer and activator of transcription 1 | (22) | miR-146a regulation | aGvHD | Human |
|  | (36) | Activation of inflammatory signaling pathways | GvHD | Murine |
| TBX5, T-box transcription factor 5 | NA |  |  |  |
| TERT, telomerase reverse transcriptase | (37) | Loss-of-function mutation | aGvHD | Human |
| TRAF3, TNF receptor associated factor 3 | (38,39) | TLR signalling | aGvHD | Human + murine |
| TRAF6, TNF receptor associated factor 6 | (40) | tTreg function | GvHD | Murine + human |
|  | (41) | TNF transcription | aGvHD | Murine + human |
|  | (38,39) | TLR signalling | aGvHD | Human + murine |
| VDR, vitamin D receptor | (42) | Immunomodulatory effects of vitamin D | cGvHD | Human |
|  | (43) | Actions of vitamin D | aGvHD | Human |
| ZDHHC13, zinc finger DHHC-type containing 13 | NA |  |  |  |
| ZMIZ1, zinc finger MIZ-type containing 1 | NA |  |  |  |
| ZNF527, zinc finger protein 527 | NA |  |  |  |


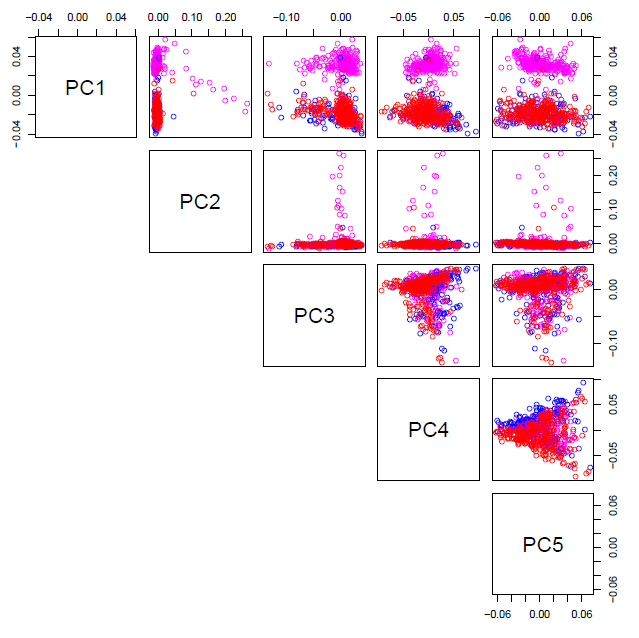


**Supplementary Figure 1. Scatterplot matrix of five principal components of the study cohorts.** The principal component analysis was executed as depicted in the Methods section of the main article. Red circles, Finnish Cohort 1; blue circles, Finnish Cohort 2; magenta circles, Spanish Cohort 1. PC, principal component.


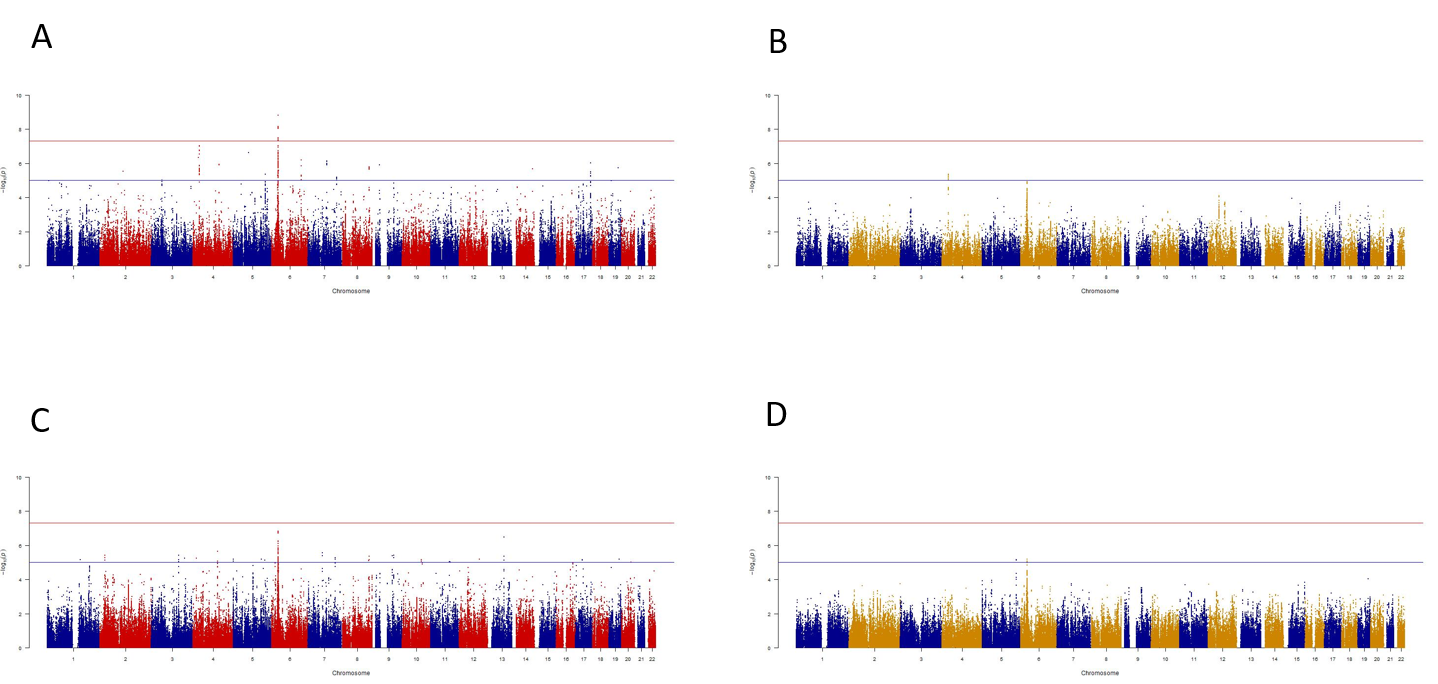


**Supplementary Figure 2. Manhattan plots of severe aGvHD in Finnish Cohort 1 before and after adjusting the results.** GWAS was performed as described in the Methods section of the main article. Age, gender, and stem cell source were used as covariates in the adjusted models. A) Recipient genotype unadjusted results for aGvDH grades III–IV vs 0. B) Recipient genotype adjusted results for aGvDH grades III–IV vs 0. C) Donor genotype unadjusted results for aGvDH grades III–IV vs 0. D) Donor genotype adjusted results for aGvDH grades III–IV vs 0.

**REFERENCES**

1. Baron C, Somogyi R, Greller LD, Rineau V, Wilkinson P, Cho CR, et al. Prediction of Graft-versus-host disease in humans by donor gene-expression profiling. PLoS Med. 2007;4(1):0069–83.

2. Glauzy S, Peffault de Latour R, André-Schmutz I, Lachuer J, Servais S, Socié G, et al. Alterations of circulating lymphoid committed progenitor cellular metabolism after allogeneic stem cell transplantation in humans. Exp Hematol. 2016;44(9):811–816.e3.

3. Lupsa N, Érsek B, Horváth A, Bencsik A, Lajkó E, Silló P, et al. Skin-homing CD8+ T cells preferentially express GPI-anchored peptidase inhibitor 16, an inhibitor of cathepsin K. Eur J Immunol. 2018;48(12):1944–57.

4. Furlan SN, Watkins B, Tkachev V, Flynn R, Cooley S, Ramakrishnan S, et al. Transcriptome analysis of GVHD reveals aurora kinase a as a targetable pathway for disease prevention. Sci Transl Med. 2015;7(315).

5. Takahashi N, Sato N, Takahashi S, Tojo A. Gene-expression profiles of peripheral blood mononuclear cell subpopulations in acute graft-vs-host disease following cord blood transplantation. Exp Hematol [Internet]. 2008;36(12):1760–74. Available from: http://dx.doi.org/10.1016/j.exphem.2008.07.007

6. Buzzeo MP, Yang J, Casella G, Reddy V. A preliminary gene expression profile of acute graft-versus-host disease. Cell Transplant. 2008;17(5):489–94.

7. Miller AM, Schneider R, Vance EA, Agura E, Powell S, Pascual V, et al. Differential Gene Expression Analysis Predicts For Response To Bortezomib In The Treatment Of Steroid Refractory Chronic Graft-Versus-Host Disease (cGVHD). Miller AM, editor. Blood [Internet]. 2013 Nov 15;122(21):122:4587. Available from: http://www.bloodjournal.org/content/122/21/4587

8. Hakim FT, Memon S, Jin P, Imanguli MM, Wang H, Rehman N, et al. Upregulation of IFN-Inducible and Damage-Response Pathways in Chronic Graft-versus-Host Disease. J Immunol. 2016;197(9):3490–503.

9. Kohrt HE, Tian L, Li L, Alizadeh AA, Hsieh S, Tibshirani RJ, et al. Identification of gene microarray expression profiles in patients with chronic graft-versus-host disease following allogeneic hematopoietic cell transplantation. Clin Immunol. 2013;148(1):124–35.

10. Kolde R, Laur S, Adler P, Vilo J. Robust rank aggregation for gene list integration and meta-analysis. Bioinformatics. 2012;28(4):573–80.

11. Yu G, Wang L-G, Han Y, He Q-Y. clusterProfiler: an R Package for Comparing Biological Themes Among Gene Clusters. Omi A J Integr Biol. 2012;16(5):284–7.

12. Cocho L, Fernández I, Calonge M, Martínez V, González-García MJ, Caballero D, et al. Gene Expression-Based Predictive Models of Graft Versus Host Disease-Associated Dry Eye. Invest Ophthalmol Vis Sci. 2015;56(8):4570–81.

13. Fan X, Guo D, Cheung AMS, Poon ZY, Yap CS, Goh SE, et al. Mesenchymal Stromal Cell (MSC)-Derived Combination of CXCL5 and Anti-CCL24 Is Synergistic and Superior to MSC and Cyclosporine for the Treatment of Graft-versus-Host Disease. Biol Blood Marrow Transplant. 2018;24(10):1971–80.

14. Li Y, Guan X, Liu W, Chen H-L, Truscott J, Beyatli S, et al. Helminth-Induced Production of TGF-β and Suppression of Graft-versus-Host Disease Is Dependent on IL-4 Production by Host Cells. J Immunol. 2018;201(10):2910–22.

15. Reddy P, Maeda Y, Hotary K, Liu C, Reznikov LL, Dinarello CA, et al. Histone deacetylase inhibitor suberoylanilide hydroxamic acid reduces acute graft-versus-host disease and preserves graft-versus-leukemia effect. Proc Natl Acad Sci U S A. 2004;101(11):3921–6.

16. Choi SW, Braun T, Henig I, Gatza E, Magenau J, Parkin B, et al. Vorinostat plus tacrolimus/methotrexate to prevent GVHD after myeloablative conditioning, unrelated donor HCT. Blood. 2017;130(15):1760–7.

17. Vodanovic-Jankovic S, Hari P, Jacobs P, Komorowski R, Drobyski WR. NF-kappaB as a target for the prevention of graft-versus-host disease: comparative efficacy of bortezomib and PS-1145. Blood. 2006;107(2):827–34.

18. Hill GR, Cooke KR, Teshima T, Crawford JM, Keith JC, Brinson YS, et al. Interleukin-11 promotes T cell polarization and prevents acute graft-versus-host disease after allogeneic bone marrow transplantation. J Clin Invest. 1998;102(1):115–23.

19. Teshima T, Hill GR, Pan L, Brinson YS, van den Brink MR, Cooke KR, et al. IL-11 separates graft-versus-leukemia effects from graft-versus-host disease after bone marrow transplantation. J Clin Invest. 1999;104(3):317–25.

20. Blaser BW, Roychowdhury S, Kim DJ, Schwind NR, Bhatt D, Yuan W, et al. Donor-derived IL-15 is critical for acute allogeneic graft-versus-host disease. Blood. 2005;105(2):894–901.

21. Pidala J, Sigdel TK, Wang A, Hsieh S, Inamoto Y, Martin PJ, et al. A combined biomarker and clinical panel for chronic graft versus host disease diagnosis. J Pathol Clin Res. 2017;3(1):3–16.

22. Atarod S, Ahmed MM, Lendrem C, Pearce KF, Cope W, Norden J, et al. miR-146a and miR-155 Expression Levels in Acute Graft-Versus-Host Disease Incidence. Front Immunol. 2016;7:56.

23. Spoerl S, Mathew NR, Bscheider M, Schmitt-Graeff A, Chen S, Mueller T, et al. Activity of therapeutic JAK 1/2 blockade in graft-versus-host disease. Blood. 2014;123(24):3832–42.

24. Choi J, Cooper ML, Alahmari B, Ritchey J, Collins L, Holt M, et al. Pharmacologic blockade of JAK1/JAK2 reduces GvHD and preserves the graft-versus-leukemia effect. Boussiotis VA, editor. PLoS One. 2014;9(10):e109799.

25. Carniti C, Gimondi S, Vendramin A, Recordati C, Confalonieri D, Bermema A, et al. Pharmacologic Inhibition of JAK1/JAK2 Signaling Reduces Experimental Murine Acute GVHD While Preserving GVT Effects. Clin Cancer Res. 2015;21(16):3740–9.

26. Zeiser R, Burchert A, Lengerke C, Verbeek M, Maas-Bauer K, Metzelder SK, et al. Ruxolitinib in corticosteroid-refractory graft-versus-host disease after allogeneic stem cell transplantation: a multicenter survey. Leukemia. 2015 Oct;29(10):2062–8.

27. Pabbisetty SK, Rabacal W, Volanakis EJ, Parekh V V., Olivares-Villagómez D, Cendron D, et al. Peripheral tolerance can be modified by altering KLF2-regulated Treg migration. Proc Natl Acad Sci. 2016;113(32):E4662–70.

28. Srinivasan M, Flynn R, Price A, Ranger A, Browning JL, Taylor PA, et al. Donor B-cell alloantibody deposition and germinal center formation are required for the development of murine chronic GVHD and bronchiolitis obliterans. Blood. 2012;119(6):1570–80.

29. Xu Y, Flies AS, Flies DB, Zhu G, Anand S, Flies SJ, et al. Selective targeting of the LIGHT-HVEM costimulatory system for the treatment of graft-versus-host disease. Blood. 2007;109(9):4097–104.

30. Brown GR, Lee EL, El-Hayek J, Kintner K, Luck C. IL-12-independent LIGHT signaling enhances MHC class II disparate CD4+ T cell alloproliferation, IFN-gamma responses, and intestinal graft-versus-host disease. J Immunol. 2005;174(8):4688–95.

31. Tamada K, Tamura H, Flies D, Fu Y-X, Celis E, Pease LR, et al. Blockade of LIGHT/LTbeta and CD40 signaling induces allospecific T cell anergy, preventing graft-versus-host disease. J Clin Invest. 2002;109(4):549–57.

32. Yamakawa T, Ohigashi H, Hashimoto D, Hayase E, Takahashi S, Miyazaki M, et al. Vitamin A-coupled liposomes containing siRNA against HSP47 ameliorate skin fibrosis in chronic graft-versus-host disease. Blood. 2018;131(13):1476–85.

33. Iyengar S, Zhan C, Lu J, Korngold R, Schwartz DH. Treatment with a rho kinase inhibitor improves survival from graft-versus-host disease in mice after MHC-haploidentical hematopoietic cell transplantation. Biol Blood Marrow Transplant. 2014;20(8):1104–11.

34. Chopra M, Biehl M, Steinfatt T, Brandl A, Kums J, Amich J, et al. Exogenous TNFR2 activation protects from acute GvHD via host T reg cell expansion. J Exp Med. 2016;213(9):1881–900.

35. Siegmund D, Ehrenschwender M, Wajant H. TNFR2 unlocks a RIPK1 kinase activity-dependent mode of proinflammatory TNFR1 signaling. Cell Death Dis. 2018;9(9):921.

36. Ma H-H, Ziegler J, Li C, Sepulveda A, Bedeir A, Grandis J, et al. Sequential activation of inflammatory signaling pathways during graft-versus-host disease (GVHD): Early role for STAT1 and STAT3. Cell Immunol. 2011;268(1):37–46.

37. Brestoff JR, Vessoni AT, Brenner KA, Uy GL, DiPersio JF, Blinder M, et al. Acute graft-versus-host disease following lung transplantation in a patient with a novel TERT mutation. Thorax. 2018;73(5):489–92.

38. Häcker H, Redecke V, Blagoev B, Kratchmarova I, Hsu L-C, Wang GG, et al. Specificity in Toll-like receptor signalling through distinct effector functions of TRAF3 and TRAF6. Nature. 2006;439(7073):204–7.

39. Xiao HW, Luo Y, Lai XY, Shi JM, Tan YM, He JS, et al. Donor TLR9 gene tagSNPs influence susceptibility to aGVHD and CMV reactivation in the allo-HSCT setting without polymorphisms in the TLR4 and NOD2 genes. Bone Marrow Transplant. 2014;49(2):241–7.

40. Lu Y, Hippen KL, Lemire AL, Gu J, Wang W, Ni X, et al. MiR-146b antagomir-treated human Tregs acquire increased GVHD inhibitory potency. Blood. 2016;128(10):1424–35.

41. Stickel N, Prinz G, Pfeifer D, Hasselblatt P, Schmitt-Graeff A, Follo M, et al. MiR-146a regulates the TRAF6 / TNF-axis in donor T cells during GVHD. Blood. 2014;124(16):2586–96.

42. Carrillo-Cruz E, García-Lozano JR, Márquez-Malaver FJ, Sánchez-Guijo FM, Montero Cuadrado I, Ferra-i-Coll C, et al. Vitamin D modifies the incidence of graft-versus-host disease after allogeneic stem cell transplantation depending on the vitamin D receptor (VDR) polymorphisms. Clin Cancer Res. 2019;25(15):4616–23.

43. Middleton P, Cullup H, Dickinson A, Norden J, Jackson G, Taylor P, et al. Vitamin D receptor gene polymorphism associates with graft-versus-host disease and survival in HLA-matched sibling allogeneic bone marrow transplantation. Bone Marrow Transplant. 2002;30(4):223–8.
