# Supplementary material for: Meta-Analysis of Genome-Wide Association and Gene Expression Studies Implicates Donor T Cell Function and Cytokine Pathways in Acute GvHD
Source: Front Immunol. 2020 Feb 3;11:19. doi: 10.3389/fimmu.2020.00019 (PMC7008714; doi:10.3389/fimmu.2020.00019)
Supplement: Supplementary file 2 [file Data_Sheet_2.pdf]

LocusCompare plots of Finnish cohort 1

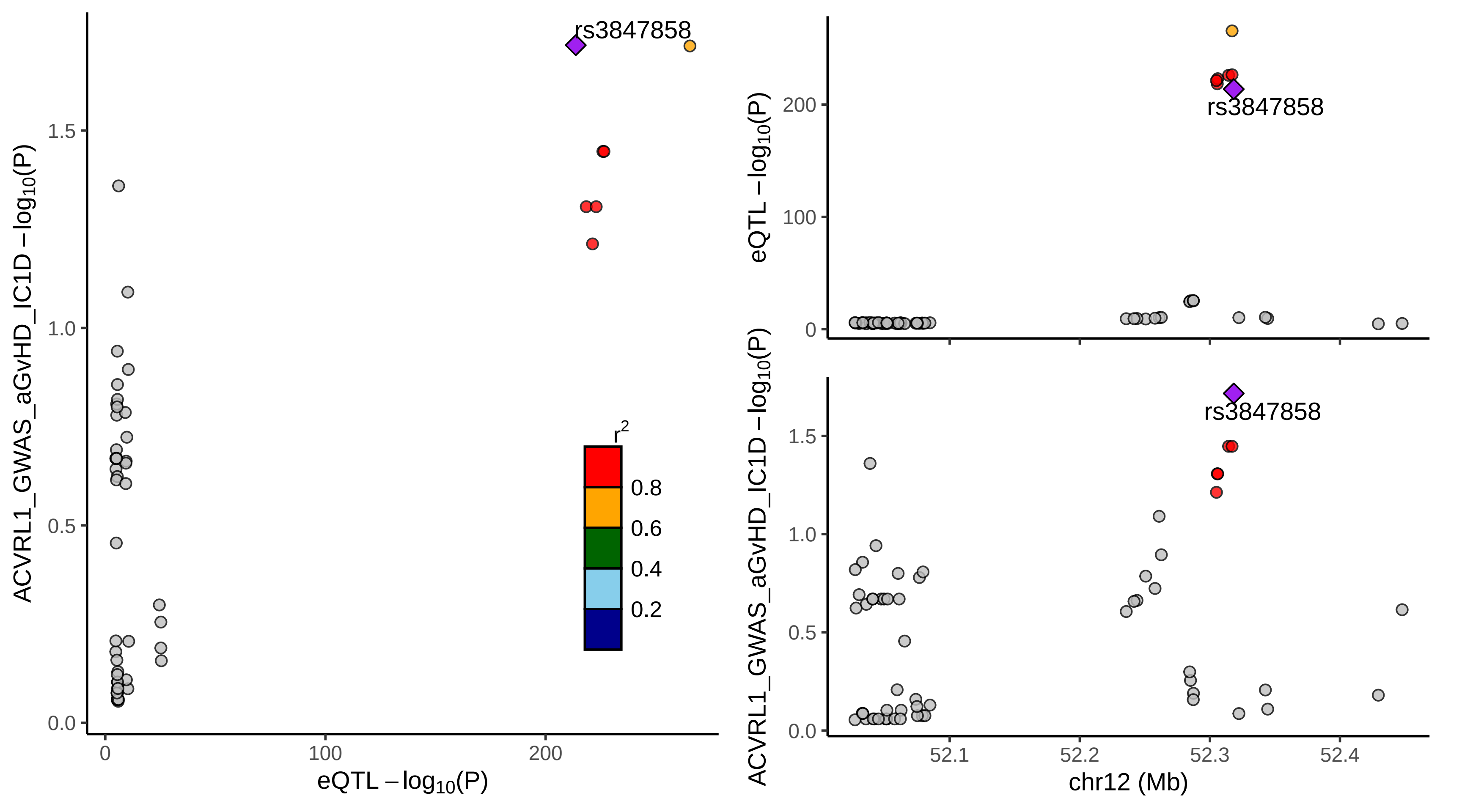

rs3847858
ACVRL1_GWAS_aGvHD_IC1D −log10(P)
eQTL −log10(P)
r2
0.8
0.6
0.4
0.2
0.0
0.5
1.0
1.5
0
100
200
rs3847858
eQTL −log10(P)
rs3847858
ACVRL1_GWAS_aGvHD_IC1D −log10(P)
52.1
52.2
52.3
52.4
chr12 (Mb)

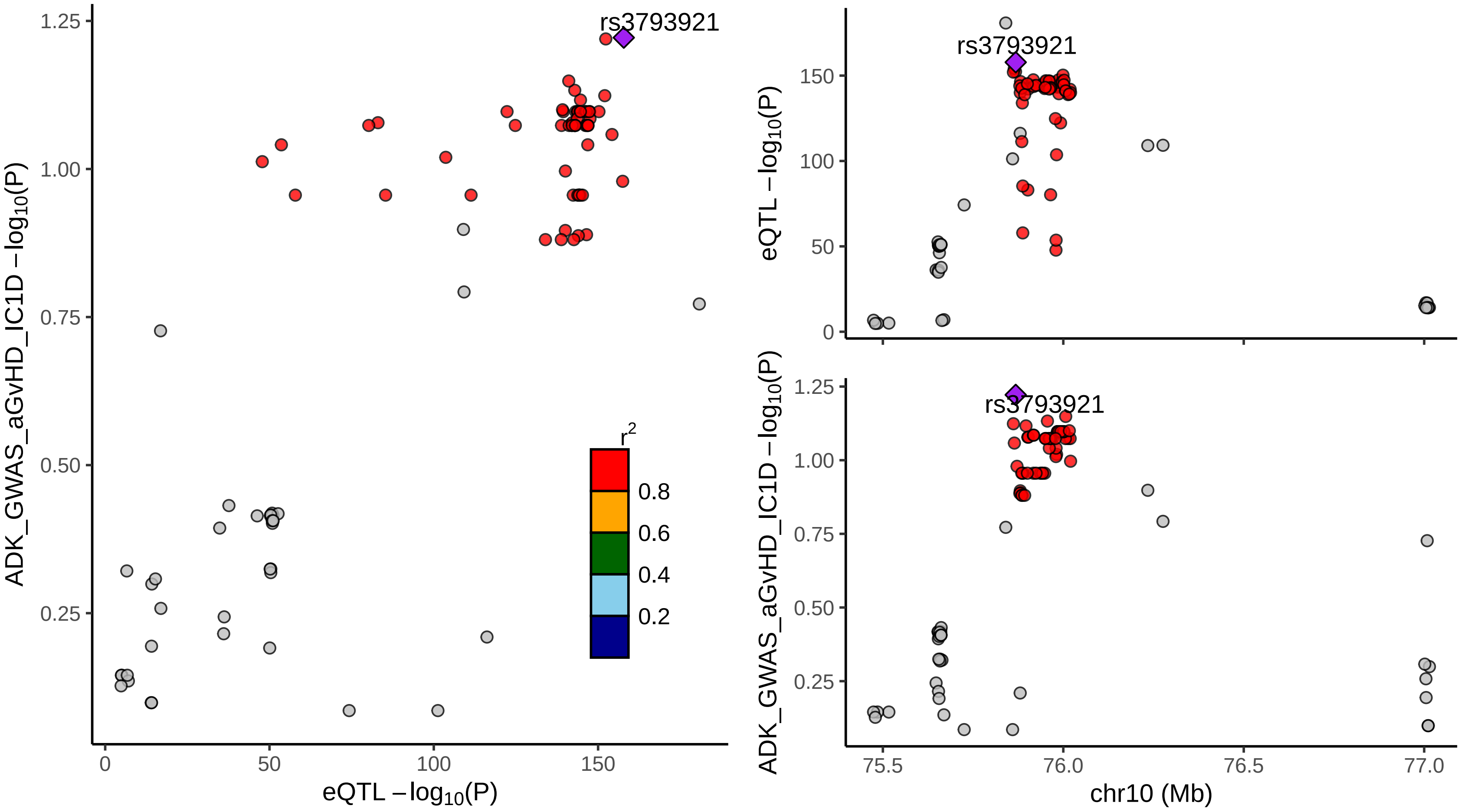

rs3793921
ADK_GWAS_aGvHD_IC1D −log10(P)
1.25
1.00
0.75
0.50
0.25
0
50
100
150
eQTL −log10(P)
r2
0.8
0.6
0.4
0.2
rs3793921
eQTL −log10(P)
150
100
50
0
rs3793921
ADK_GWAS_aGvHD_IC1D −log10(P)
1.25
1.00
0.75
0.50
0.25
75.5
76.0
76.5
77.0
chr10 (Mb)

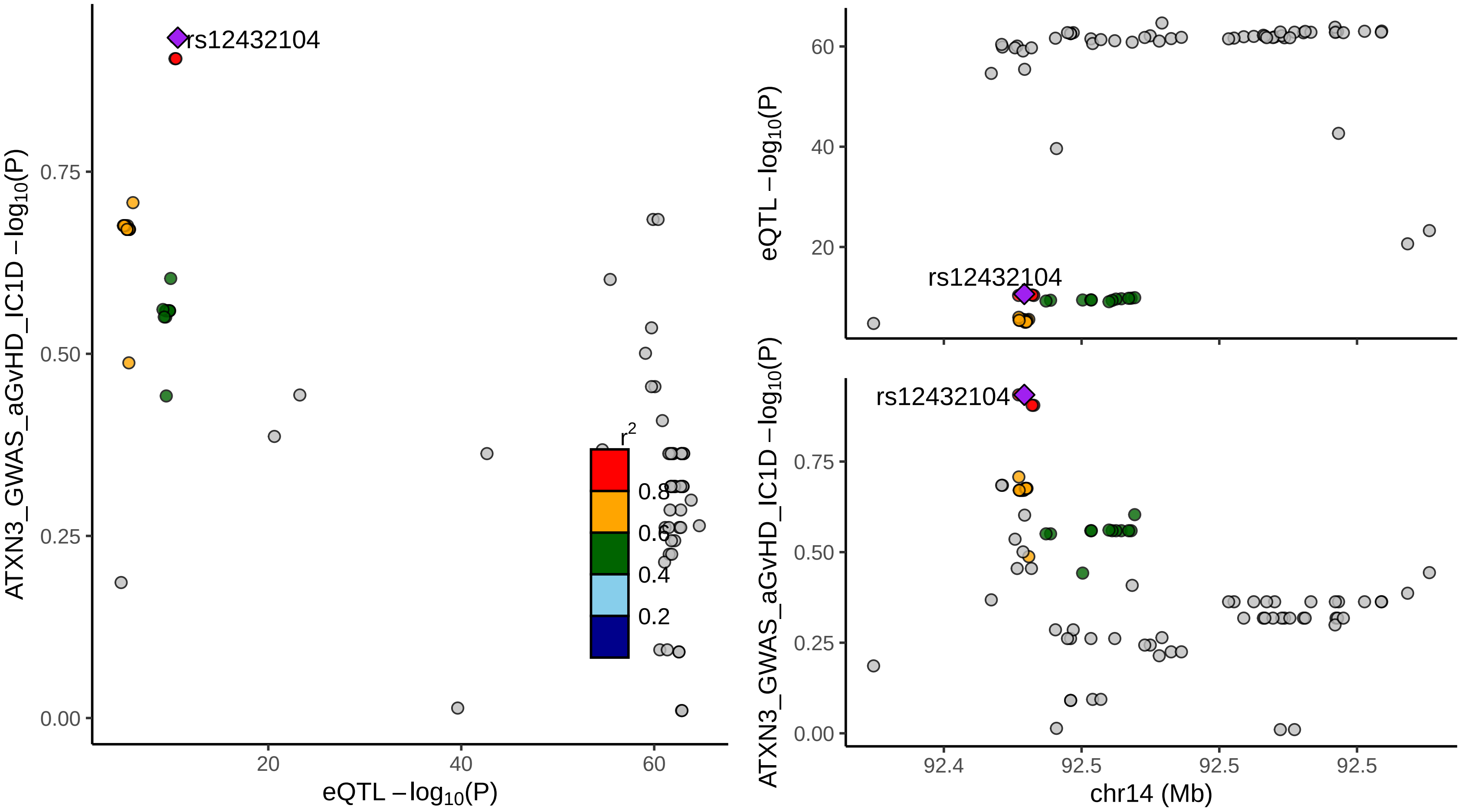

rs12432104
ATXN3_GWAS_aGvHD_IC1D −log10(P)
eQTL −log10(P)
0.00
0.25
0.50
0.75
20
40
60
r2
0.8
0.6
0.4
0.2
chr14 (Mb)
92.4
92.5

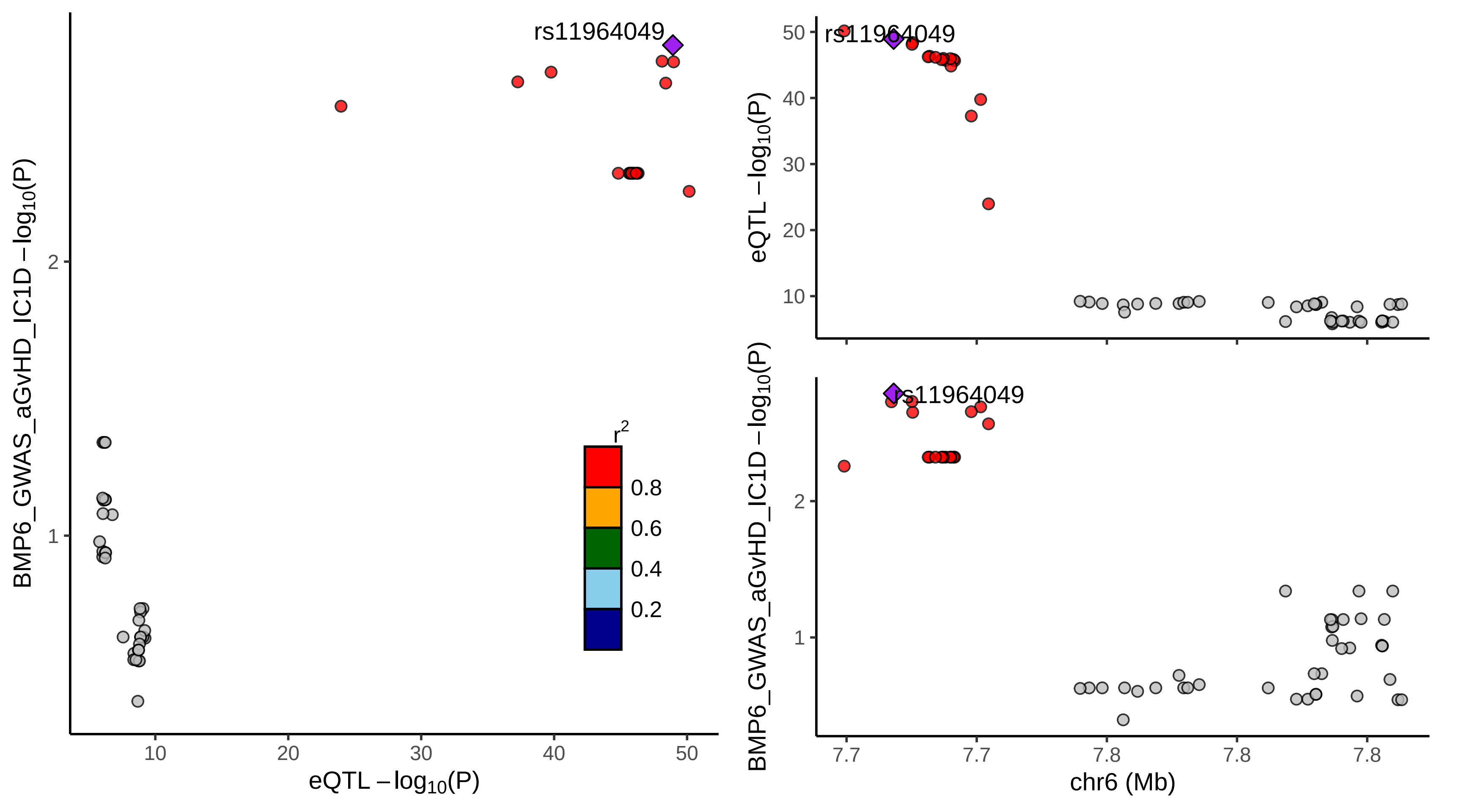

rs11964049
BMP6_GWAS_aGvHD_IC1D −log10(P)
eQTL −log10(P)
r2
0.8
0.6
0.4
0.2
rs11964049
eQTL −log10(P)
rs11964049
BMP6_GWAS_aGvHD_IC1D −log10(P)
chr6 (Mb)

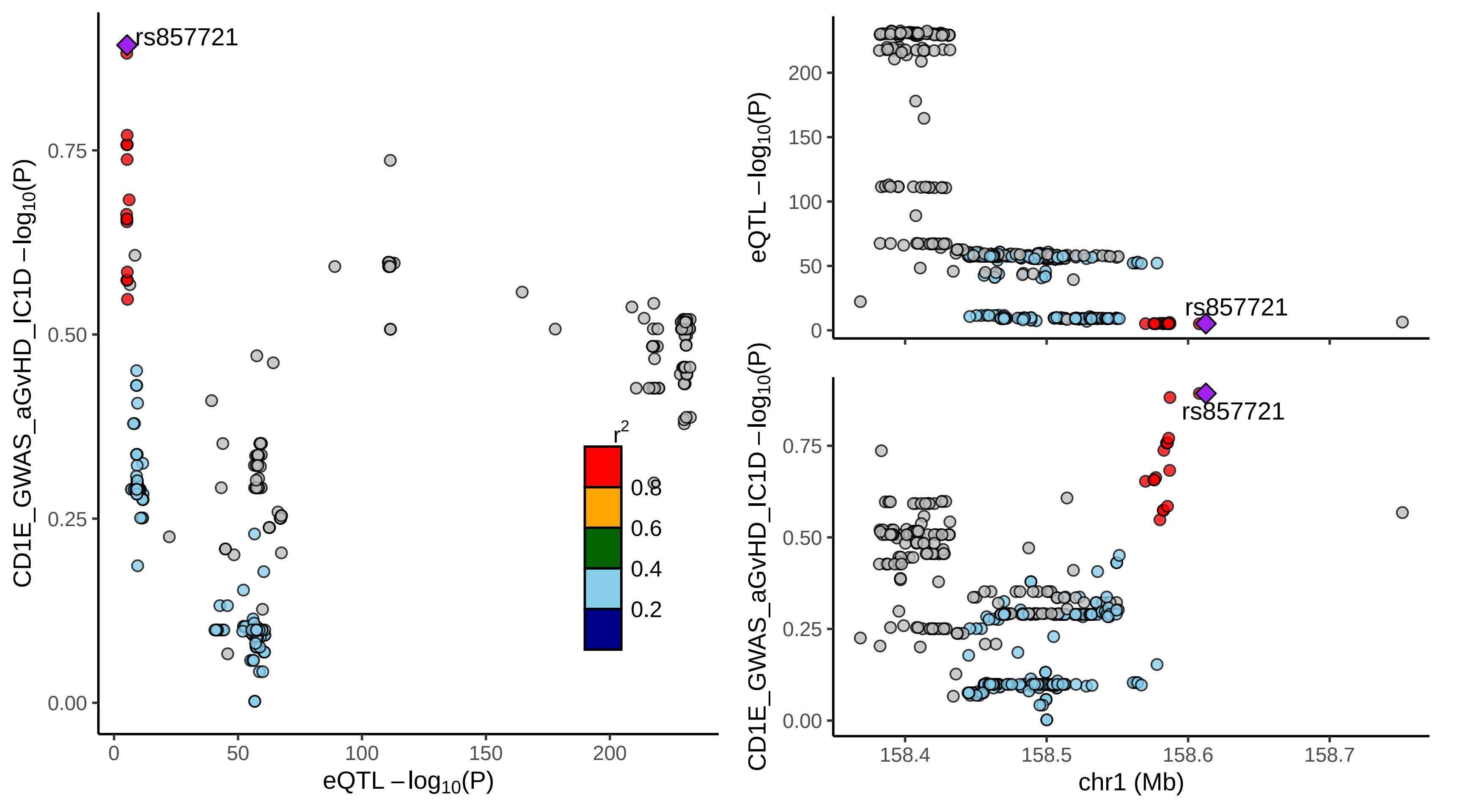
rs857721
CD1E_GWAS_aGvHD_IC1D −log10(P)
0.00
0.25
0.50
0.75
0
50
100
150
200
eQTL −log10(P)
r2
0.2
0.4
0.6
0.8
eQTL −log10(P)
rs857721
CD1E_GWAS_aGvHD_IC1D −log10(P)
rs857721
158.4
158.5
158.6
158.7
chr1 (Mb)

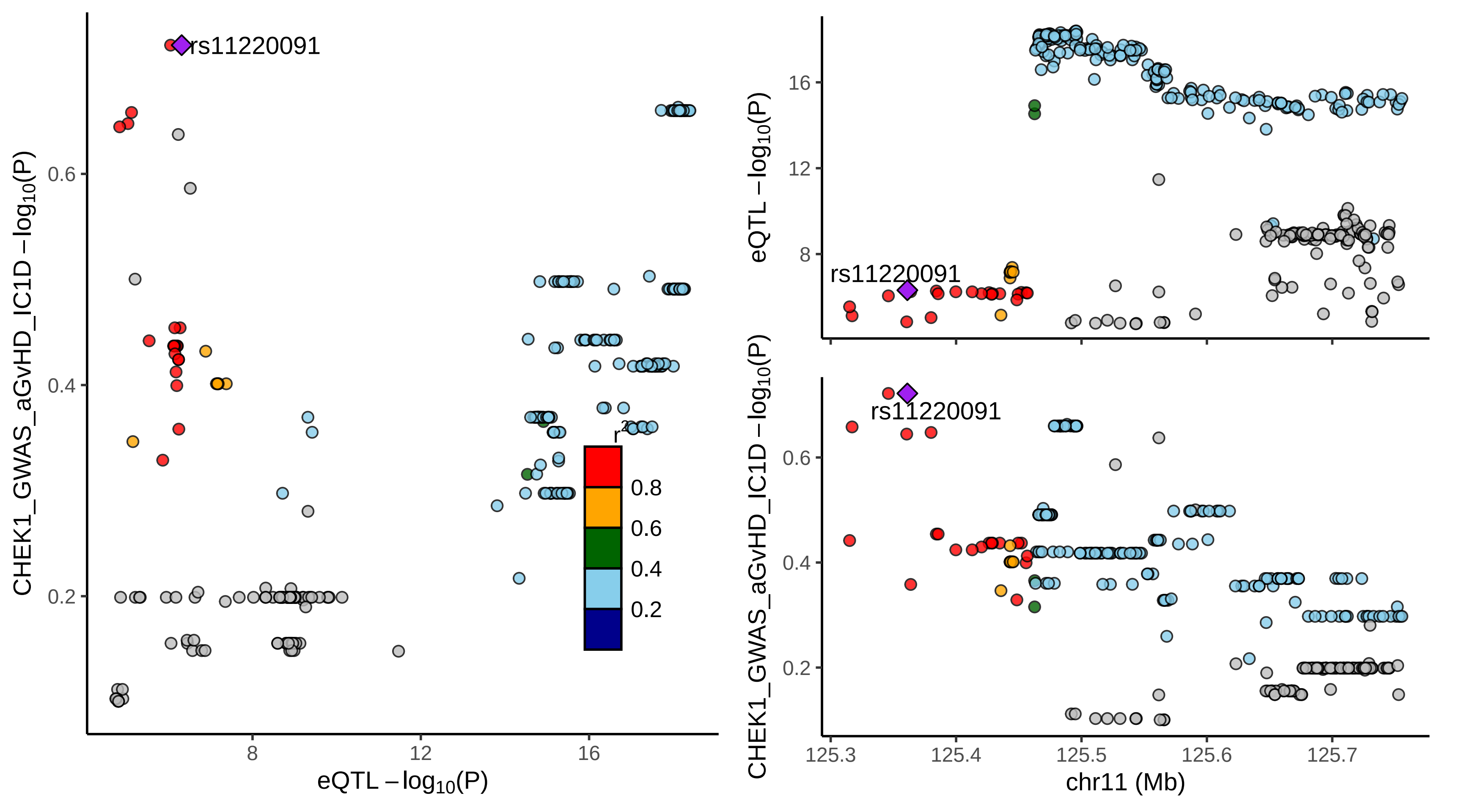

rs11220091
CHEK1_GWAS_aGvHD_IC1D −log10(P)
0.6
0.4
0.2
8
12
16
eQTL −log10(P)
r²
0.8
0.6
0.4
0.2
rs11220091
eQTL −log10(P)
16
12
8
rs11220091
CHEK1_GWAS_aGvHD_IC1D −log10(P)
0.6
0.4
0.2
125.3
125.4
125.5
125.6
125.7
chr11 (Mb)

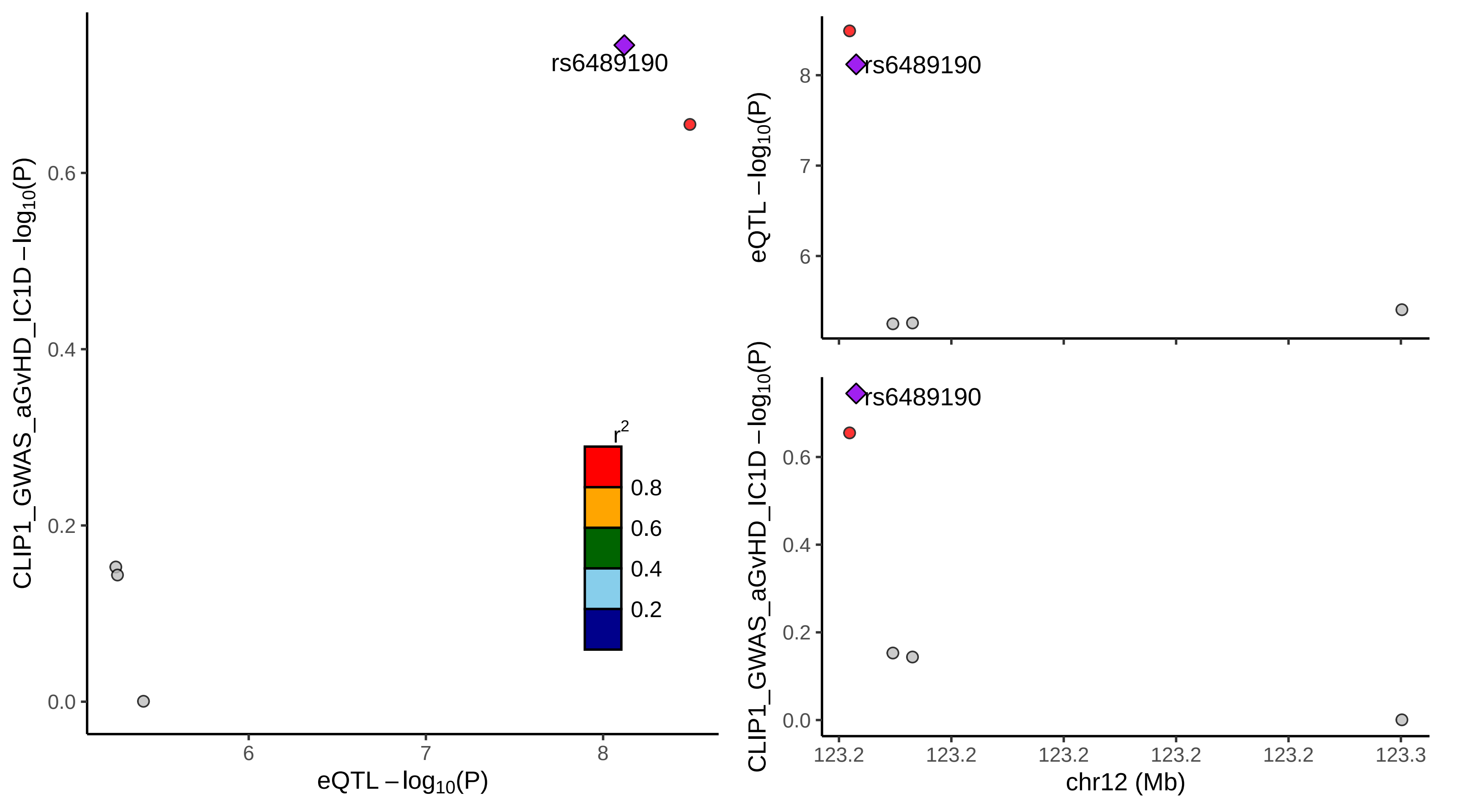

rs6489190
CLIP1_GWAS_aGvHD_IC1D −log10(P)
0.0
0.2
0.4
0.6
6
7
8
eQTL −log10(P)
r2
0.8
0.6
0.4
0.2
rs6489190
eQTL −log10(P)
6
7
8
rs6489190
CLIP1_GWAS_aGvHD_IC1D −log10(P)
0.0
0.2
0.4
0.6
123.2
123.2
123.2
123.2
123.2
123.3
chr12 (Mb)

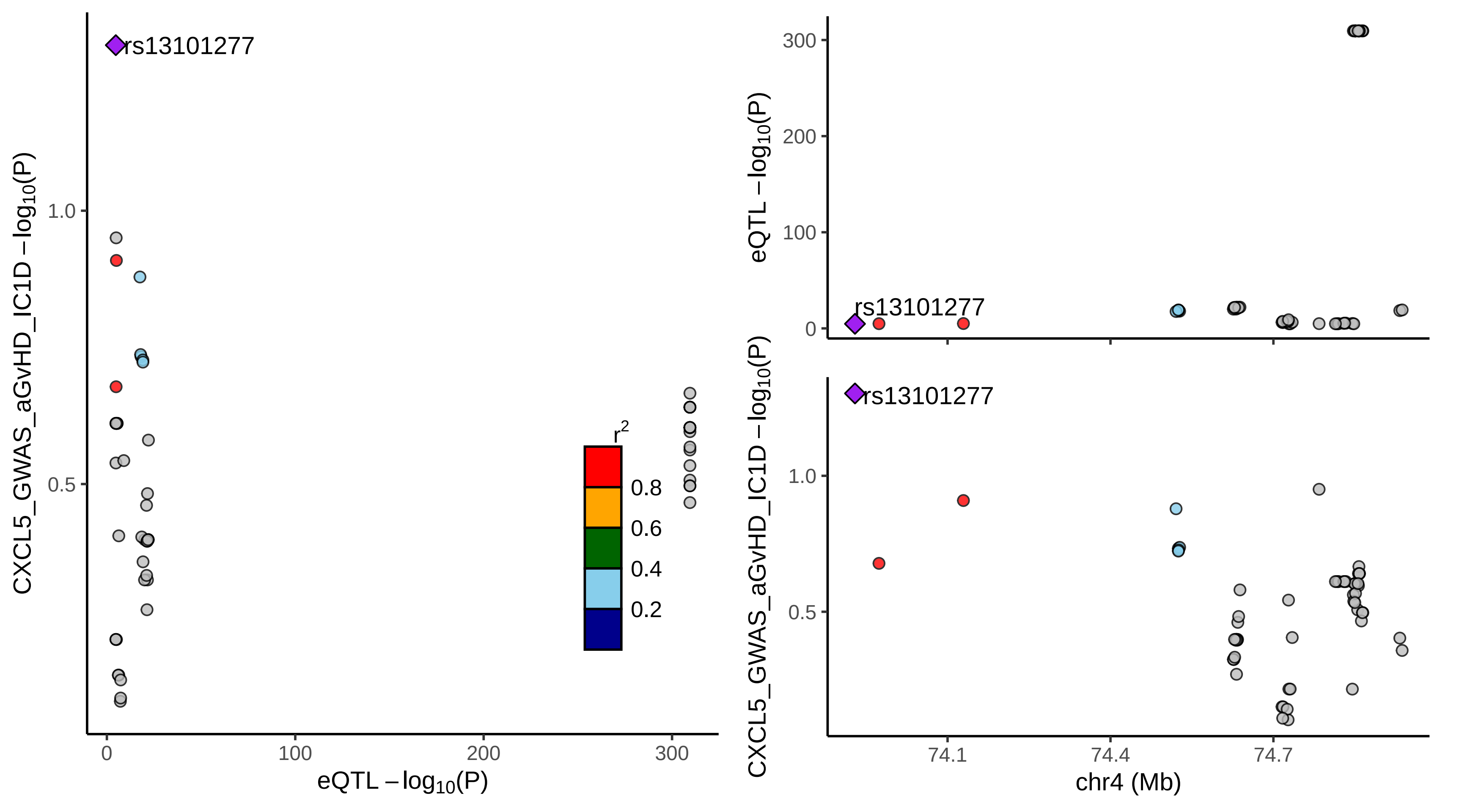

rs13101277
CXCL5_GWAS_aGvHD_IC1D −log10(P)
1.0
0.5
0
100
200
300
eQTL −log10(P)
r2
0.8
0.6
0.4
0.2
eQTL −log10(P)
300
200
100
0
rs13101277
rs13101277
CXCL5_GWAS_aGvHD_IC1D −log10(P)
1.0
0.5
74.1
74.4
74.7
chr4 (Mb)

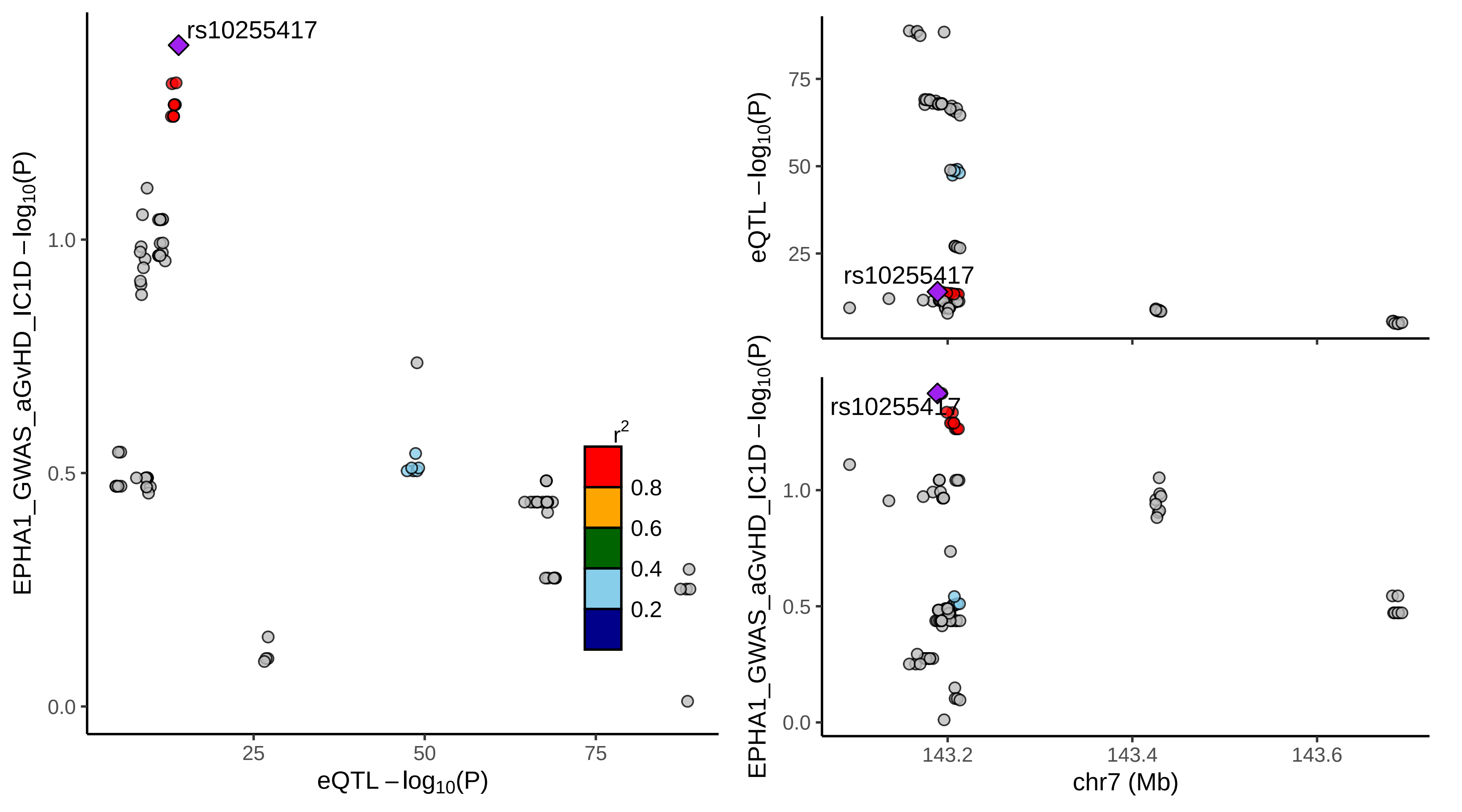

rs10255417
EPHA1_GWAS_aGvHD_IC1D −log10(P)
eQTL −log10(P)
r2
0.8
0.6
0.4
0.2
1.0
0.5
0.0
25
50
75
chr7 (Mb)
143.2
143.4
143.6

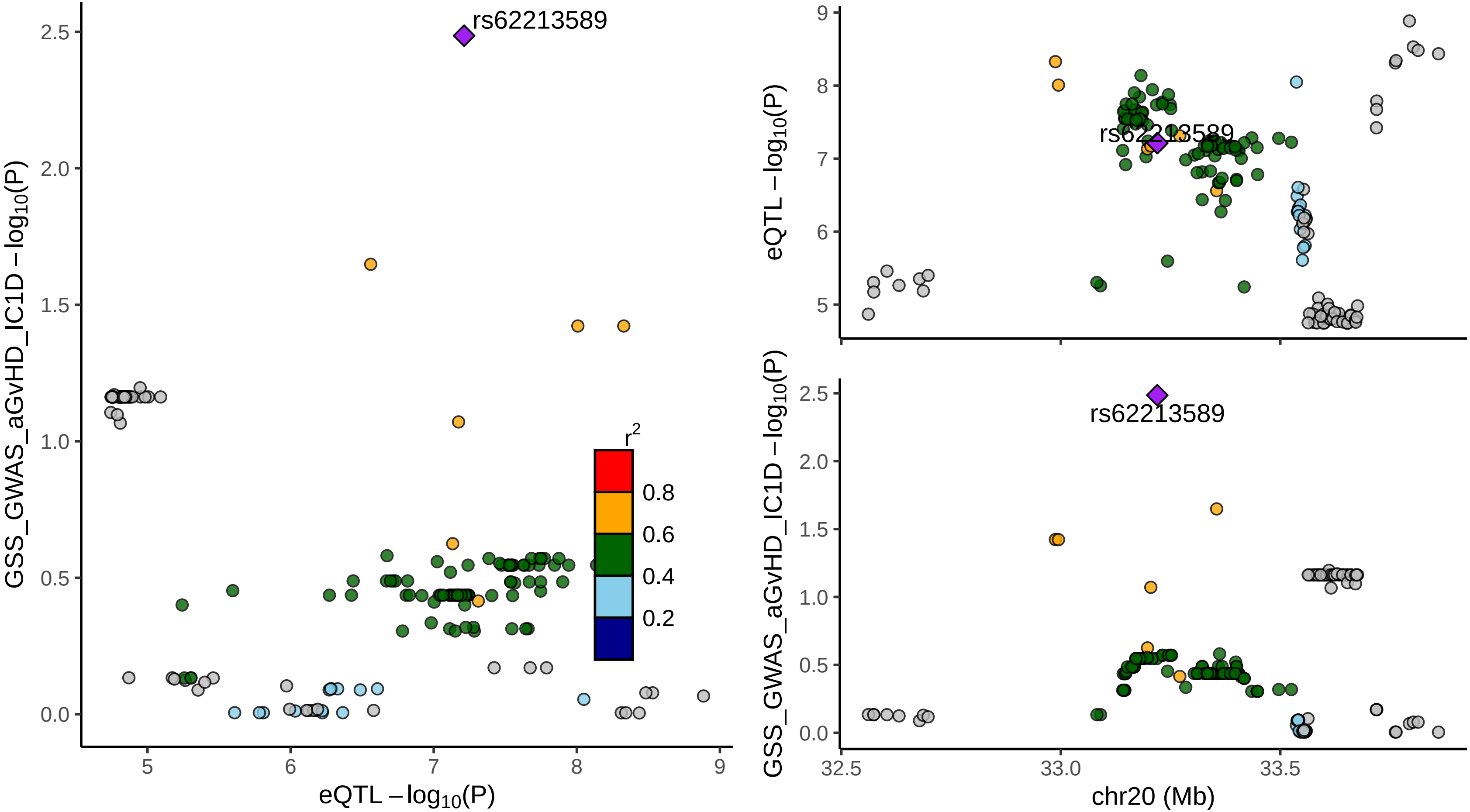
rs62213589
GSS_GWAS_aGvHD_IC1D −log10(P)
eQTL −log10(P)
r2
0.8
0.6
0.4
0.2
chr20 (Mb)

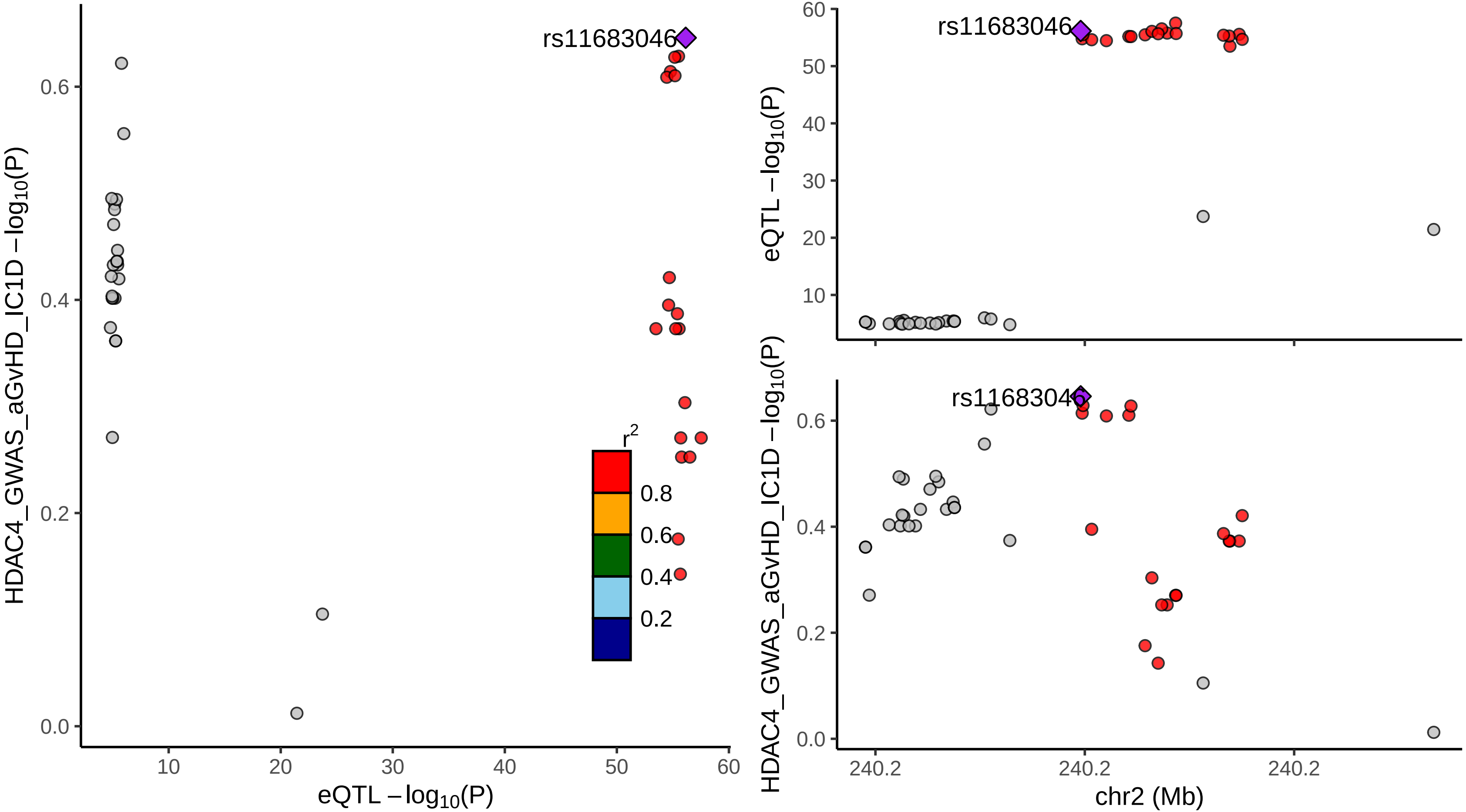

rs11683046
HDAC4_GWAS_aGvHD_IC1D −log10(P)
eQTL −log10(P)
r2
0.8
0.6
0.4
0.2
chr2 (Mb)
240.2

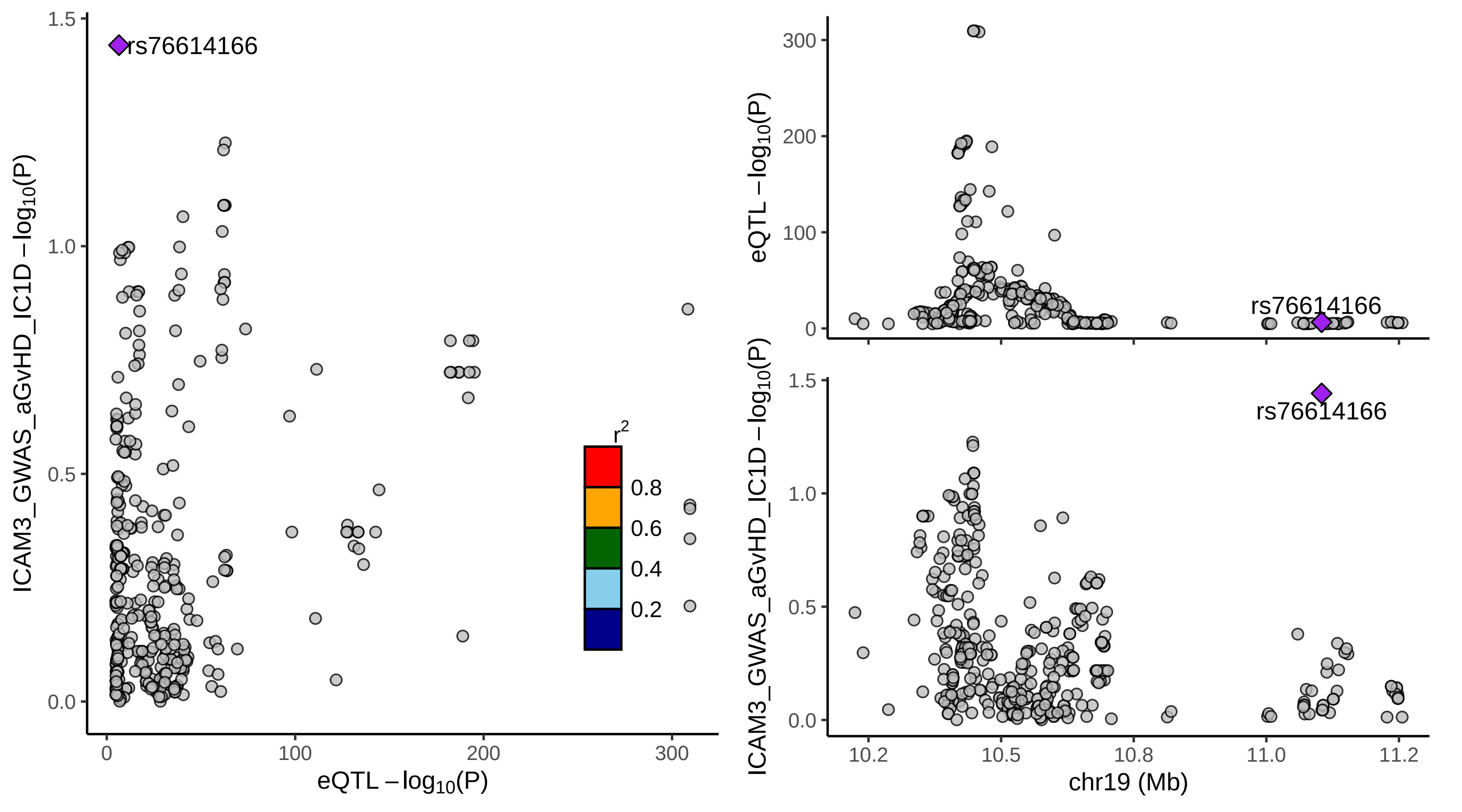

rs76614166
ICAM3_GWAS_aGvHD_IC1D −log10(P)
eQTL −log10(P)
r2
0.8
0.6
0.4
0.2
0.0
0.5
1.0
1.5
0
100
200
300
rs76614166
eQTL −log10(P)
rs76614166
ICAM3_GWAS_aGvHD_IC1D −log10(P)
10.2
10.5
10.8
11.0
11.2
chr19 (Mb)

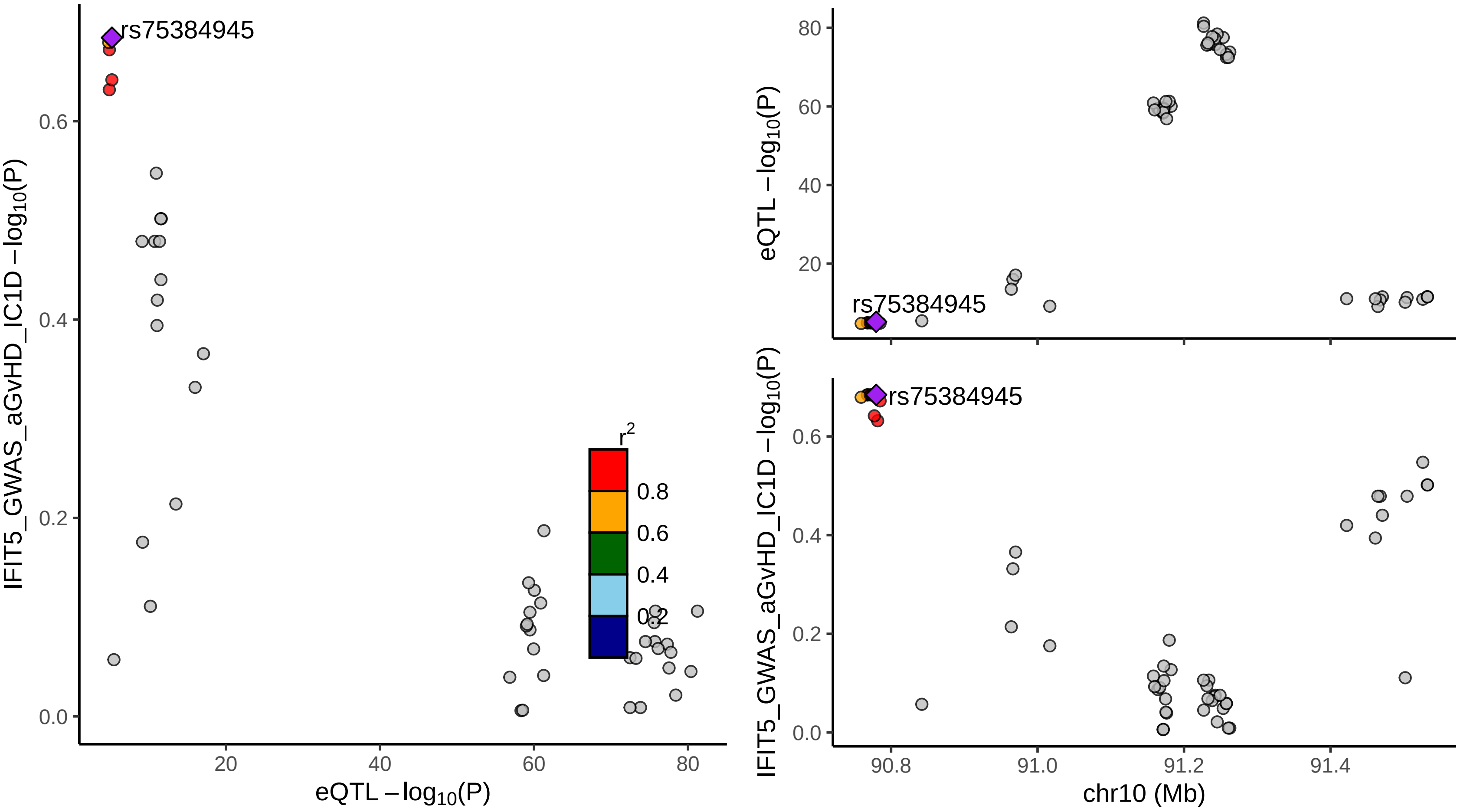

rs75384945
IFIT5_GWAS_aGvHD_IC1D −log10(P)
eQTL −log10(P)
0.0
0.2
0.4
0.6
20
40
60
80
r2
0.8
0.6
0.4
0.2
rs75384945
eQTL −log10(P)
rs75384945
IFIT5_GWAS_aGvHD_IC1D −log10(P)
90.8
91.0
91.2
91.4
chr10 (Mb)

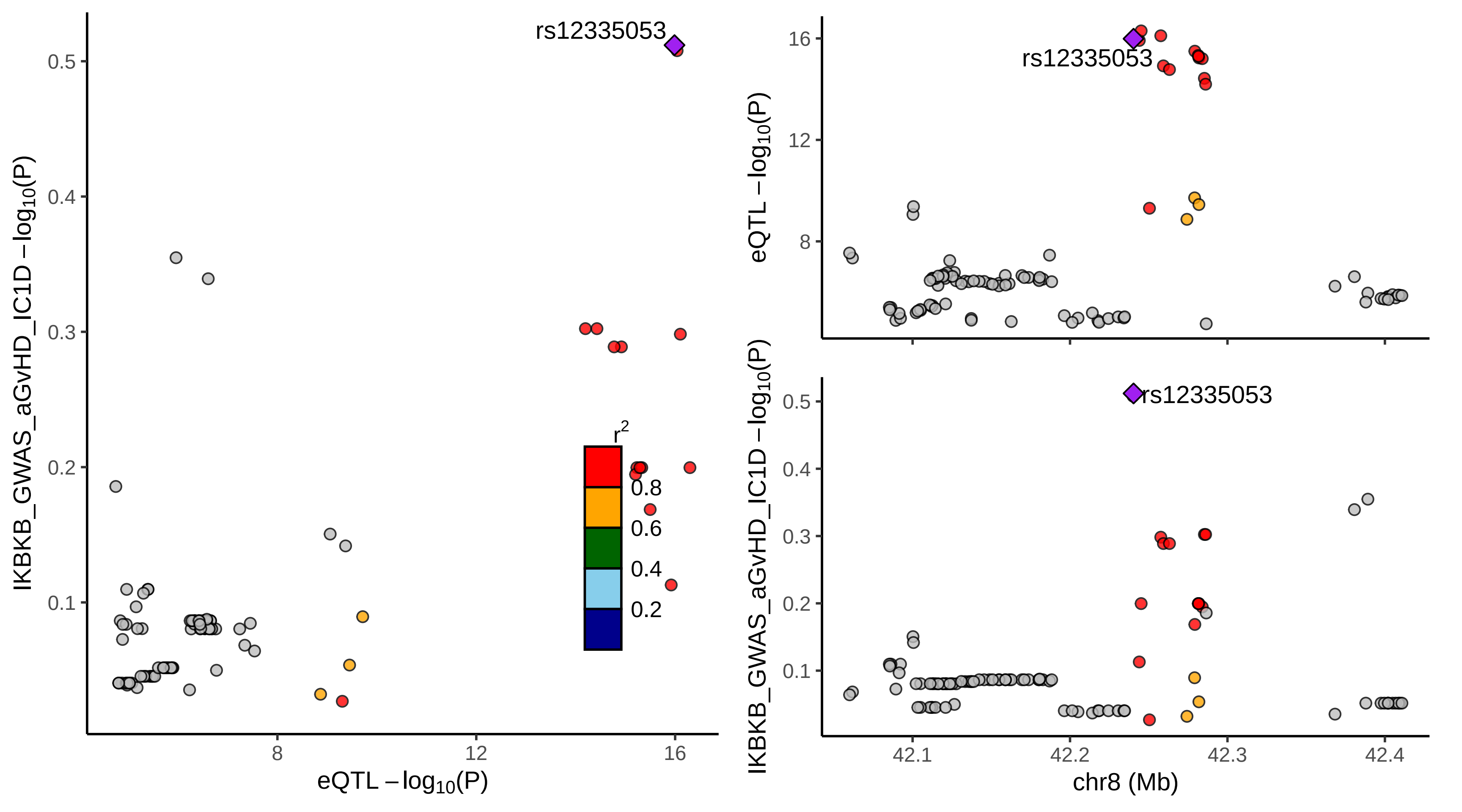
rs12335053
IKBKB_GWAS_aGvHD_IC1D −log10(P)
eQTL −log10(P)
r2
0.8
0.6
0.4
0.2
0.1
0.2
0.3
0.4
0.5
8
12
16
chr8 (Mb)
42.1
42.2
42.3
42.4

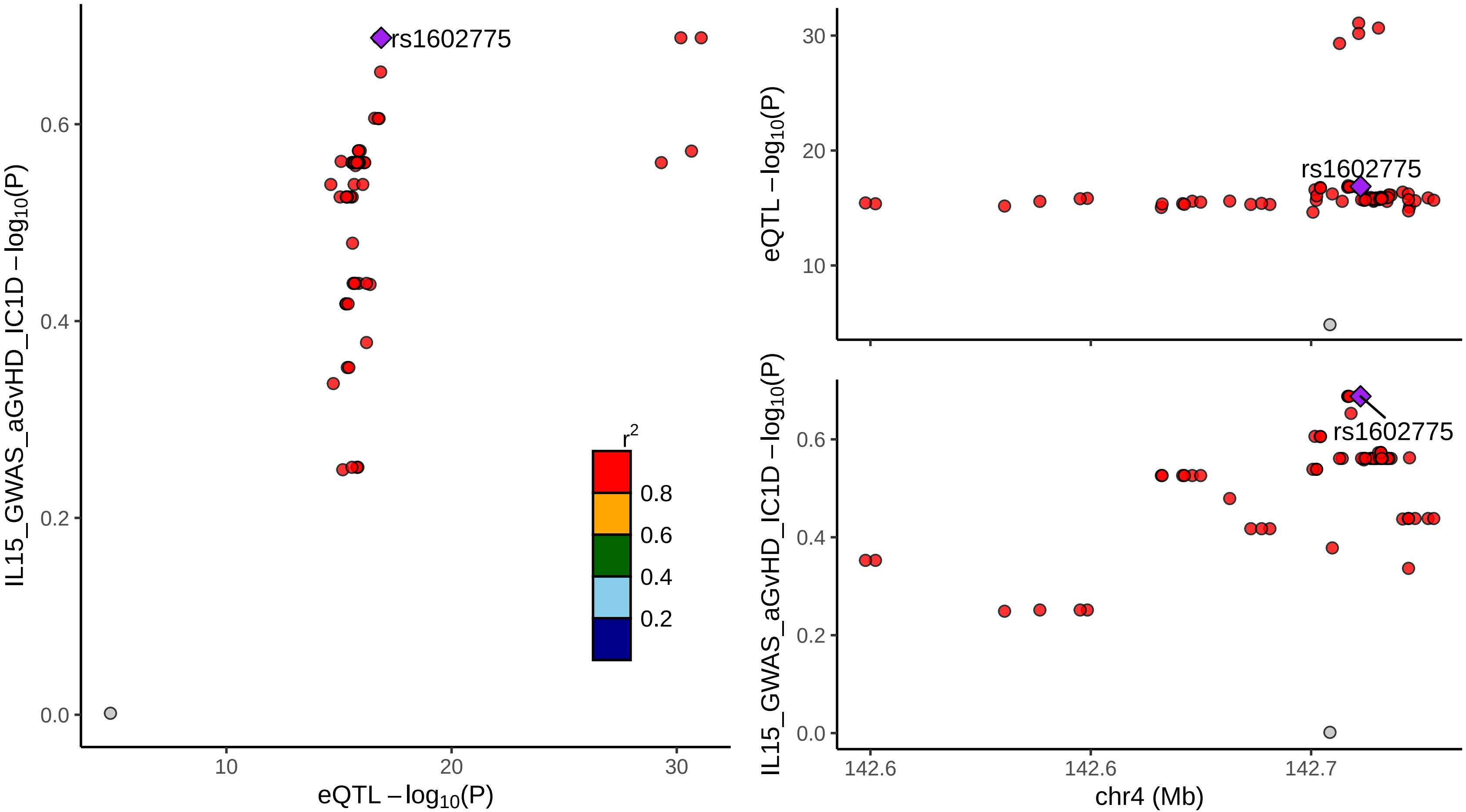

rs1602775
IL15_GWAS_aGvHD_IC1D −log10(P)
eQTL −log10(P)
r2
0.8
0.6
0.4
0.2
0.0
10
20
30
rs1602775
eQTL −log10(P)
rs1602775
IL15_GWAS_aGvHD_IC1D −log10(P)
142.6
142.7
chr4 (Mb)

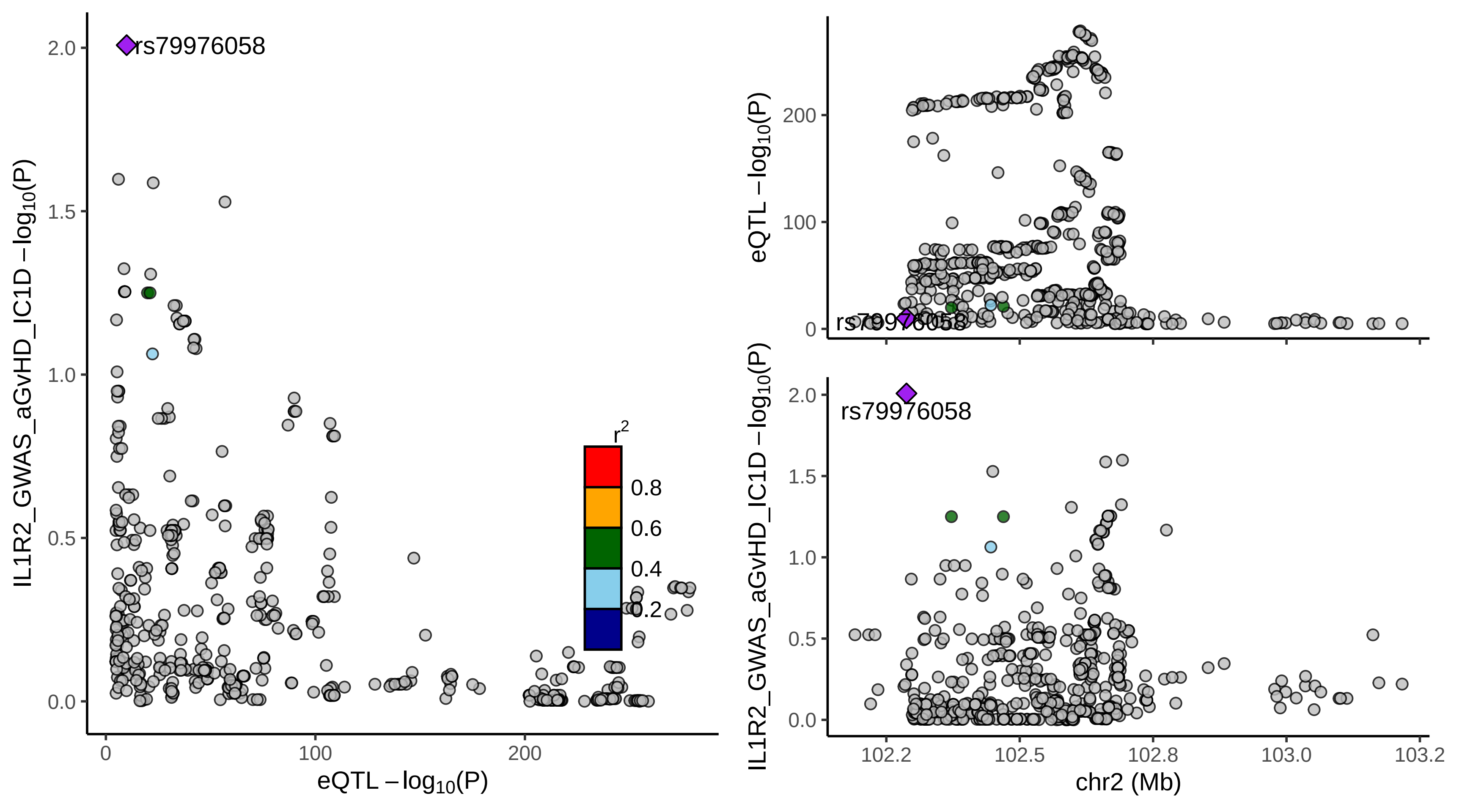
rs79976058
IL1R2_GWAS_aGvHD_IC1D −log10(P)
eQTL −log10(P)
r2
0.8
0.6
0.4
0.2
chr2 (Mb)
102.2
102.5
102.8
103.0
103.2

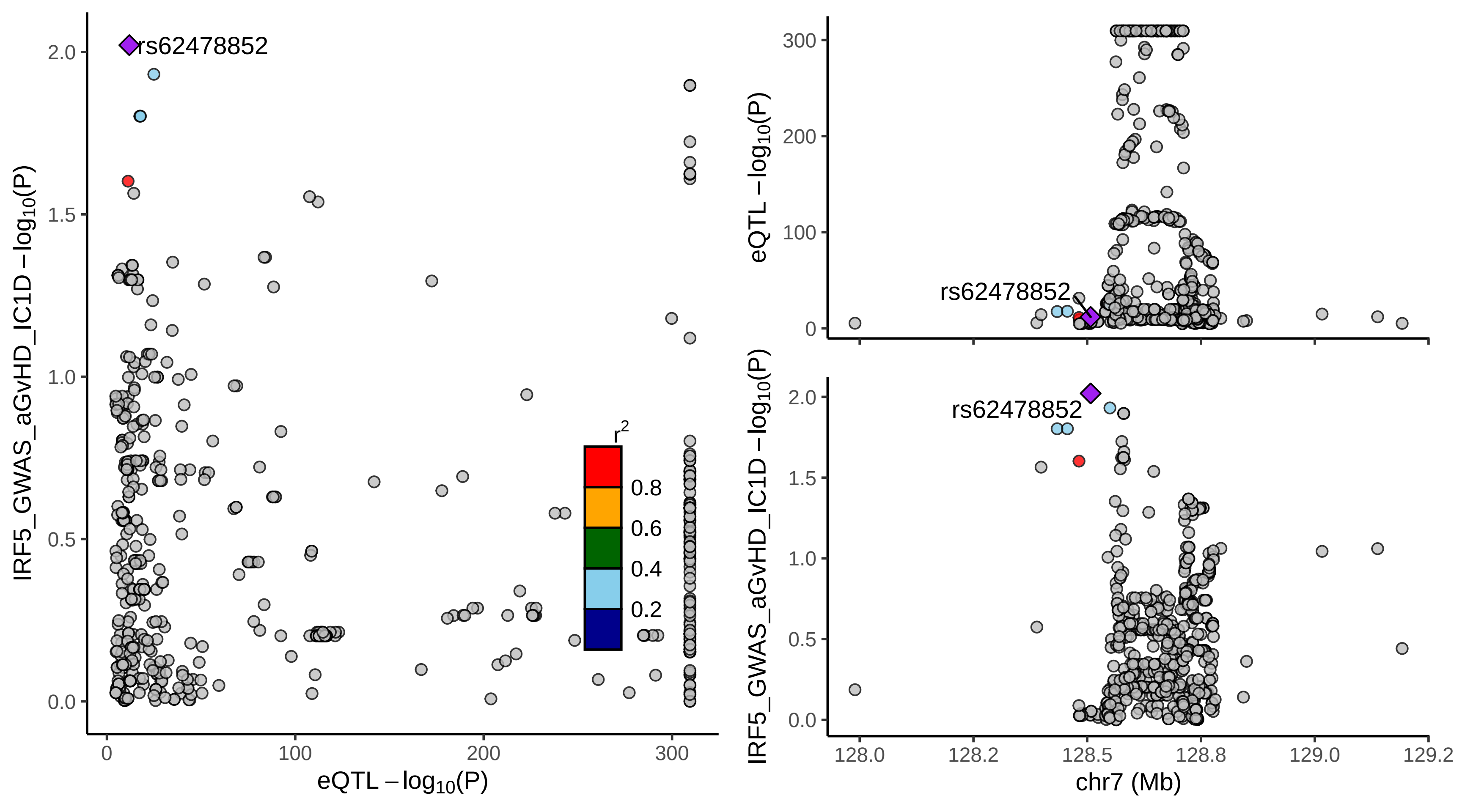

rs62478852
IRF5_GWAS_aGvHD_IC1D −log10(P)
eQTL −log10(P)
r2
0.8
0.6
0.4
0.2
eQTL −log10(P)
rs62478852
IRF5_GWAS_aGvHD_IC1D −log10(P)
rs62478852
chr7 (Mb)

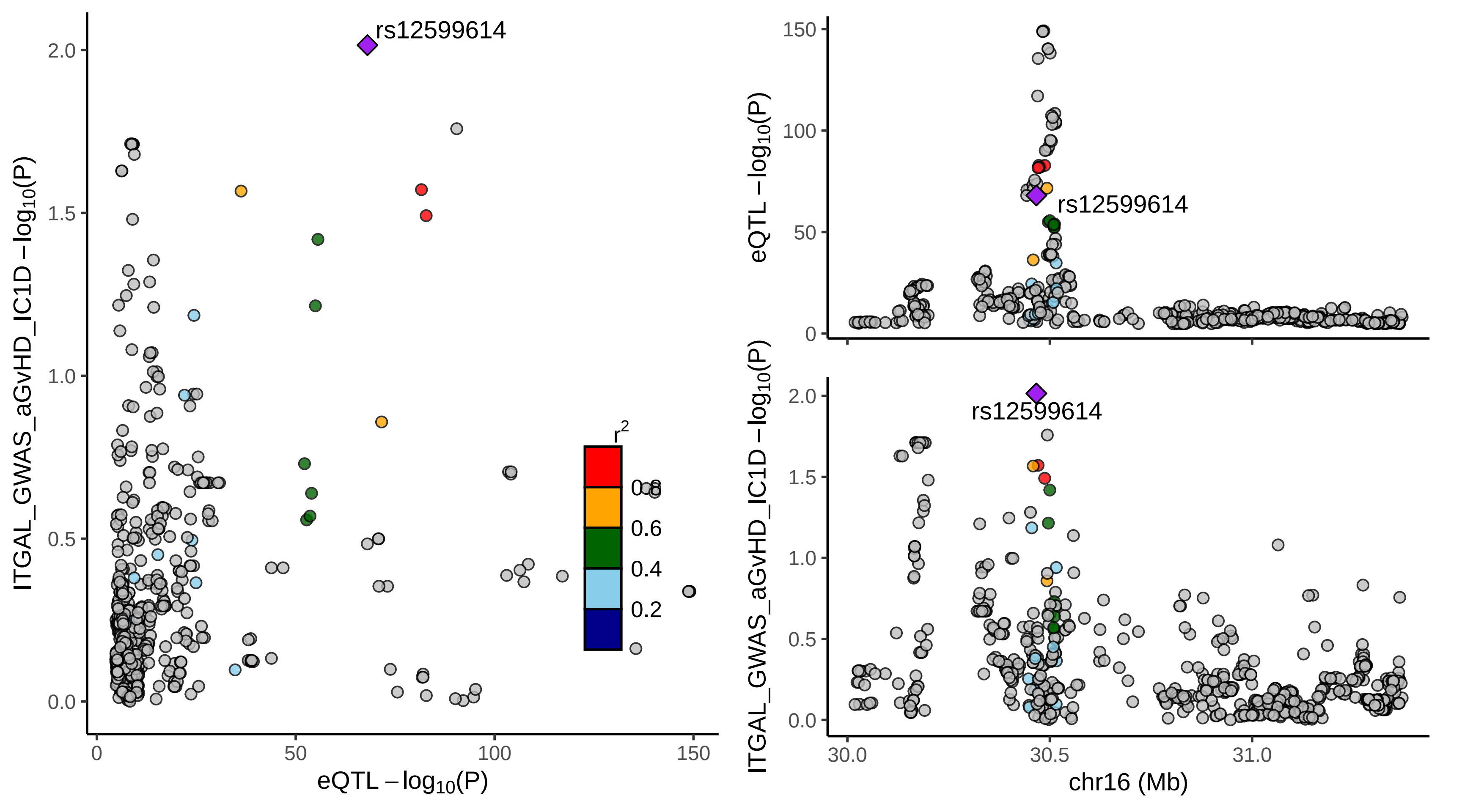

rs12599614
ITGAL_GWAS_aGvHD_IC1D −log10(P)
eQTL −log10(P)
r2
0.8
0.6
0.4
0.2
chr16 (Mb)

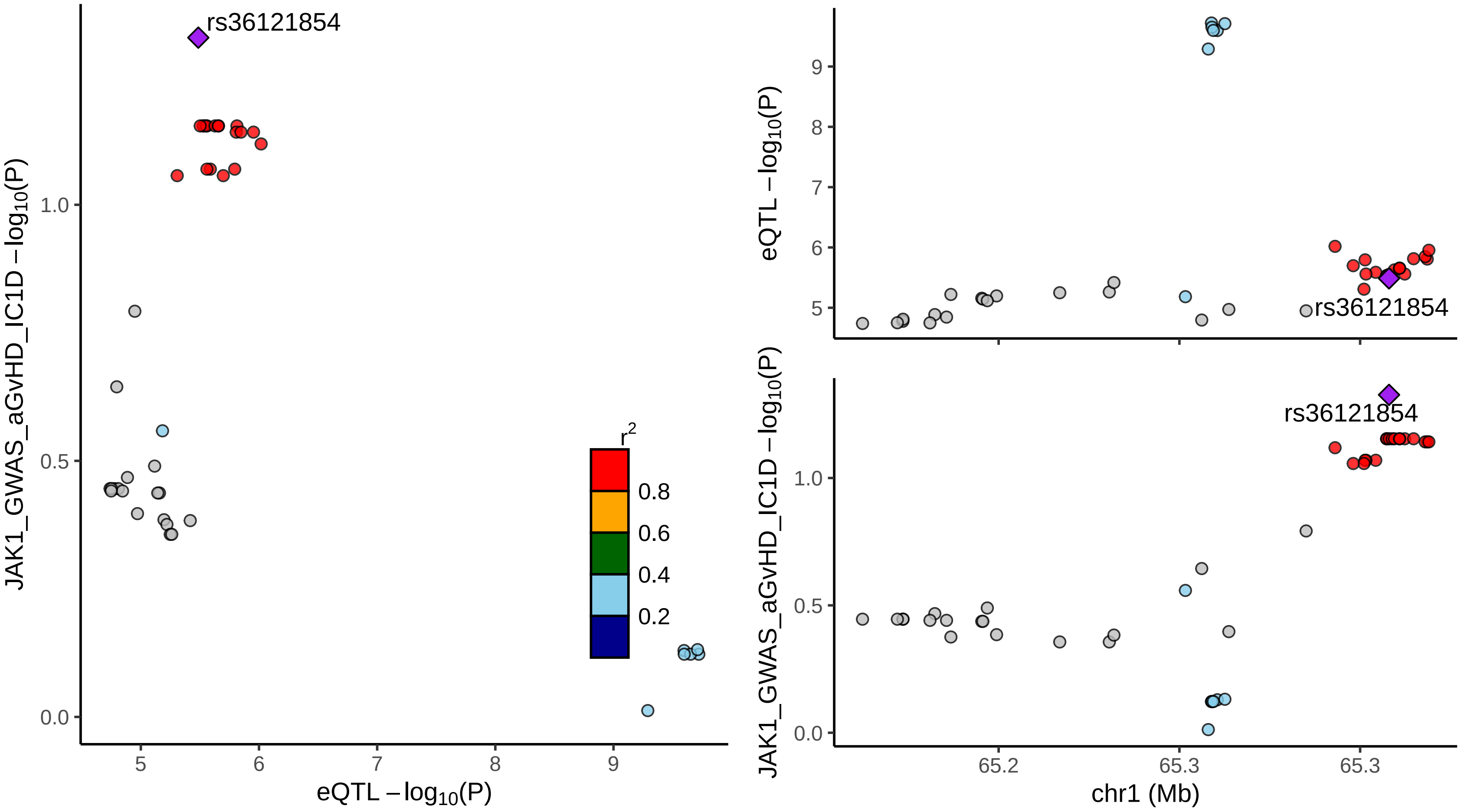

rs36121854
JAK1_GWAS_aGvHD_IC1D −log10(P)
1.0
0.5
0.0
5
6
7
8
9
eQTL −log10(P)
r2
0.8
0.6
0.4
0.2
eQTL −log10(P)
9
8
7
6
5
rs36121854
JAK1_GWAS_aGvHD_IC1D −log10(P)
rs36121854
1.0
0.5
0.0
65.2
65.3
65.3
chr1 (Mb)

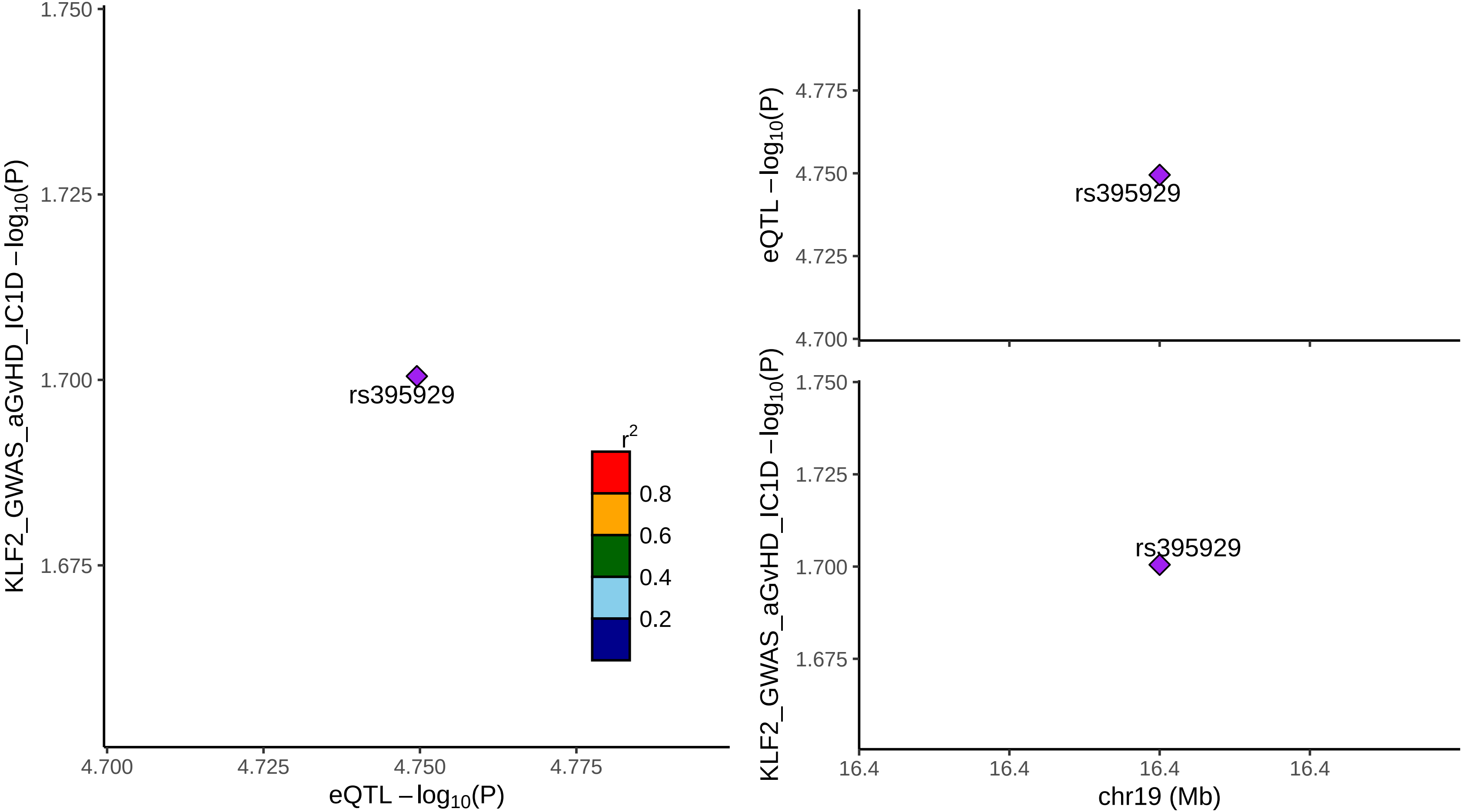
KLF2_GWAS_aGvHD_IC1D −log10(P)
1.750
1.725
1.700
1.675
rs395929
r2
0.8
0.6
0.4
0.2
4.700
4.725
4.750
4.775
eQTL −log10(P)
eQTL −log10(P)
4.775
4.750
4.725
4.700
rs395929
KLF2_GWAS_aGvHD_IC1D −log10(P)
1.750
1.725
1.700
1.675
rs395929
16.4
16.4
16.4
16.4
chr19 (Mb)

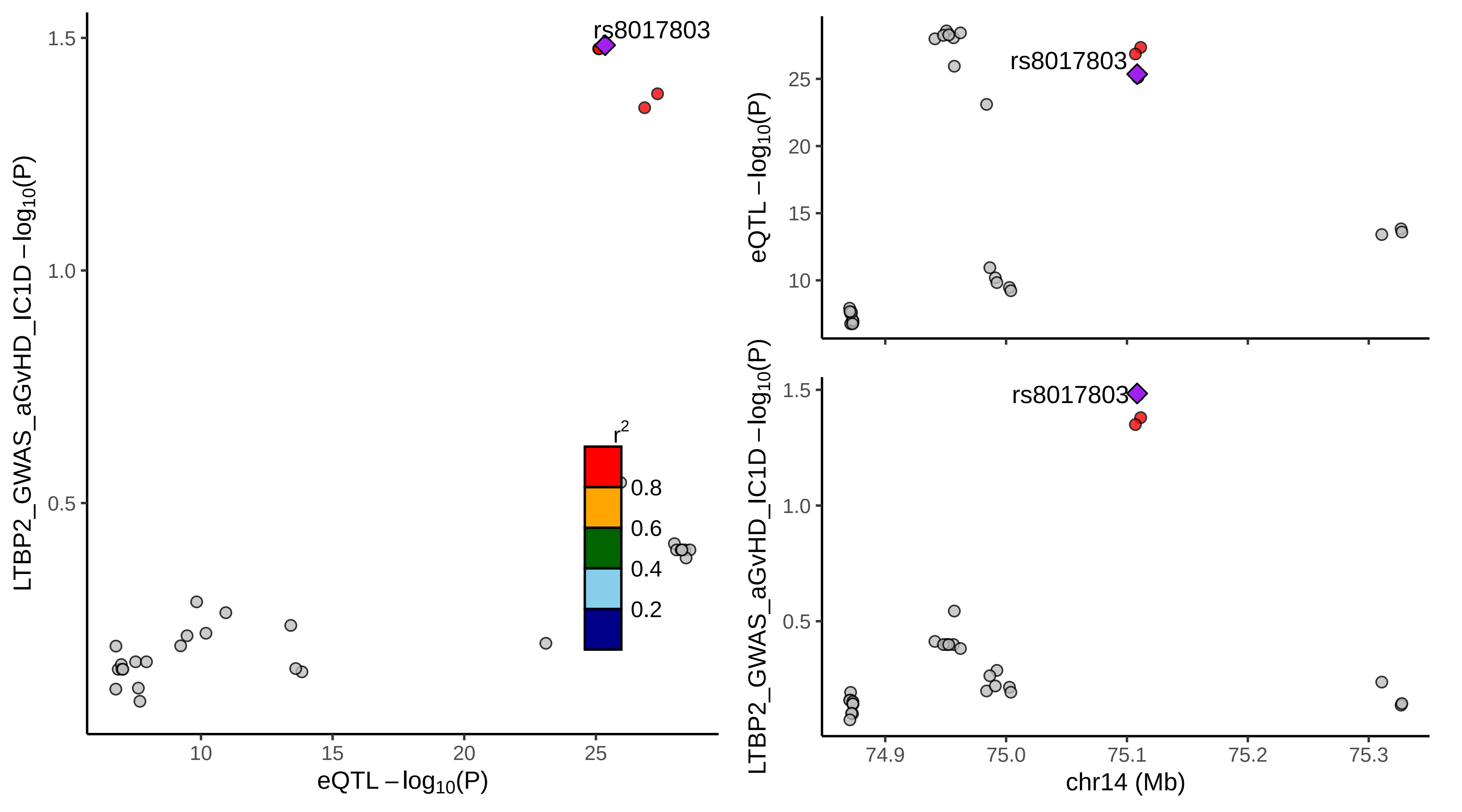

rs8017803
LTBP2_GWAS_aGvHD_IC1D −log10(P)
eQTL −log10(P)
r2
0.8
0.6
0.4
0.2
chr14 (Mb)

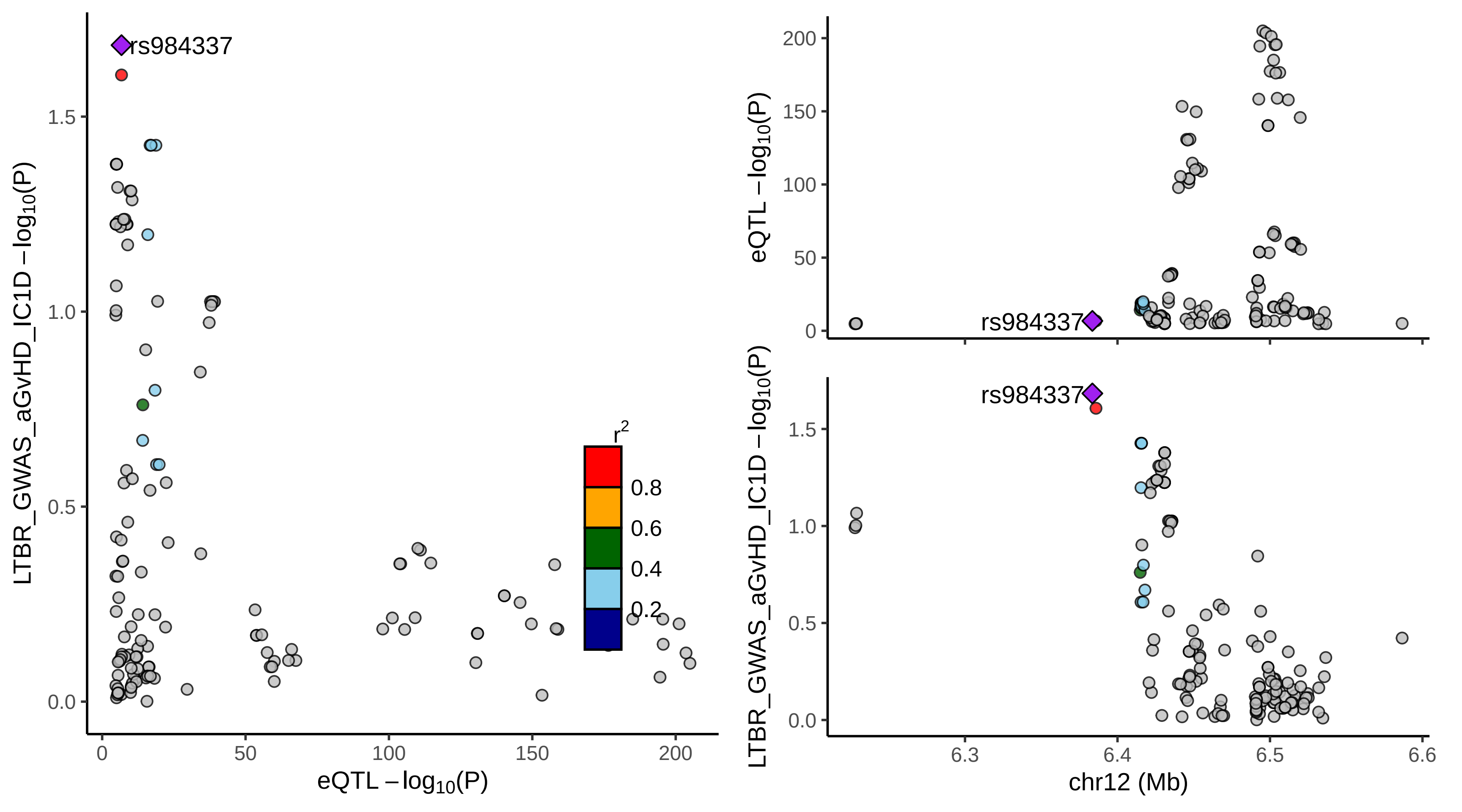

rs984337
LTBR_GWAS_aGvHD_IC1D −log10(P)
eQTL −log10(P)
r2
0.8
0.6
0.4
0.2
chr12 (Mb)

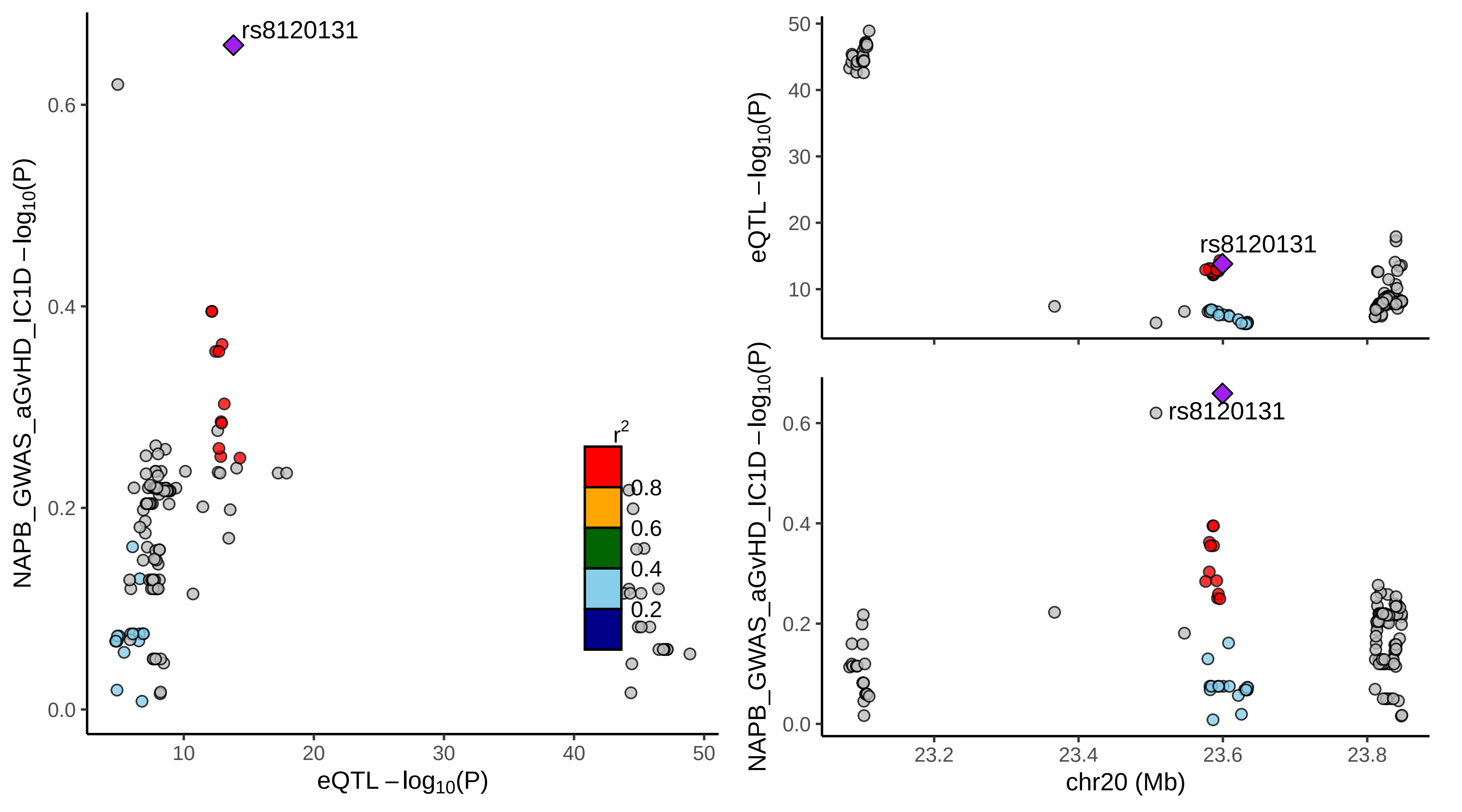

rs8120131
NAPB_GWAS_aGvHD_IC1D −log10(P)
eQTL −log10(P)
0.0
0.2
0.4
0.6
10
20
30
40
50
r2
0.2
0.4
0.6
0.8
eQTL −log10(P)
rs8120131
NAPB_GWAS_aGvHD_IC1D −log10(P)
rs8120131
23.2
23.4
23.6
23.8
chr20 (Mb)

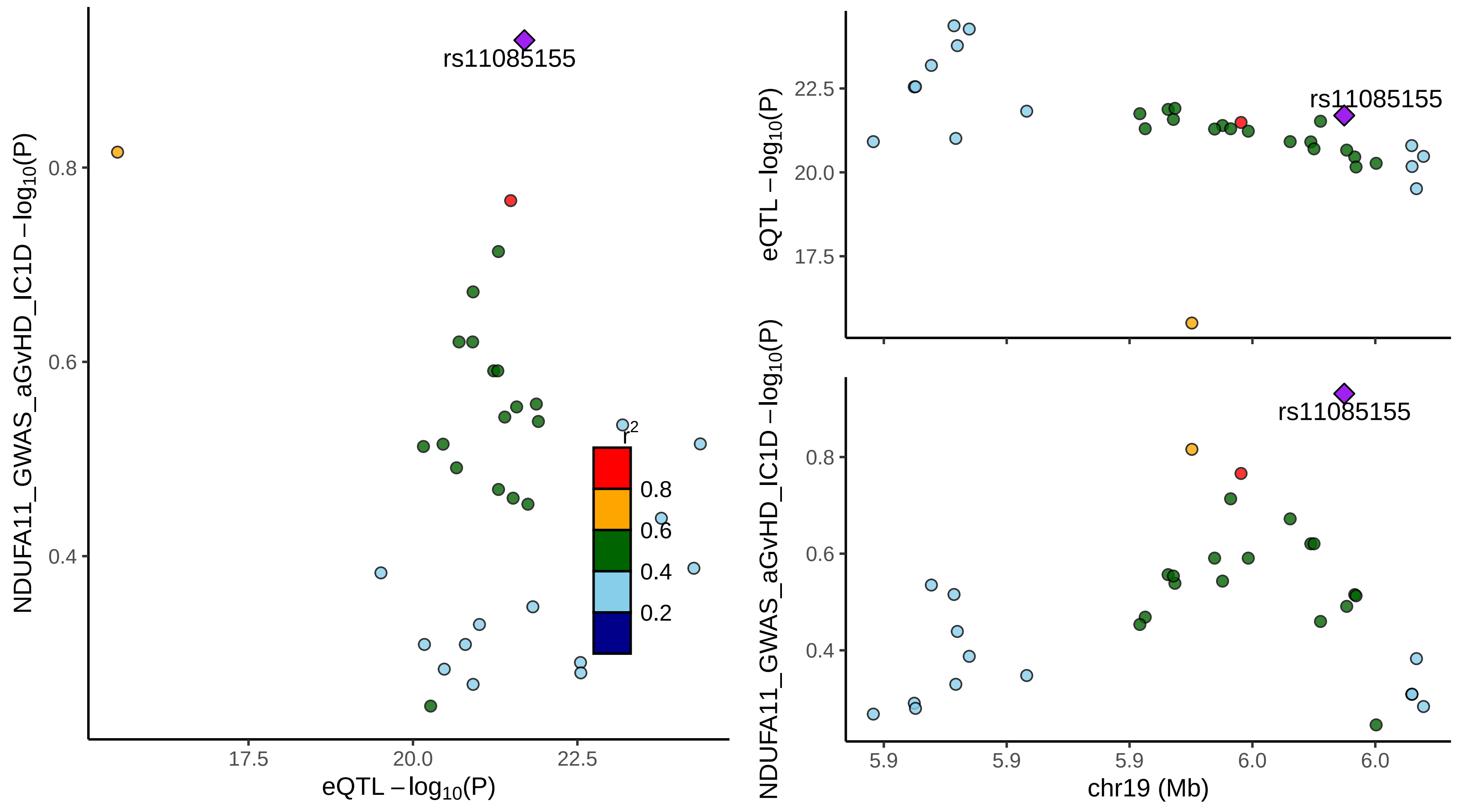

rs11085155
NDUFA11_GWAS_aGvHD_IC1D −log10(P)
0.8
0.6
0.4
17.5
20.0
22.5
eQTL −log10(P)
r2
0.8
0.6
0.4
0.2
eQTL −log10(P)
22.5
20.0
17.5
rs11085155
rs11085155
NDUFA11_GWAS_aGvHD_IC1D −log10(P)
0.8
0.6
0.4
5.9
5.9
5.9
6.0
6.0
chr19 (Mb)

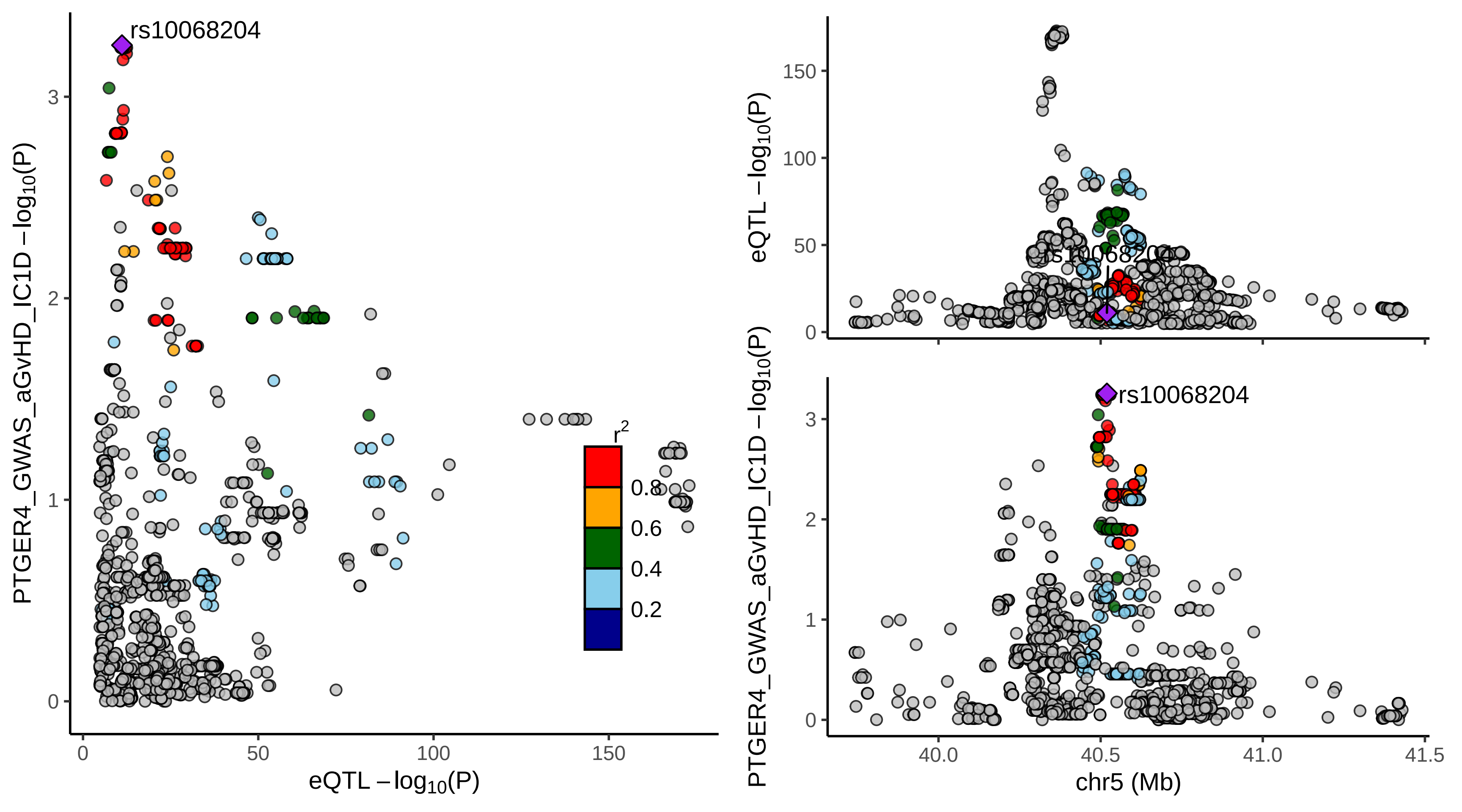

rs10068204
PTGER4_GWAS_aGvHD_IC1D −log10(P)
eQTL −log10(P)
r2
0.8
0.6
0.4
0.2
0
50
100
150
1
2
3
chr5 (Mb)
40.0
40.5
41.0
41.5

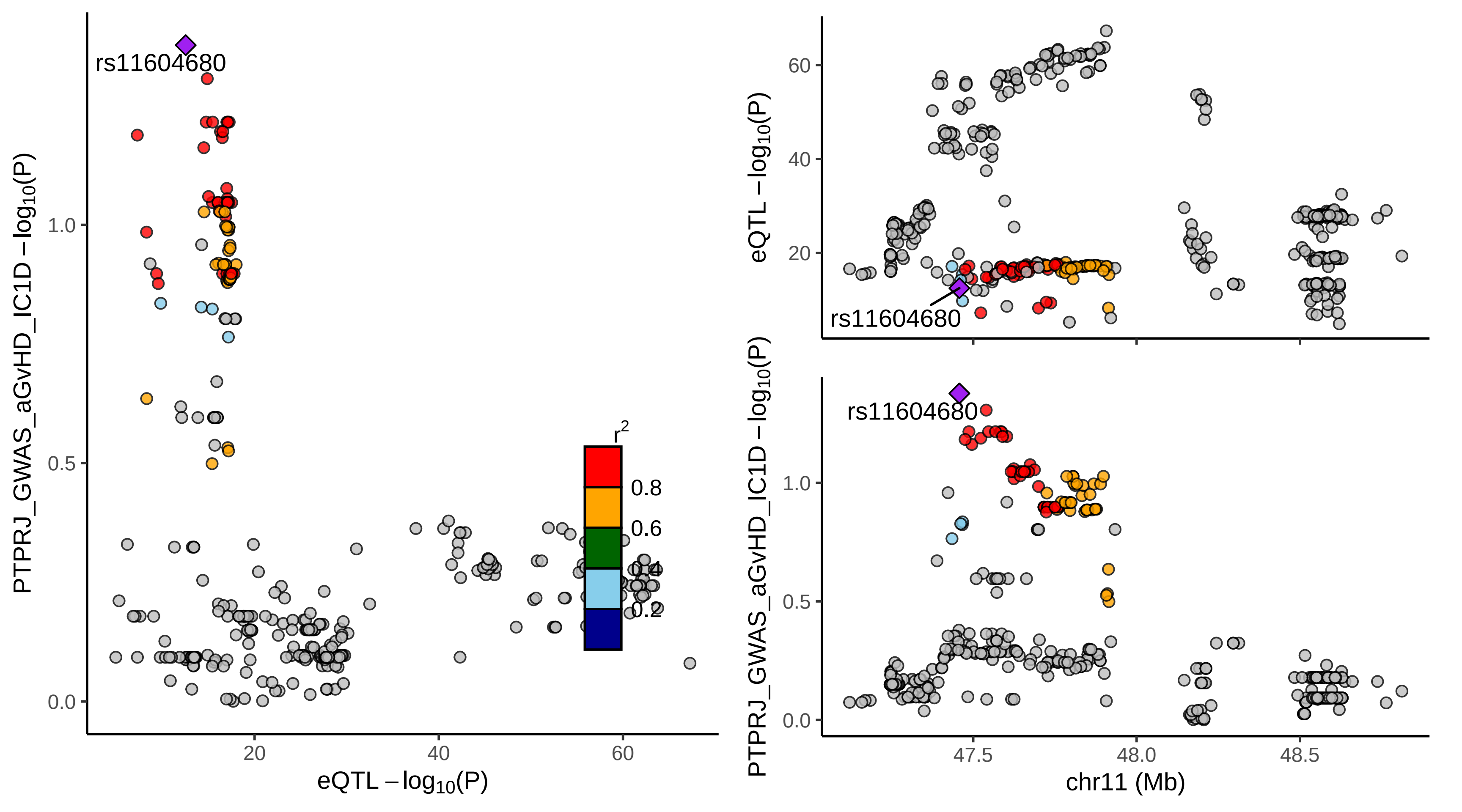

rs11604680
PTPRJ_GWAS_aGvHD_IC1D −log10(P)
eQTL −log10(P)
r2
0.8
0.6
0.4
0.2
1.0
0.5
0.0
20
40
60
chr11 (Mb)
47.5
48.0
48.5

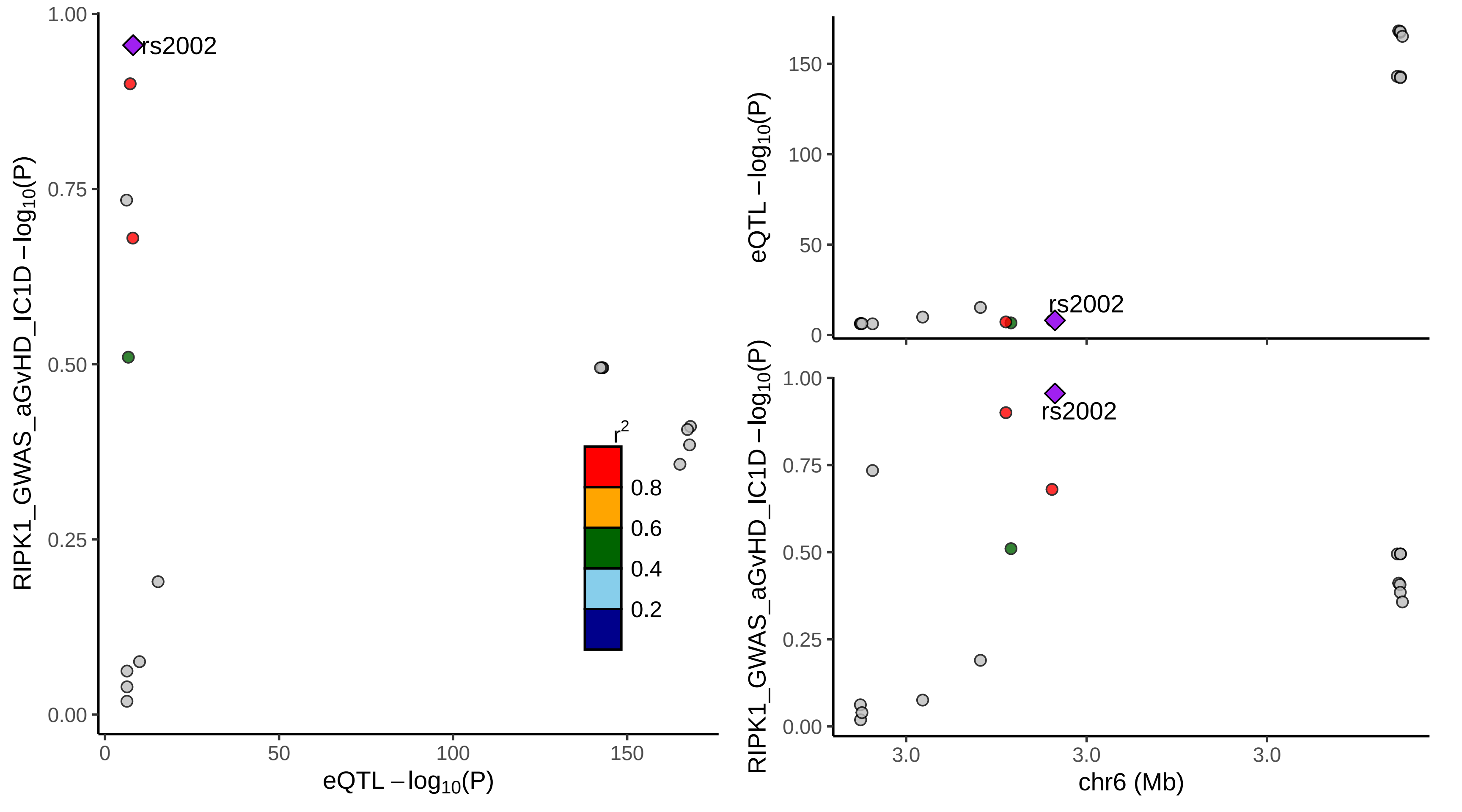

rs2002
RIPK1_GWAS_aGvHD_IC1D −log10(P)
eQTL −log10(P)
r2
0.8
0.6
0.4
0.2
1.00
0.75
0.50
0.25
0.00
0
50
100
150
rs2002
eQTL −log10(P)
rs2002
RIPK1_GWAS_aGvHD_IC1D −log10(P)
3.0
3.0
3.0
chr6 (Mb)

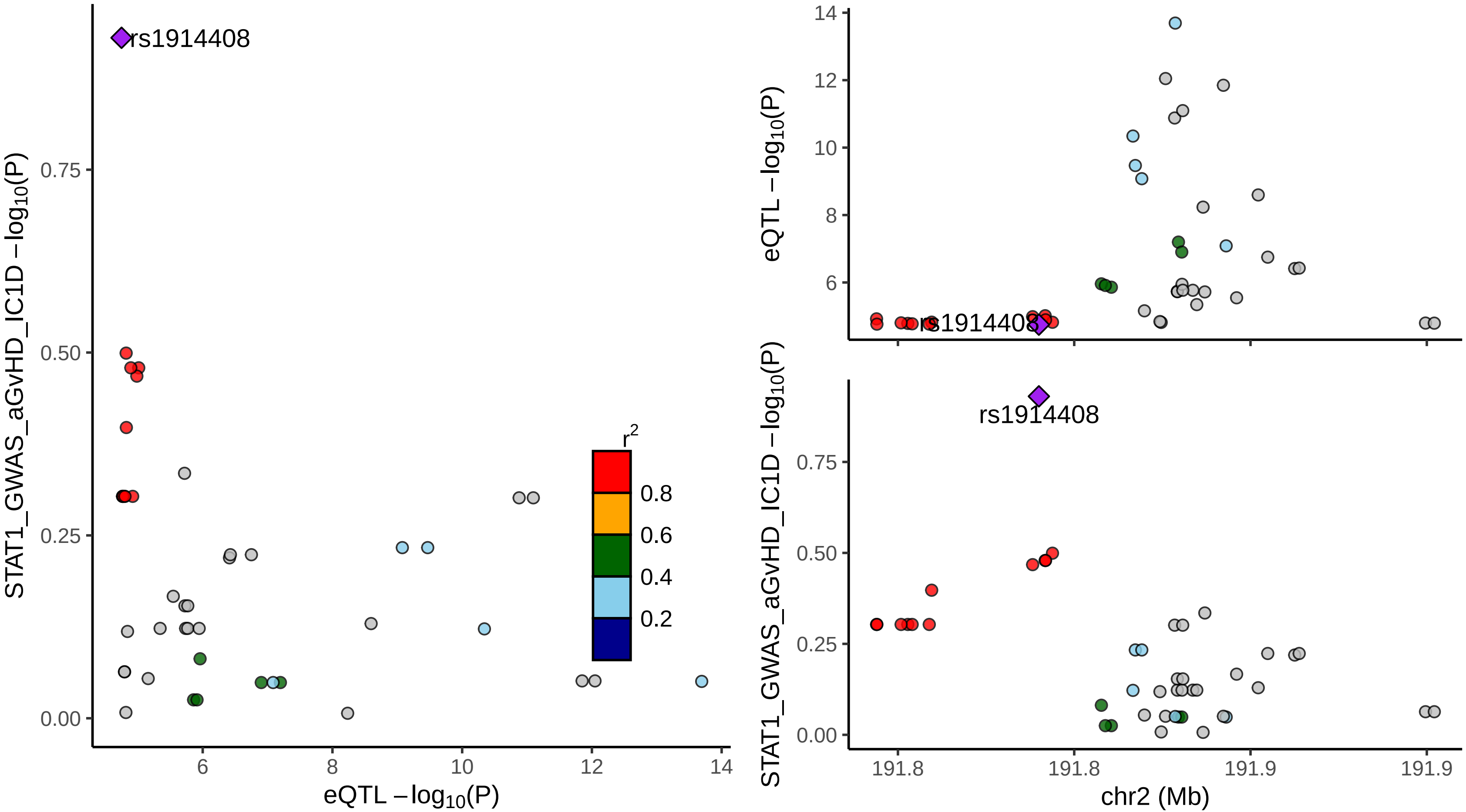

rs1914408
STAT1_GWAS_aGvHD_IC1D $-\log_{10}(P)$
0.00
0.25
0.50
0.75
6
8
10
12
14
eQTL $-\log_{10}(P)$
r^2
0.8
0.6
0.4
0.2
eQTL $-\log_{10}(P)$
rs1914408
rs1914408
191.8
191.9
chr2 (Mb)

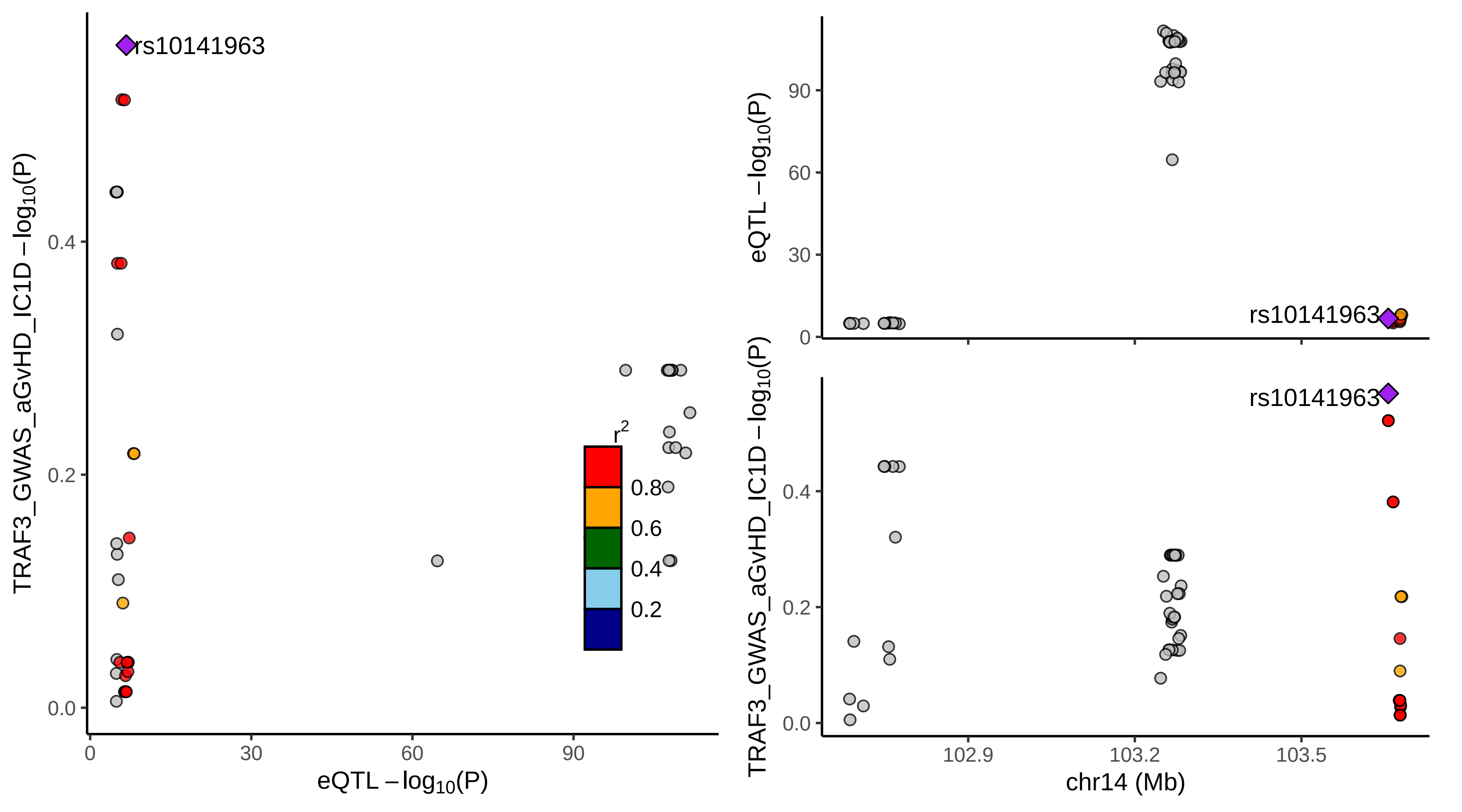

rs10141963
TRAF3_GWAS_aGvHD_IC1D −log10(P)
0.0
0.2
0.4
0
30
60
90
eQTL −log10(P)
r2
0.8
0.6
0.4
0.2
eQTL −log10(P)
0
30
60
90
rs10141963
TRAF3_GWAS_aGvHD_IC1D −log10(P)
0.0
0.2
0.4
rs10141963
102.9
103.2
103.5
chr14 (Mb)

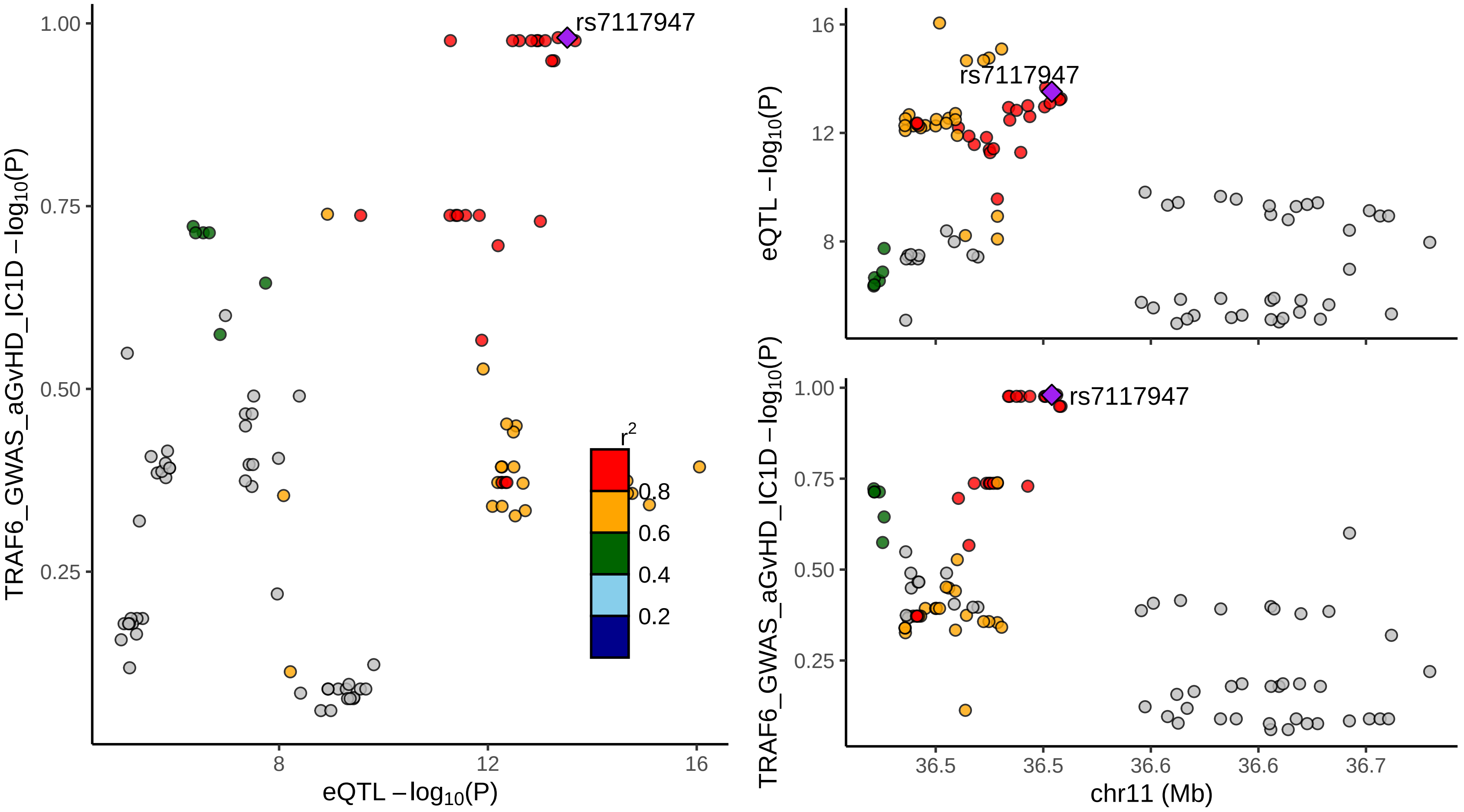

rs7117947
1.00
0.75
0.50
0.25
TRAF6_GWAS_aGvHD_IC1D −log10(P)
8
12
16
eQTL −log10(P)
r2
0.8
0.6
0.4
0.2
rs7117947
16
12
8
eQTL −log10(P)
rs7117947
1.00
0.75
0.50
0.25
TRAF6_GWAS_aGvHD_IC1D −log10(P)
36.5
36.5
36.6
36.6
36.7
chr11 (Mb)

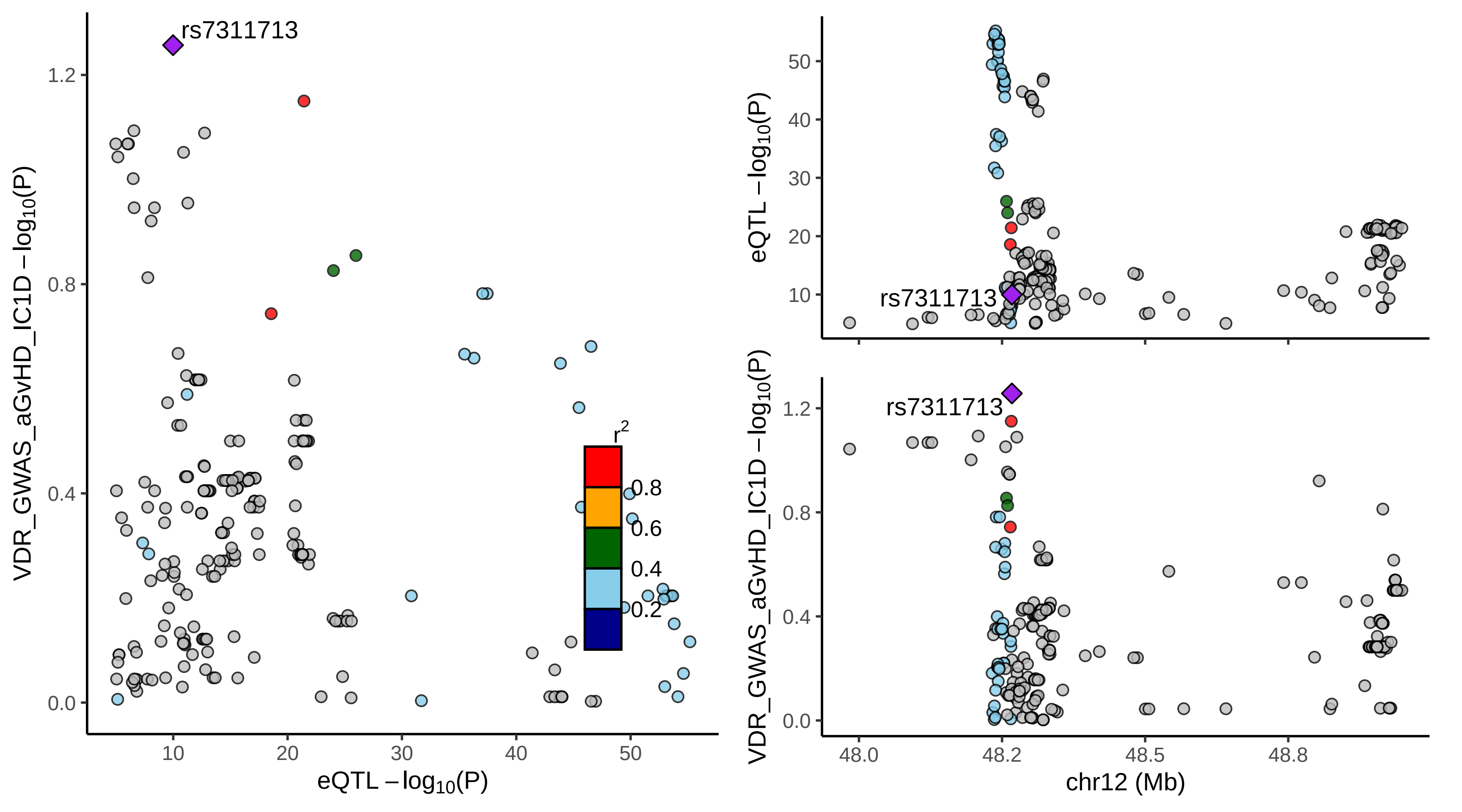

rs7311713
VDR_GWAS_aGvHD_IC1D −log10(P)
eQTL −log10(P)
r2
0.8
0.6
0.4
0.2
chr12 (Mb)
48.0
48.2
48.5
48.8
1.2
0.8
0.4
0.0
10
20
30
40
50

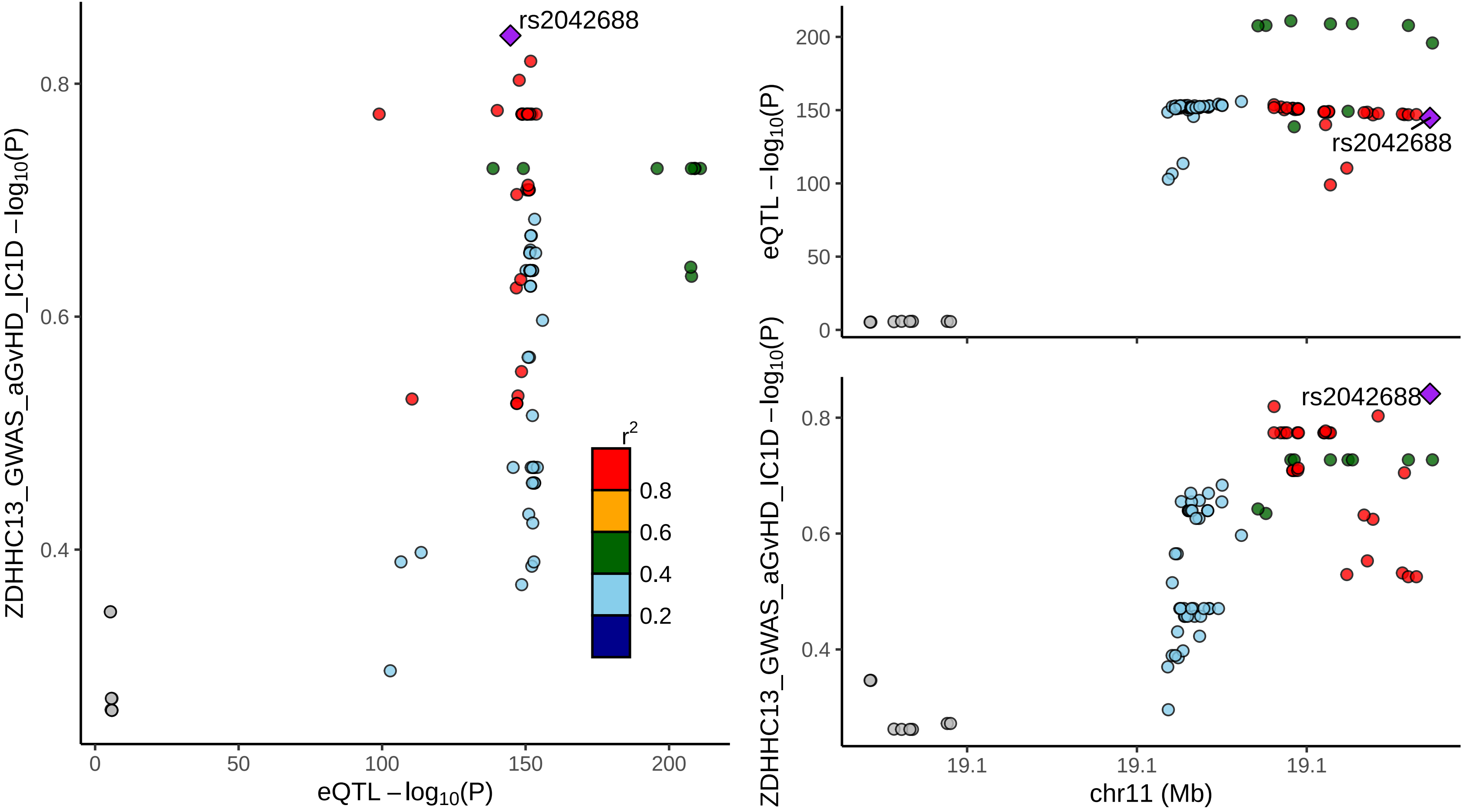

rs2042688
ZDHHC13_GWAS_aGvHD_IC1D −log10(P)
eQTL −log10(P)
r2
0.8
0.6
0.4
0.2
0
50
100
150
200
0.4
0.6
0.8
rs2042688
eQTL −log10(P)
rs2042688
ZDHHC13_GWAS_aGvHD_IC1D −log10(P)
19.1
19.1
19.1
chr11 (Mb)

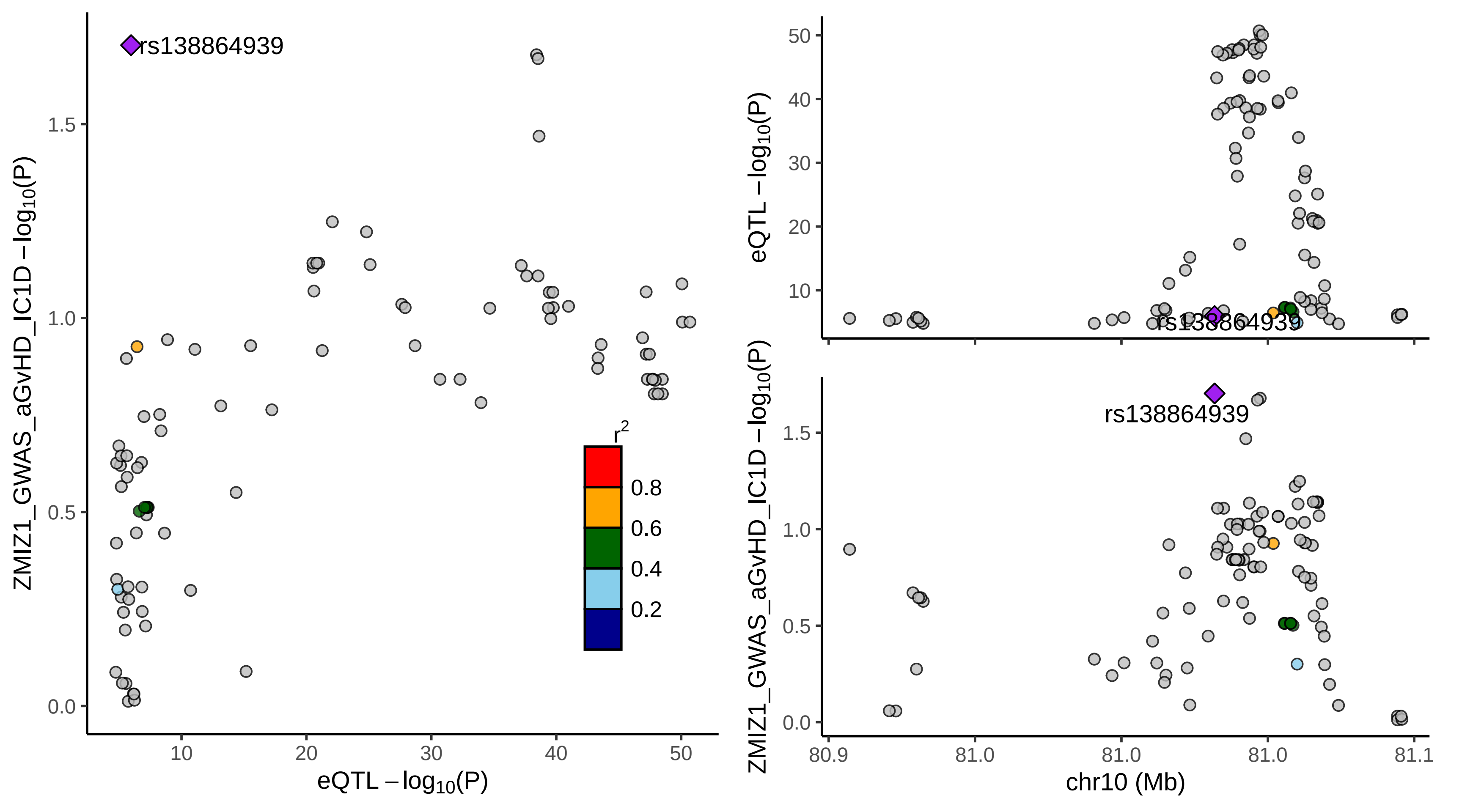

rs138864939
ZMIZ1_GWAS_aGvHD_IC1D −log10(P)
eQTL −log10(P)
r2
0.8
0.6
0.4
0.2
rs138864939
eQTL −log10(P)
rs138864939
ZMIZ1_GWAS_aGvHD_IC1D −log10(P)
chr10 (Mb)

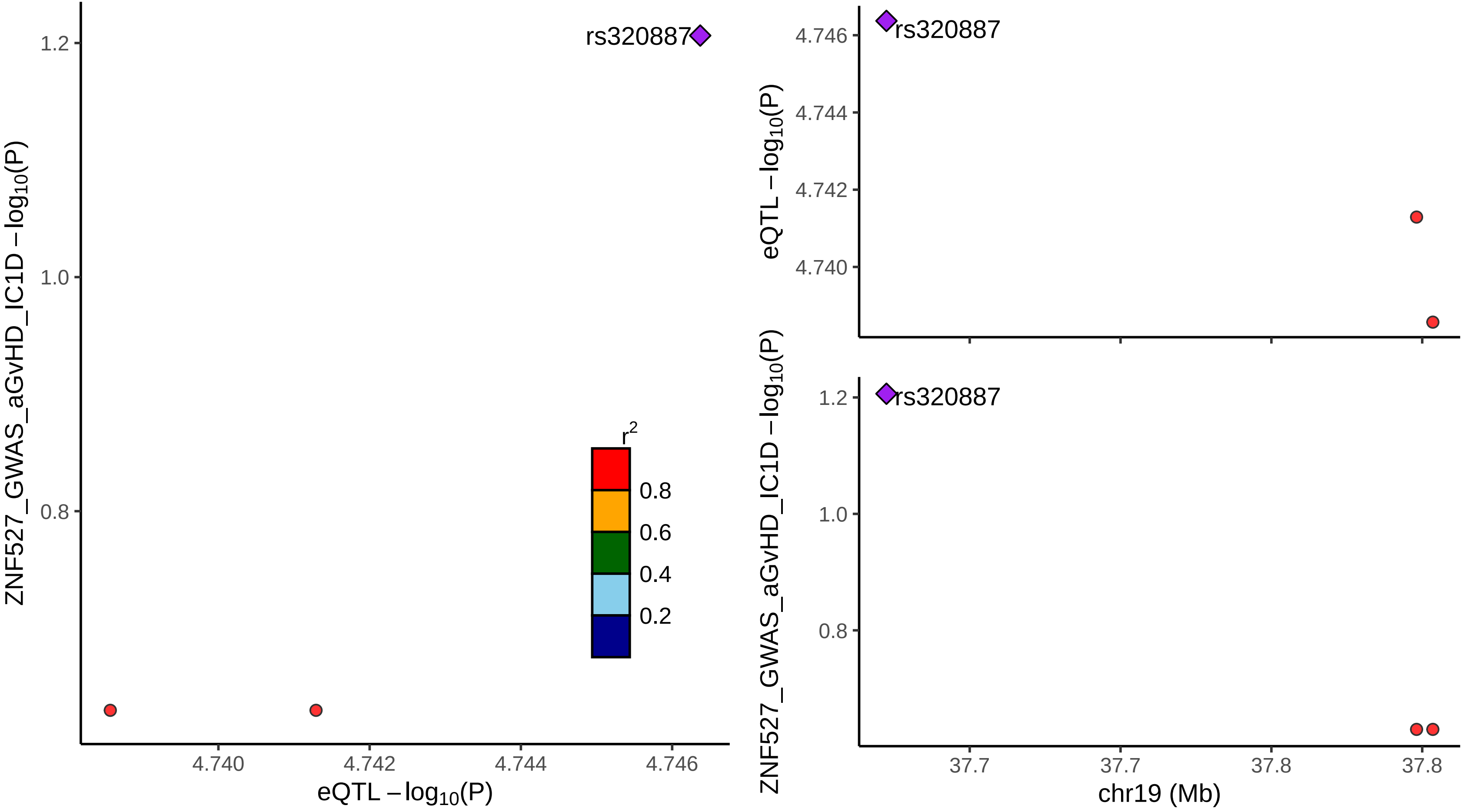

rs320887
1.2
1.0
0.8
4.740
4.742
4.744
4.746
eQTL −log10(P)
ZNF527_GWAS_aGvHD_IC1D −log10(P)
r2
0.8
0.6
0.4
0.2
4.746
4.744
4.742
4.740
eQTL −log10(P)
37.7
37.7
37.8
37.8
chr19 (Mb)
