# Supplementary material for: Meta-Analysis of Genome-Wide Association and Gene Expression Studies Implicates Donor T Cell Function and Cytokine Pathways in Acute GvHD
Source: Front Immunol. 2020 Feb 3;11:19. doi: 10.3389/fimmu.2020.00019 (PMC7008714; doi:10.3389/fimmu.2020.00019)
Supplement: Supplementary file 3 [file Data_Sheet_3.pdf]

LocusCompare plots of Spanish cohort 1

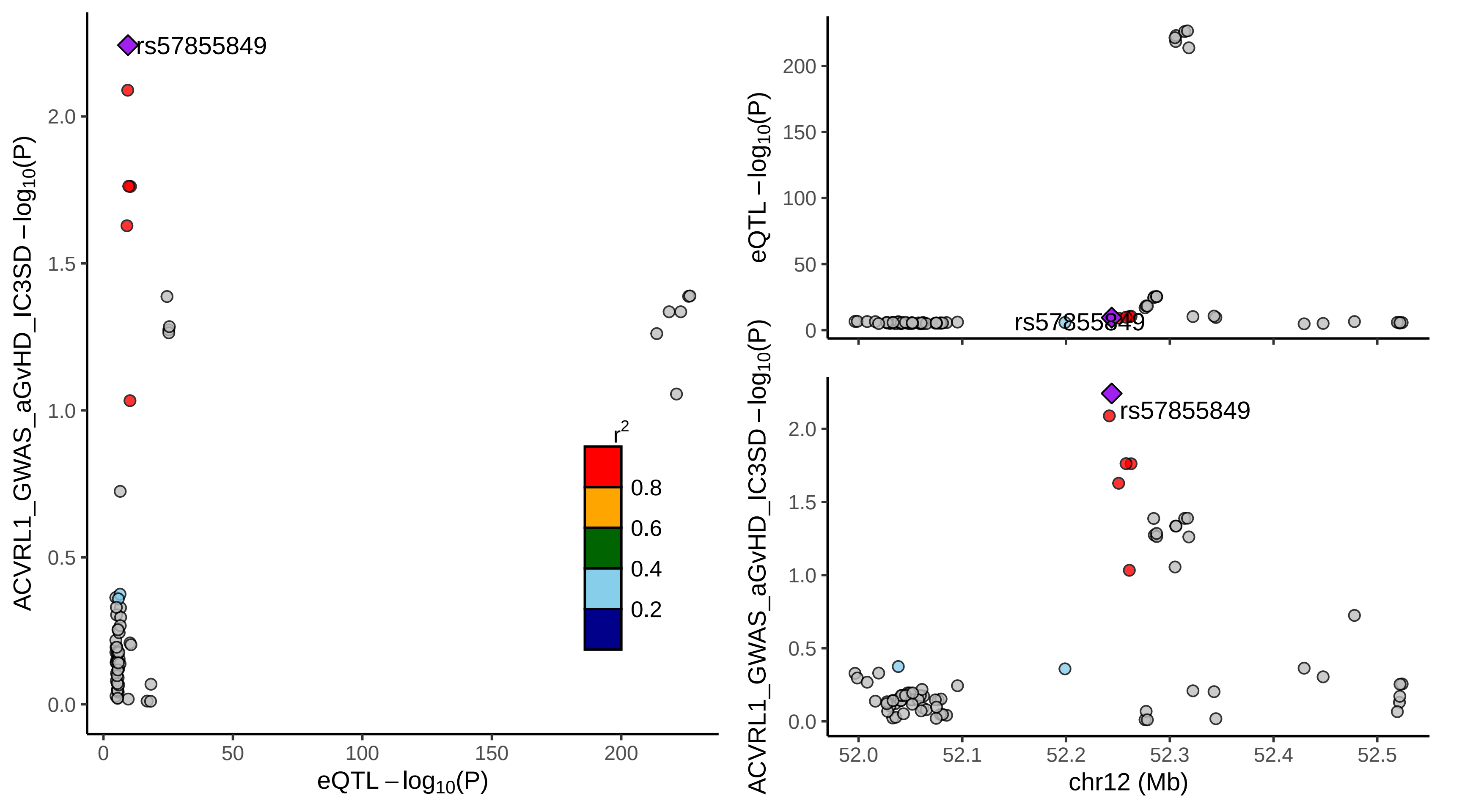

rs57855849
ACVRL1_GWAS_aGvHD_IC3SD −log10(P)
eQTL −log10(P)
r2
0.8
0.6
0.4
0.2
chr12 (Mb)

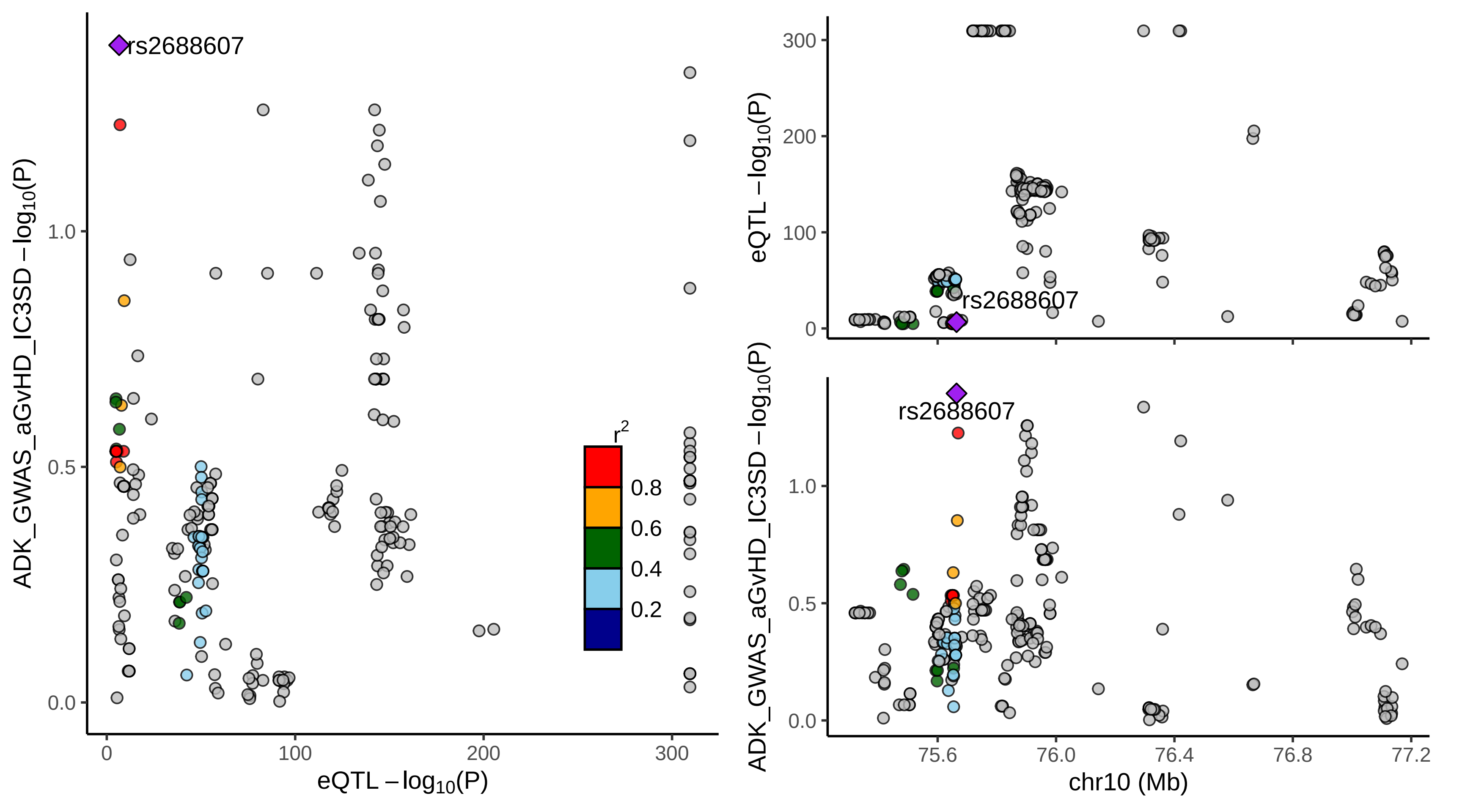

rs2688607
ADK_GWAS_aGvHD_IC3SD −log10(P)
1.0
0.5
0.0
0
100
200
300
eQTL −log10(P)
r2
0.8
0.6
0.4
0.2
eQTL −log10(P)
300
200
100
0
rs2688607
ADK_GWAS_aGvHD_IC3SD −log10(P)
rs2688607
1.0
0.5
0.0
75.6
76.0
76.4
76.8
77.2
chr10 (Mb)

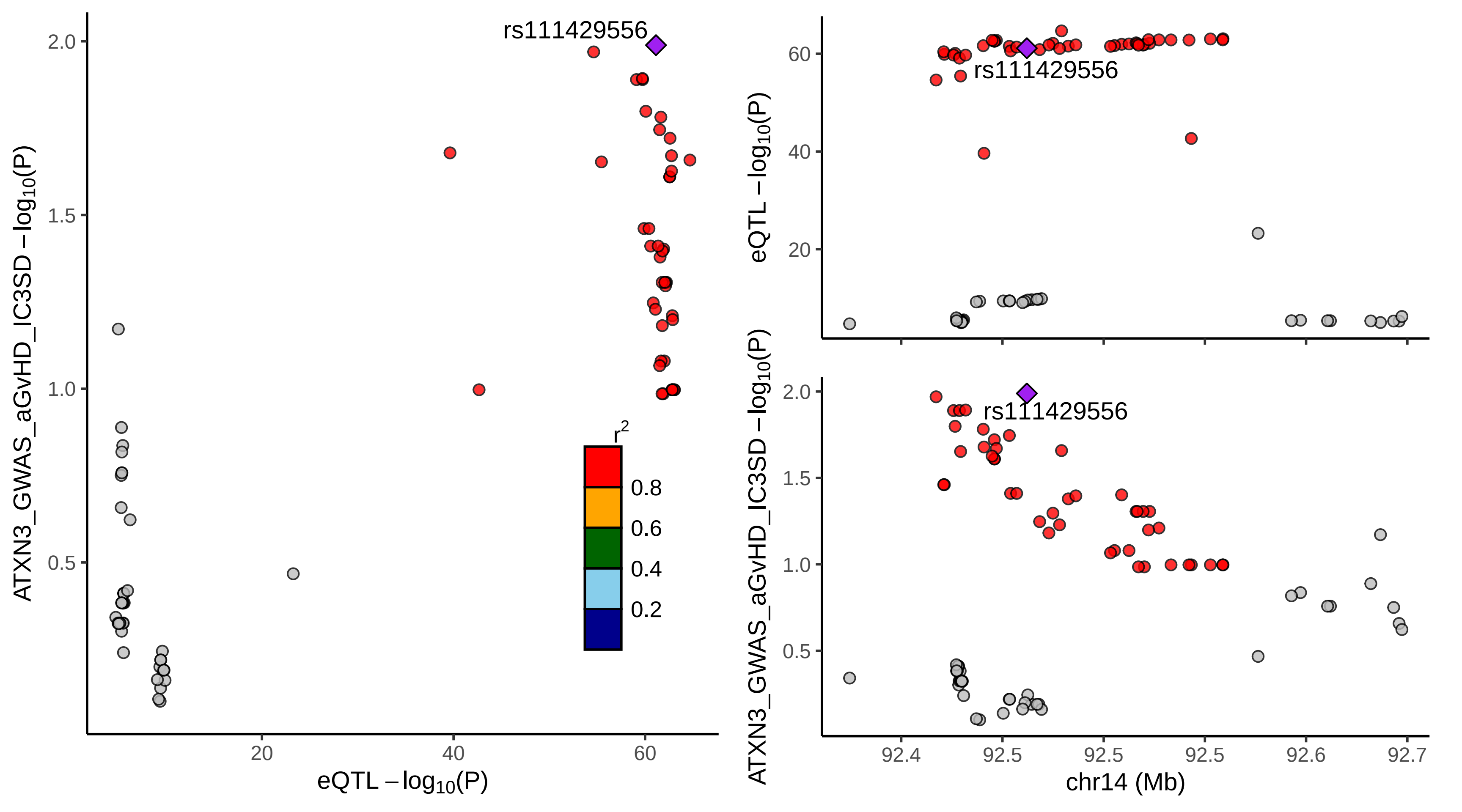

rs111429556
ATXN3_GWAS_aGvHD_IC3SD −log10(P)
eQTL −log10(P)
r2
0.8
0.6
0.4
0.2
2.0
1.5
1.0
0.5
20
40
60
chr14 (Mb)
92.4
92.5
92.6
92.7

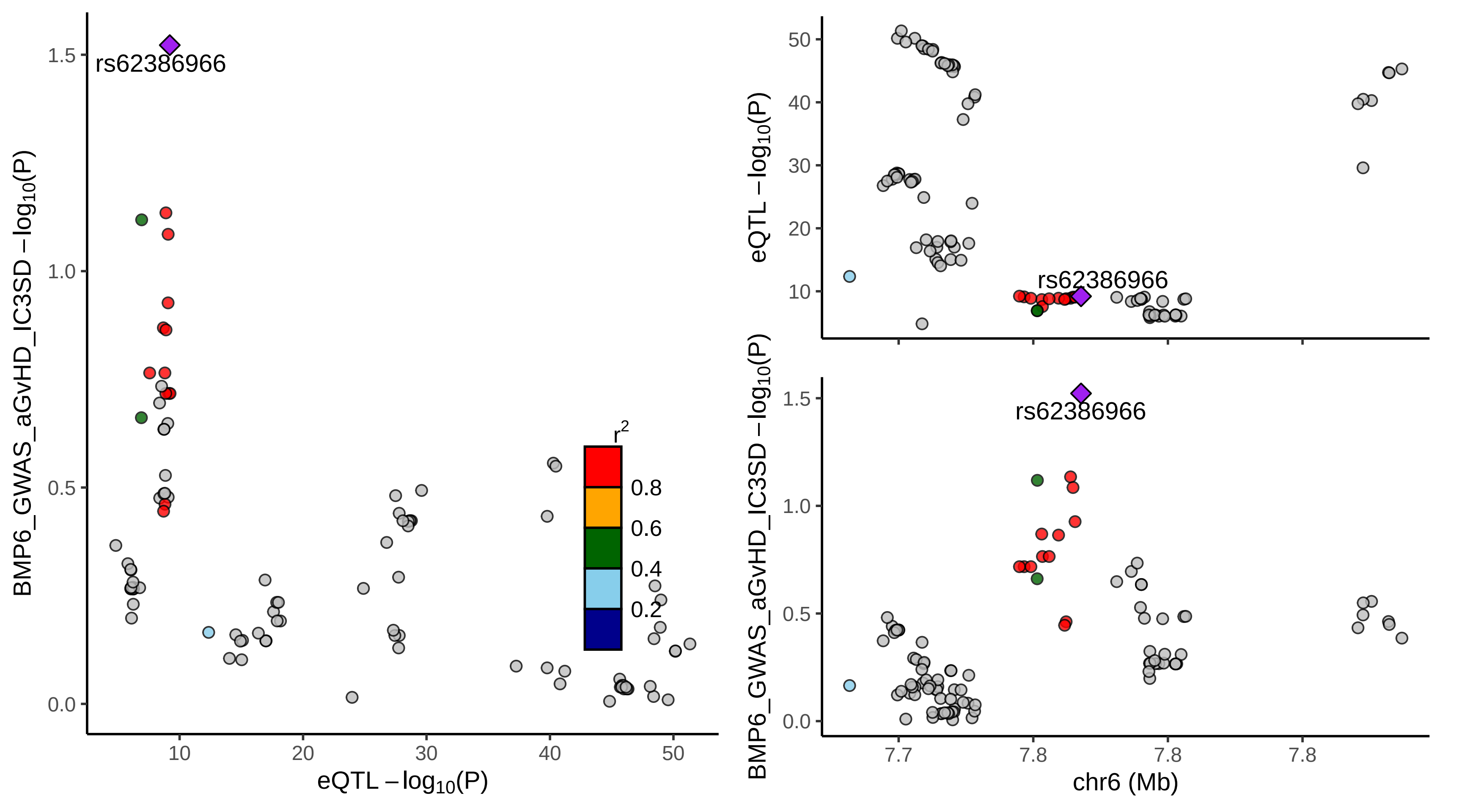
rs62386966
BMP6_GWAS_aGvHD_IC3SD −log10(P)
eQTL −log10(P)
r2
0.8
0.6
0.4
0.2
1.5
1.0
0.5
0.0
10
20
30
40
50
eQTL −log10(P)
chr6 (Mb)
7.7
7.8
7.8
7.8

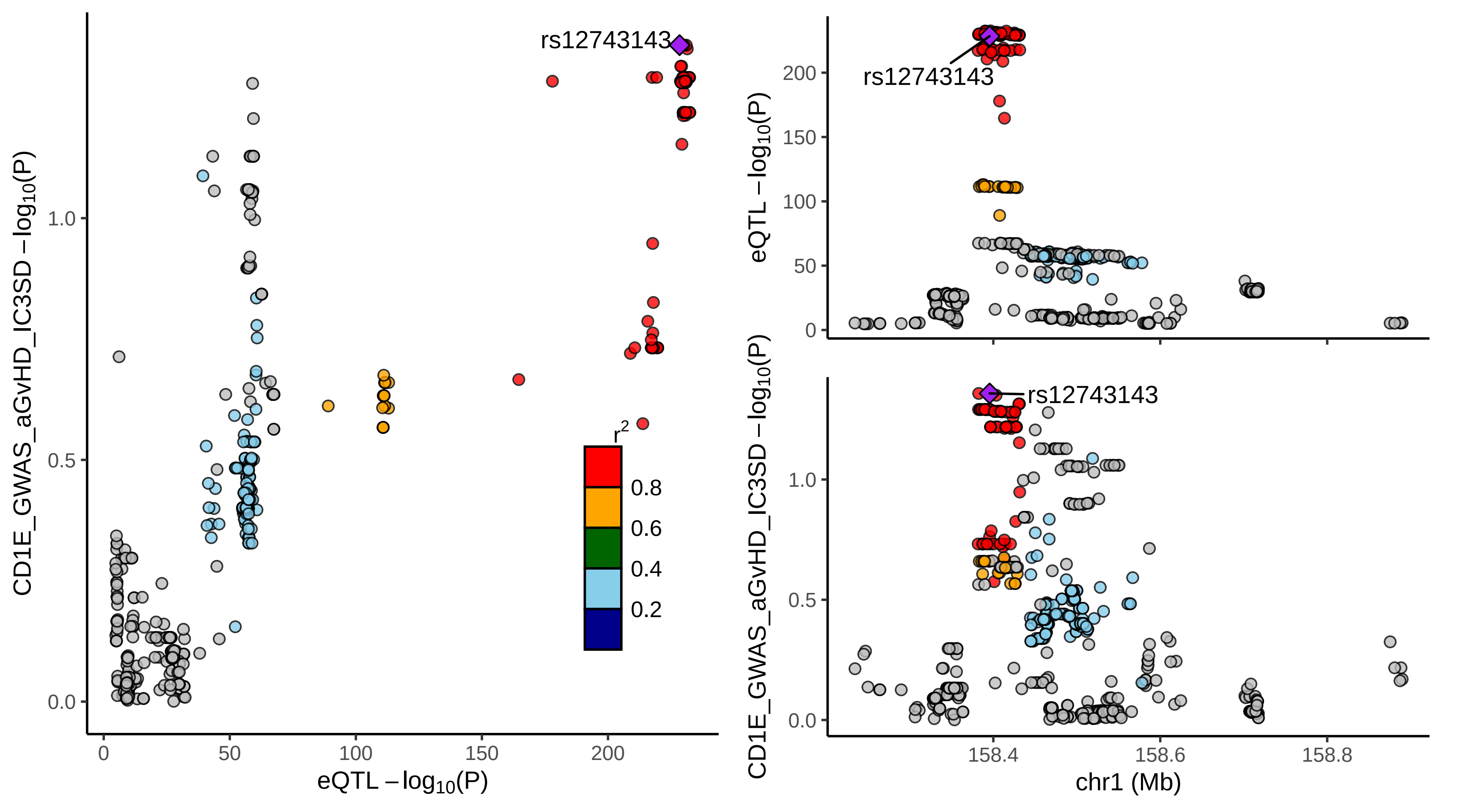
rs12743143
CD1E_GWAS_aGvHD_IC3SD −log10(P)
1.0
0.5
0.0
0
50
100
150
200
eQTL −log10(P)
r2
0.8
0.6
0.4
0.2
rs12743143
eQTL −log10(P)
200
150
100
50
0
rs12743143
CD1E_GWAS_aGvHD_IC3SD −log10(P)
1.0
0.5
0.0
158.4
158.6
158.8
chr1 (Mb)

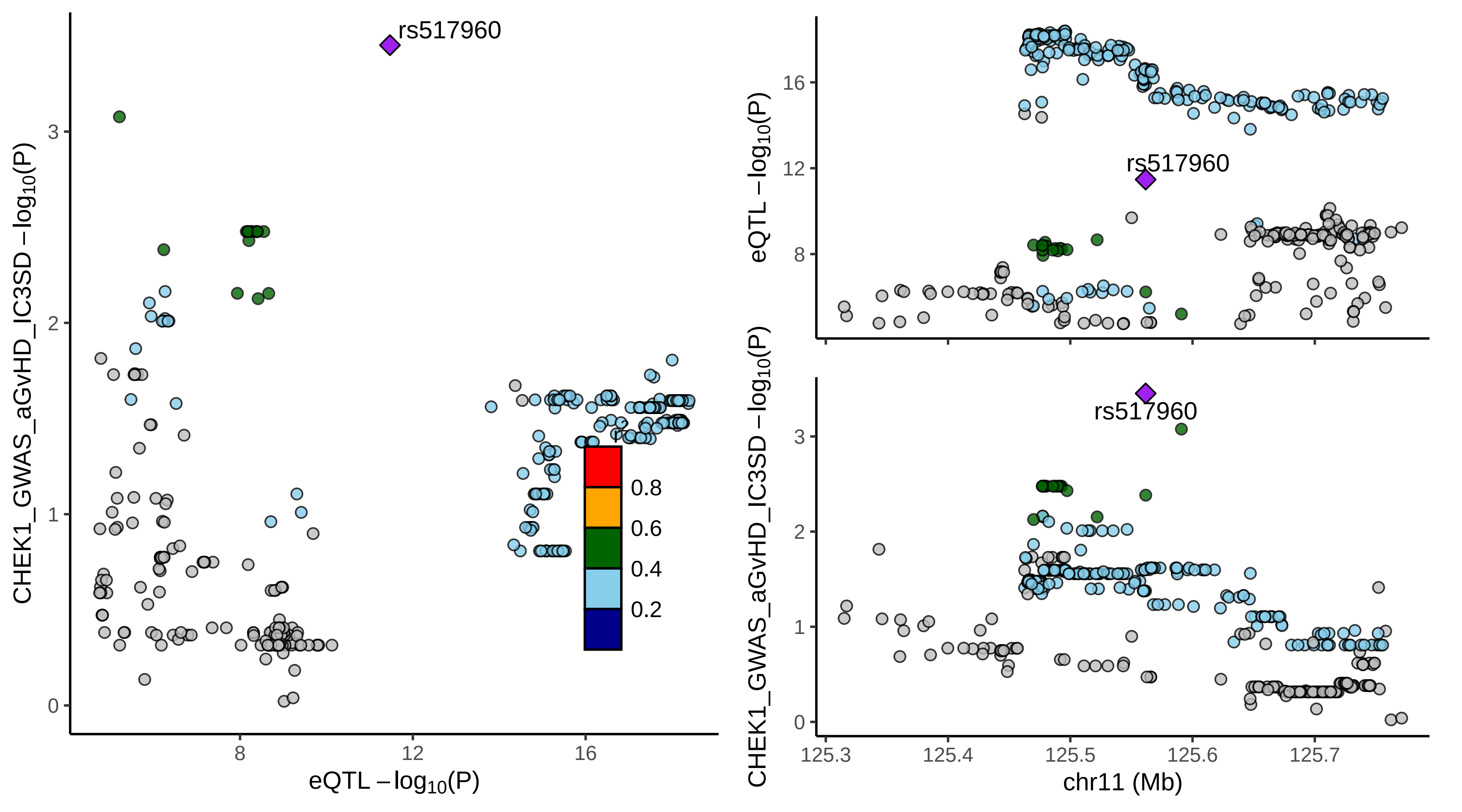
rs517960
CHEK1_GWAS_aGvHD_IC3SD −log10(P)
eQTL −log10(P)
r²
0.8
0.6
0.4
0.2
0
1
2
3
8
12
16
rs517960
eQTL −log10(P)
CHEK1_GWAS_aGvHD_IC3SD −log10(P)
125.3
125.4
125.5
125.6
125.7
chr11 (Mb)

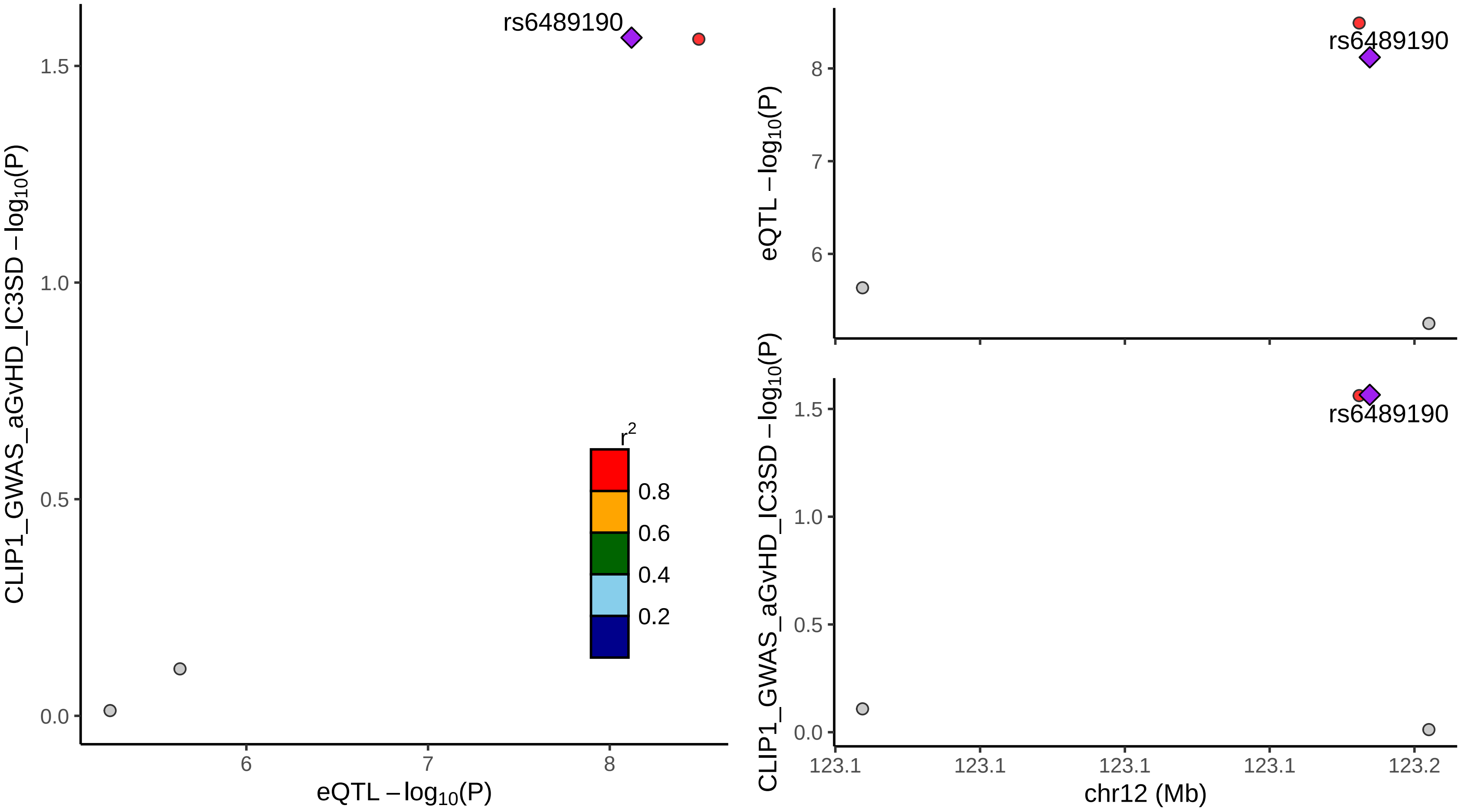

rs6489190
1.5
1.0
0.5
0.0
CLIP1_GWAS_aGvHD_IC3SD −log10(P)
r2
0.8
0.6
0.4
0.2
6
7
8
eQTL −log10(P)
8
7
6
eQTL −log10(P)
rs6489190
1.5
1.0
0.5
0.0
CLIP1_GWAS_aGvHD_IC3SD −log10(P)
rs6489190
123.1
123.1
123.1
123.1
123.2
chr12 (Mb)

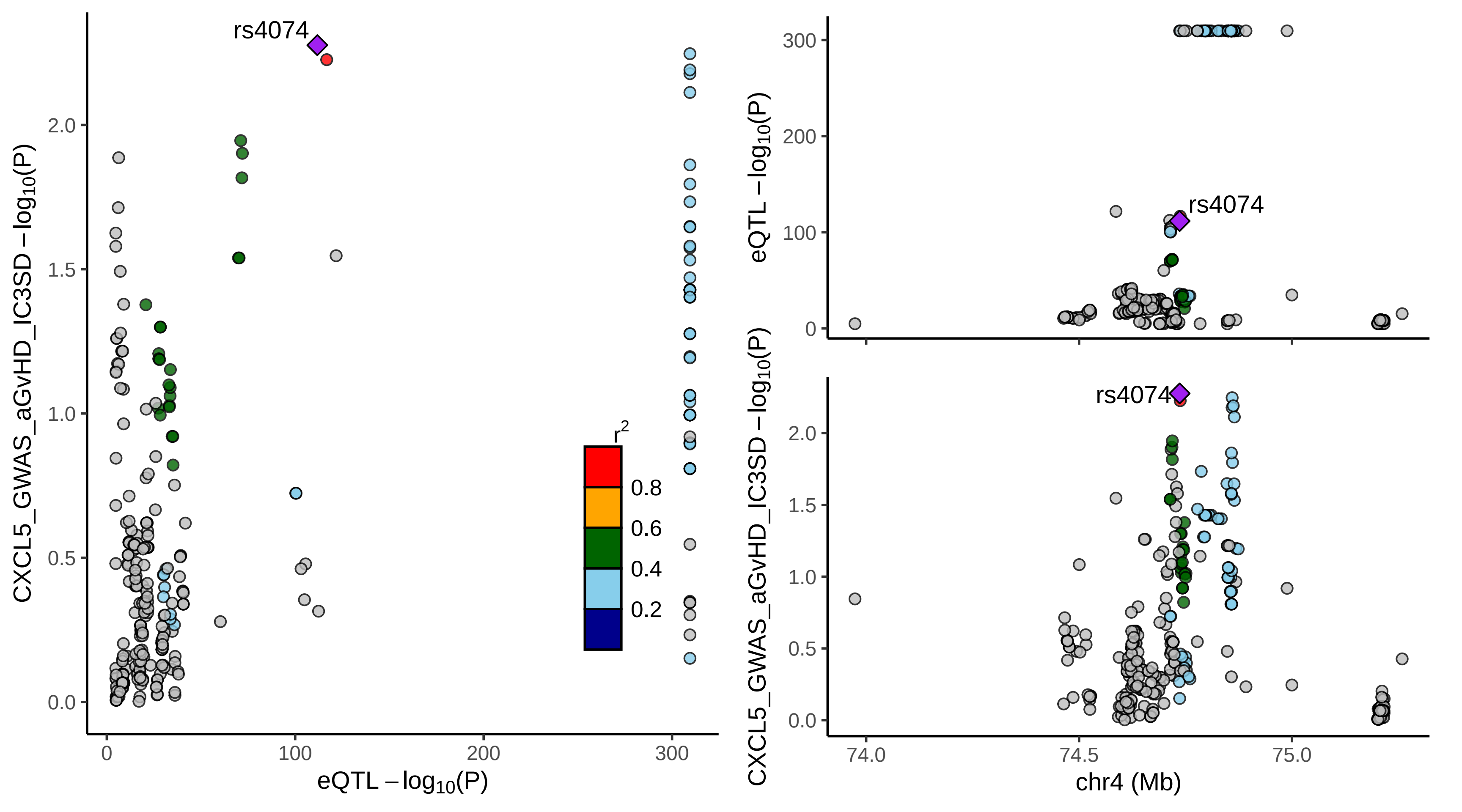
rs4074
CXCL5_GWAS_aGvHD_IC3SD −log10(P)
eQTL −log10(P)
r2
0.8
0.6
0.4
0.2
eQTL −log10(P)
rs4074
CXCL5_GWAS_aGvHD_IC3SD −log10(P)
rs4074
chr4 (Mb)

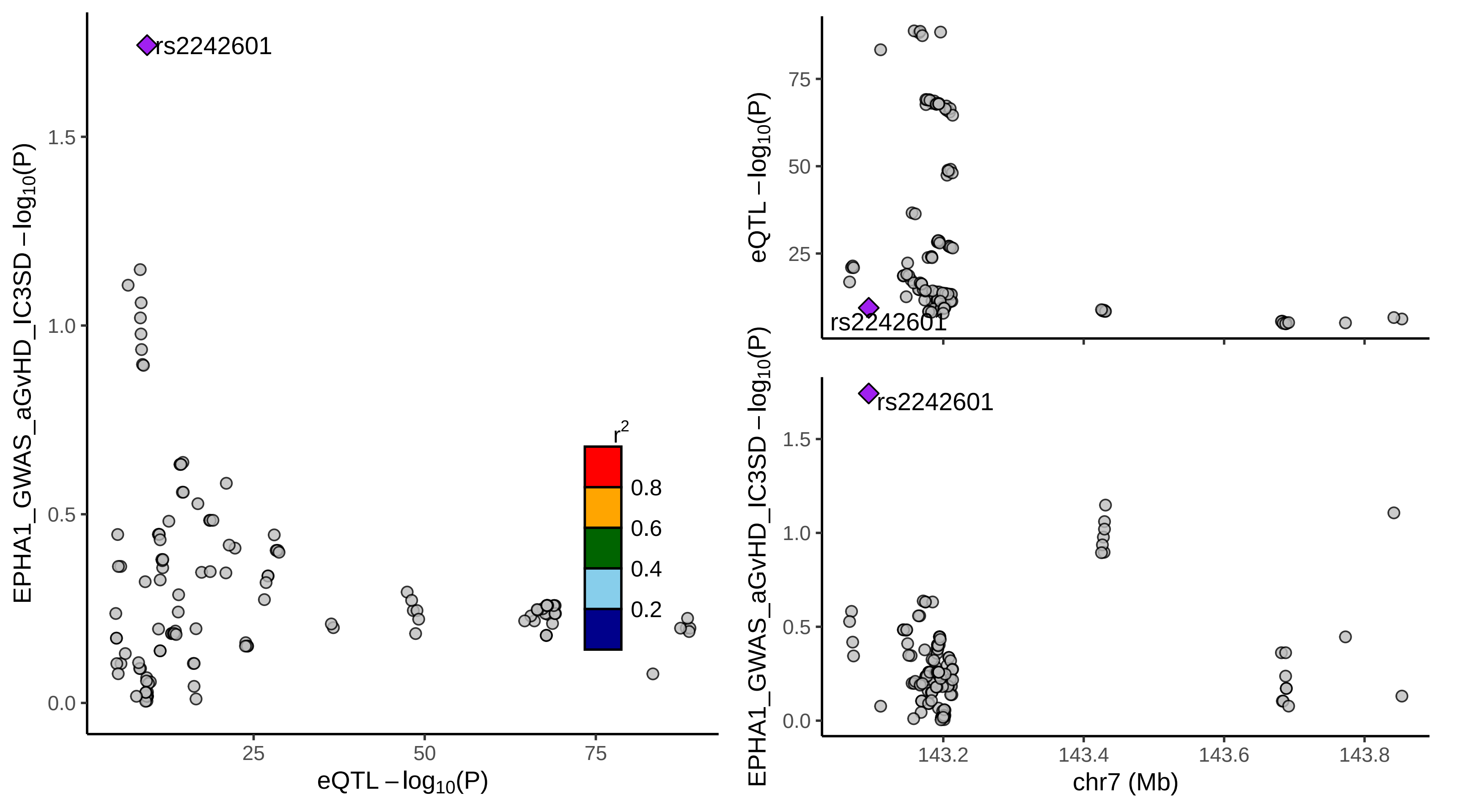

rs2242601
EPHA1_GWAS_aGvHD_IC3SD −log10(P)
eQTL −log10(P)
r2
0.8
0.6
0.4
0.2
chr7 (Mb)
0.0
0.5
1.0
1.5
25
50
75
143.2
143.4
143.6
143.8

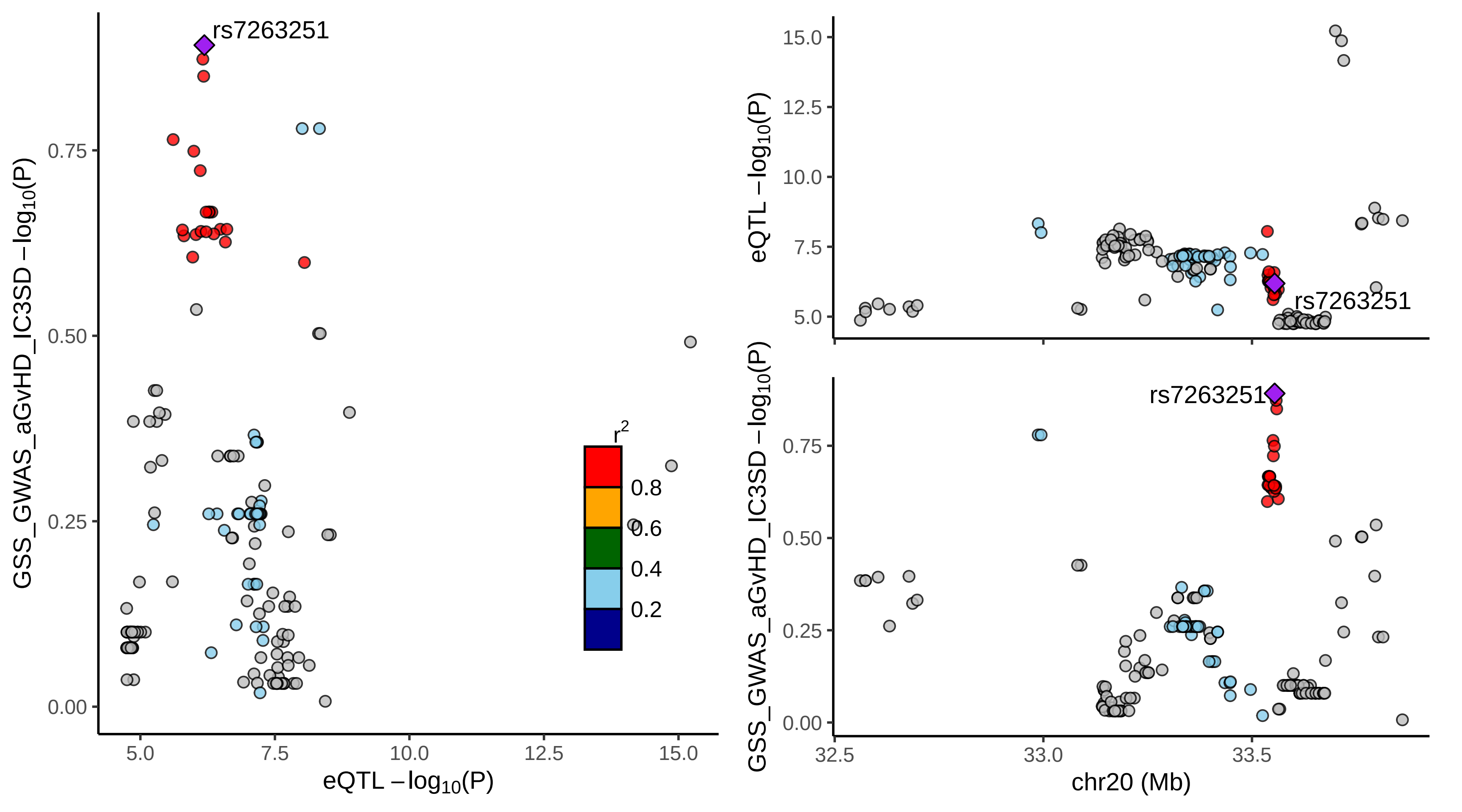
rs7263251
GSS_GWAS_aGvHD_IC3SD −log10(P)
eQTL −log10(P)
0.00
0.25
0.50
0.75
5.0
7.5
10.0
12.5
15.0
r2
0.2
0.4
0.6
0.8
32.5
33.0
33.5
chr20 (Mb)

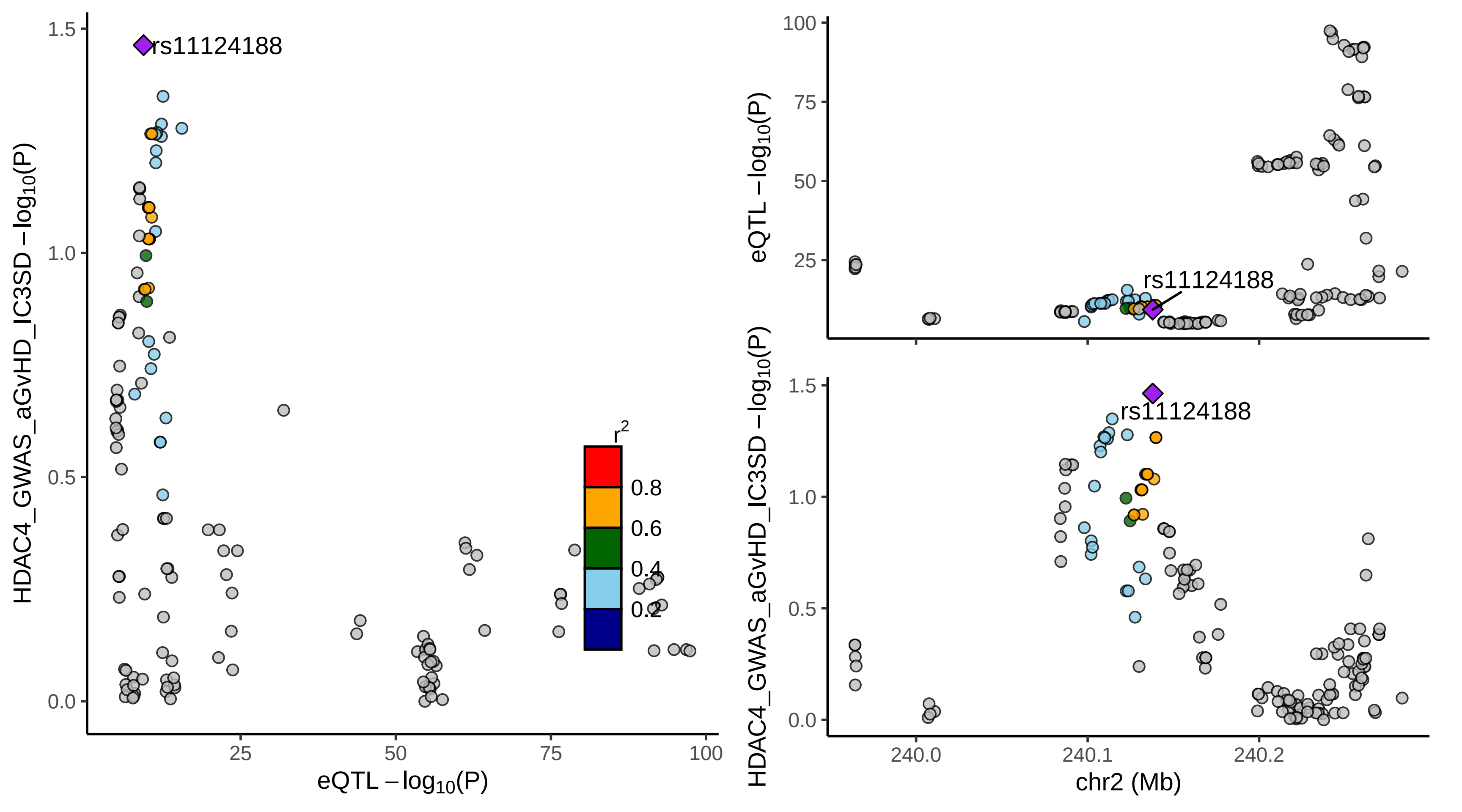

rs11124188
HDAC4_GWAS_aGvHD_IC3SD −log10(P)
eQTL −log10(P)
r2
0.8
0.6
0.4
0.2
chr2 (Mb)

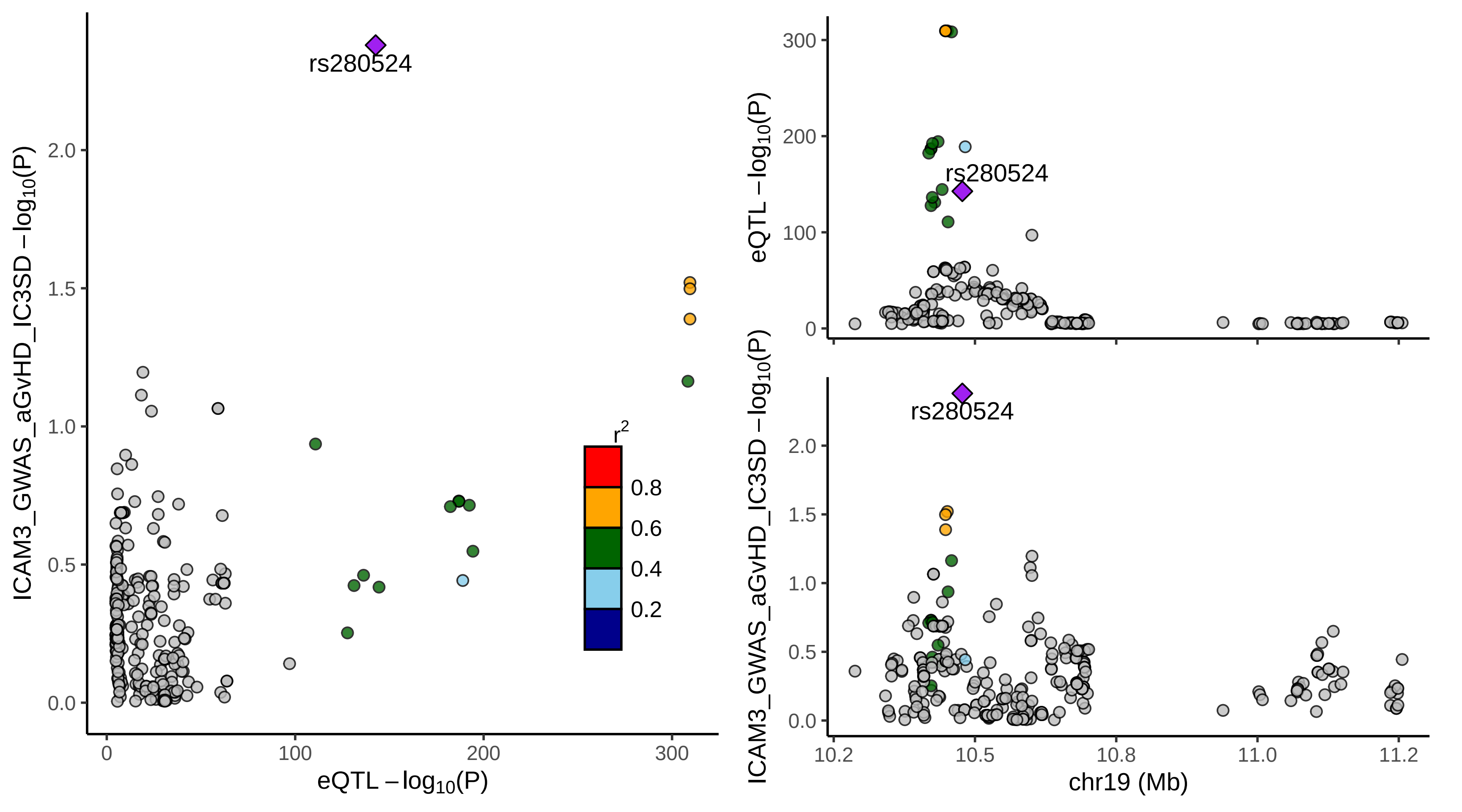

rs280524
ICAM3_GWAS_aGvHD_IC3SD −log10(P)
eQTL −log10(P)
r2
0.8
0.6
0.4
0.2
chr19 (Mb)

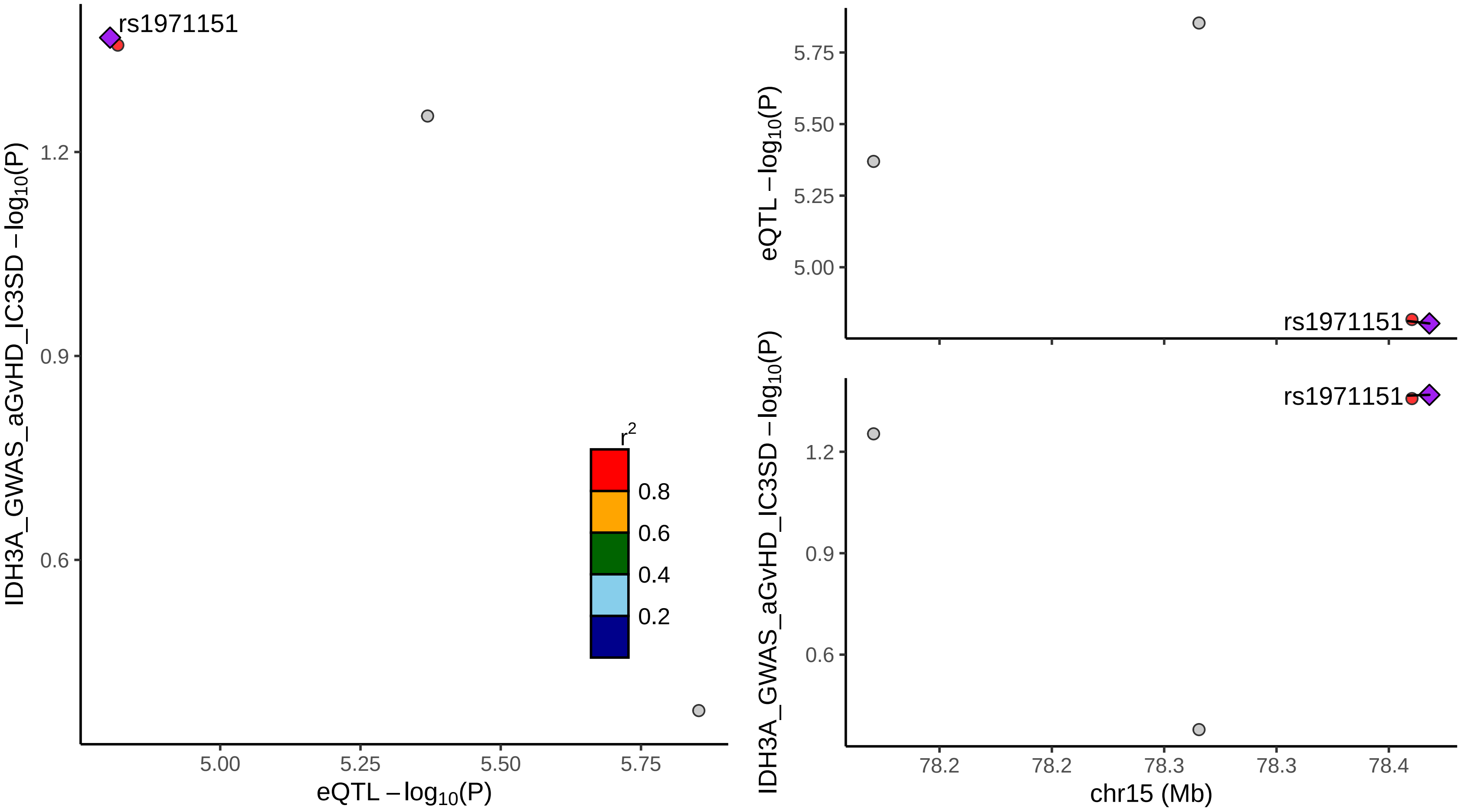

rs1971151
IDH3A_GWAS_aGvHD_IC3SD −log10(P)
1.2
0.9
0.6
r2
0.8
0.6
0.4
0.2
5.00
5.25
5.50
5.75
eQTL −log10(P)
5.75
5.50
5.25
5.00
rs1971151
78.2
78.2
78.3
78.3
78.4
chr15 (Mb)

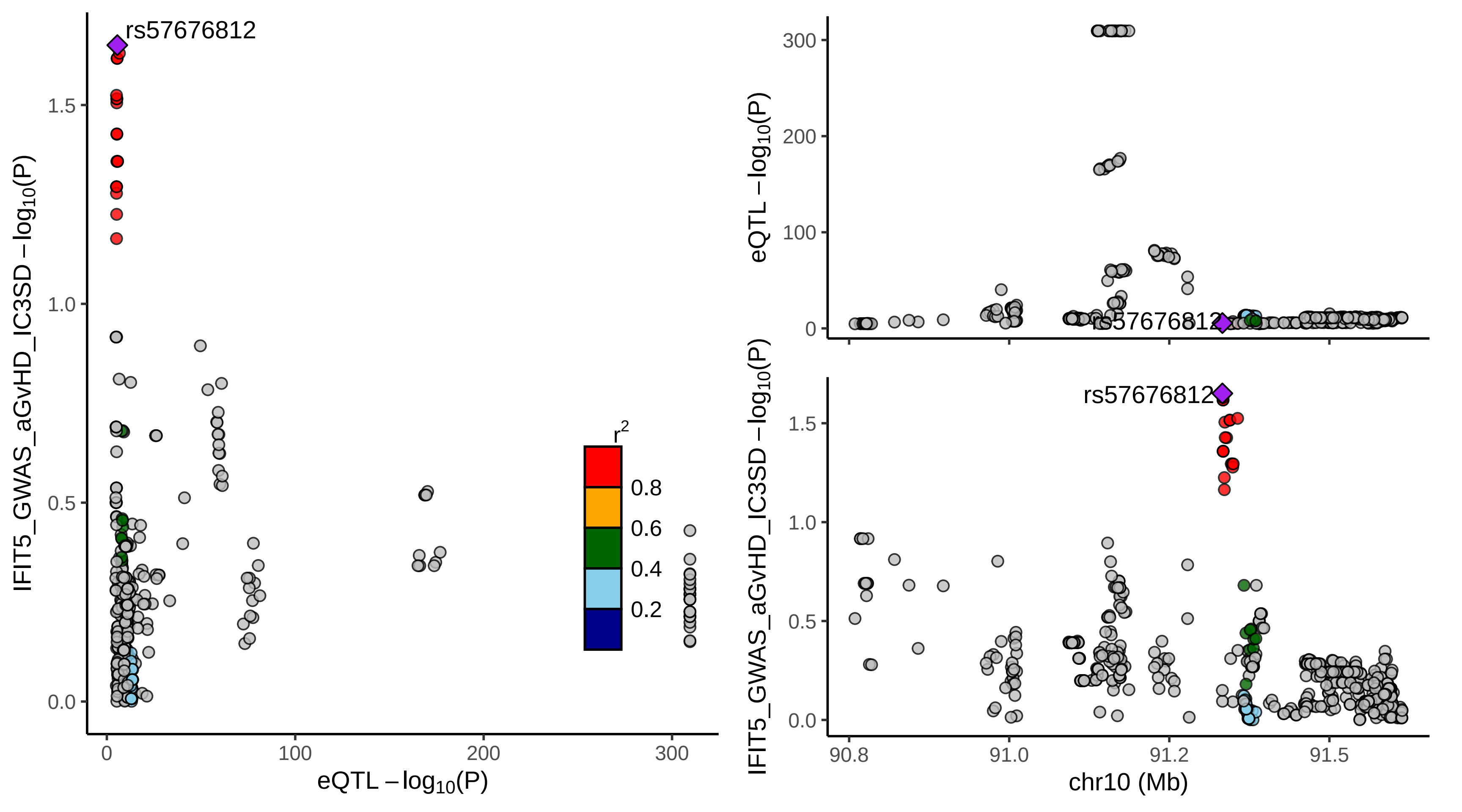
rs57676812
IFIT5_GWAS_aGvHD_IC3SD −log10(P)
eQTL −log10(P)
r2
0.8
0.6
0.4
0.2
chr10 (Mb)
90.8
91.0
91.2
91.5

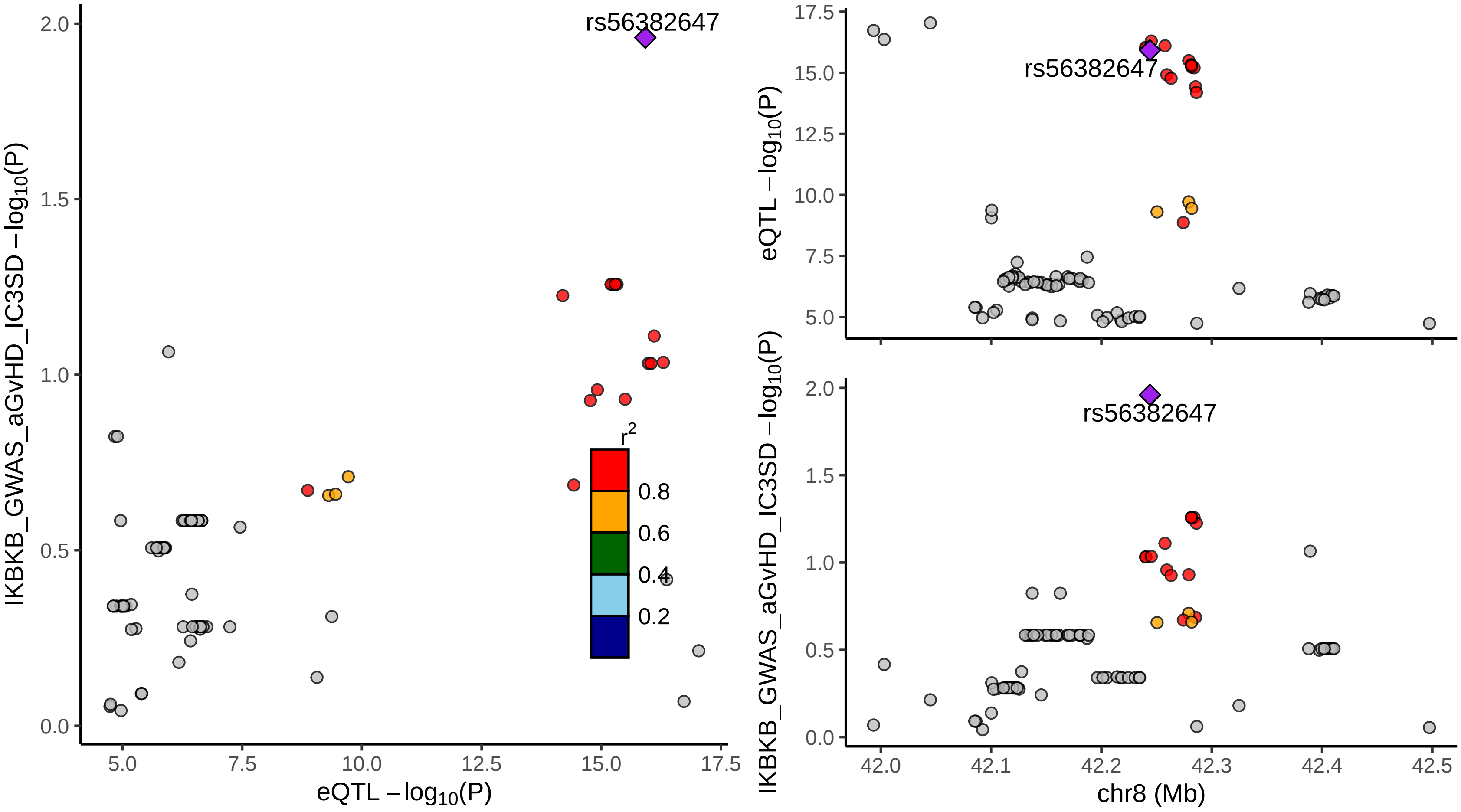

rs56382647
IKBKB_GWAS_aGvHD_IC3SD −log10(P)
eQTL −log10(P)
r2
0.8
0.6
0.4
0.2
chr8 (Mb)

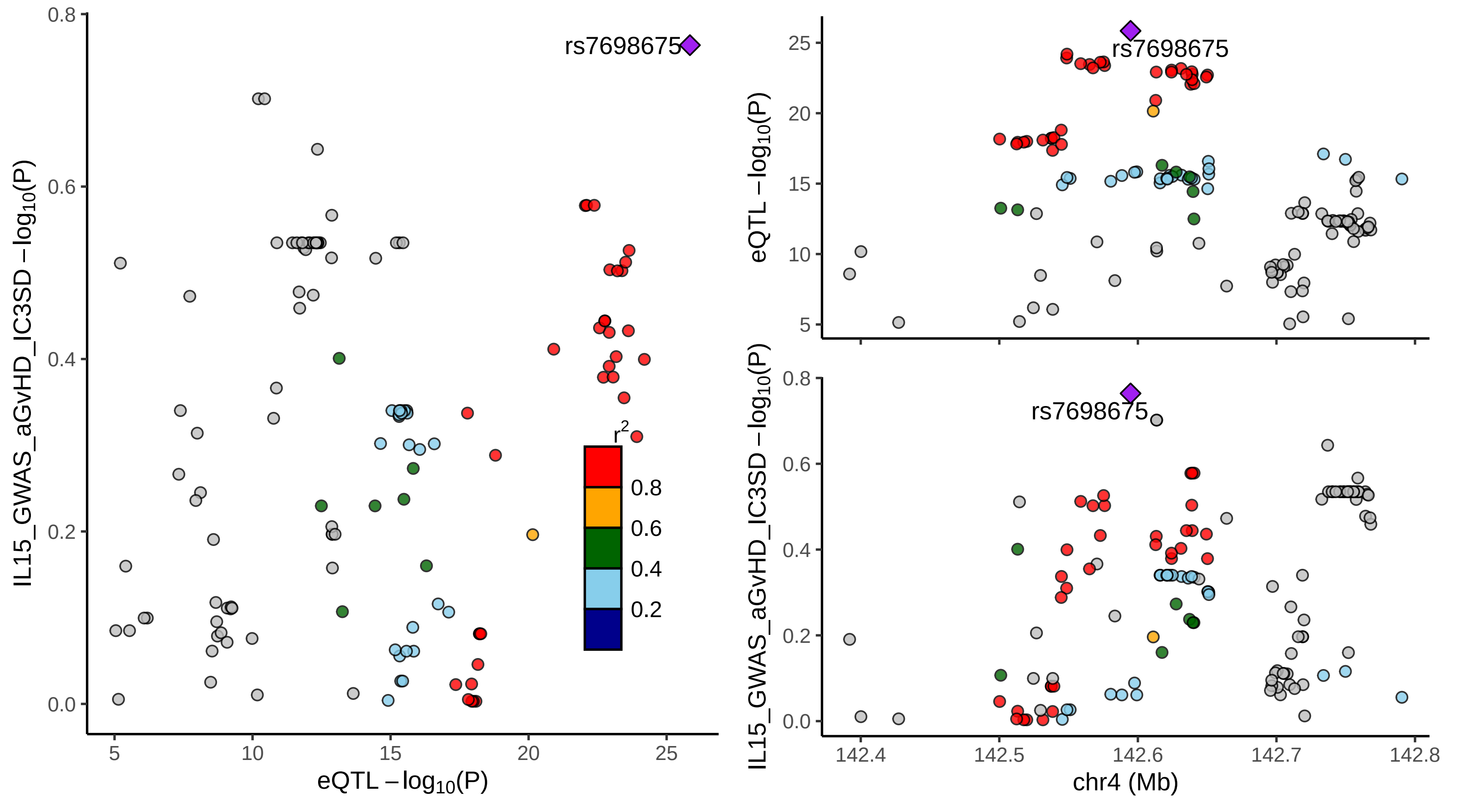
rs7698675
IL15_GWAS_aGvHD_IC3SD −log10(P)
eQTL −log10(P)
r2
0.8
0.6
0.4
0.2
chr4 (Mb)
142.4
142.5
142.6
142.7
142.8

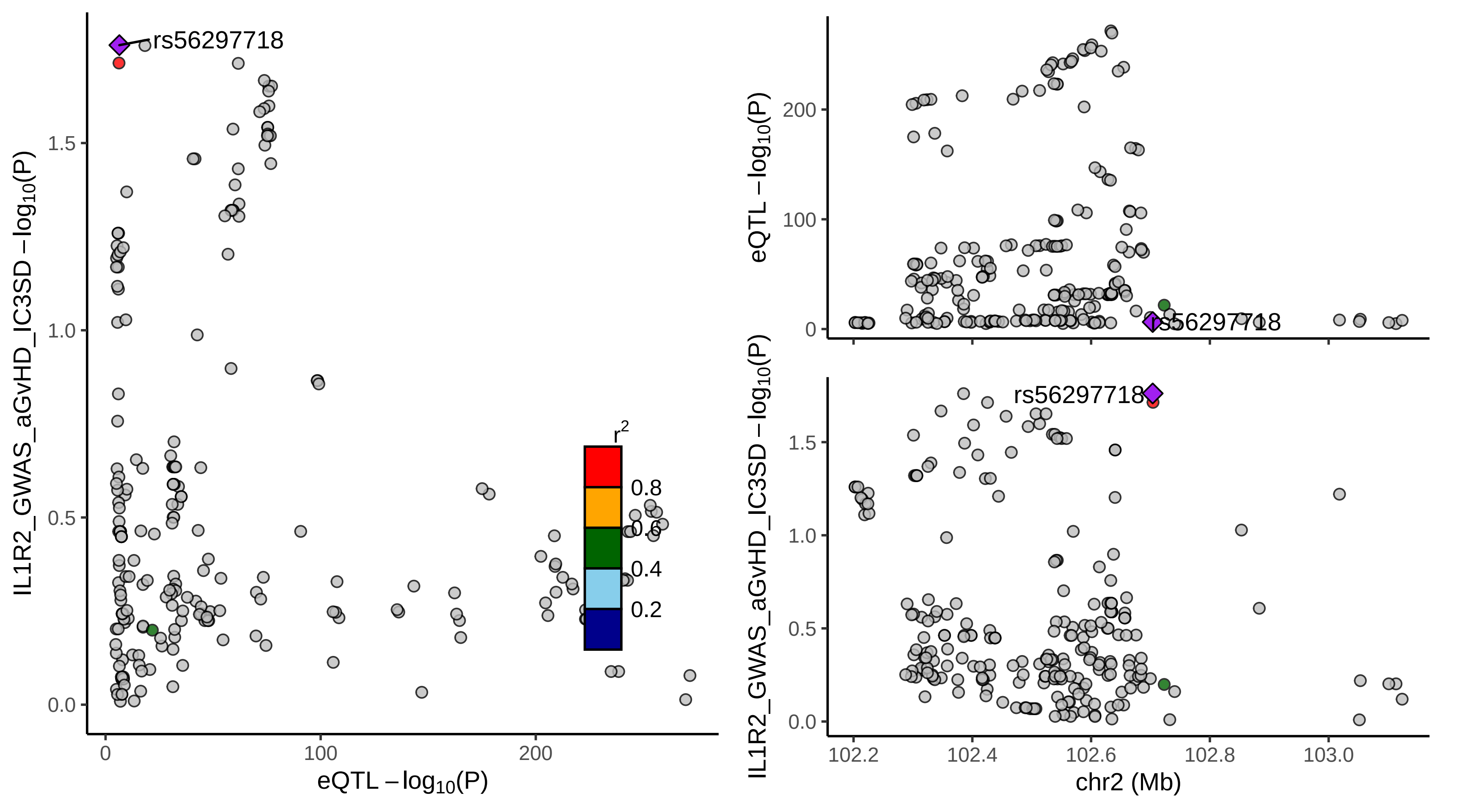
rs56297718
IL1R2_GWAS_aGvHD_IC3SD −log10(P)
eQTL −log10(P)
r2
0.8
0.6
0.4
0.2
chr2 (Mb)
0
100
200
0.0
0.5
1.0
1.5
102.2
102.4
102.6
102.8
103.0

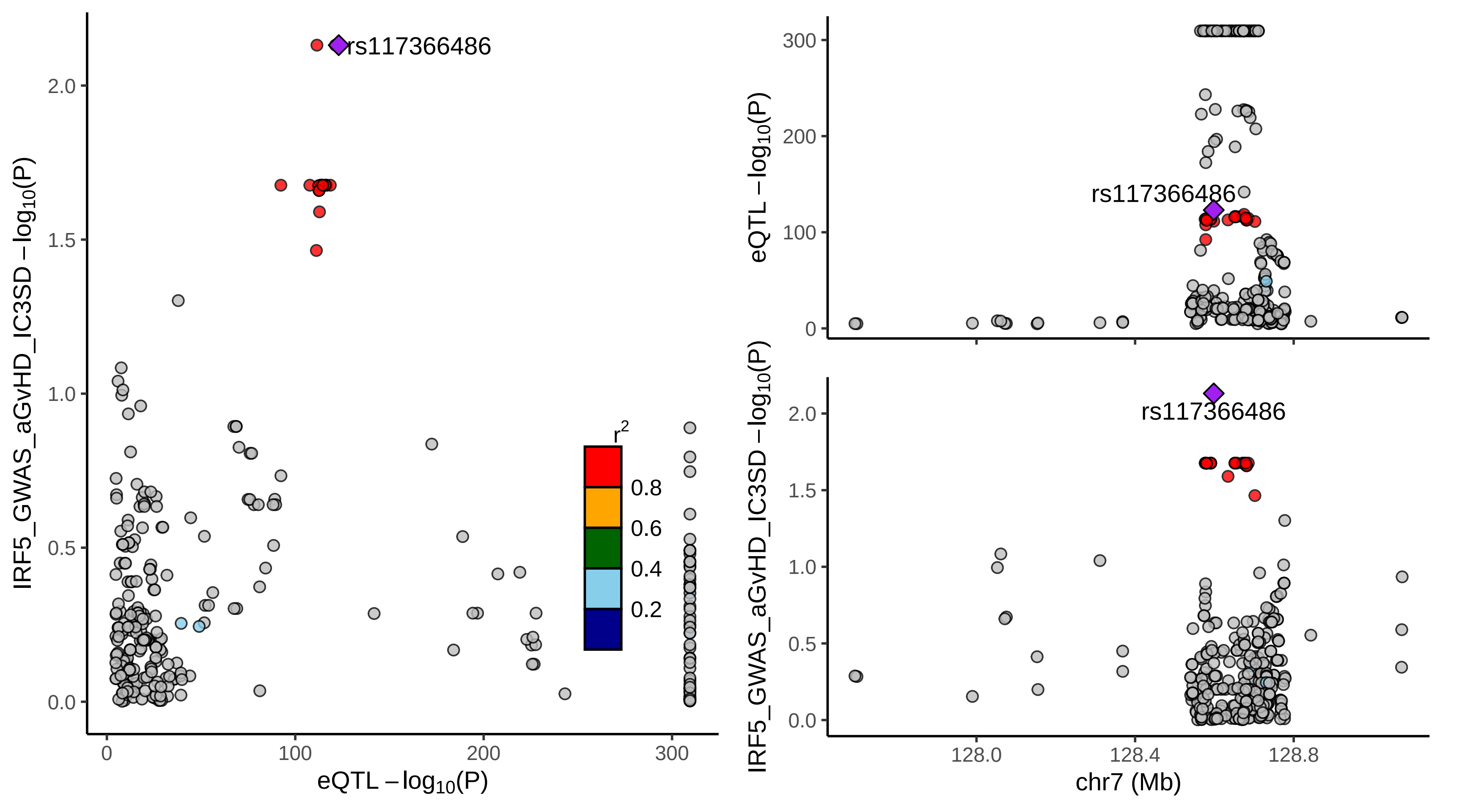
rs117366486
IRF5_GWAS_aGvHD_IC3SD −log10(P)
eQTL −log10(P)
r2
0.8
0.6
0.4
0.2
0.0
0.5
1.0
1.5
2.0
0
100
200
300
chr7 (Mb)
128.0
128.4
128.8

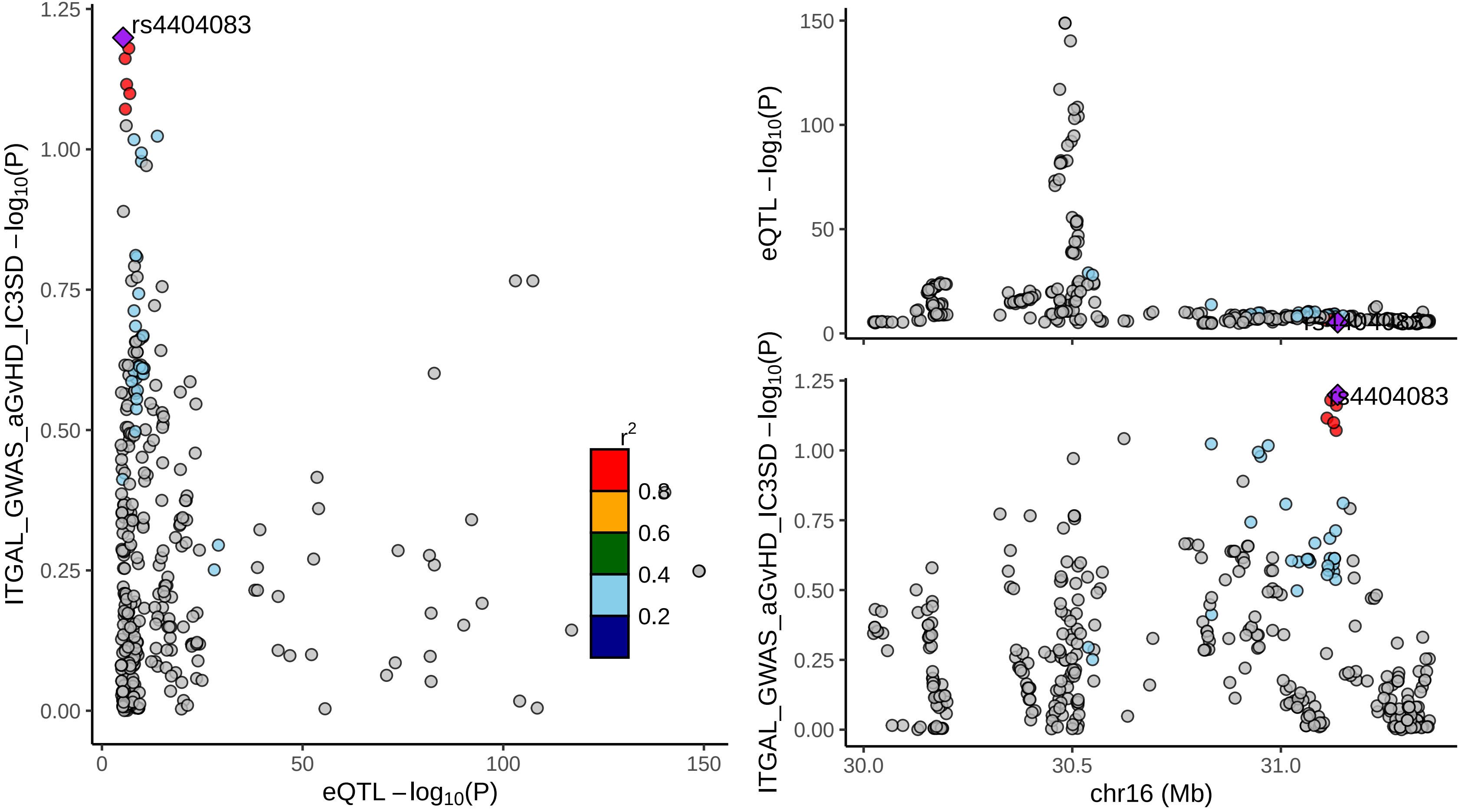
rs4404083
ITGAL_GWAS_aGvHD_IC3SD −log10(P)
eQTL −log10(P)
r2
0.8
0.6
0.4
0.2
chr16 (Mb)
30.0
30.5
31.0

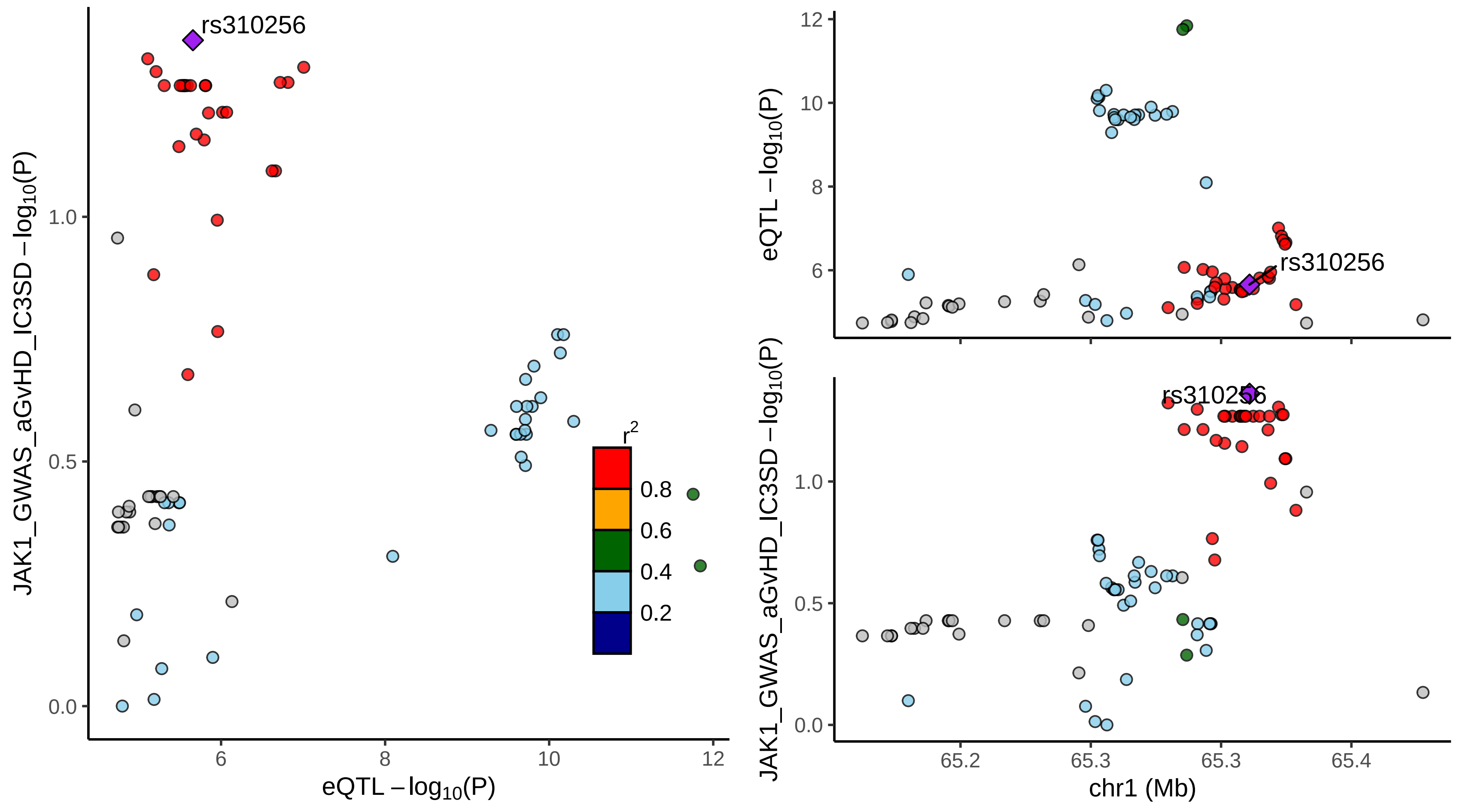

rs310256
JAK1_GWAS_aGvHD_IC3SD −log10(P)
1.0
0.5
0.0
eQTL −log10(P)
6
8
10
12
r2
0.8
0.6
0.4
0.2
eQTL −log10(P)
12
10
8
6
rs310256
JAK1_GWAS_aGvHD_IC3SD −log10(P)
rs310256
1.0
0.5
0.0
65.2
65.3
65.3
65.4
chr1 (Mb)

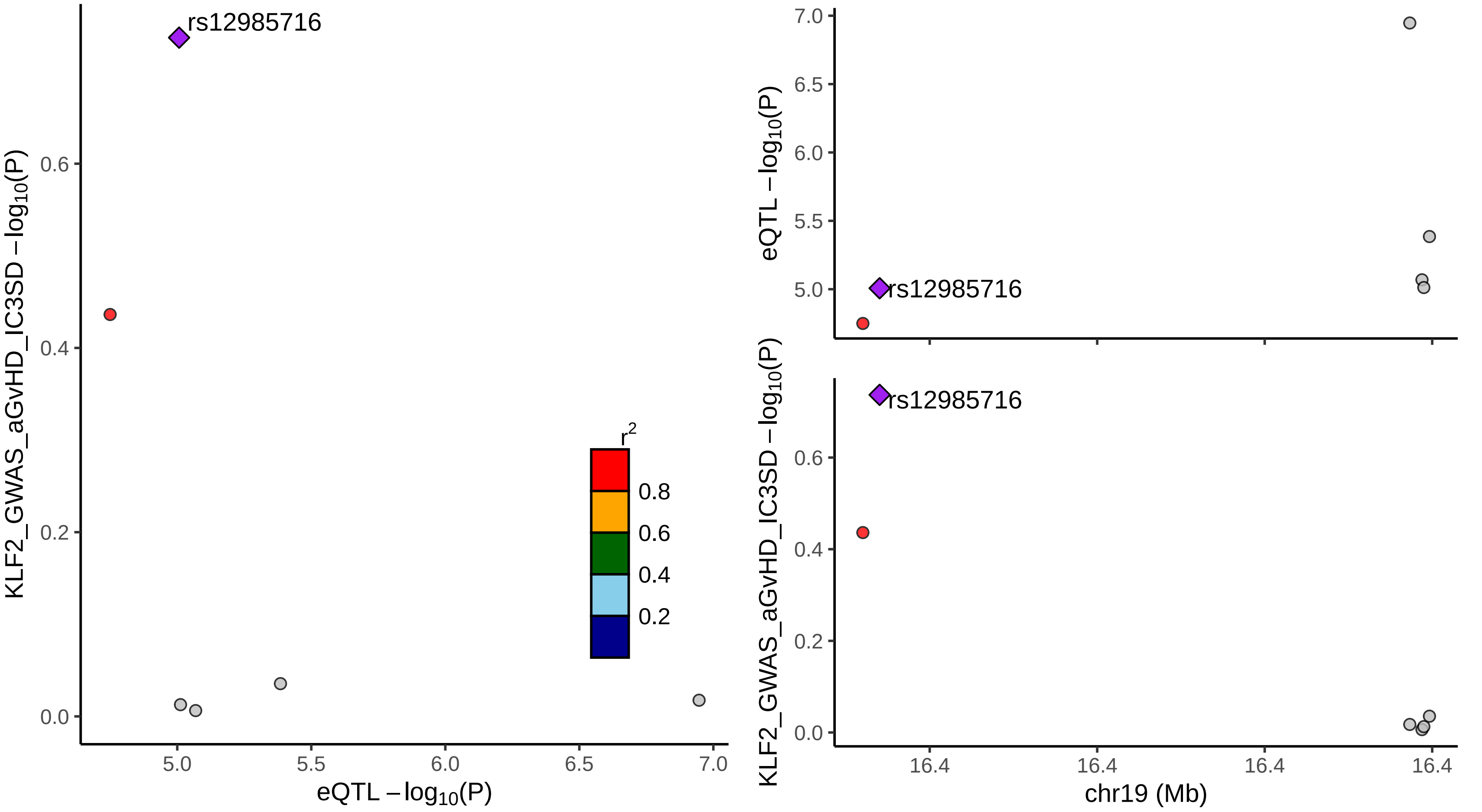
rs12985716
KLF2_GWAS_aGvHD_IC3SD −log10(P)
eQTL −log10(P)
r2
0.8
0.6
0.4
0.2
0.0
0.2
0.4
0.6
5.0
5.5
6.0
6.5
7.0
eQTL −log10(P)
rs12985716
5.0
5.5
6.0
6.5
7.0
KLF2_GWAS_aGvHD_IC3SD −log10(P)
rs12985716
16.4
16.4
16.4
16.4
chr19 (Mb)

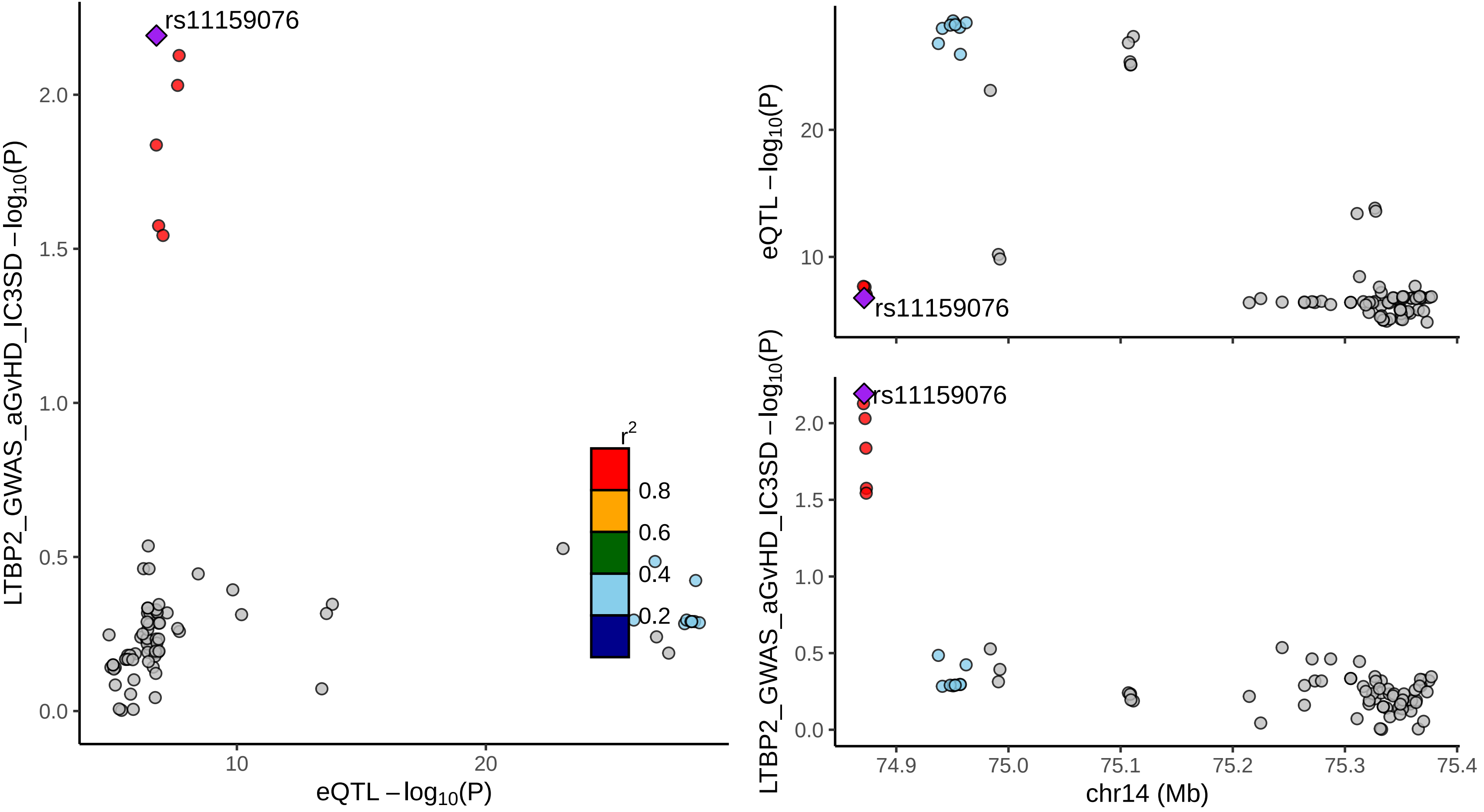
rs11159076
LTBP2_GWAS_aGvHD_IC3SD −log10(P)
eQTL −log10(P)
r2
0.8
0.6
0.4
0.2
0.0
0.5
1.0
1.5
2.0
10
20
chr14 (Mb)
74.9
75.0
75.1
75.2
75.3
75.4

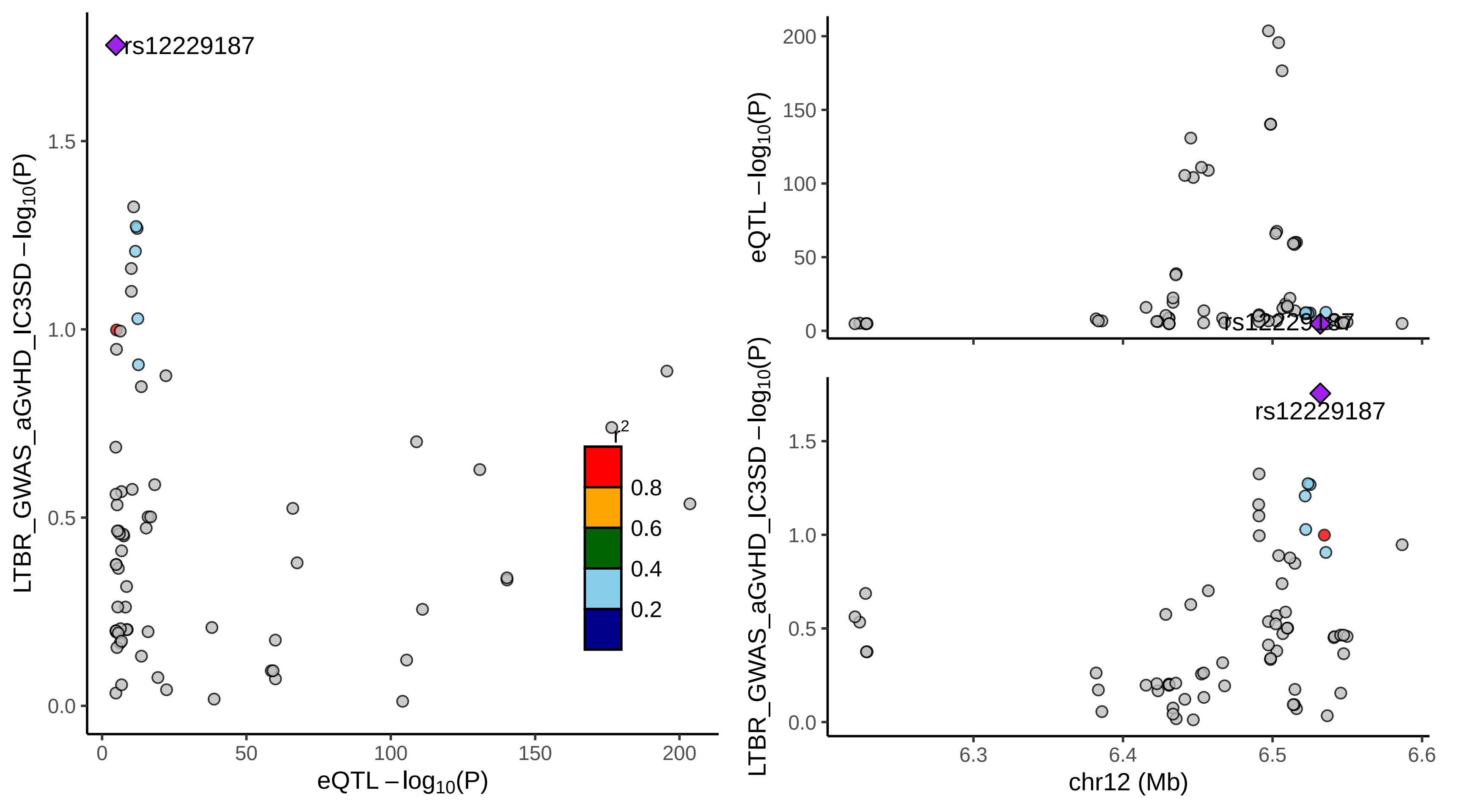

rs12229187
LTBR_GWAS_aGvHD_IC3SD −log10(P)
eQTL −log10(P)
r2
0.8
0.6
0.4
0.2
chr12 (Mb)

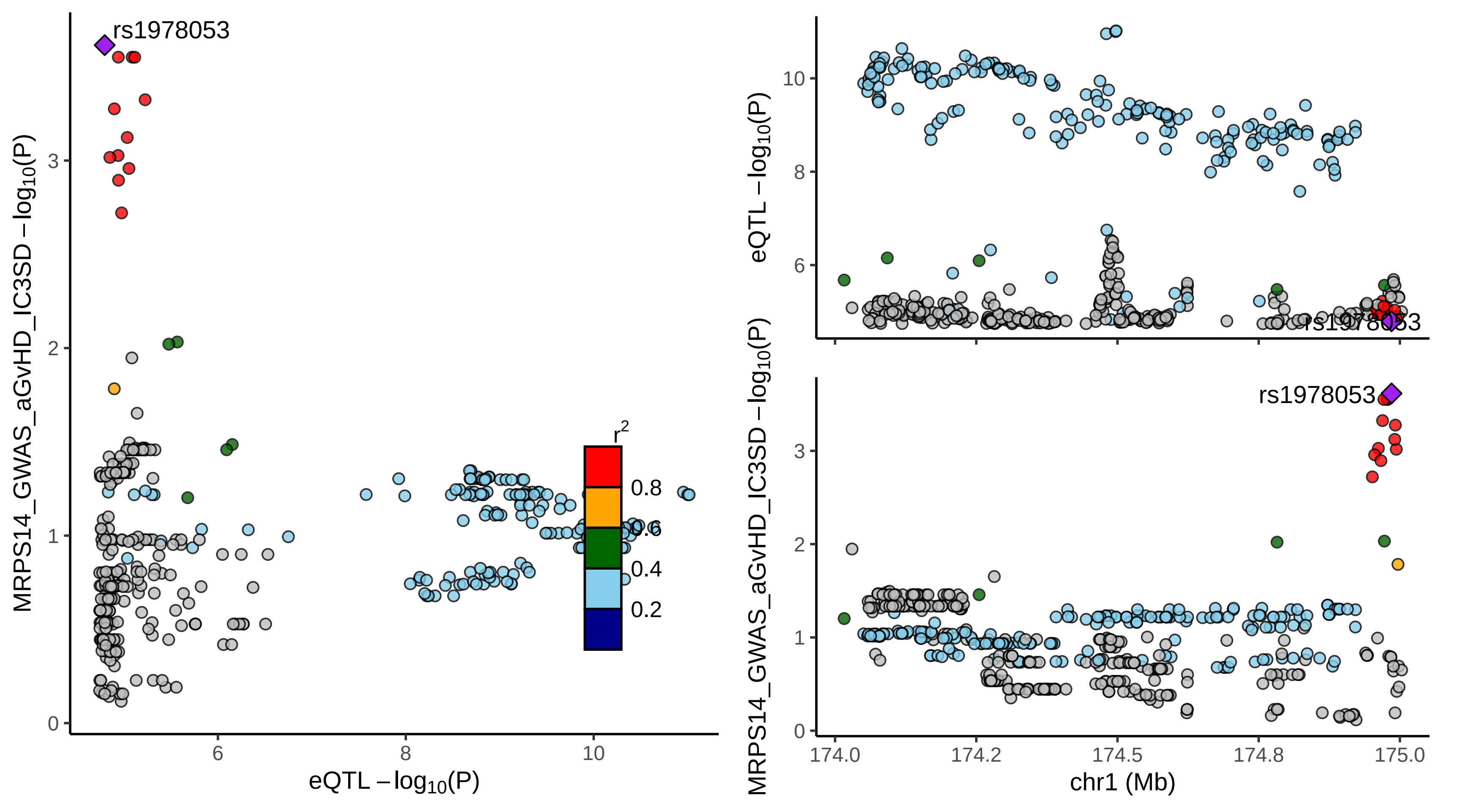
rs1978053
MRPS14_GWAS_aGvHD_IC3SD −log10(P)
eQTL −log10(P)
r2
0.8
0.6
0.4
0.2
eQTL −log10(P)
rs1978053
MRPS14_GWAS_aGvHD_IC3SD −log10(P)
rs1978053
chr1 (Mb)
174.0
174.2
174.5
174.8
175.0

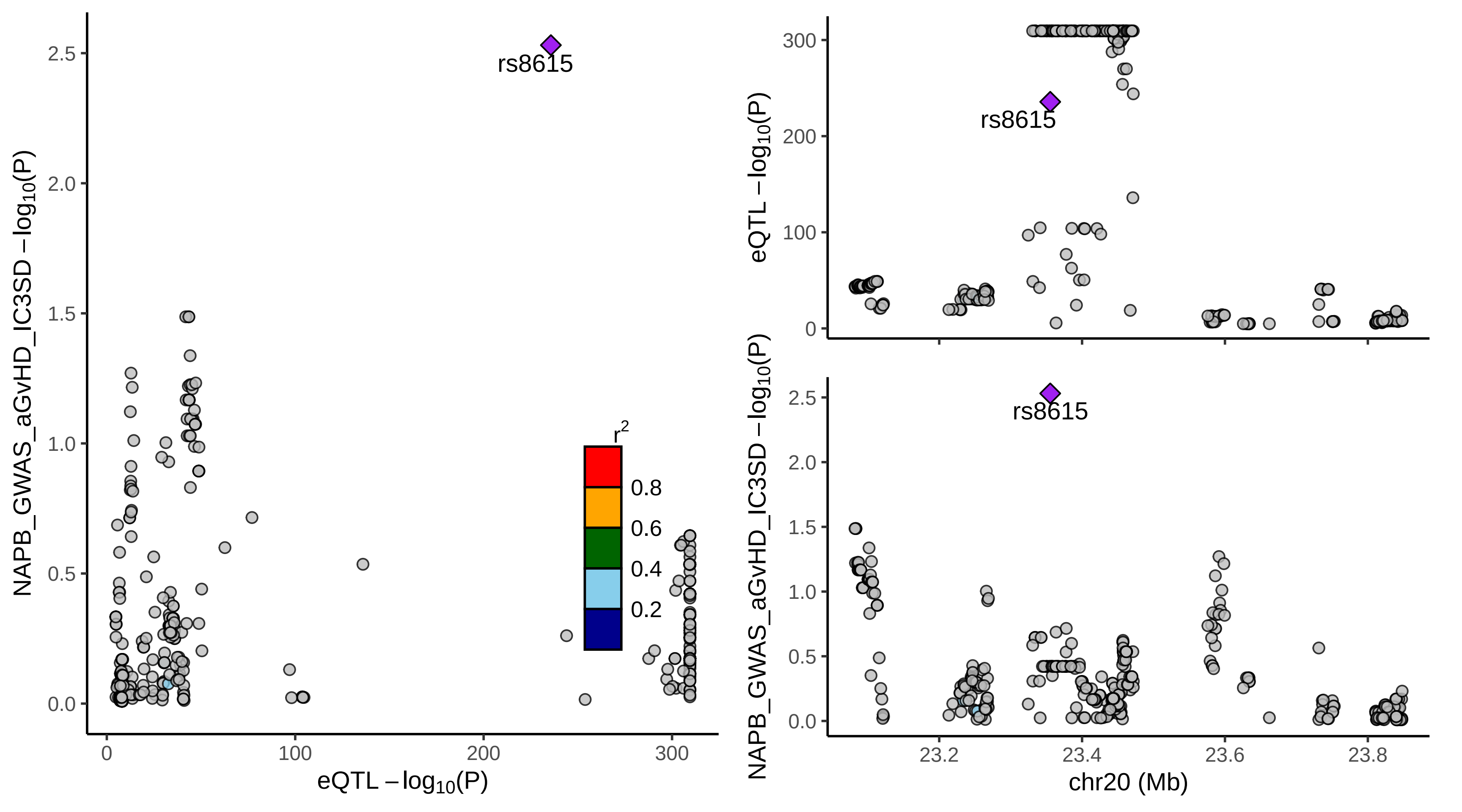

rs8615
NAPB_GWAS_aGvHD_IC3SD −log10(P)
eQTL −log10(P)
r2
0.8
0.6
0.4
0.2
0
100
200
300
0.0
0.5
1.0
1.5
2.0
2.5
chr20 (Mb)
23.2
23.4
23.6
23.8

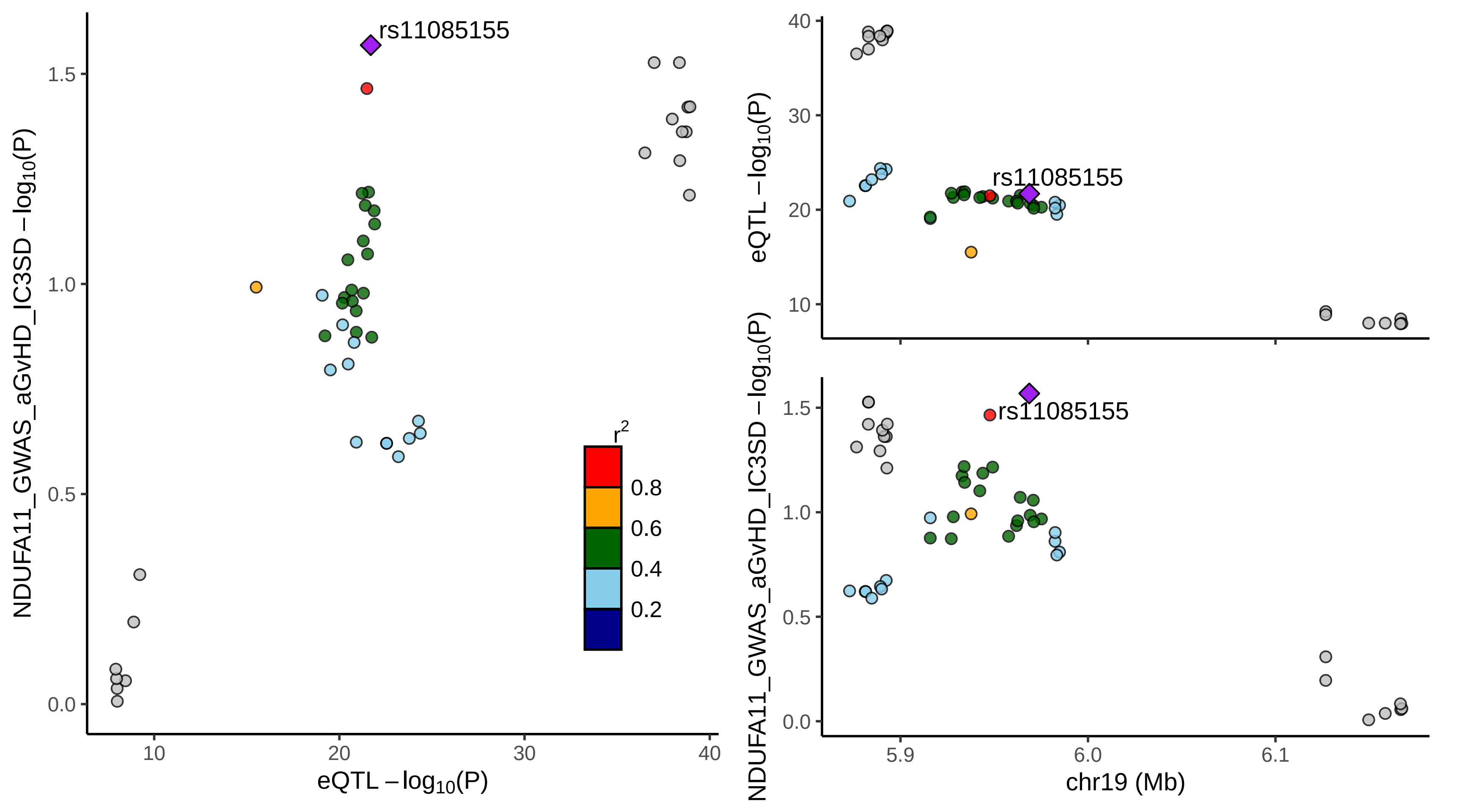

rs11085155
NDUFA11_GWAS_aGvHD_IC3SD −log10(P)
eQTL −log10(P)
r2
0.8
0.6
0.4
0.2
chr19 (Mb)
5.9
6.0
6.1

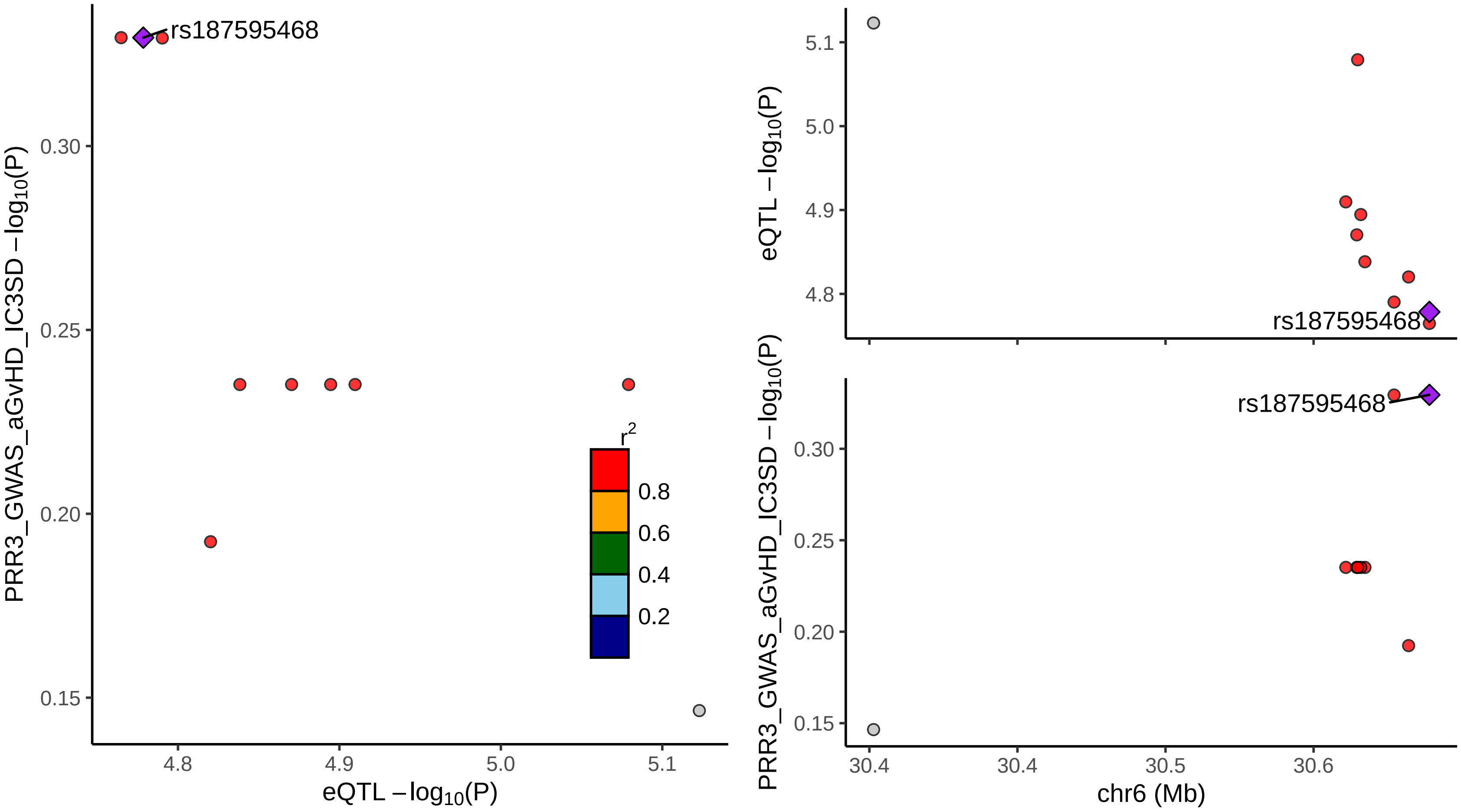
rs187595468
PRR3_GWAS_aGvHD_IC3SD −log10(P)
0.30
0.25
0.20
0.15
4.8
4.9
5.0
5.1
eQTL −log10(P)
r2
0.8
0.6
0.4
0.2
eQTL −log10(P)
5.1
5.0
4.9
4.8
rs187595468
PRR3_GWAS_aGvHD_IC3SD −log10(P)
rs187595468
0.30
0.25
0.20
0.15
30.4
30.4
30.5
30.6
chr6 (Mb)

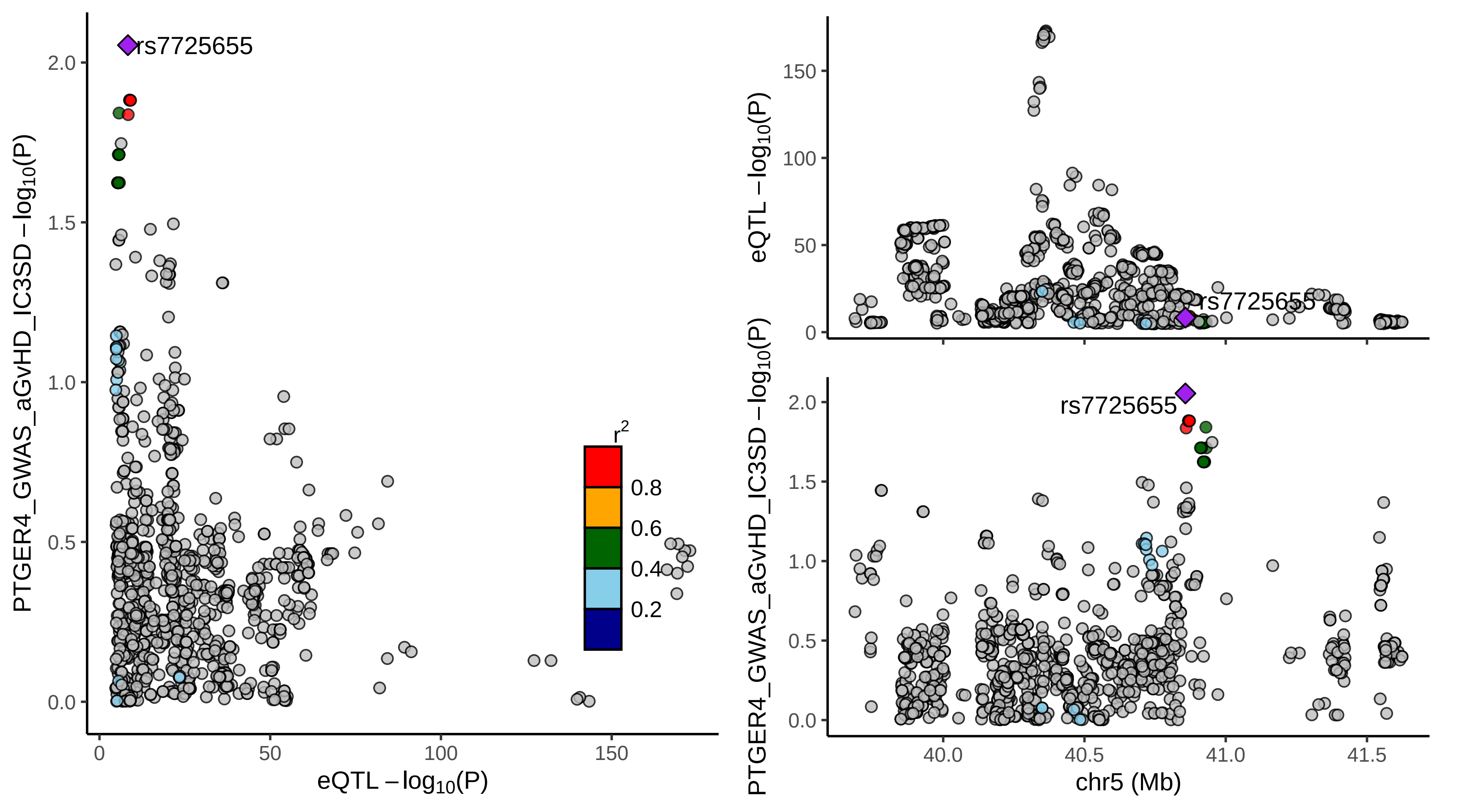
rs7725655
PTGER4_GWAS_aGvHD_IC3SD −log10(P)
eQTL −log10(P)
r2
0.8
0.6
0.4
0.2
eQTL −log10(P)
rs7725655
PTGER4_GWAS_aGvHD_IC3SD −log10(P)
rs7725655
chr5 (Mb)

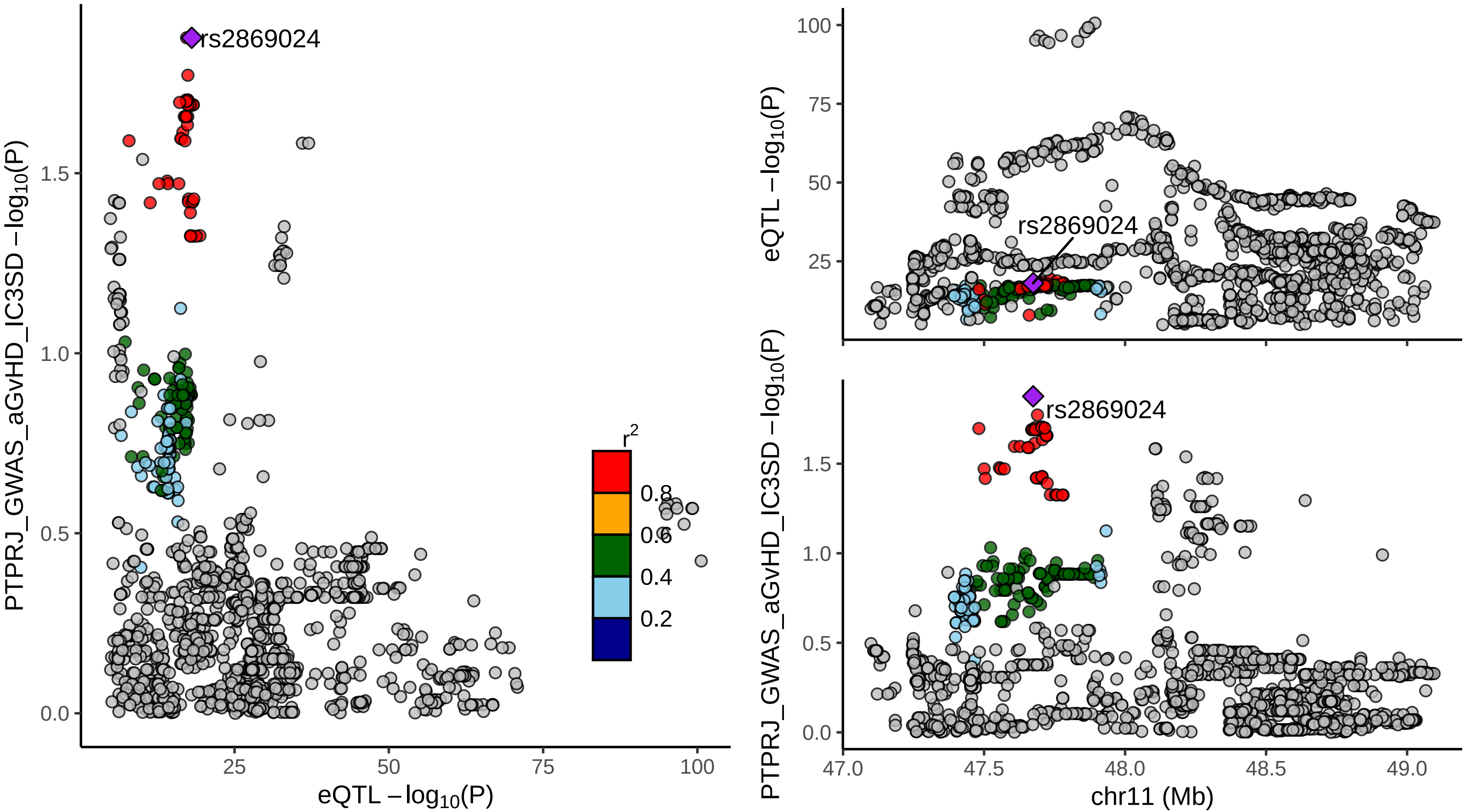

rs2869024
PTPRJ_GWAS_aGvHD_IC3SD −log10(P)
1.5
1.0
0.5
0.0
25
50
75
100
eQTL −log10(P)
r2
0.8
0.6
0.4
0.2
100
75
50
25
eQTL −log10(P)
rs2869024
rs2869024
PTPRJ_GWAS_aGvHD_IC3SD −log10(P)
1.5
1.0
0.5
0.0
47.0
47.5
48.0
48.5
49.0
chr11 (Mb)

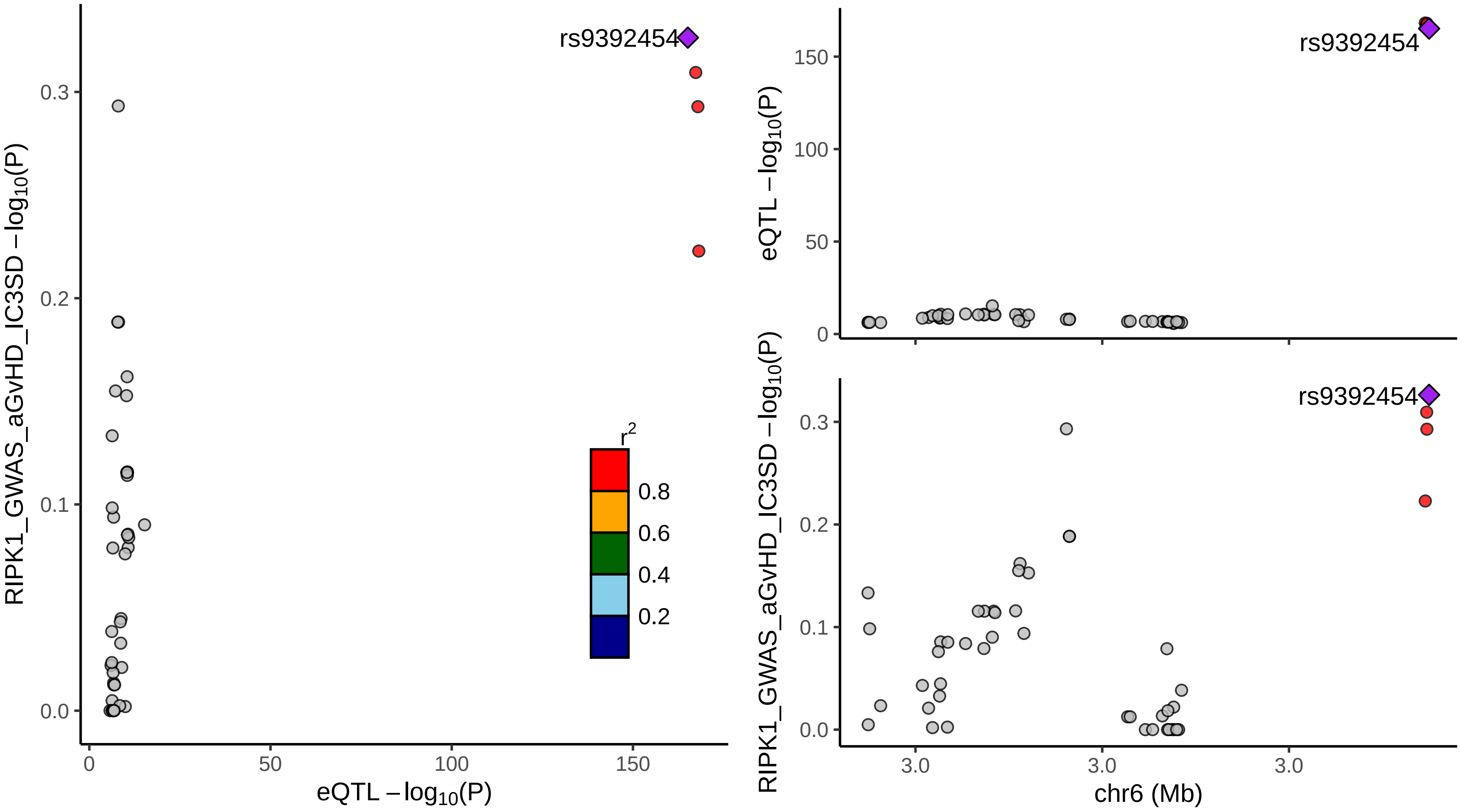
rs9392454
RIPK1_GWAS_aGvHD_IC3SD −log10(P)
eQTL −log10(P)
r2
0.8
0.6
0.4
0.2
0.3
0.2
0.1
0.0
0
50
100
150
chr6 (Mb)
3.0

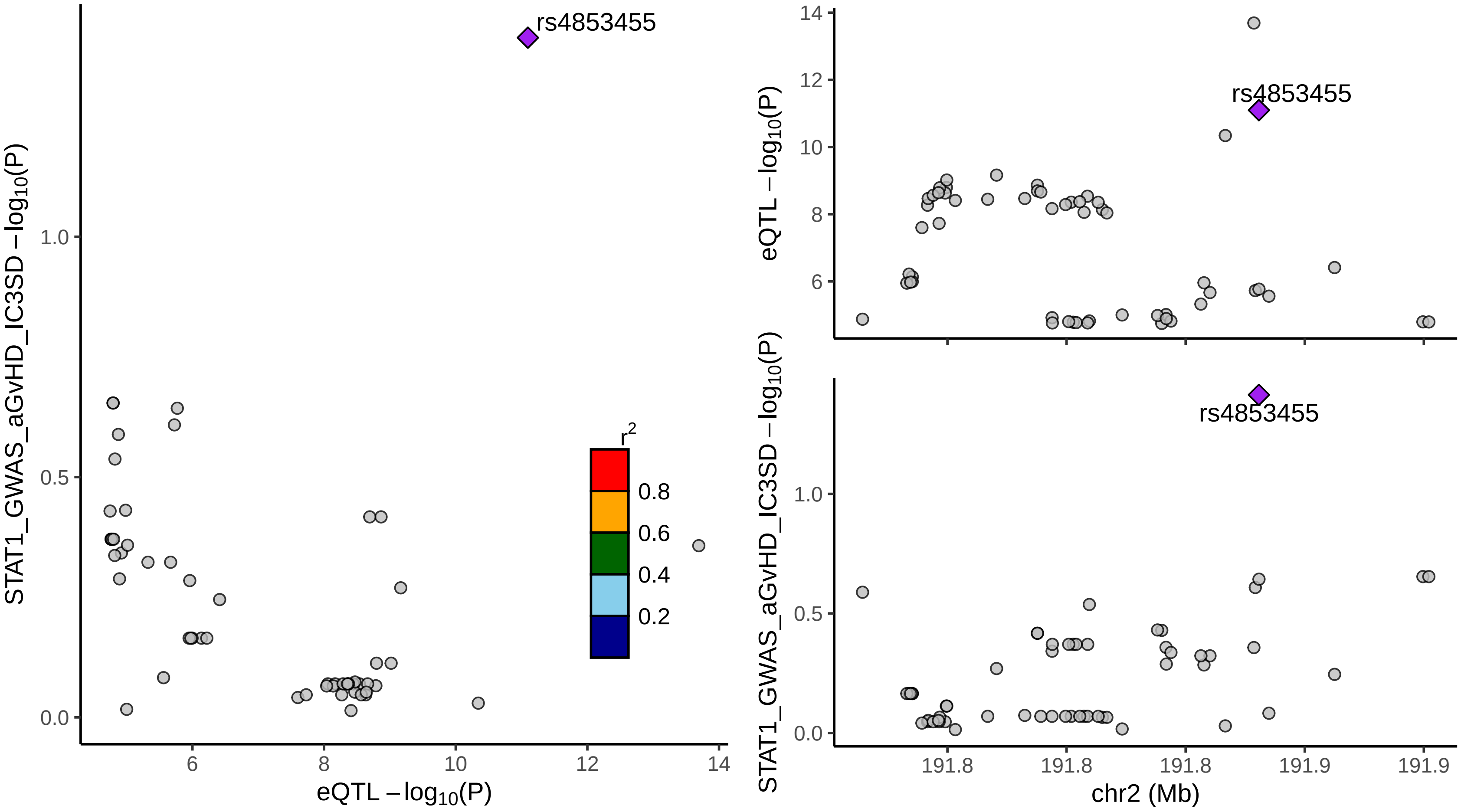
rs4853455
STAT1_GWAS_aGvHD_IC3SD −log10(P)
1.0
0.5
0.0
6
8
10
12
14
eQTL −log10(P)
r2
0.8
0.6
0.4
0.2
eQTL −log10(P)
14
12
10
8
6
rs4853455
rs4853455
STAT1_GWAS_aGvHD_IC3SD −log10(P)
191.8
191.8
191.8
191.9
191.9
chr2 (Mb)

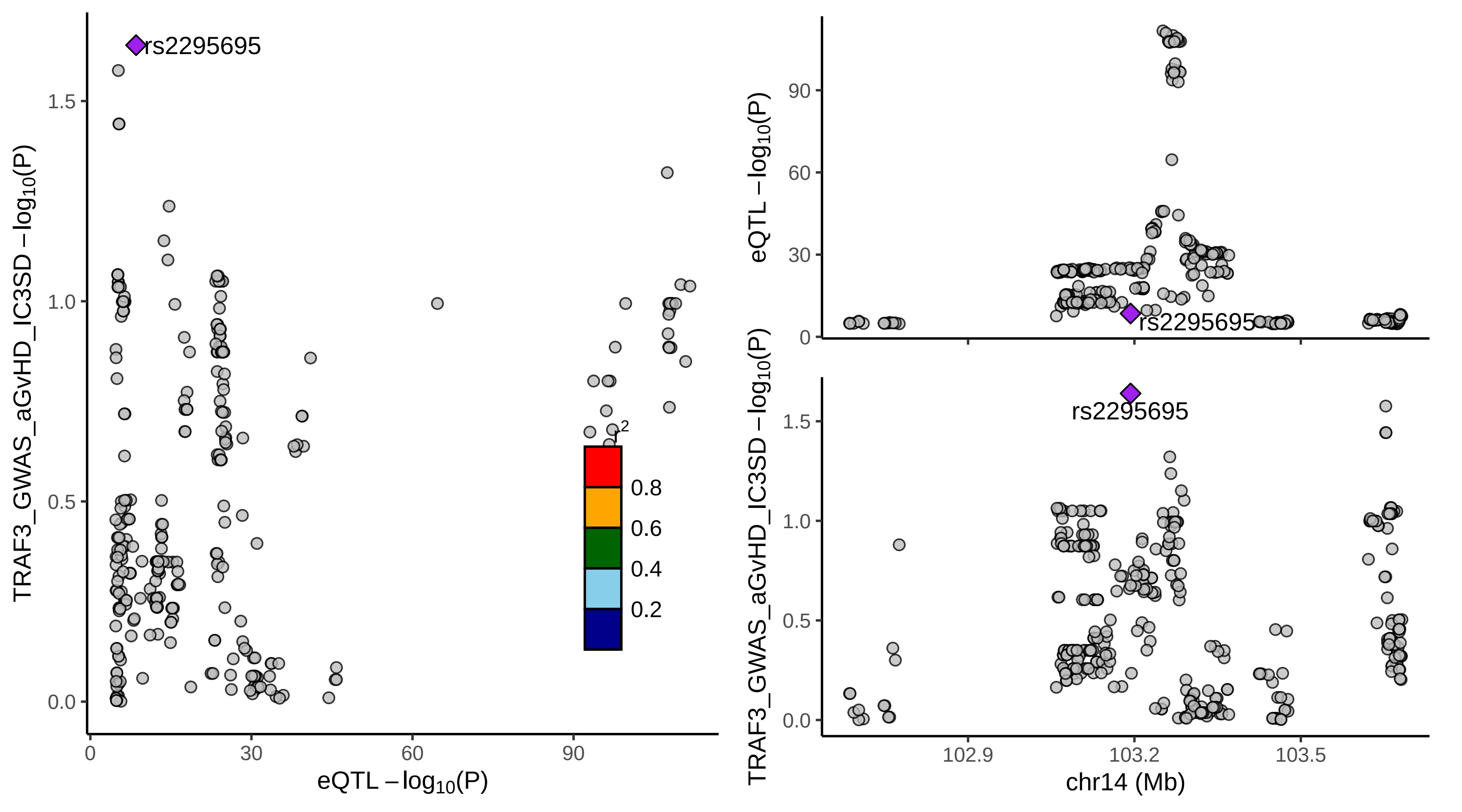

rs2295695
TRAF3_GWAS_aGvHD_IC3SD −log10(P)
eQTL −log10(P)
r2
0.8
0.6
0.4
0.2
0
30
60
90
0.0
0.5
1.0
1.5
102.9
103.2
103.5
chr14 (Mb)

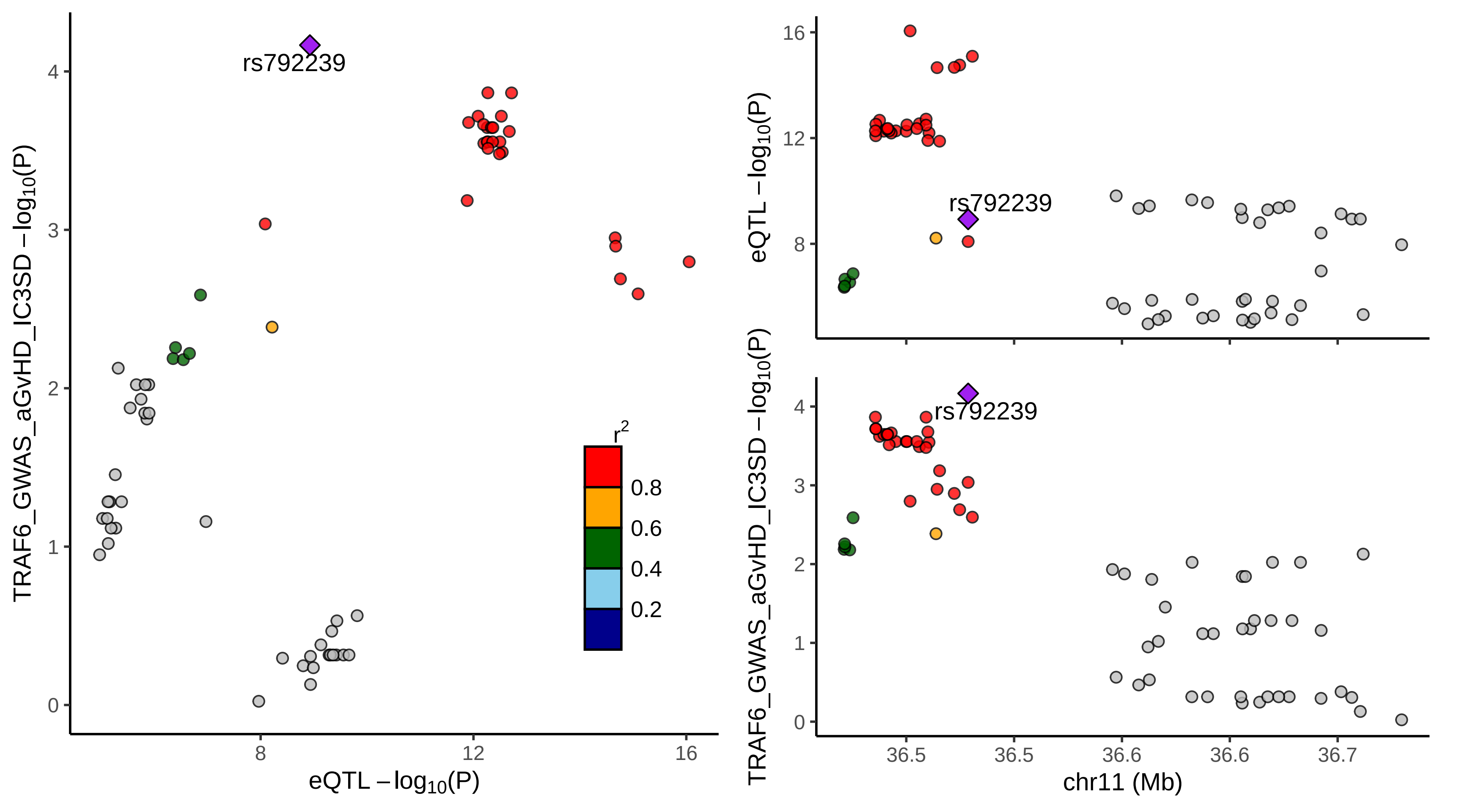

rs792239
TRAF6_GWAS_aGvHD_IC3SD −log10(P)
eQTL −log10(P)
r2
0.8
0.6
0.4
0.2
chr11 (Mb)
36.5
36.6
36.7

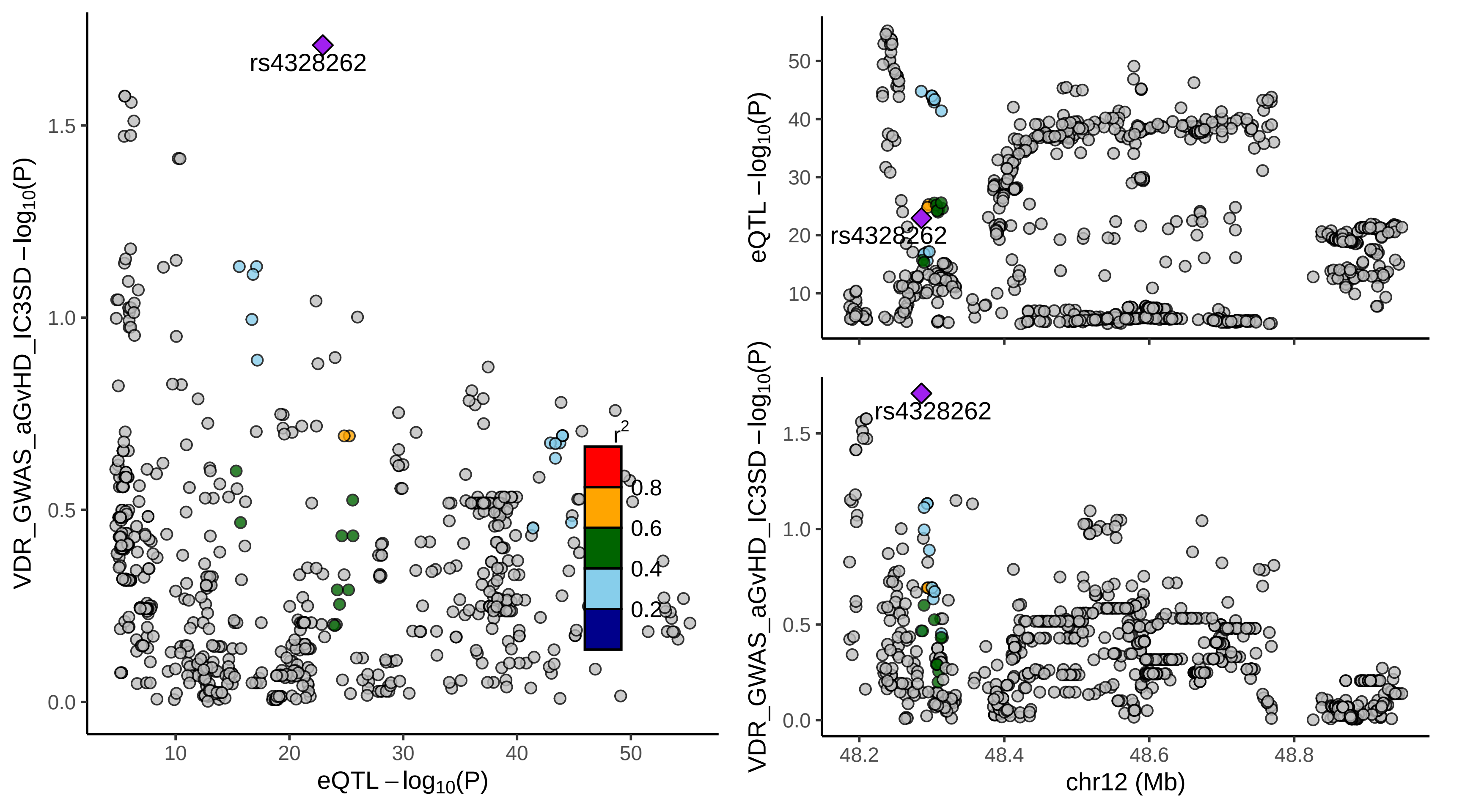

rs4328262
VDR_GWAS_aGvHD_IC3SD $-\log_{10}(P)$
eQTL $-\log_{10}(P)$
0.0
0.5
1.0
1.5
10
20
30
40
50
r^2
0.8
0.6
0.4
0.2
chr12 (Mb)
48.2
48.4
48.6
48.8

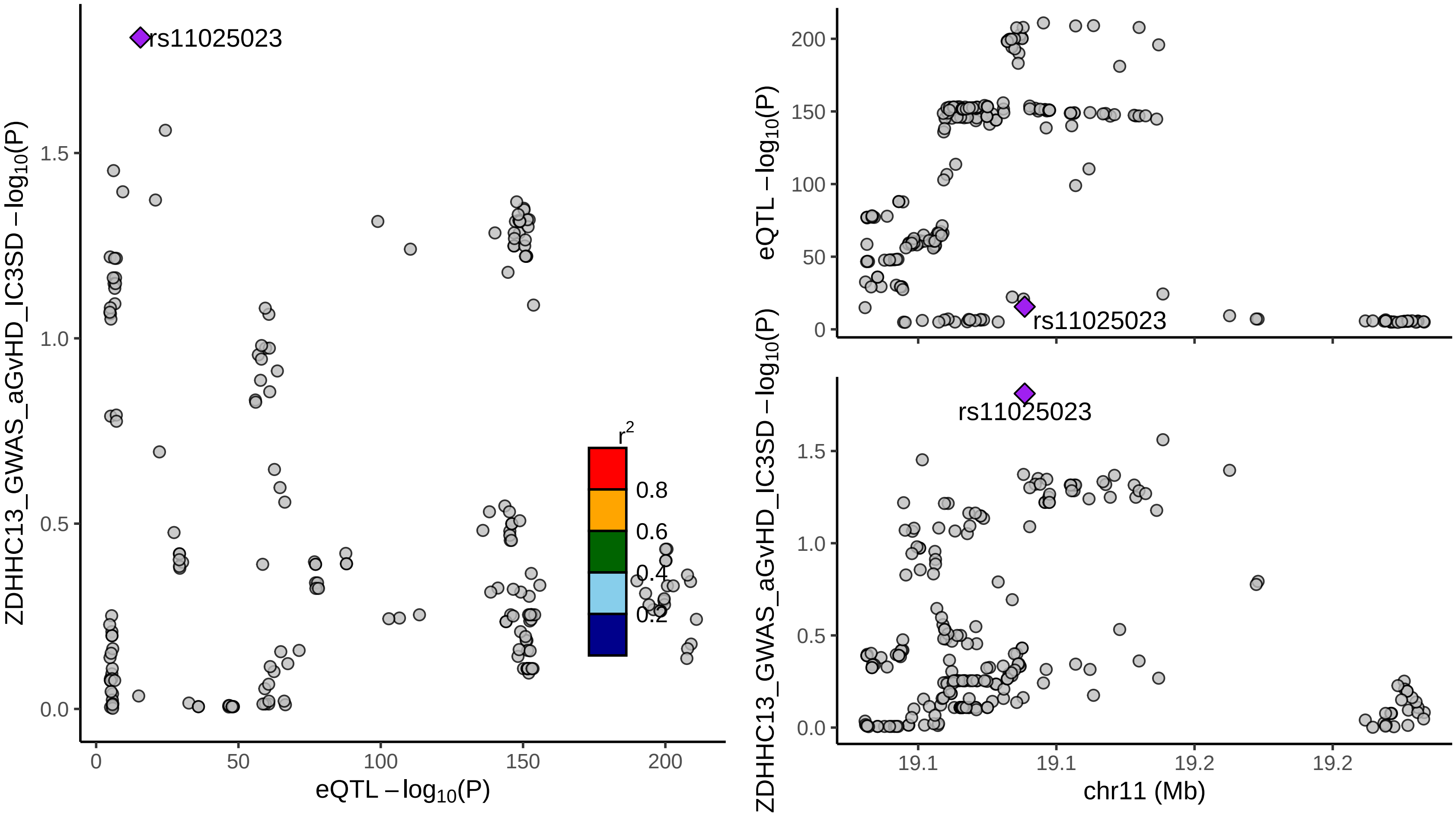
rs11025023
ZDHHC13_GWAS_aGvHD_IC3SD −log10(P)
eQTL −log10(P)
0
50
100
150
200
0.0
0.5
1.0
1.5
r2
0.8
0.6
0.4
0.2
rs11025023
rs11025023
chr11 (Mb)
19.1
19.1
19.2
19.2

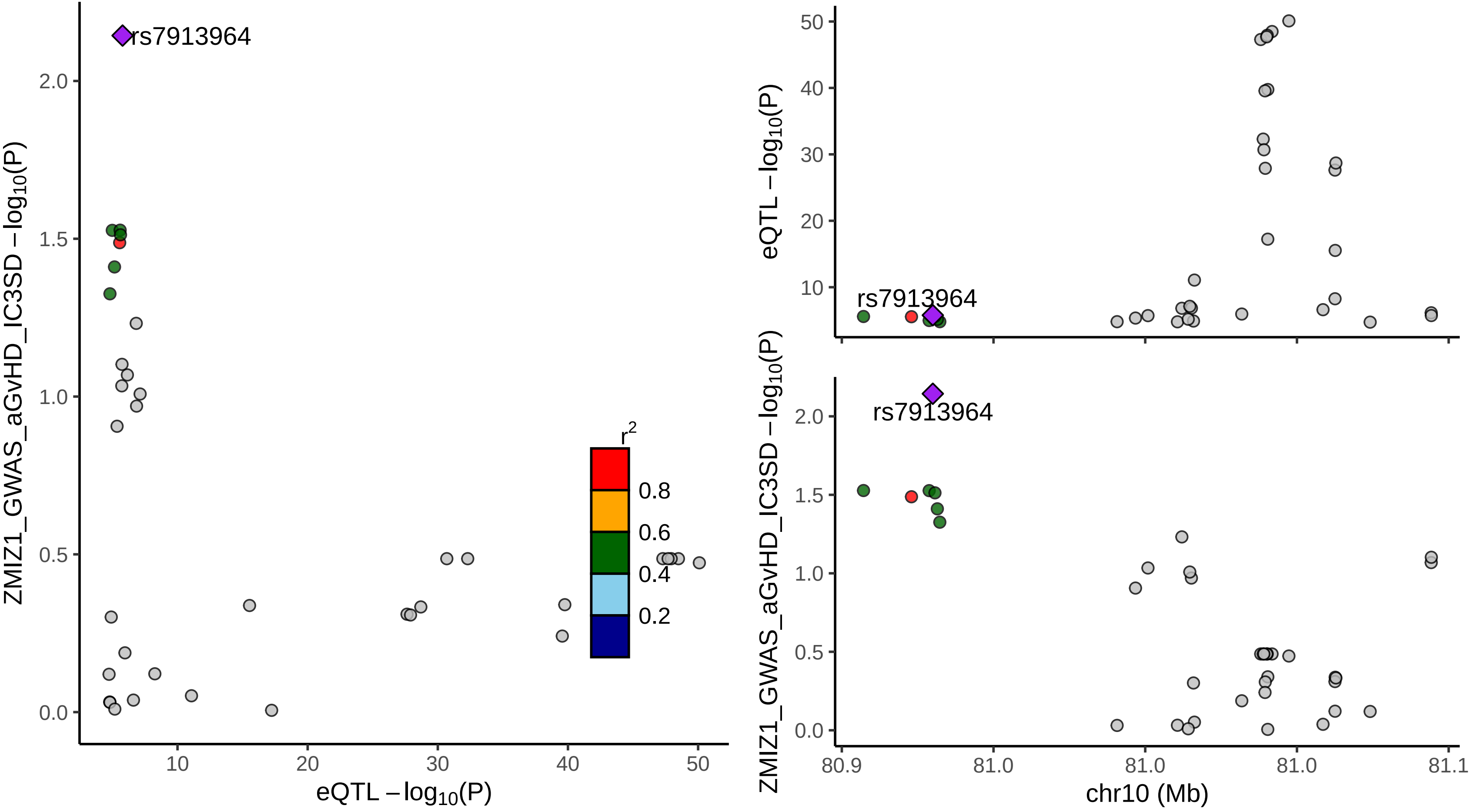

rs7913964
ZMIZ1_GWAS_aGvHD_IC3SD −log10(P)
2.0
1.5
1.0
0.5
0.0
10
20
30
40
50
eQTL −log10(P)
r2
0.8
0.6
0.4
0.2
eQTL −log10(P)
50
40
30
20
10
rs7913964
ZMIZ1_GWAS_aGvHD_IC3SD −log10(P)
rs7913964
2.0
1.5
1.0
0.5
0.0
80.9
81.0
81.0
81.0
81.1
chr10 (Mb)

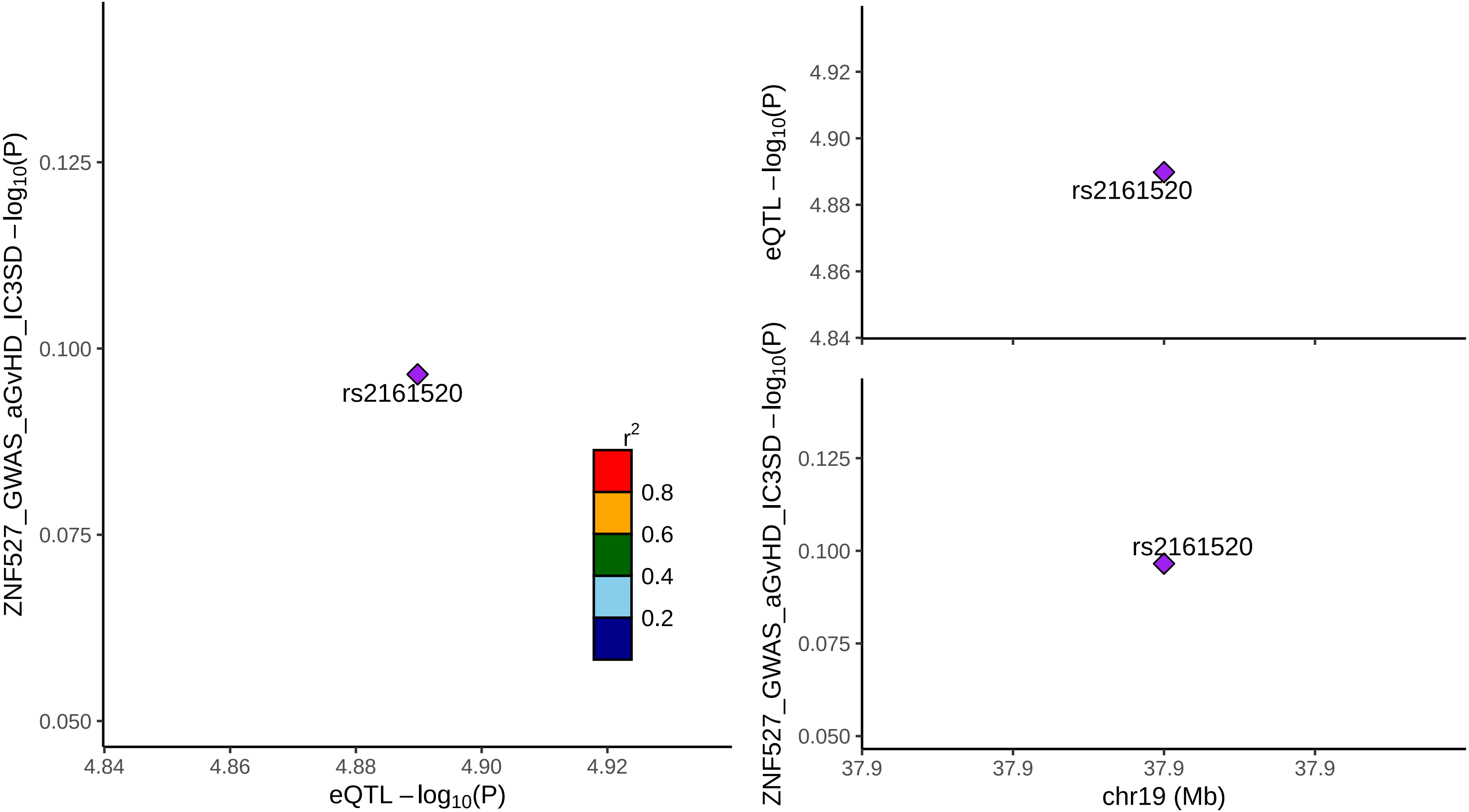
ZNF527_GWAS_aGvHD_IC3SD −log10(P)
0.125
0.100
0.075
0.050
rs2161520
r2
0.8
0.6
0.4
0.2
4.84
4.86
4.88
4.90
4.92
eQTL −log10(P)
eQTL −log10(P)
4.92
4.90
4.88
4.86
4.84
rs2161520
ZNF527_GWAS_aGvHD_IC3SD −log10(P)
0.125
0.100
0.075
0.050
rs2161520
37.9
37.9
37.9
37.9
chr19 (Mb)
