# Supplementary material for: Meta-Analysis of Genome-Wide Association and Gene Expression Studies Implicates Donor T Cell Function and Cytokine Pathways in Acute GvHD
Source: Front Immunol. 2020 Feb 3;11:19. doi: 10.3389/fimmu.2020.00019 (PMC7008714; doi:10.3389/fimmu.2020.00019)
Supplement: Supplementary file 4 [file Data_Sheet_4.pdf]

LocusCompare plots of Finnish cohort 2

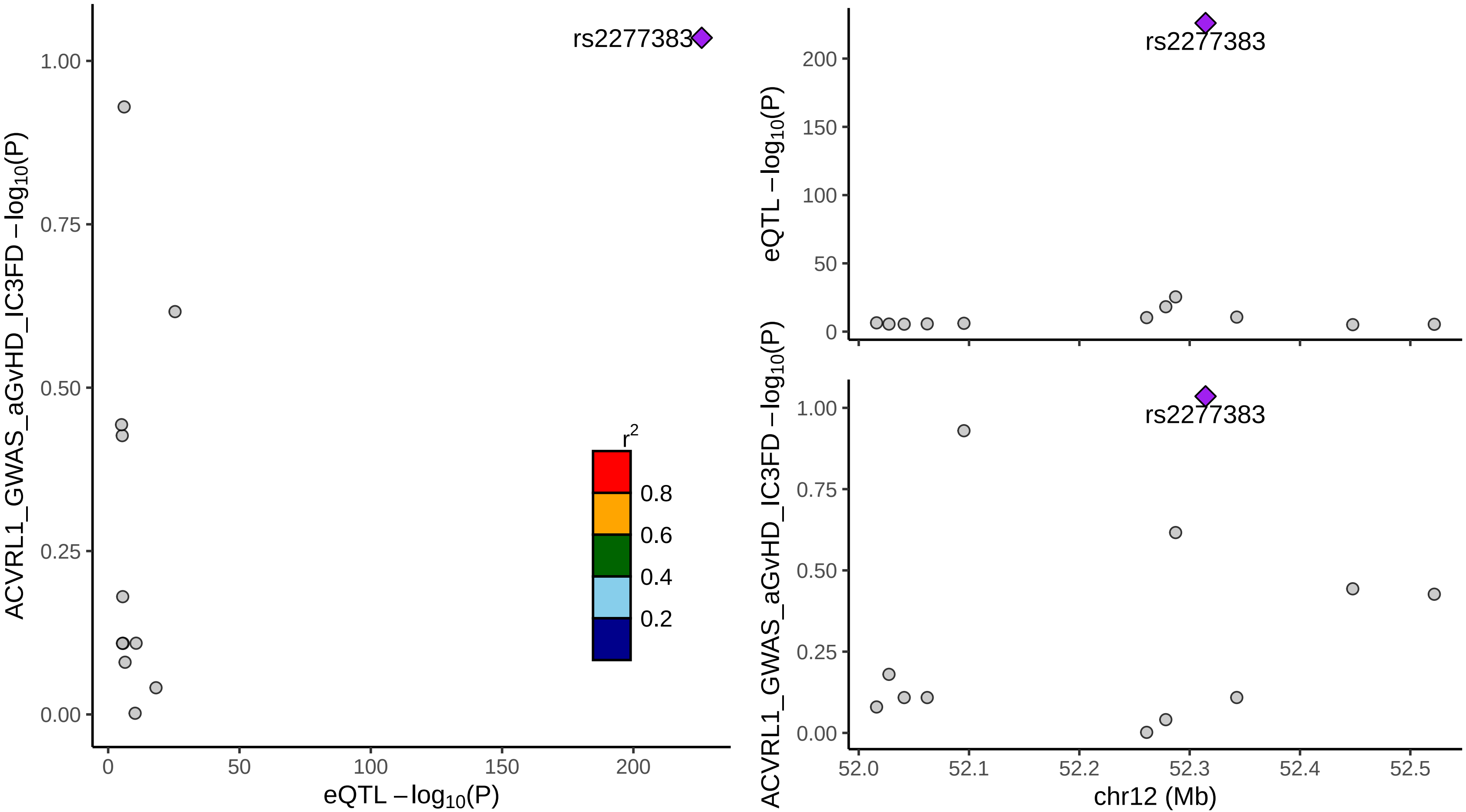

rs2277383
ACVRL1_GWAS_aGvHD_IC3FD −log10(P)
eQTL −log10(P)
r2
0.8
0.6
0.4
0.2
rs2277383
eQTL −log10(P)
rs2277383
ACVRL1_GWAS_aGvHD_IC3FD −log10(P)
chr12 (Mb)

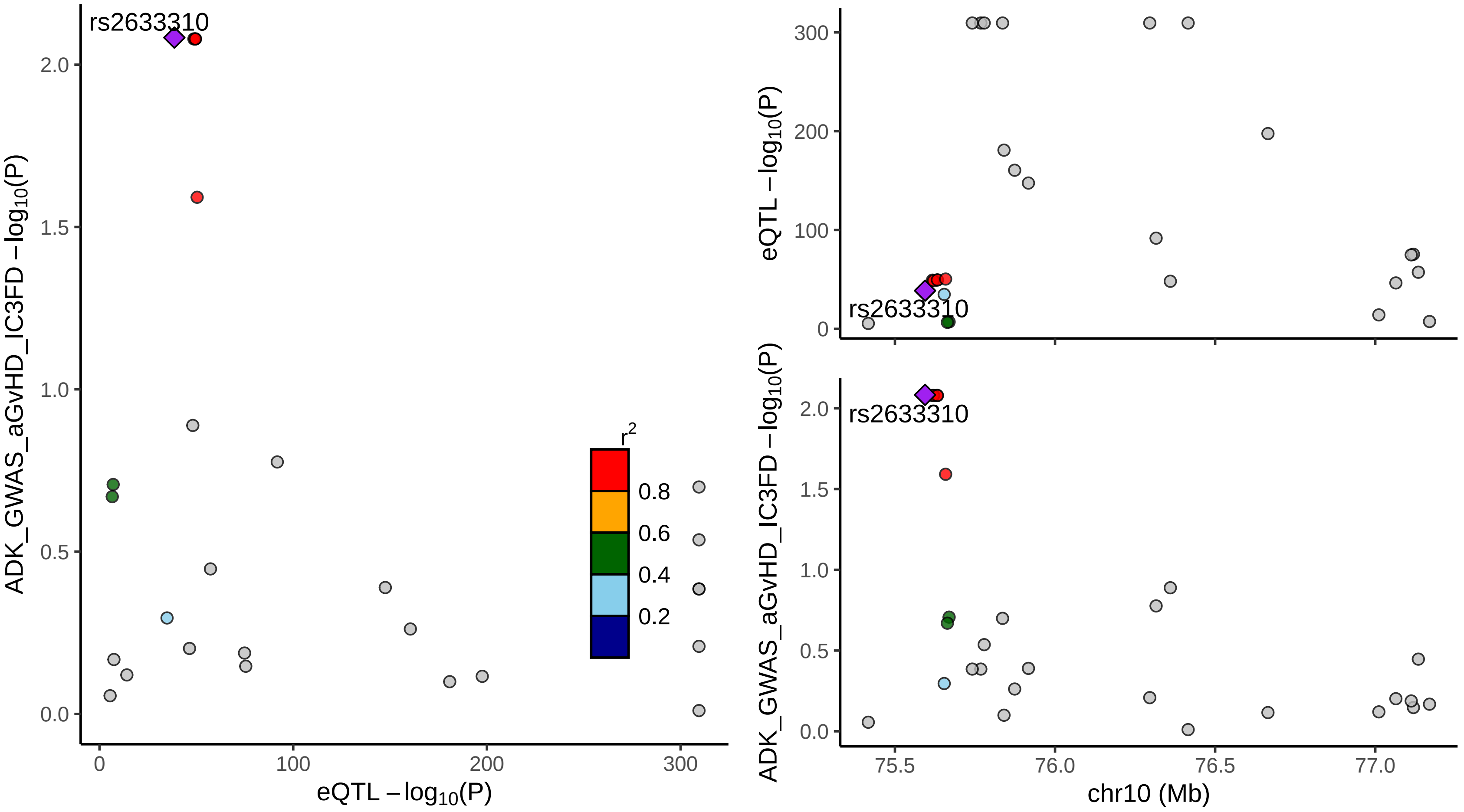

rs2633310
ADK_GWAS_aGvHD_IC3FD −log10(P)
eQTL −log10(P)
r2
0.8
0.6
0.4
0.2
0.0
0.5
1.0
1.5
2.0
0
100
200
300
rs2633310
eQTL −log10(P)
rs2633310
ADK_GWAS_aGvHD_IC3FD −log10(P)
75.5
76.0
76.5
77.0
chr10 (Mb)

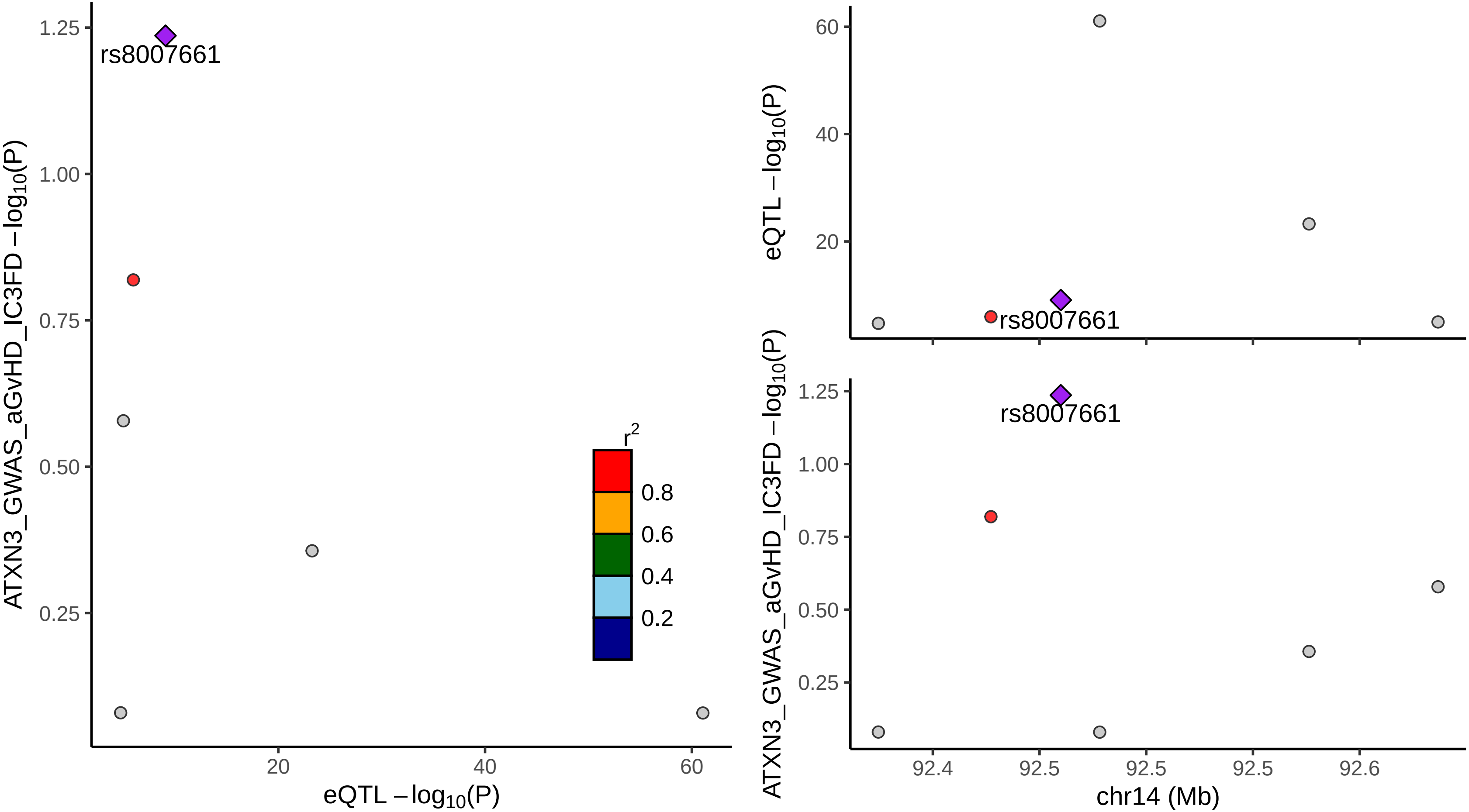

1.25
1.00
0.75
0.50
0.25
ATXN3_GWAS_aGvHD_IC3FD −log10(P)
rs8007661
r2
0.8
0.6
0.4
0.2
20
40
60
eQTL −log10(P)
60
40
20
eQTL −log10(P)
rs8007661
1.25
1.00
0.75
0.50
0.25
ATXN3_GWAS_aGvHD_IC3FD −log10(P)
rs8007661
92.4
92.5
92.5
92.5
92.6
chr14 (Mb)

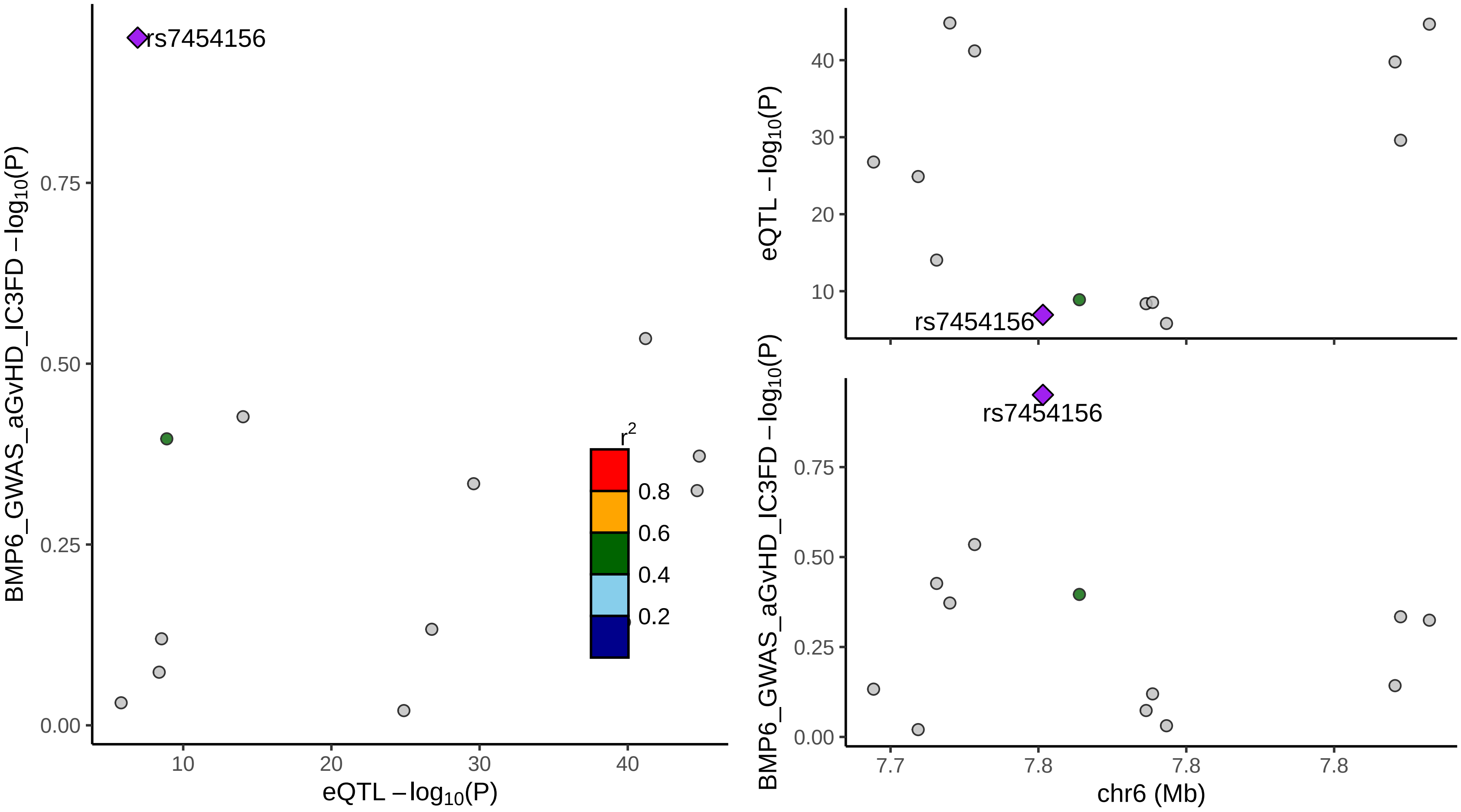
rs7454156
BMP6_GWAS_aGvHD_IC3FD −log10(P)
0.75
0.50
0.25
0.00
10
20
30
40
eQTL −log10(P)
r2
0.8
0.6
0.4
0.2
eQTL −log10(P)
40
30
20
10
rs7454156
BMP6_GWAS_aGvHD_IC3FD −log10(P)
rs7454156
0.75
0.50
0.25
0.00
7.7
7.8
7.8
7.8
chr6 (Mb)

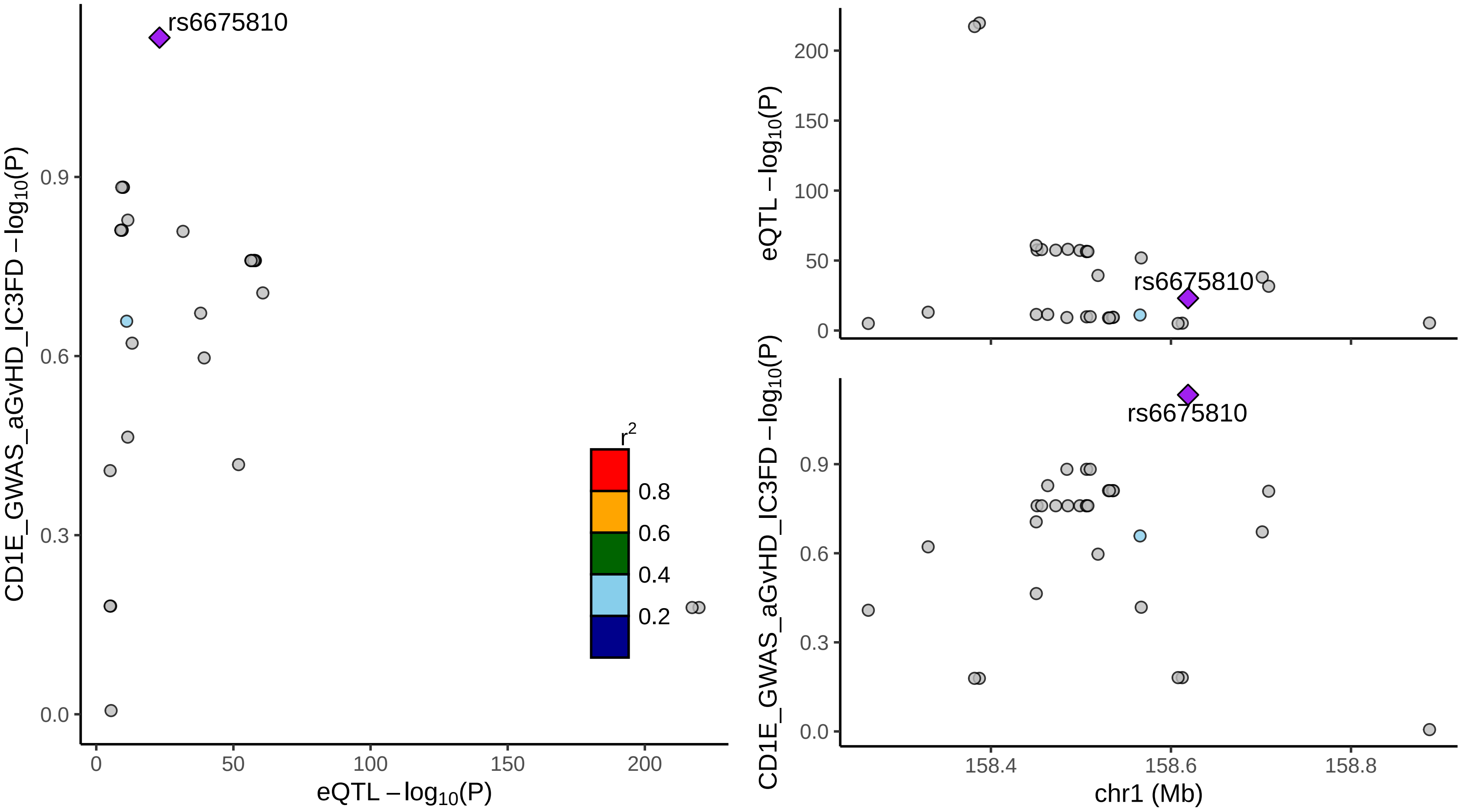

rs6675810
CD1E_GWAS_aGvHD_IC3FD −log10(P)
eQTL −log10(P)
r2
0.8
0.6
0.4
0.2
0.0
0.3
0.6
0.9
0
50
100
150
200
eQTL −log10(P)
CD1E_GWAS_aGvHD_IC3FD −log10(P)
158.4
158.6
158.8
chr1 (Mb)

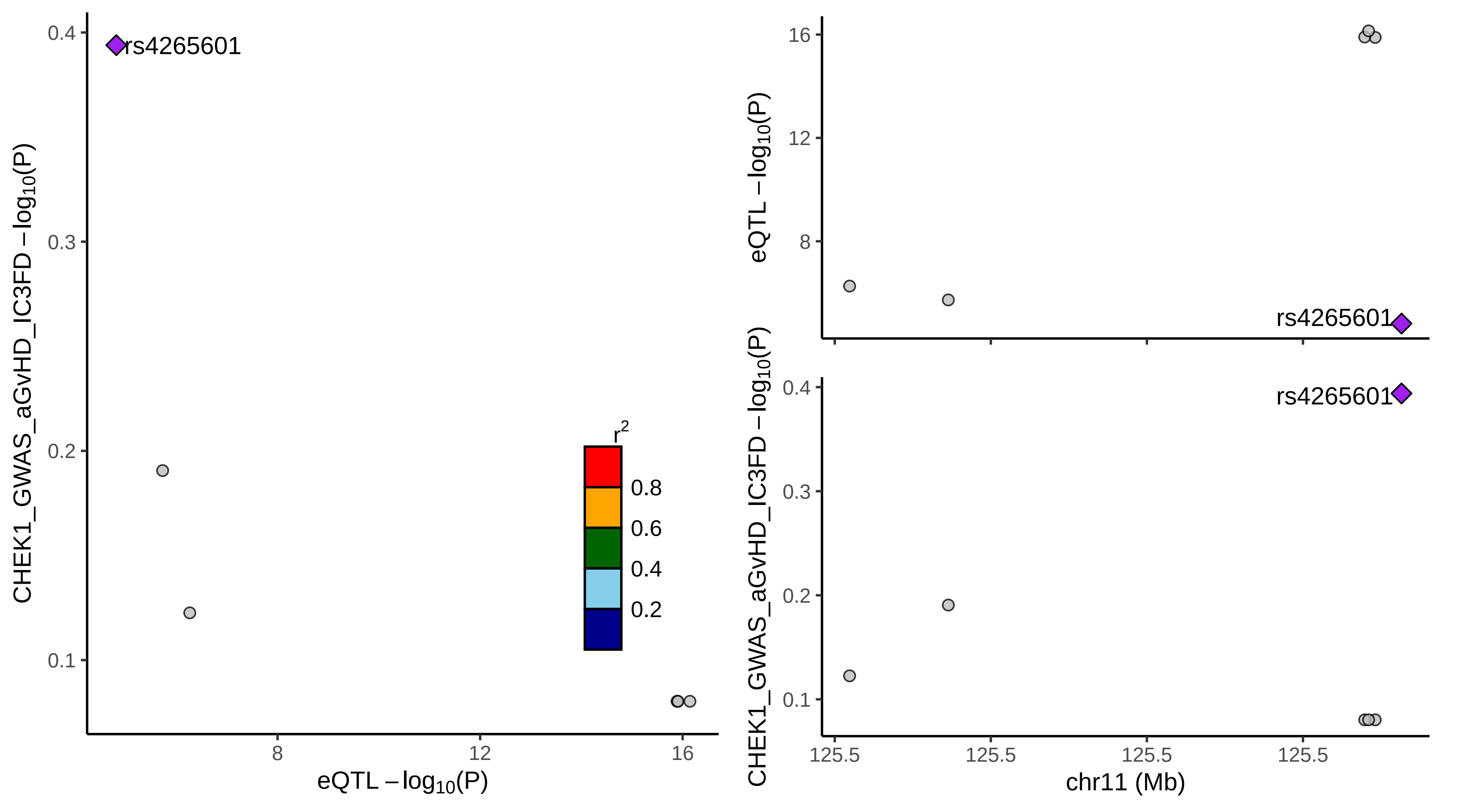
rs4265601
0.4
0.3
0.2
0.1
CHEK1_GWAS_aGvHD_IC3FD −log10(P)
8
12
16
eQTL −log10(P)
r2
0.8
0.6
0.4
0.2
eQTL −log10(P)
rs4265601
rs4265601
125.5
125.5
125.5
125.5
chr11 (Mb)

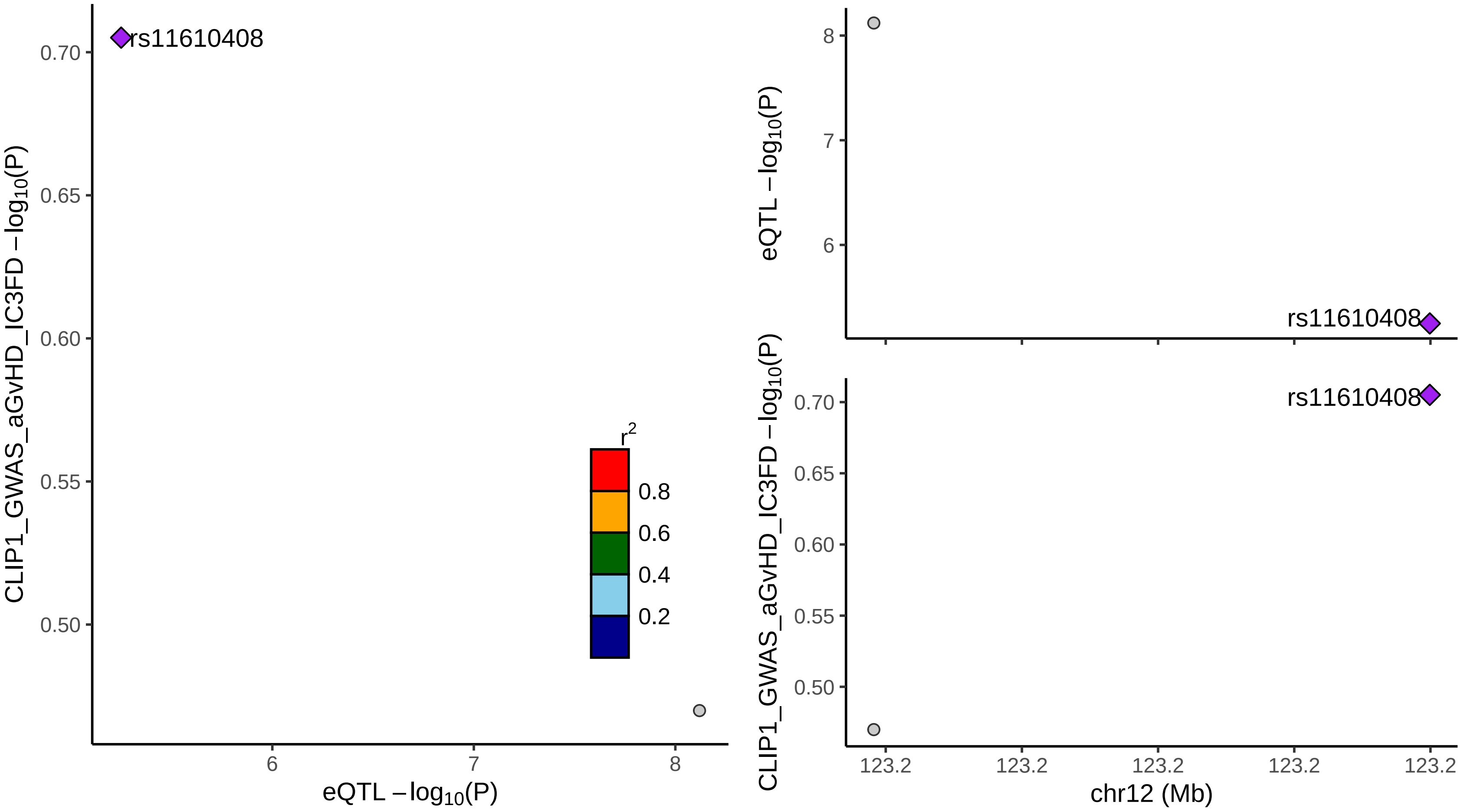
rs11610408
CLIP1_GWAS_aGvHD_IC3FD −log10(P)
0.70
0.65
0.60
0.55
0.50
r2
0.8
0.6
0.4
0.2
6
7
8
eQTL −log10(P)
eQTL −log10(P)
rs11610408
rs11610408
123.2
chr12 (Mb)

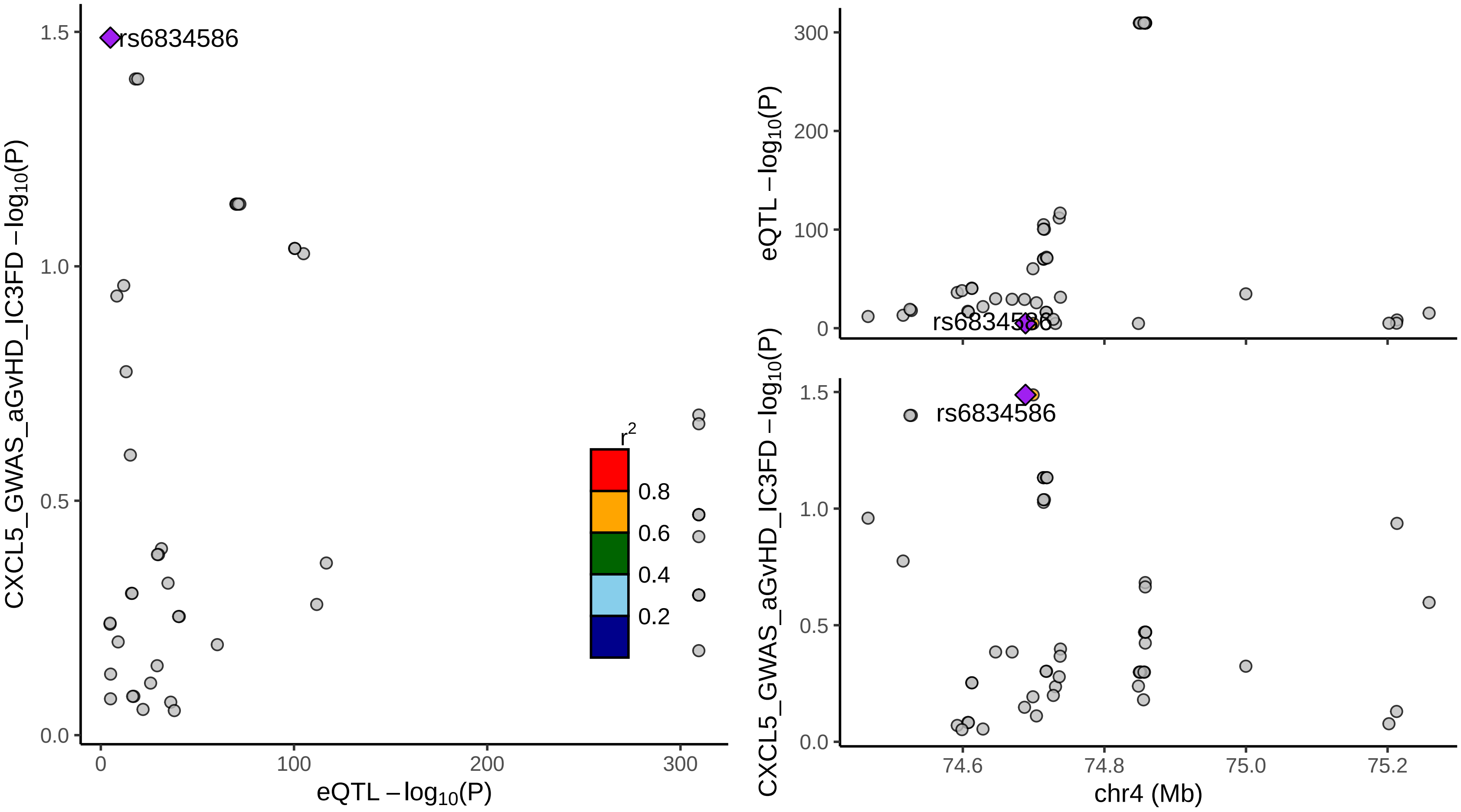

rs6834586
CXCL5_GWAS_aGvHD_IC3FD −log10(P)
eQTL −log10(P)
r2
0.8
0.6
0.4
0.2
rs6834586
eQTL −log10(P)
rs6834586
CXCL5_GWAS_aGvHD_IC3FD −log10(P)
chr4 (Mb)

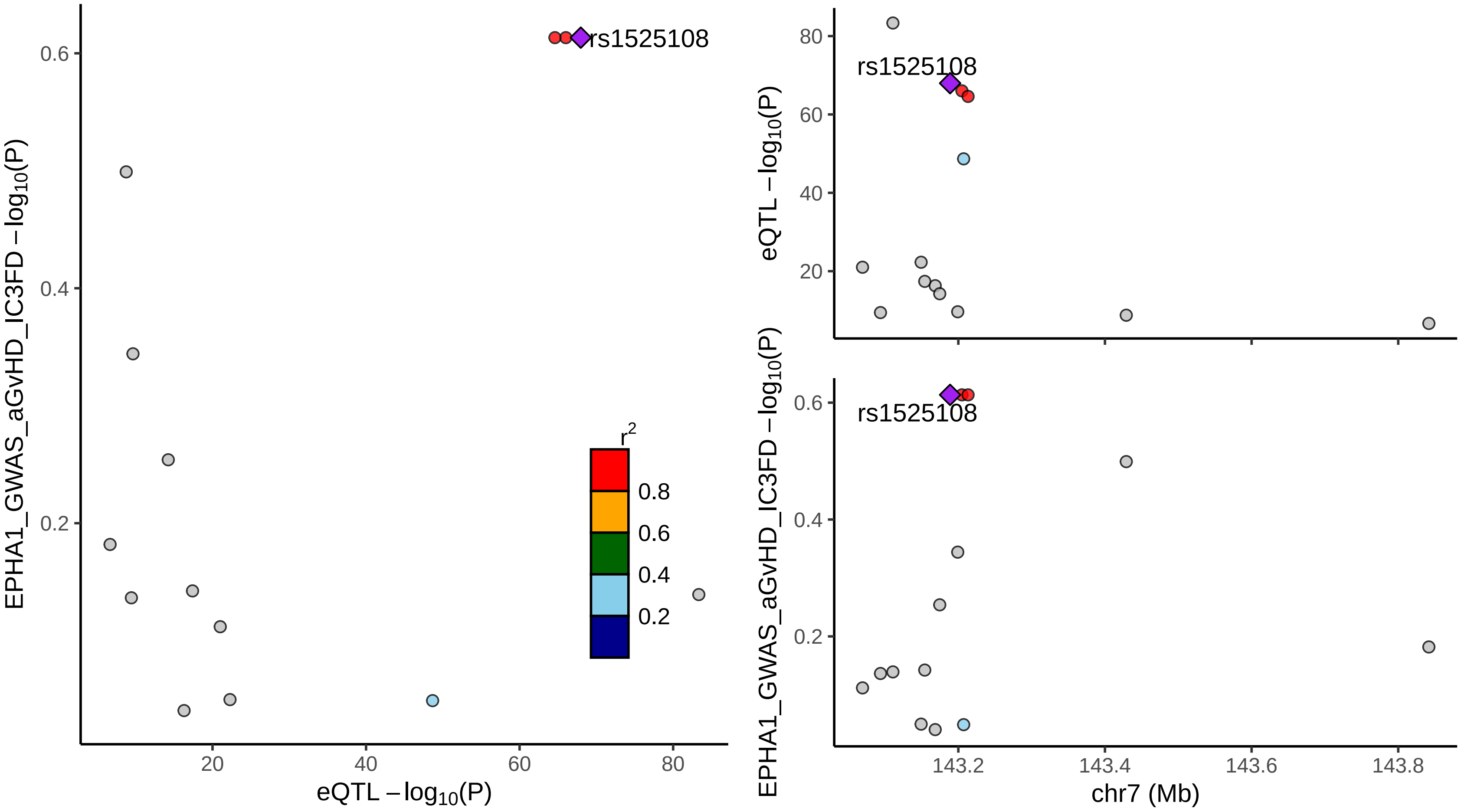

rs1525108
EPHA1_GWAS_aGvHD_IC3FD −log10(P)
0.6
0.4
0.2
20
40
60
80
eQTL −log10(P)
r2
0.8
0.6
0.4
0.2
rs1525108
eQTL −log10(P)
80
60
40
20
rs1525108
EPHA1_GWAS_aGvHD_IC3FD −log10(P)
0.6
0.4
0.2
143.2
143.4
143.6
143.8
chr7 (Mb)

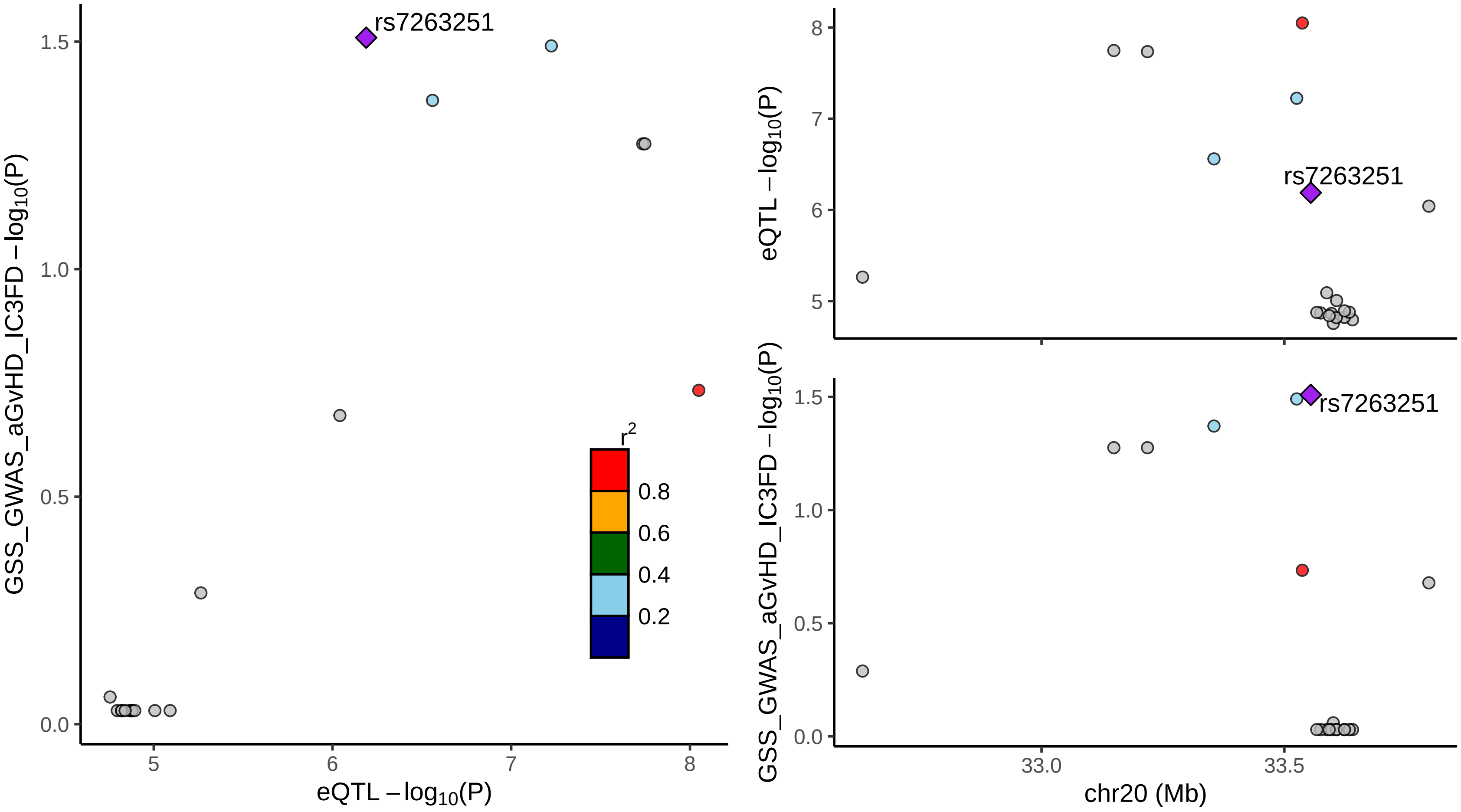

rs7263251
GSS_GWAS_aGvHD_IC3FD −log10(P)
eQTL −log10(P)
r2
0.8
0.6
0.4
0.2
1.5
1.0
0.5
0.0
5
6
7
8
chr20 (Mb)
33.0
33.5

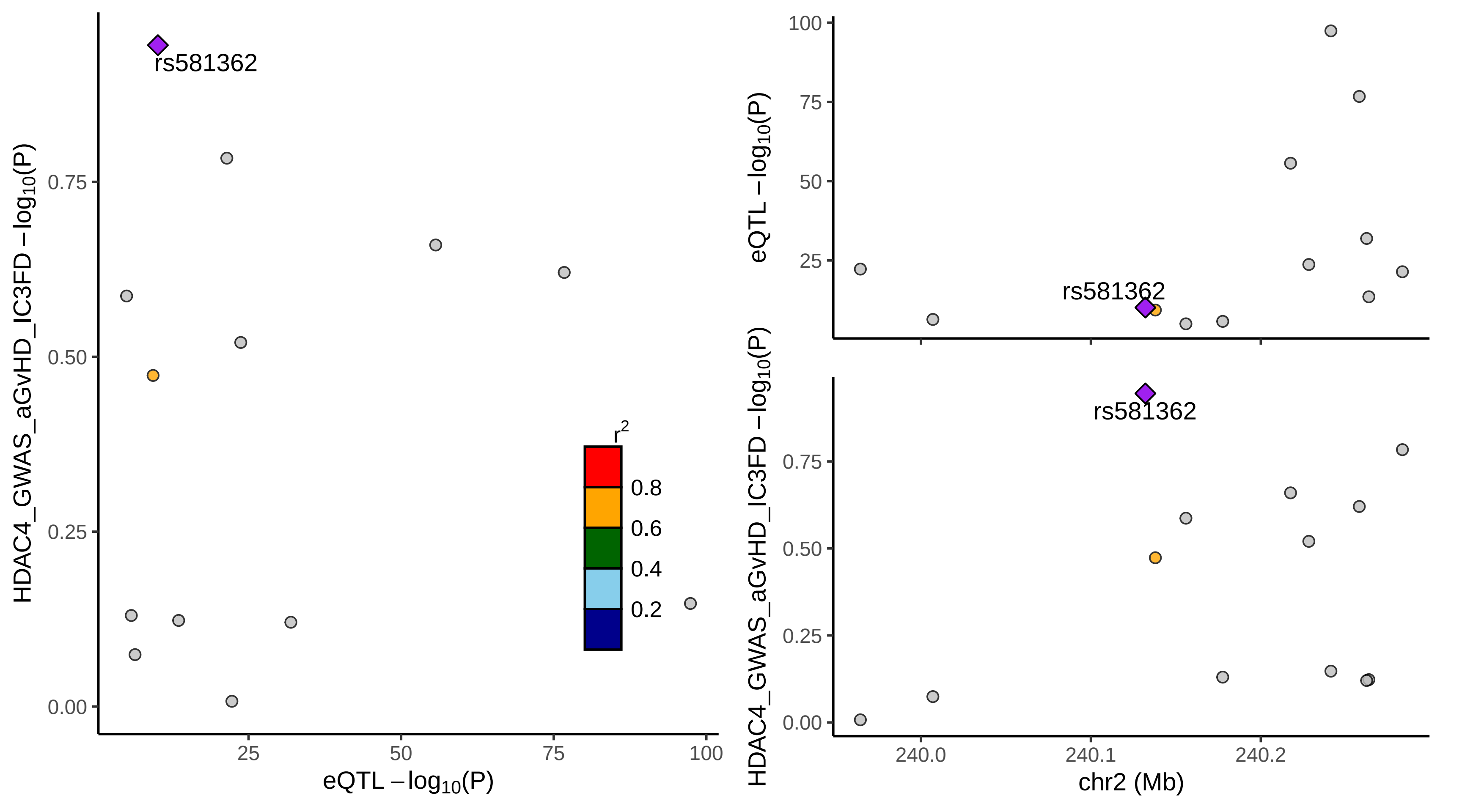
rs581362
HDAC4_GWAS_aGvHD_IC3FD −log10(P)
0.75
0.50
0.25
0.00
25
50
75
100
eQTL −log10(P)
r2
0.8
0.6
0.4
0.2
100
75
50
25
eQTL −log10(P)
rs581362
rs581362
HDAC4_GWAS_aGvHD_IC3FD −log10(P)
0.75
0.50
0.25
0.00
240.0
240.1
240.2
chr2 (Mb)

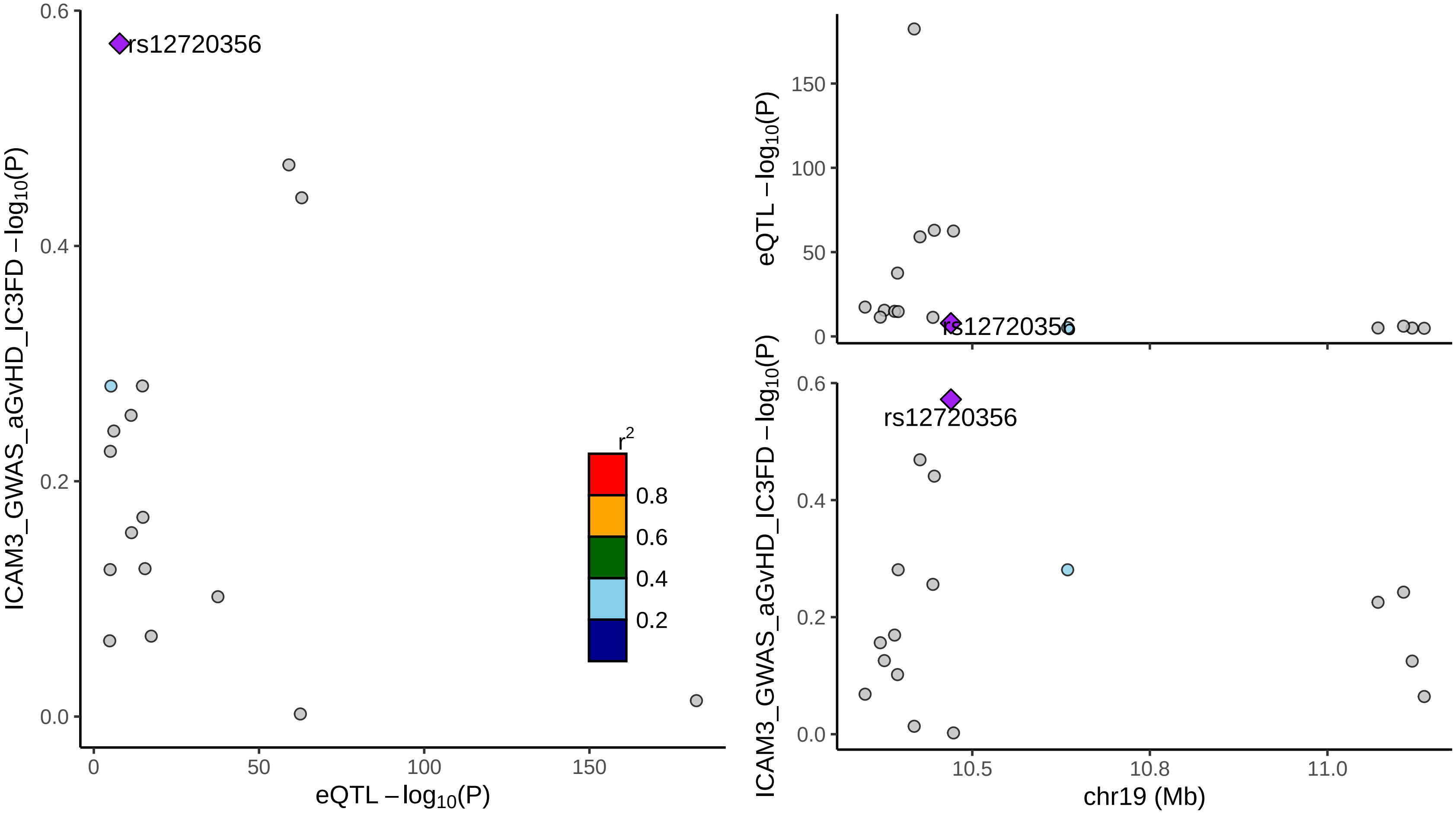

rs12720356
ICAM3_GWAS_aGvHD_IC3FD −log10(P)
0.6
0.4
0.2
0.0
0
50
100
150
eQTL −log10(P)
r2
0.8
0.6
0.4
0.2
rs12720356
eQTL −log10(P)
150
100
50
0
rs12720356
ICAM3_GWAS_aGvHD_IC3FD −log10(P)
0.6
0.4
0.2
0.0
10.5
10.8
11.0
chr19 (Mb)

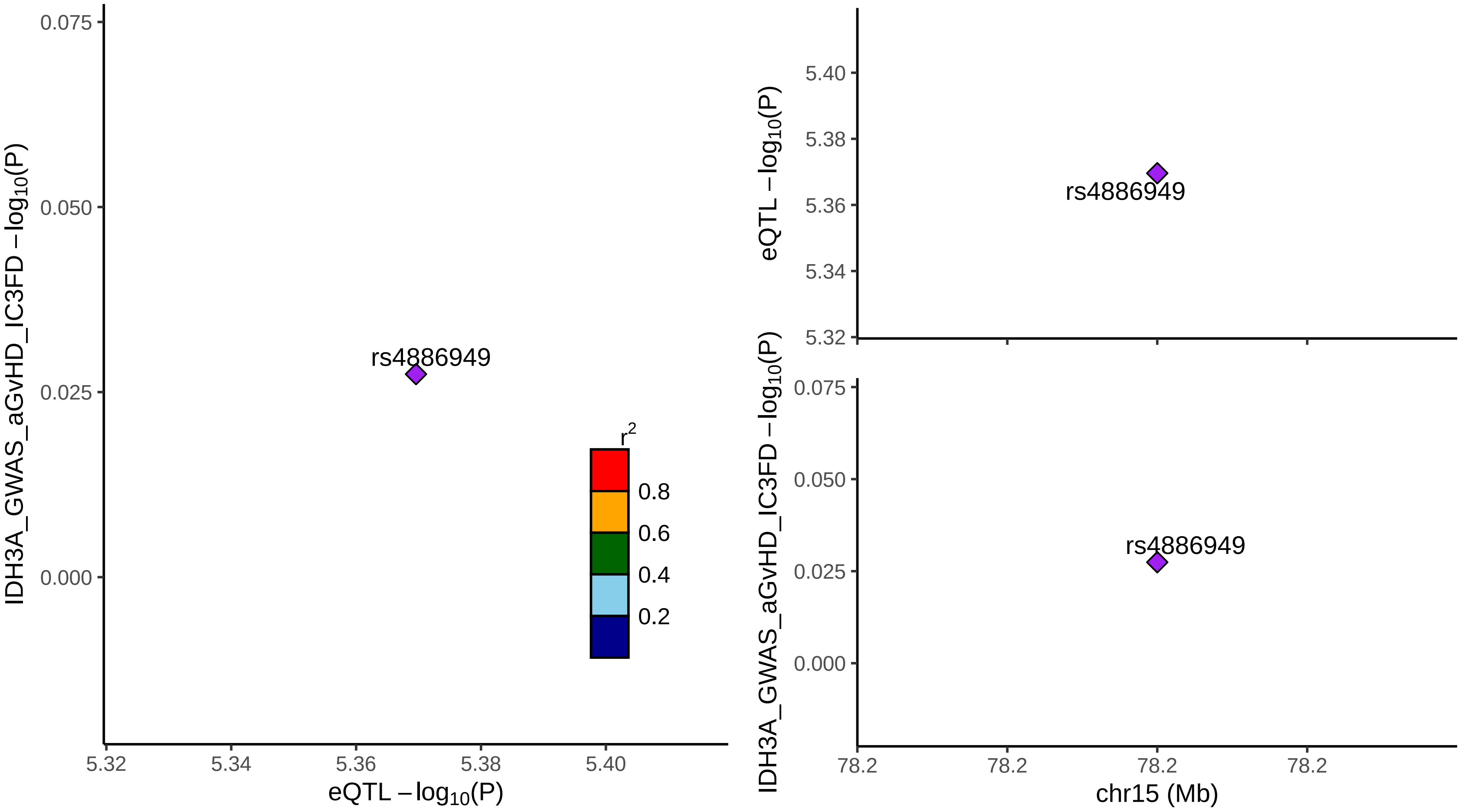
IDH3A_GWAS_aGvHD_IC3FD −log10(P)
0.075
0.050
0.025
0.000
rs4886949
r2
0.8
0.6
0.4
0.2
5.32
5.34
5.36
5.38
5.40
eQTL −log10(P)
eQTL −log10(P)
5.40
5.38
5.36
5.34
5.32
rs4886949
IDH3A_GWAS_aGvHD_IC3FD −log10(P)
0.075
0.050
0.025
0.000
rs4886949
78.2
78.2
78.2
78.2
chr15 (Mb)

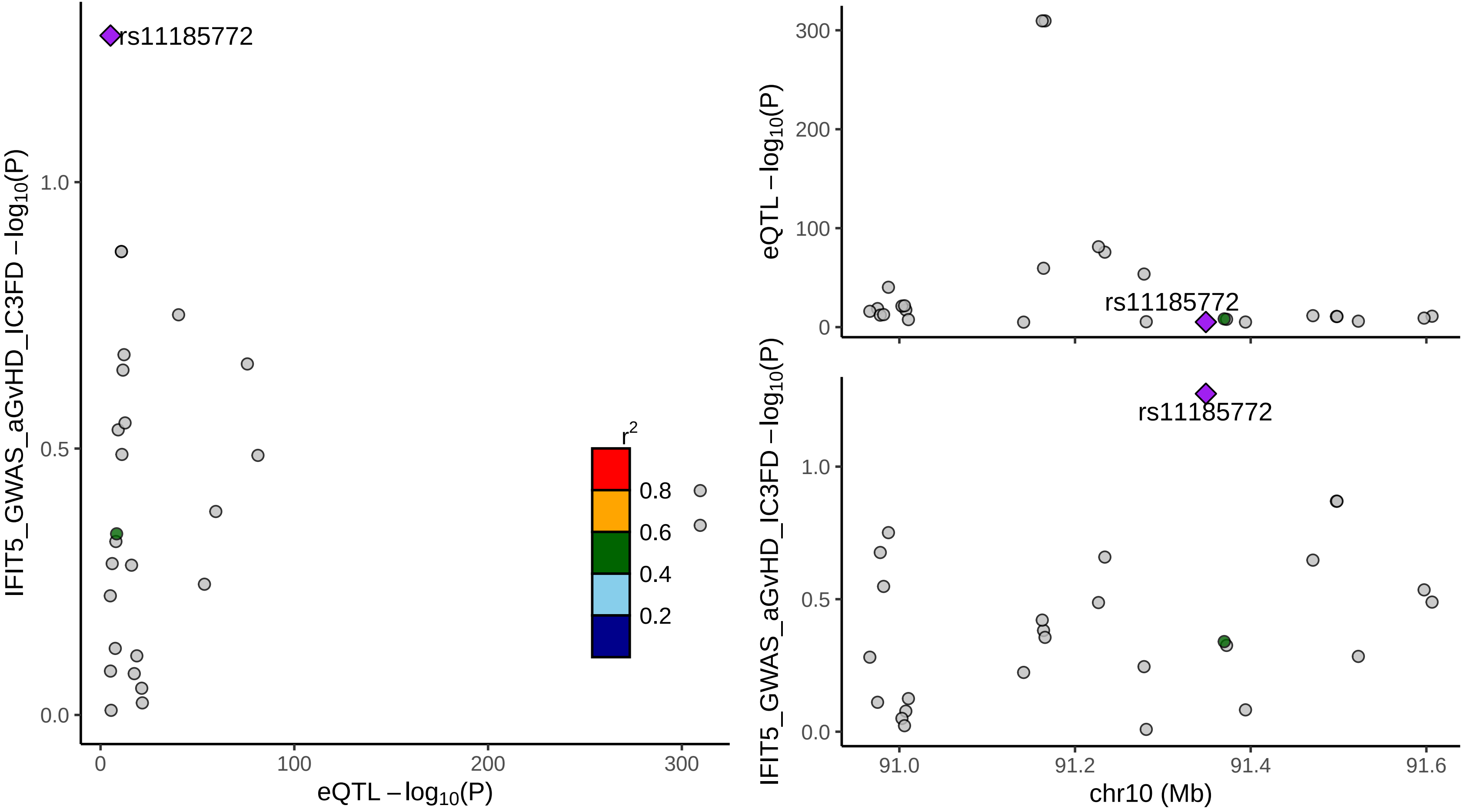

rs11185772
IFIT5_GWAS_aGvHD_IC3FD −log10(P)
eQTL −log10(P)
r2
0.8
0.6
0.4
0.2
0.0
0.5
1.0
0
100
200
300
rs11185772
rs11185772
chr10 (Mb)
91.0
91.2
91.4
91.6

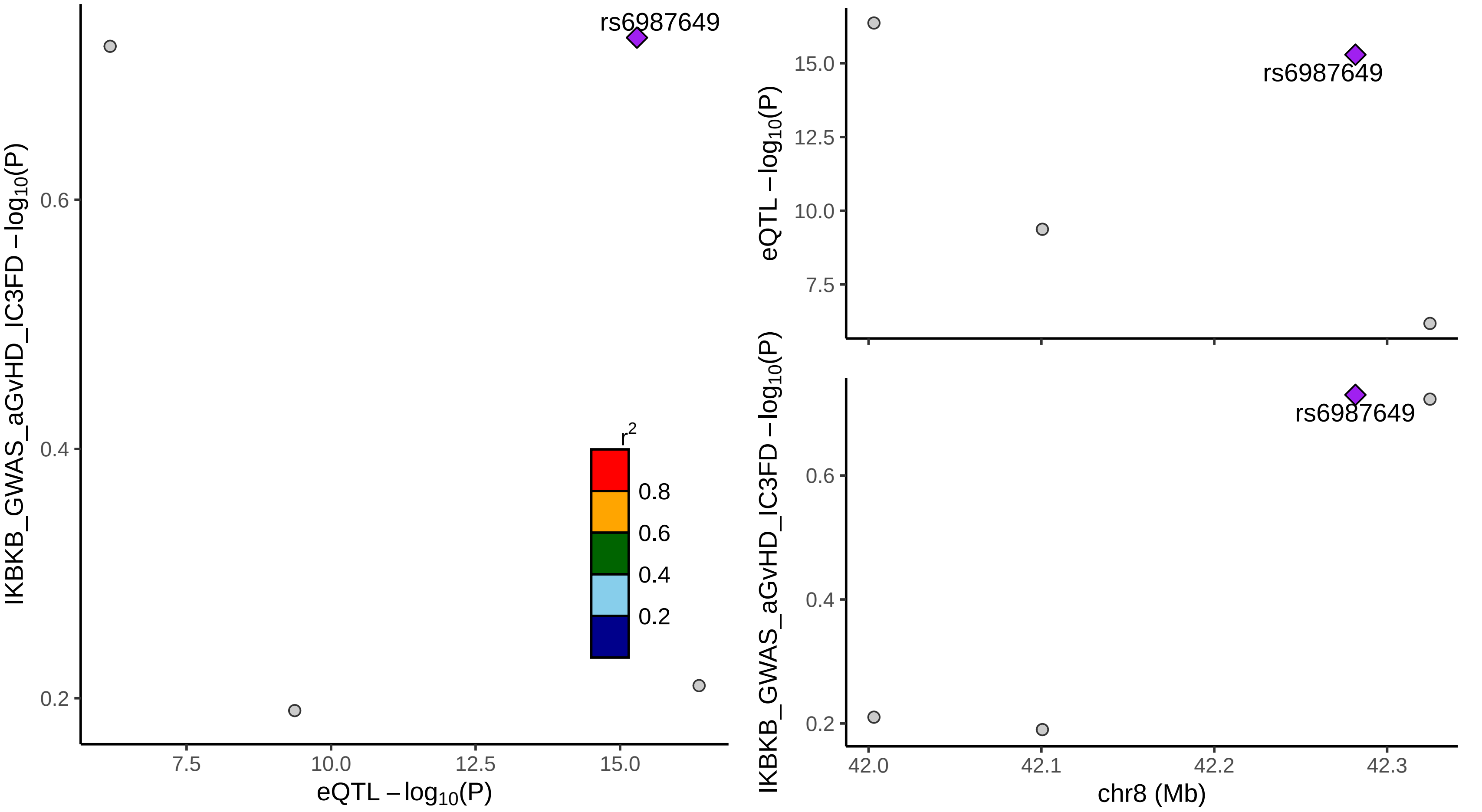
rs6987649
IKBKB_GWAS_aGvHD_IC3FD −log10(P)
0.6
0.4
0.2
7.5
10.0
12.5
15.0
eQTL −log10(P)
r2
0.8
0.6
0.4
0.2
rs6987649
eQTL −log10(P)
15.0
12.5
10.0
7.5
rs6987649
IKBKB_GWAS_aGvHD_IC3FD −log10(P)
0.6
0.4
0.2
42.0
42.1
42.2
42.3
chr8 (Mb)

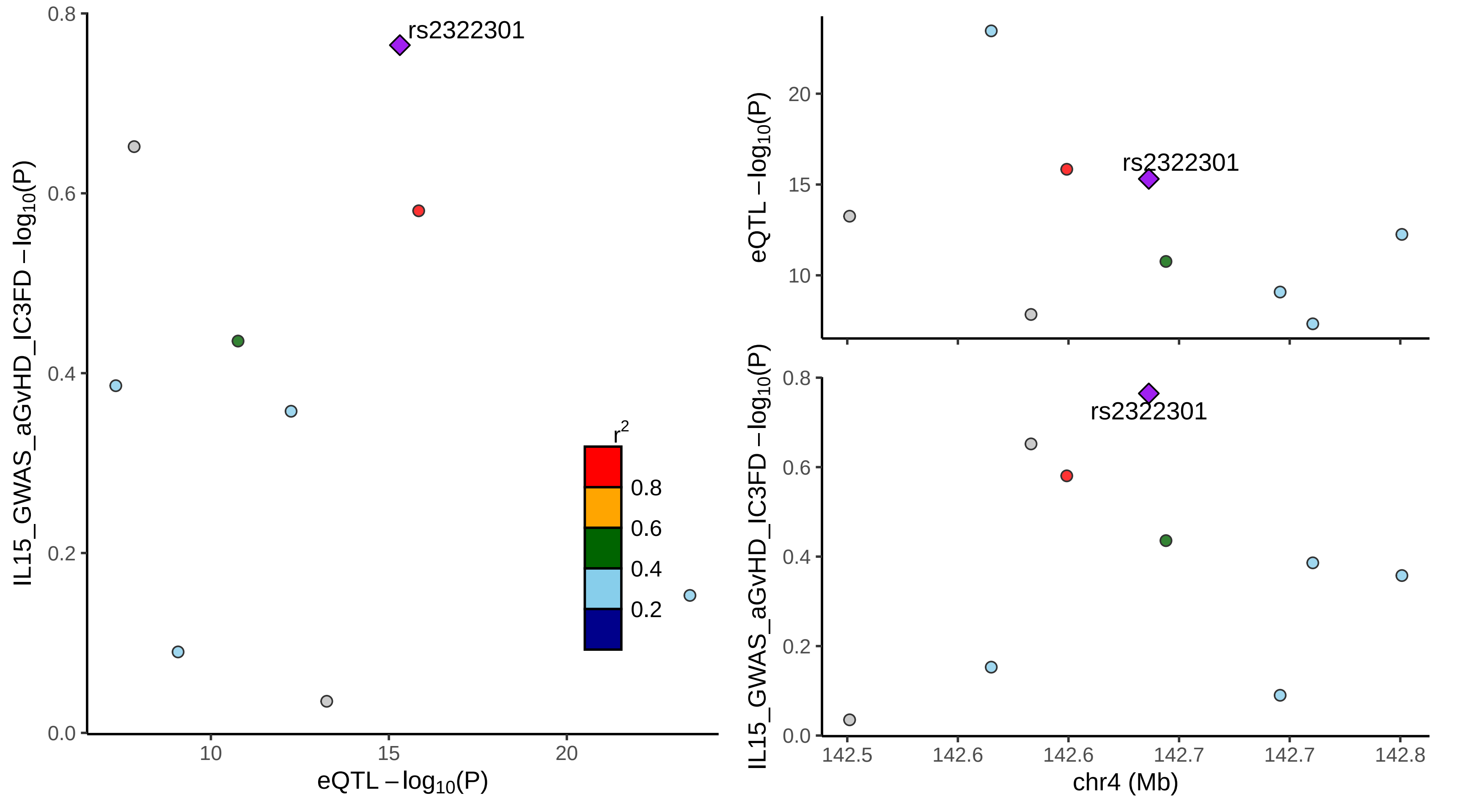

rs2322301
IL15_GWAS_aGvHD_IC3FD −log10(P)
eQTL −log10(P)
r2
0.8
0.6
0.4
0.2
eQTL −log10(P)
rs2322301
rs2322301
IL15_GWAS_aGvHD_IC3FD −log10(P)
chr4 (Mb)

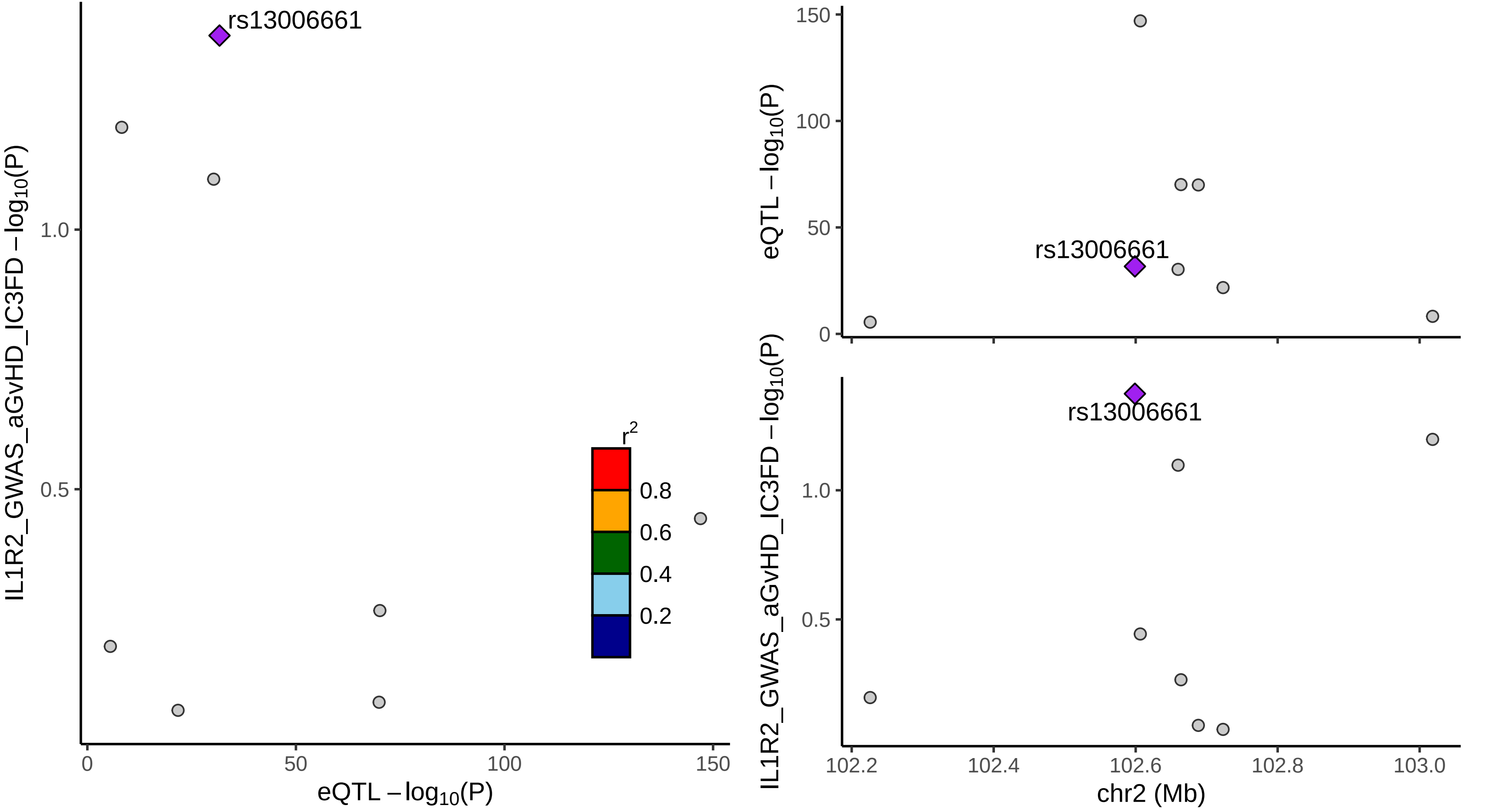

rs13006661
IL1R2_GWAS_aGvHD_IC3FD −log10(P)
1.0
0.5
r2
0.8
0.6
0.4
0.2
0
50
100
150
eQTL −log10(P)
eQTL −log10(P)
chr2 (Mb)
102.2
102.4
102.6
102.8
103.0

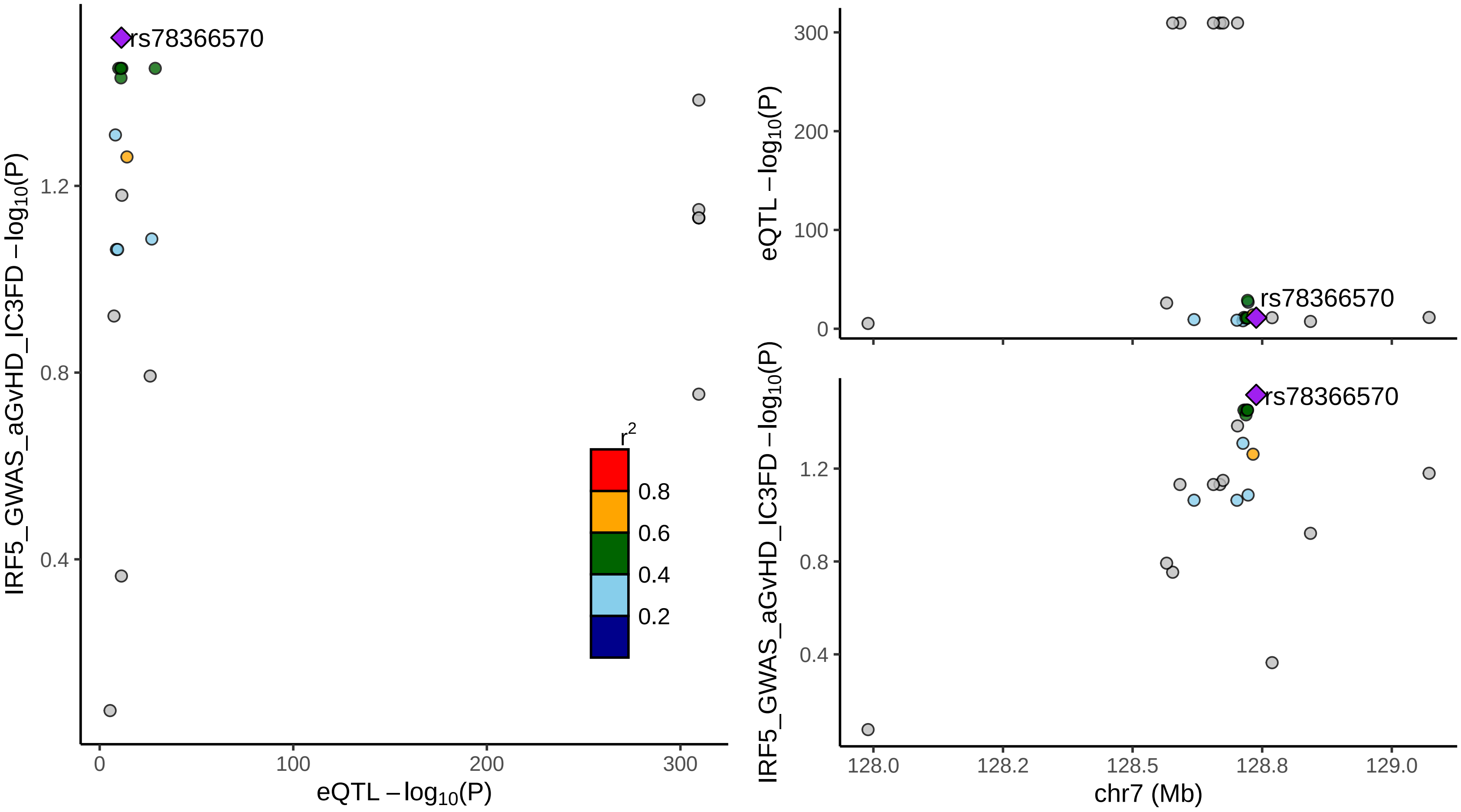

rs78366570
IRF5_GWAS_aGvHD_IC3FD −log10(P)
1.2
0.8
0.4
0
100
200
300
eQTL −log10(P)
r2
0.8
0.6
0.4
0.2
eQTL −log10(P)
300
200
100
0
rs78366570
IRF5_GWAS_aGvHD_IC3FD −log10(P)
rs78366570
1.2
0.8
0.4
128.0
128.2
128.5
128.8
129.0
chr7 (Mb)

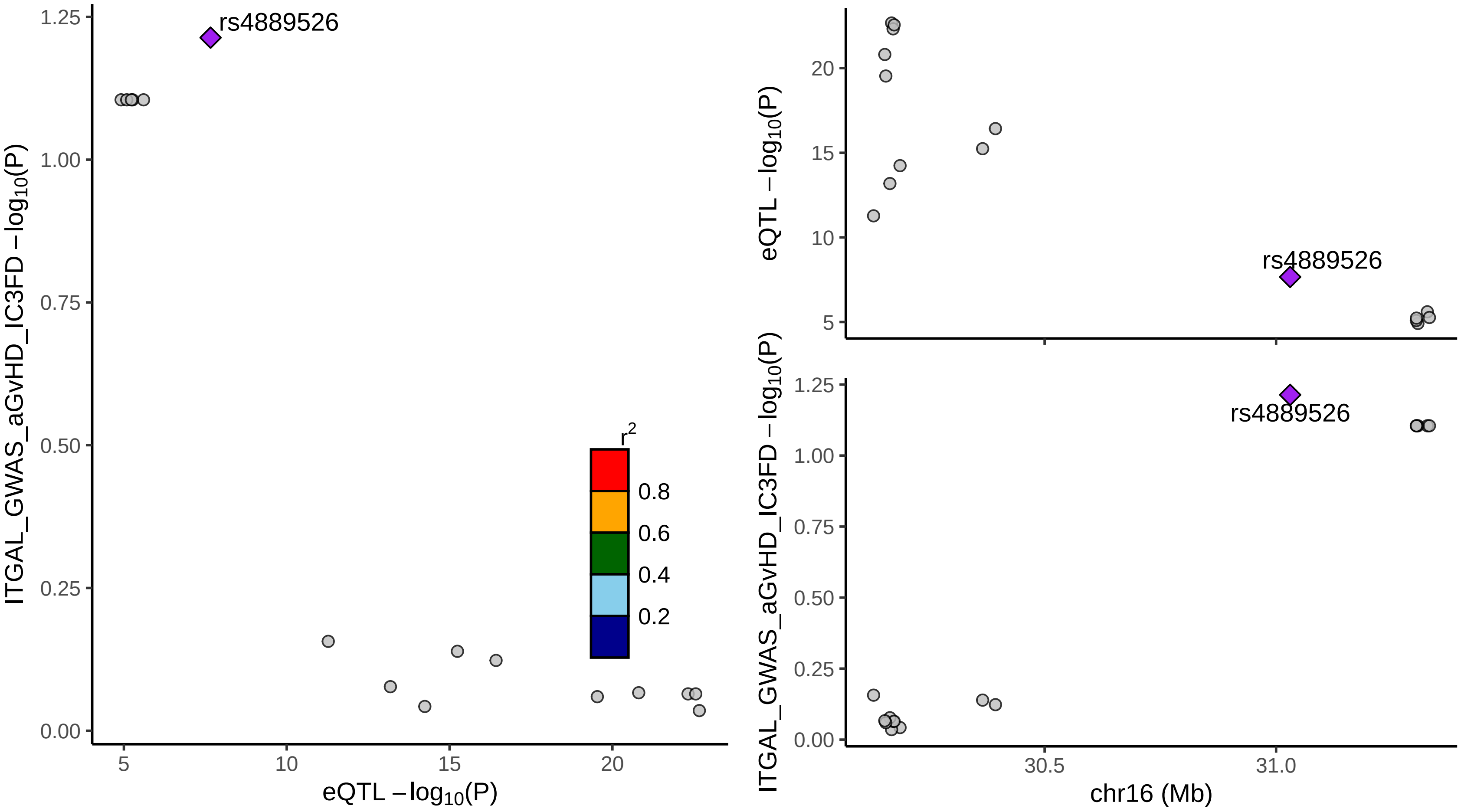

rs4889526
ITGAL_GWAS_aGvHD_IC3FD $-\log_{10}(P)$
1.25
1.00
0.75
0.50
0.25
0.00
5
10
15
20
eQTL $-\log_{10}(P)$
r^2
0.8
0.6
0.4
0.2
eQTL $-\log_{10}(P)$
20
15
10
5
rs4889526
ITGAL_GWAS_aGvHD_IC3FD $-\log_{10}(P)$
1.25
1.00
0.75
0.50
0.25
0.00
rs4889526
30.5
31.0
chr16 (Mb)

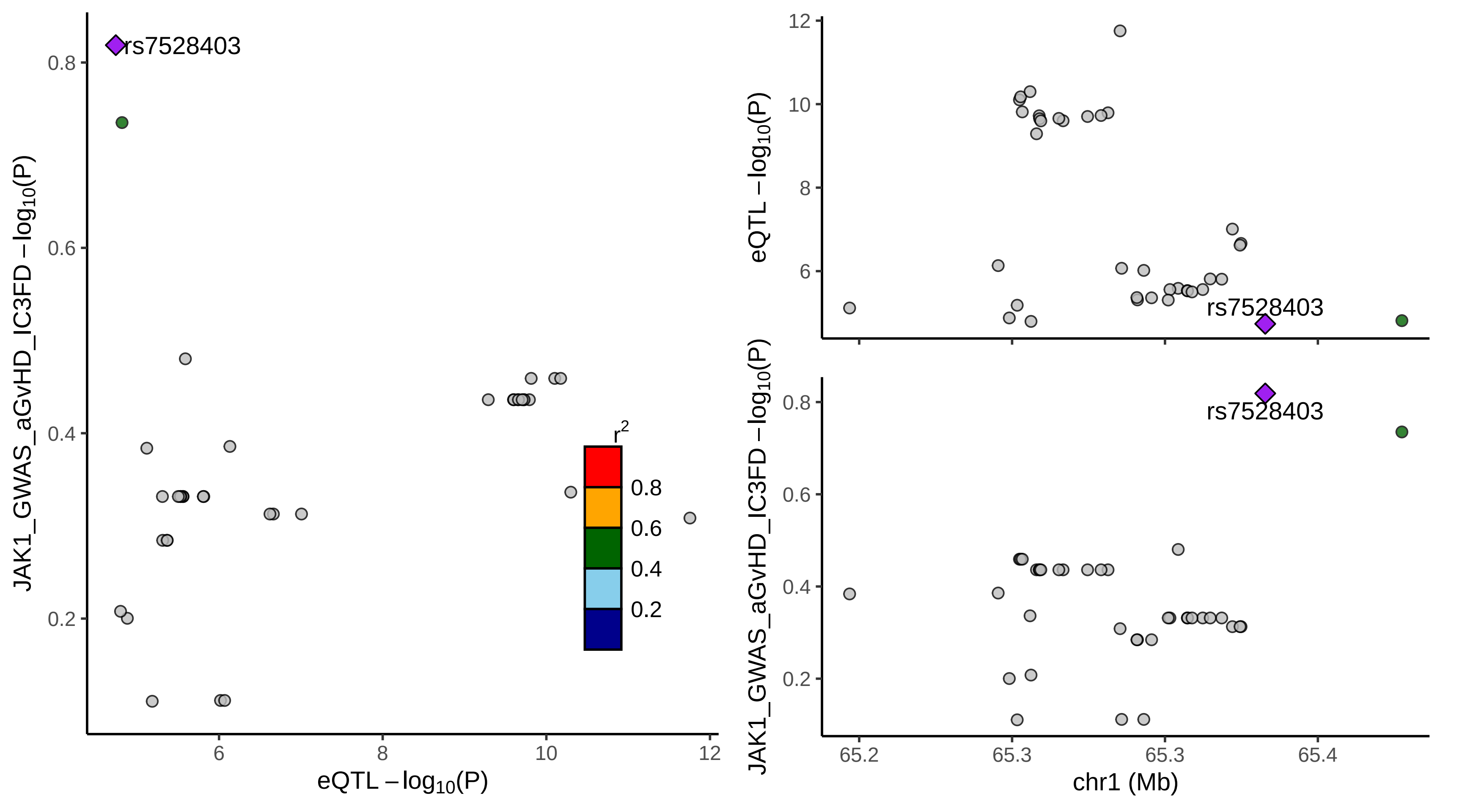

rs7528403
JAK1_GWAS_aGvHD_IC3FD −log10(P)
eQTL −log10(P)
r2
0.8
0.6
0.4
0.2
rs7528403
eQTL −log10(P)
rs7528403
JAK1_GWAS_aGvHD_IC3FD −log10(P)
chr1 (Mb)

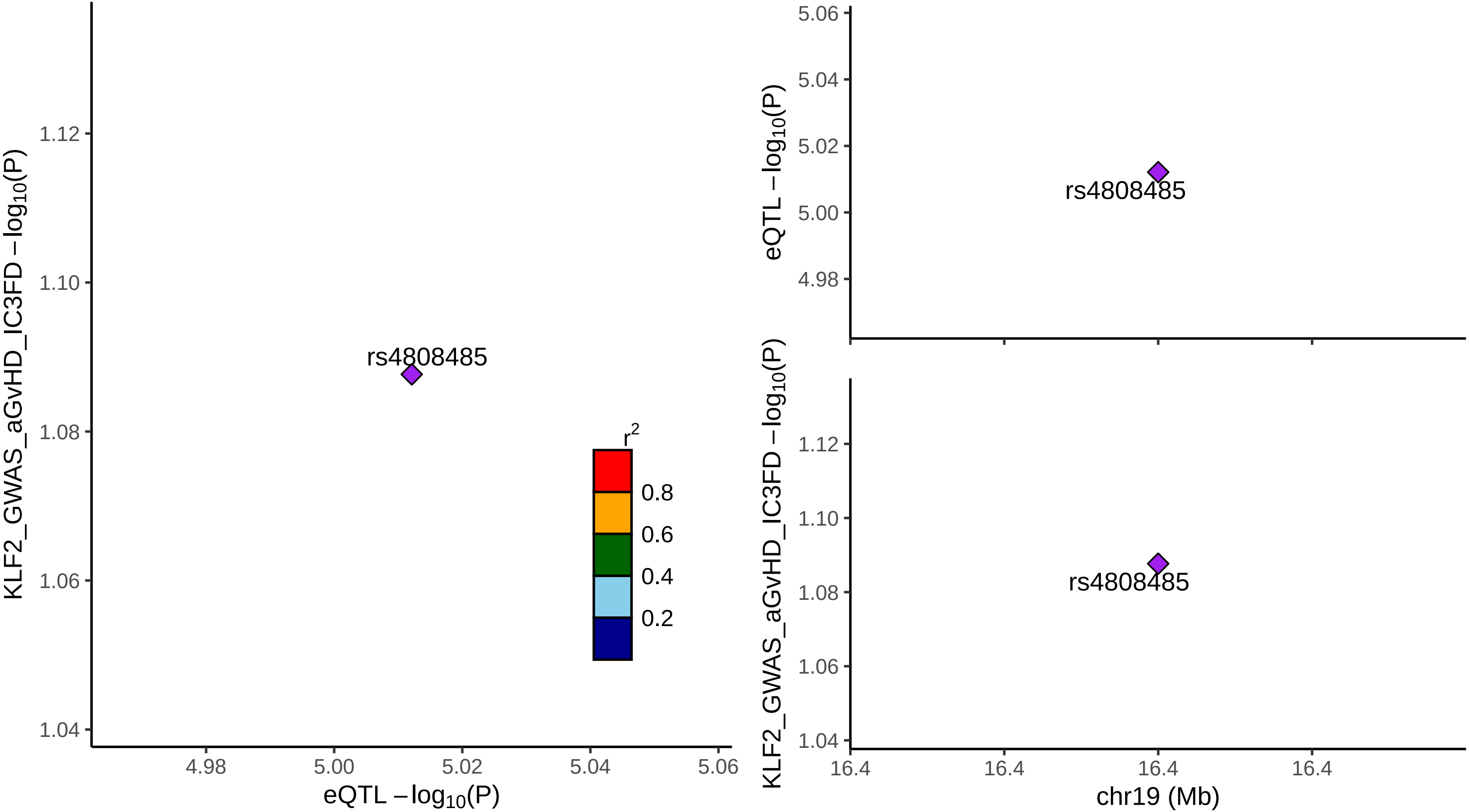

KLF2_GWAS_aGvHD_IC3FD −log10(P)
1.12
1.10
1.08
1.06
1.04
rs4808485
r2
0.8
0.6
0.4
0.2
4.98
5.00
5.02
5.04
5.06
eQTL −log10(P)
5.06
5.04
5.02
5.00
4.98
rs4808485
eQTL −log10(P)
KLF2_GWAS_aGvHD_IC3FD −log10(P)
1.12
1.10
1.08
1.06
1.04
rs4808485
16.4
16.4
16.4
16.4
chr19 (Mb)

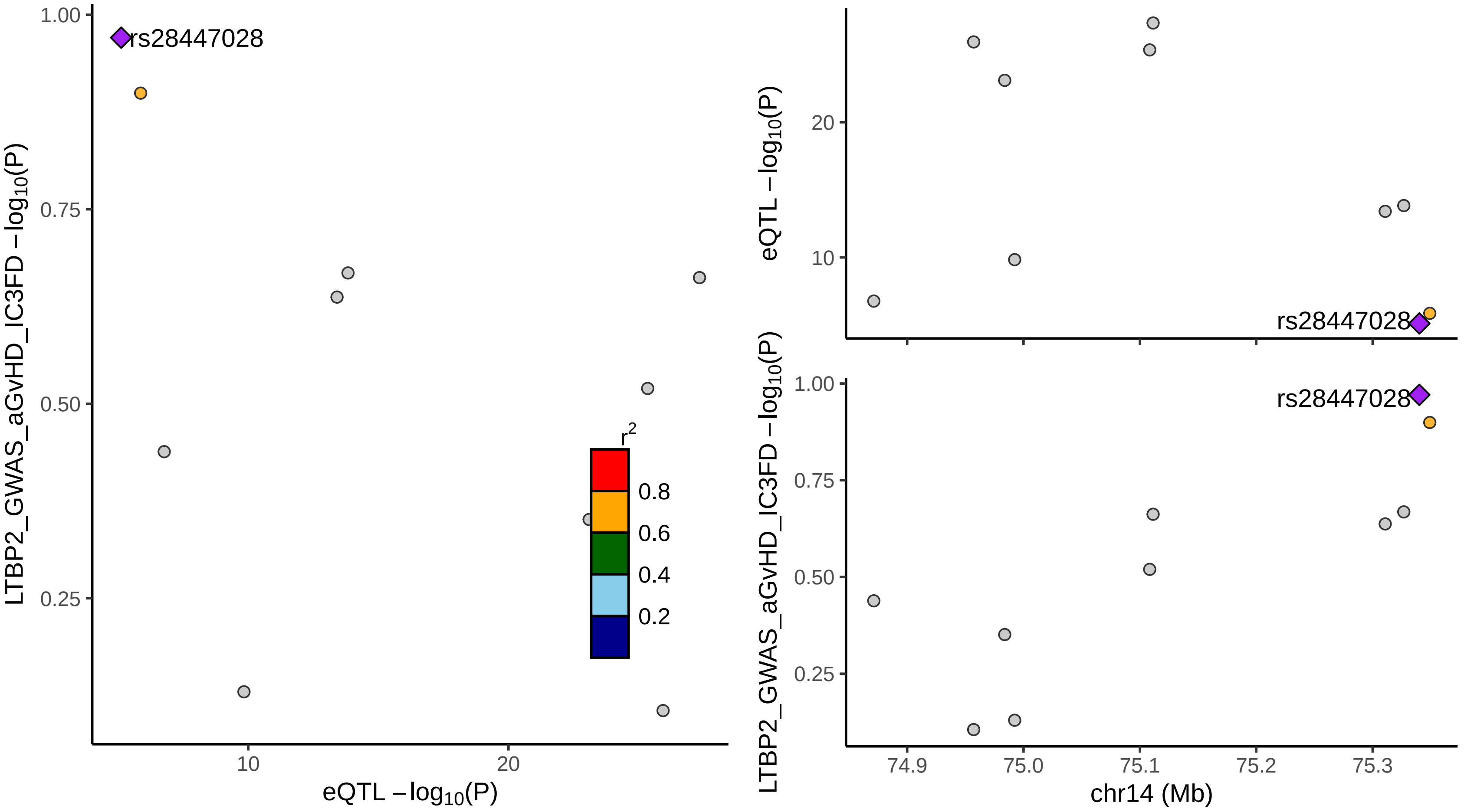

rs28447028
LTBP2_GWAS_aGvHD_IC3FD −log10(P)
1.00
0.75
0.50
0.25
10
20
eQTL −log10(P)
r2
0.8
0.6
0.4
0.2
eQTL −log10(P)
20
10
rs28447028
LTBP2_GWAS_aGvHD_IC3FD −log10(P)
rs28447028
1.00
0.75
0.50
0.25
74.9
75.0
75.1
75.2
75.3
chr14 (Mb)

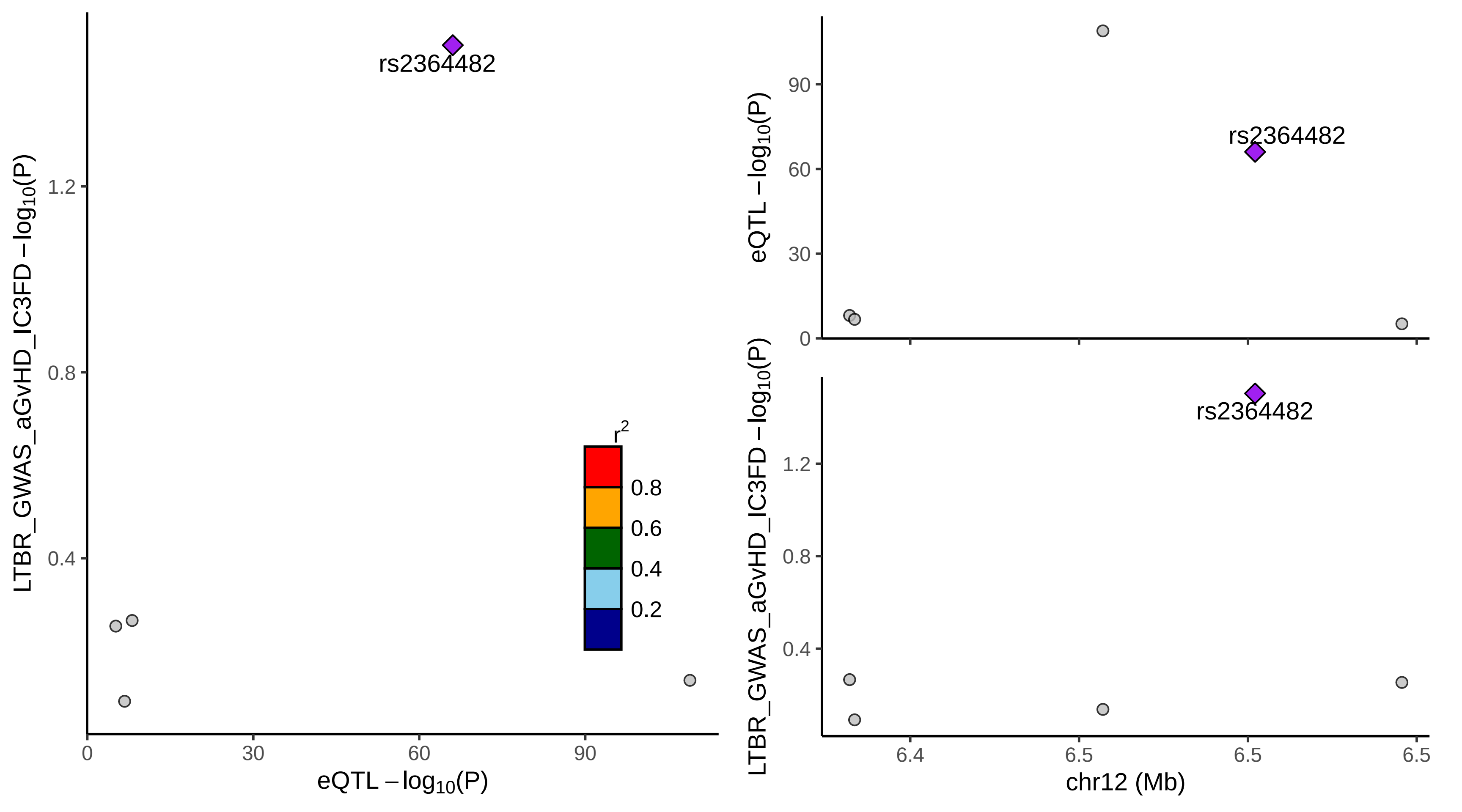
rs2364482
LTBR_GWAS_aGvHD_IC3FD −log10(P)
1.2
0.8
0.4
0
30
60
90
eQTL −log10(P)
r2
0.8
0.6
0.4
0.2
eQTL −log10(P)
90
60
30
0
rs2364482
rs2364482
LTBR_GWAS_aGvHD_IC3FD −log10(P)
1.2
0.8
0.4
6.4
6.5
6.5
6.5
chr12 (Mb)

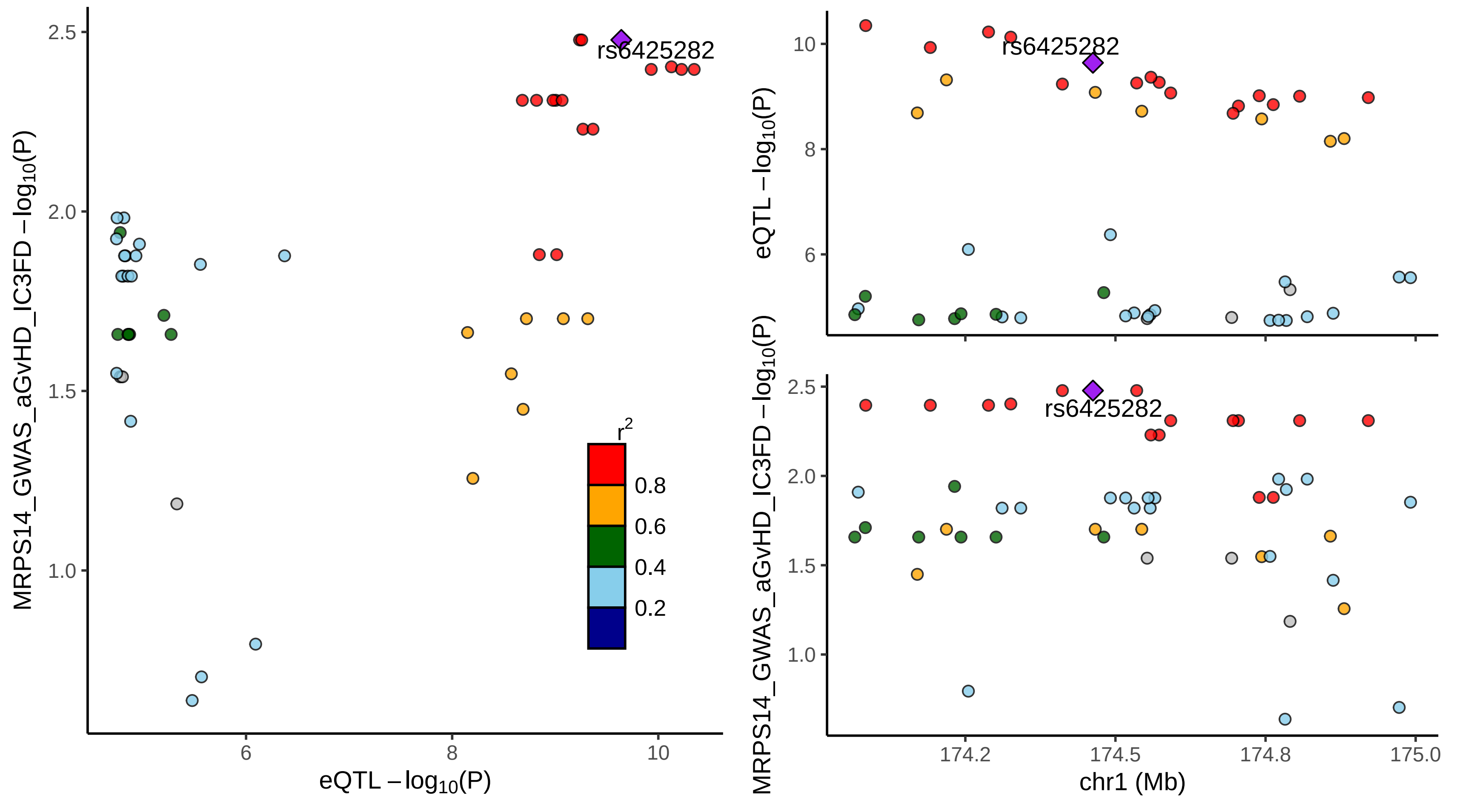

rs6425282
MRPS14_GWAS_aGvHD_IC3FD −log10(P)
eQTL −log10(P)
r2
0.8
0.6
0.4
0.2
chr1 (Mb)

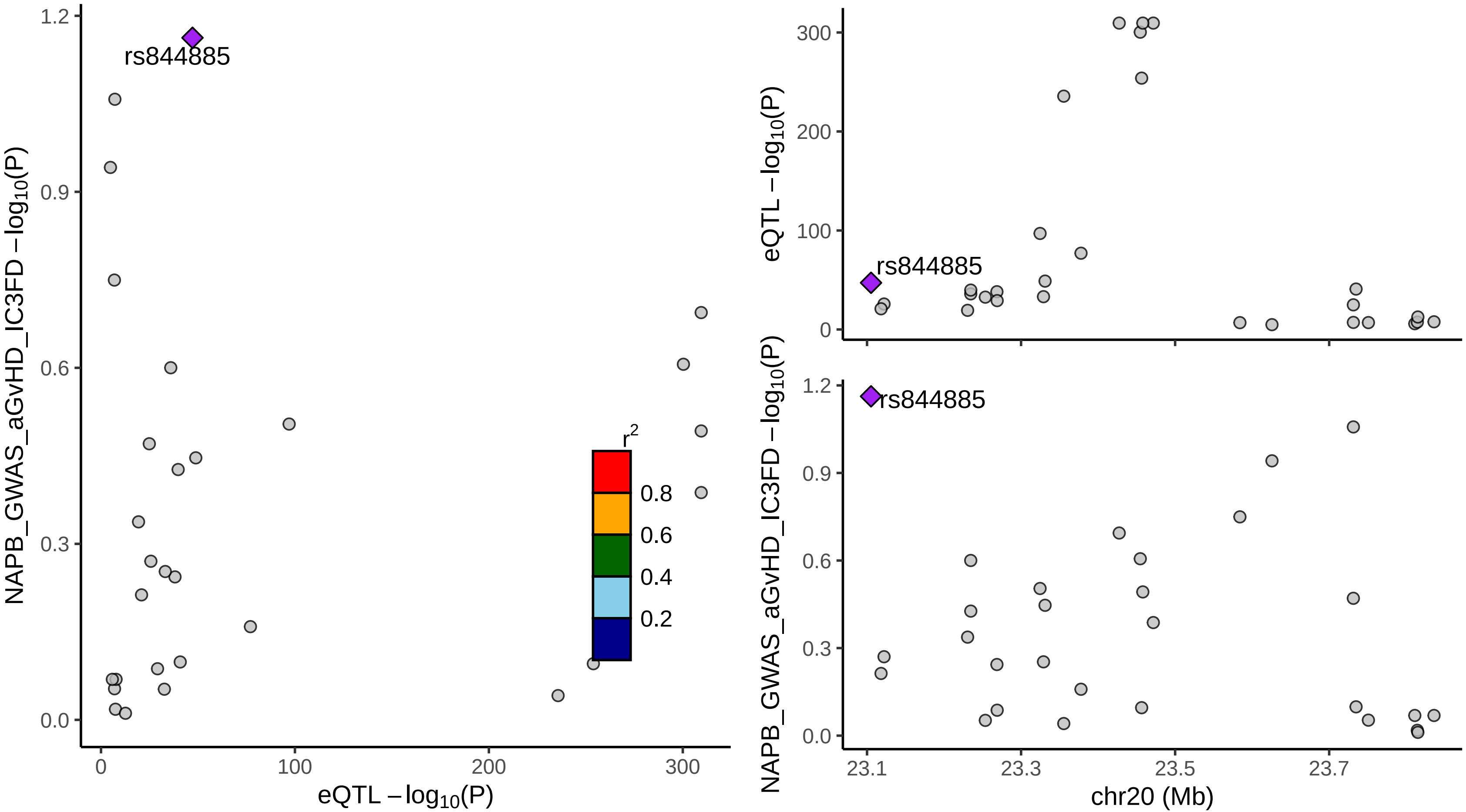
rs844885
NAPB_GWAS_aGvHD_IC3FD −log10(P)
eQTL −log10(P)
r2
0.8
0.6
0.4
0.2
chr20 (Mb)
23.1
23.3
23.5
23.7
0.0
0.3
0.6
0.9
1.2
0
100
200
300

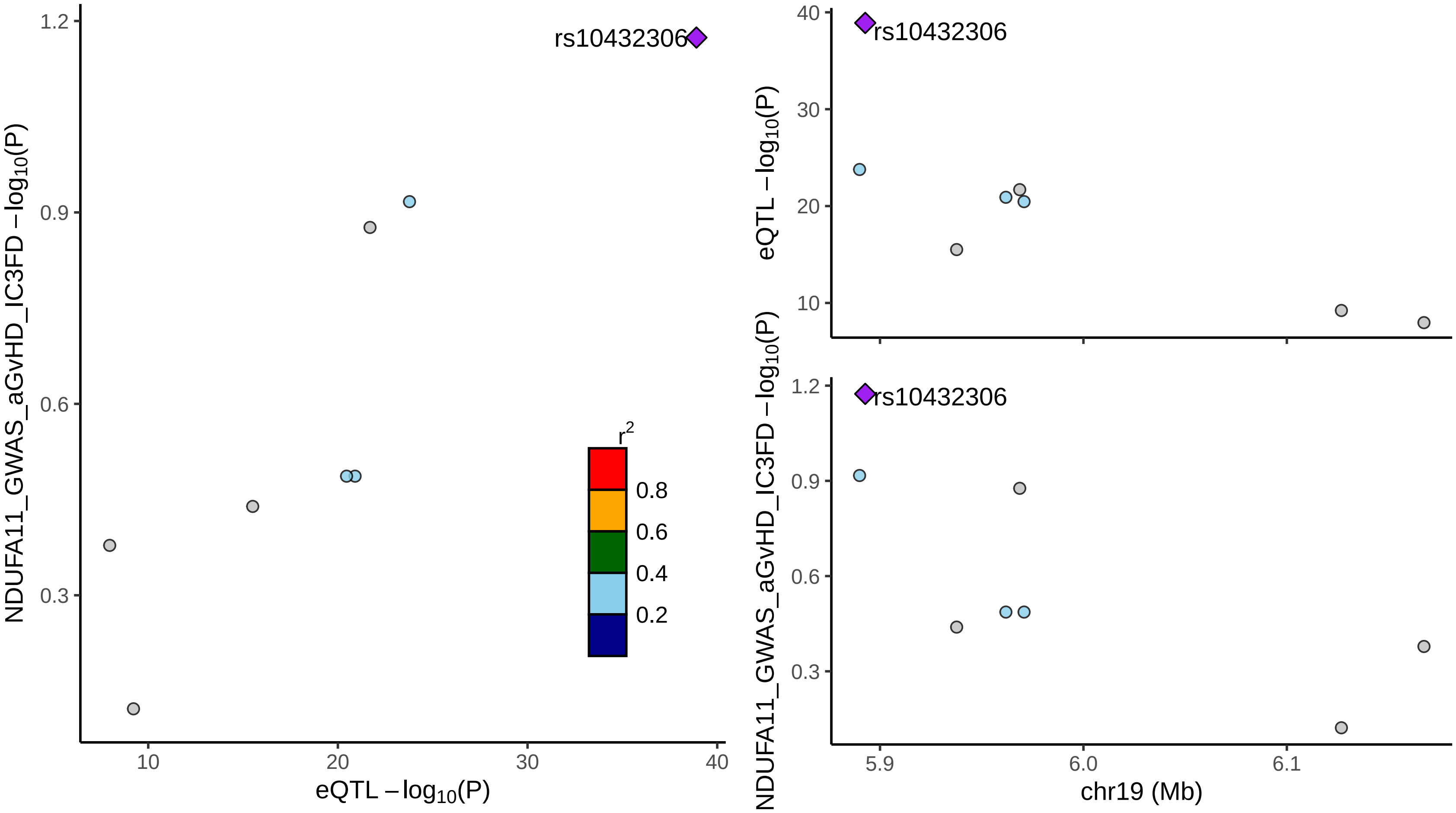

rs10432306
NDUFA11_GWAS_aGvHD_IC3FD −log10(P)
eQTL −log10(P)
r2
0.8
0.6
0.4
0.2
1.2
0.9
0.6
0.3
10
20
30
40
chr19 (Mb)
5.9
6.0
6.1

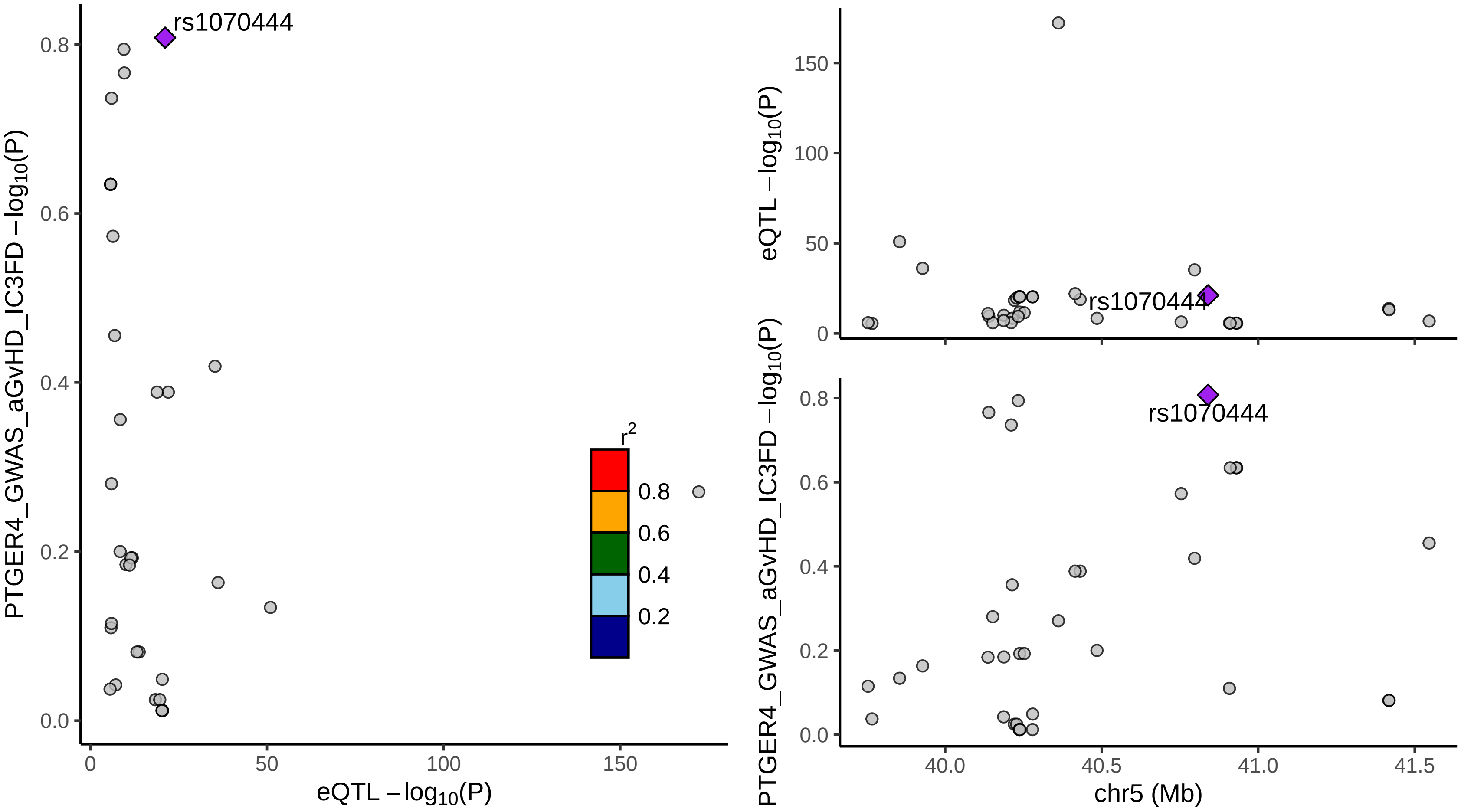

rs1070444
PTGER4_GWAS_aGvHD_IC3FD −log10(P)
0.0
0.2
0.4
0.6
0.8
eQTL −log10(P)
0
50
100
150
r2
0.2
0.4
0.6
0.8
eQTL −log10(P)
0
50
100
150
rs1070444
PTGER4_GWAS_aGvHD_IC3FD −log10(P)
0.0
0.2
0.4
0.6
0.8
rs1070444
40.0
40.5
41.0
41.5
chr5 (Mb)

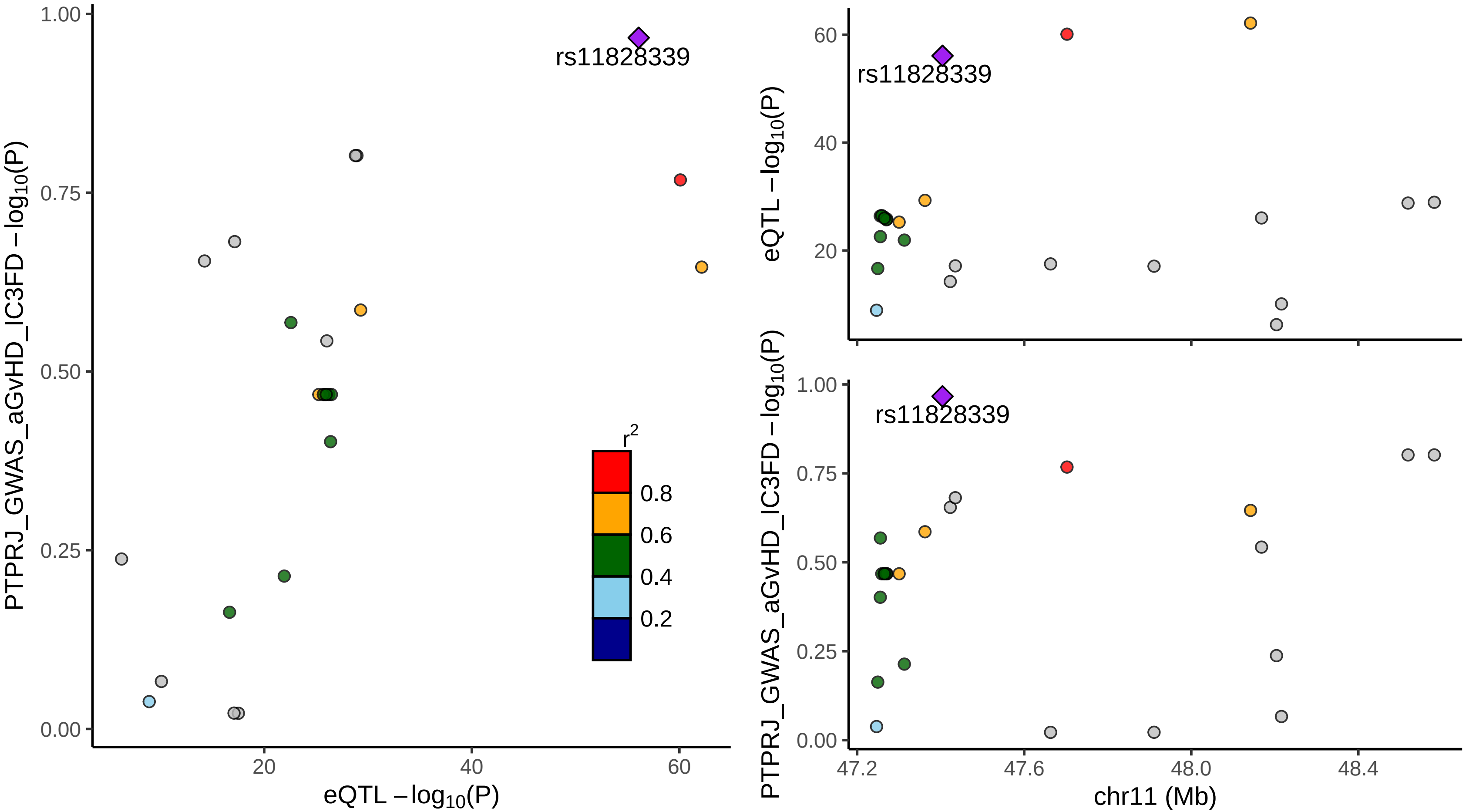

rs11828339
PTPRJ_GWAS_aGvHD_IC3FD −log10(P)
eQTL −log10(P)
1.00
0.75
0.50
0.25
0.00
20
40
60
r2
0.8
0.6
0.4
0.2
chr11 (Mb)
47.2
47.6
48.0
48.4

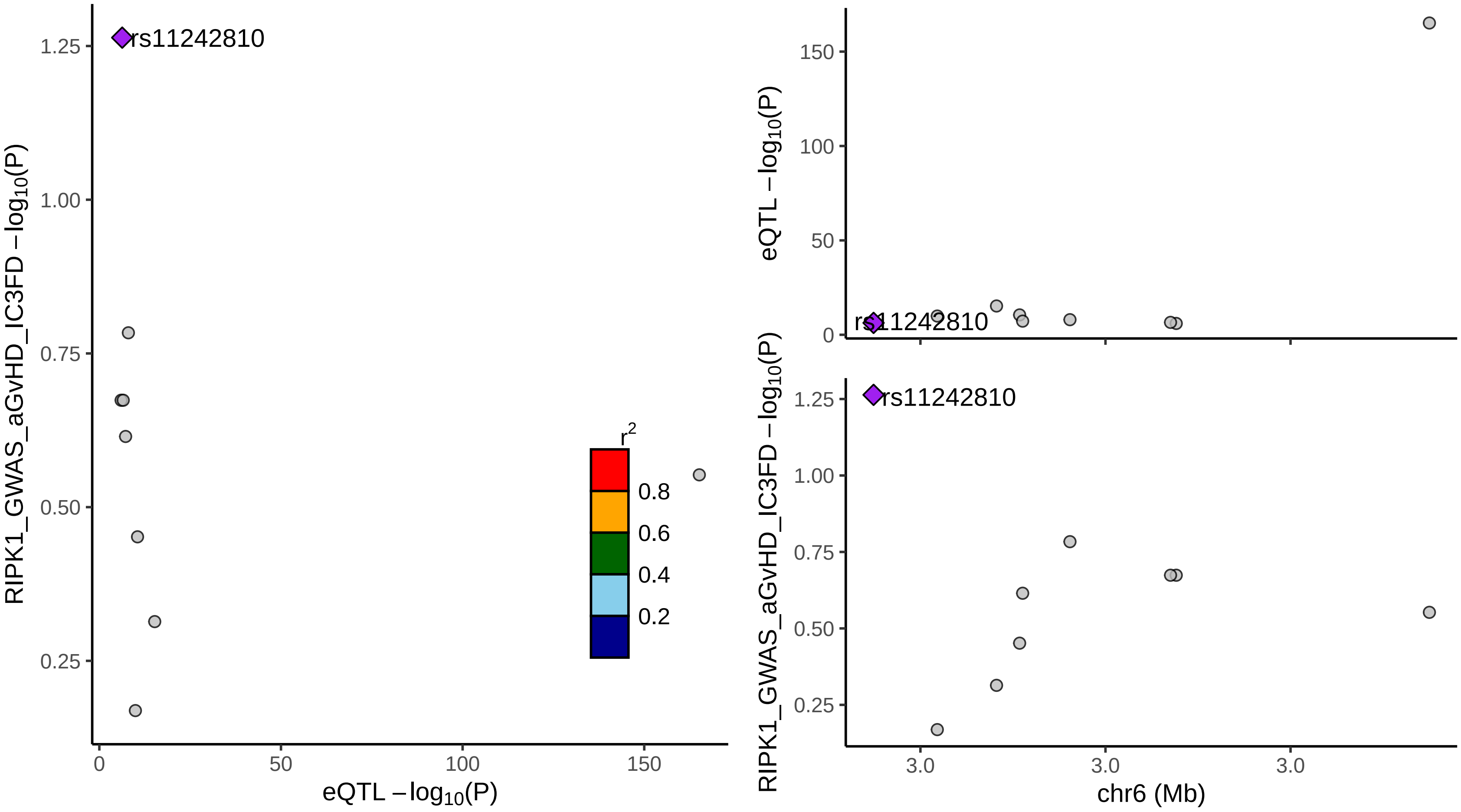

rs11242810
RIPK1_GWAS_aGvHD_IC3FD −log10(P)
1.25
1.00
0.75
0.50
0.25
0
50
100
150
eQTL −log10(P)
r2
0.8
0.6
0.4
0.2
eQTL −log10(P)
150
100
50
0
rs11242810
RIPK1_GWAS_aGvHD_IC3FD −log10(P)
rs11242810
1.25
1.00
0.75
0.50
0.25
3.0
3.0
3.0
chr6 (Mb)

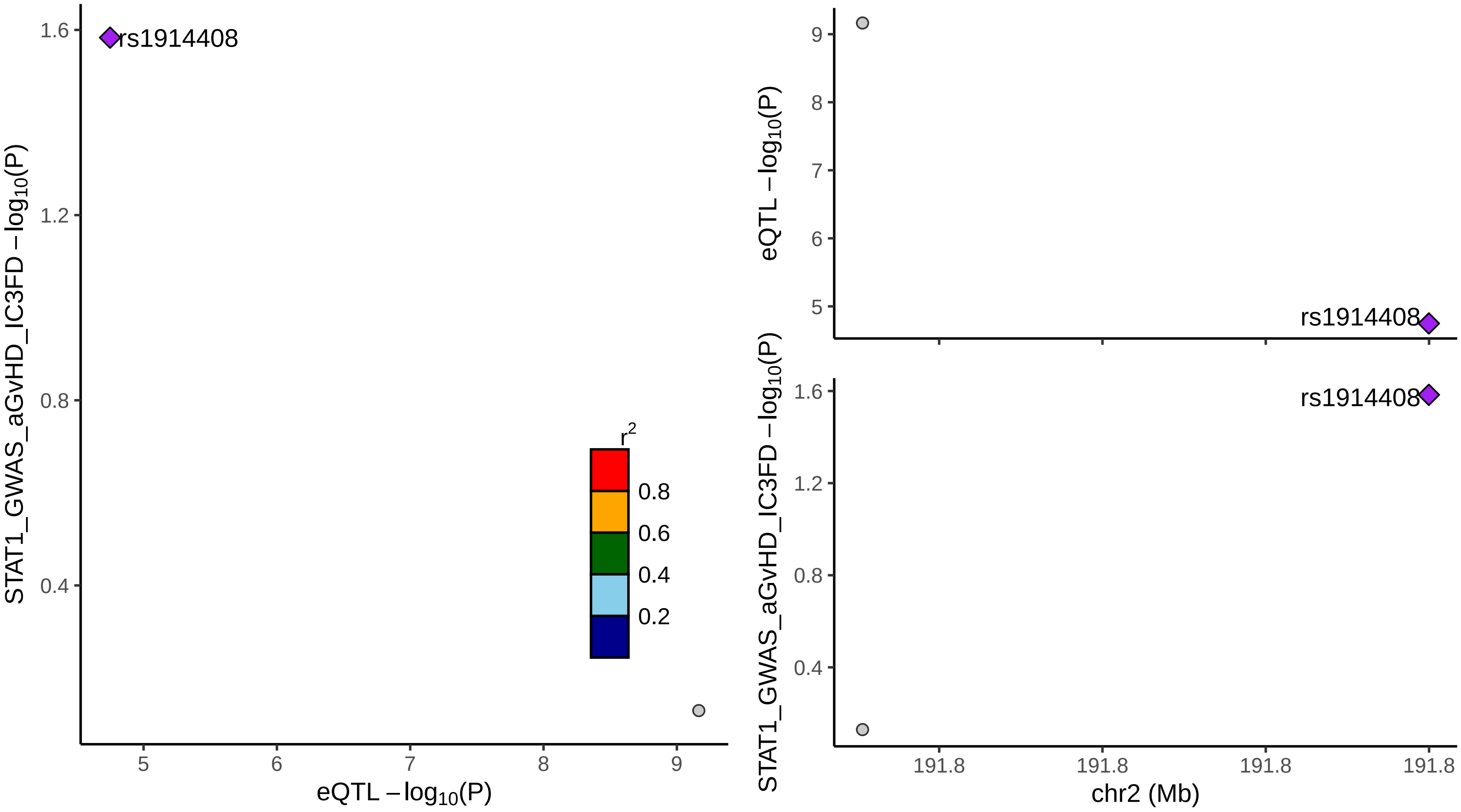
rs1914408
1.6
1.2
0.8
0.4
STAT1_GWAS_aGvHD_IC3FD −log10(P)
5
6
7
8
9
eQTL −log10(P)
r2
0.8
0.6
0.4
0.2
9
8
7
6
5
eQTL −log10(P)
rs1914408
rs1914408
1.6
1.2
0.8
0.4
STAT1_GWAS_aGvHD_IC3FD −log10(P)
191.8
191.8
191.8
191.8
chr2 (Mb)

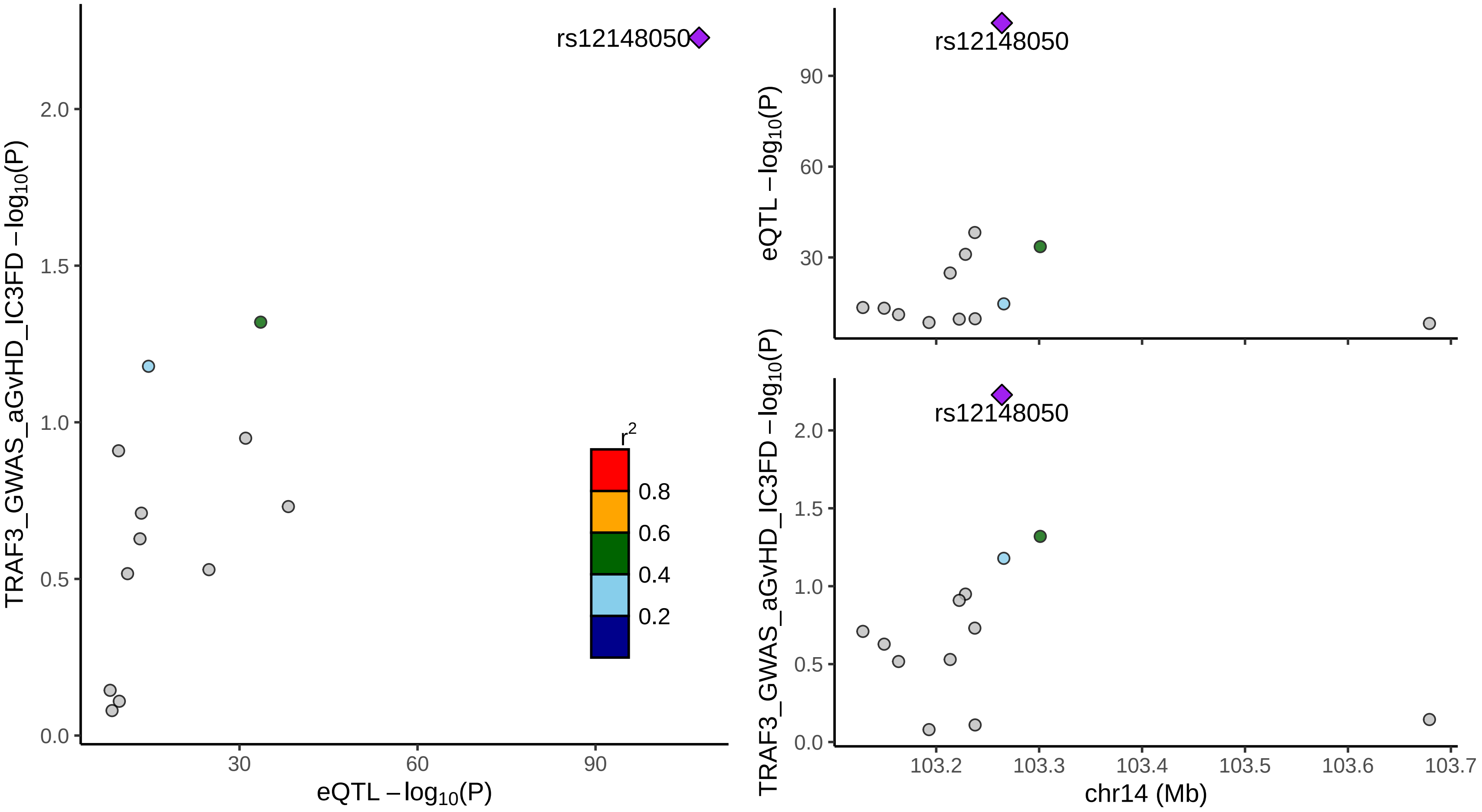
rs12148050
TRAF3_GWAS_aGvHD_IC3FD −log10(P)
eQTL −log10(P)
r2
0.8
0.6
0.4
0.2
chr14 (Mb)
103.2
103.3
103.4
103.5
103.6
103.7

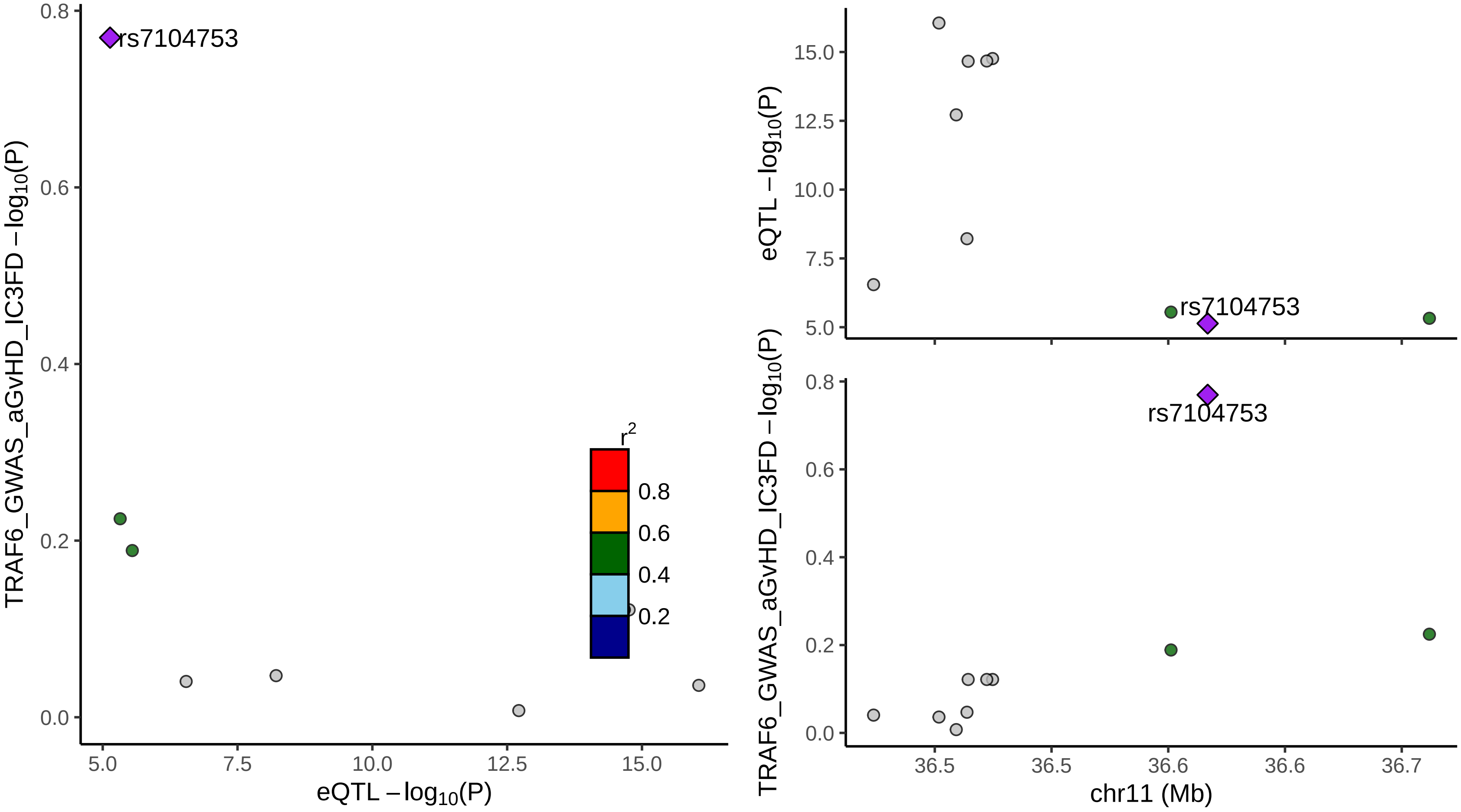
rs7104753
TRAF6_GWAS_aGvHD_IC3FD −log10(P)
eQTL −log10(P)
r2
0.8
0.6
0.4
0.2
0.0
5.0
7.5
10.0
12.5
15.0
rs7104753
eQTL −log10(P)
rs7104753
TRAF6_GWAS_aGvHD_IC3FD −log10(P)
36.5
36.6
36.7
chr11 (Mb)

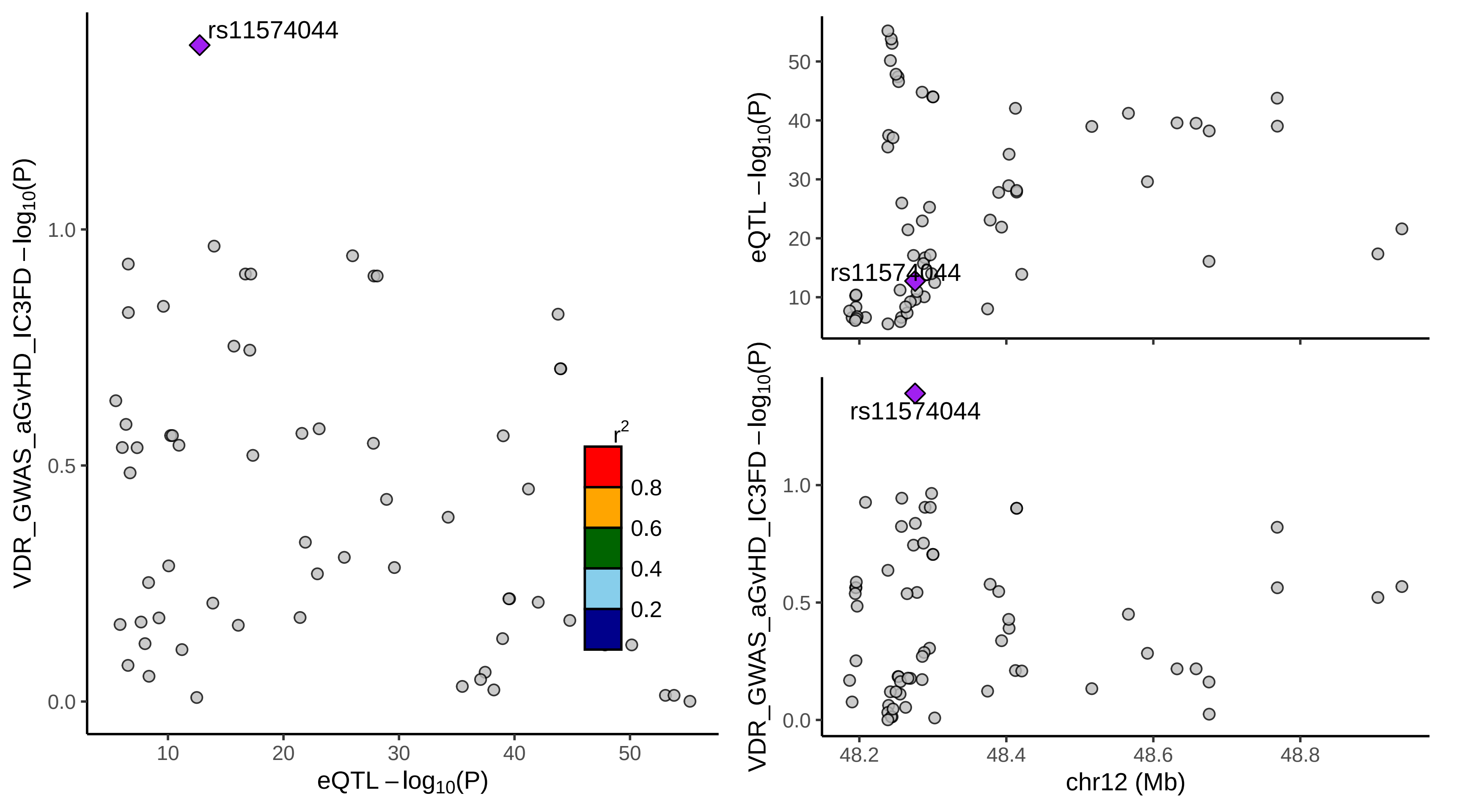

rs11574044
VDR_GWAS_aGvHD_IC3FD −log10(P)
eQTL −log10(P)
r2
0.8
0.6
0.4
0.2
chr12 (Mb)

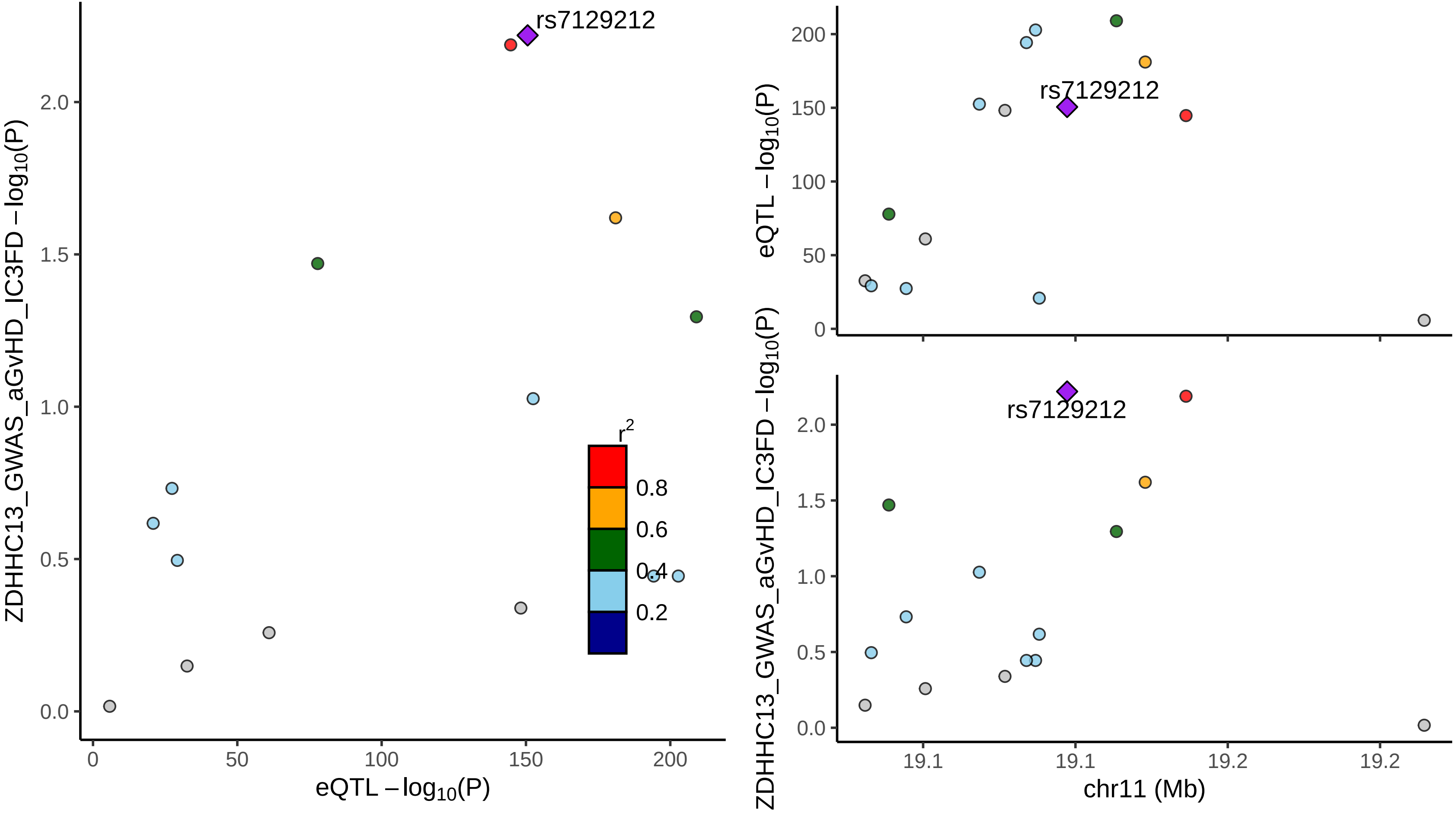
rs7129212
ZDHHC13_GWAS_aGvHD_IC3FD −log10(P)
2.0
1.5
1.0
0.5
0.0
0
50
100
150
200
eQTL −log10(P)
r2
0.8
0.6
0.4
0.2
rs7129212
eQTL −log10(P)
200
150
100
50
0
rs7129212
ZDHHC13_GWAS_aGvHD_IC3FD −log10(P)
2.0
1.5
1.0
0.5
0.0
19.1
19.1
19.2
19.2
chr11 (Mb)

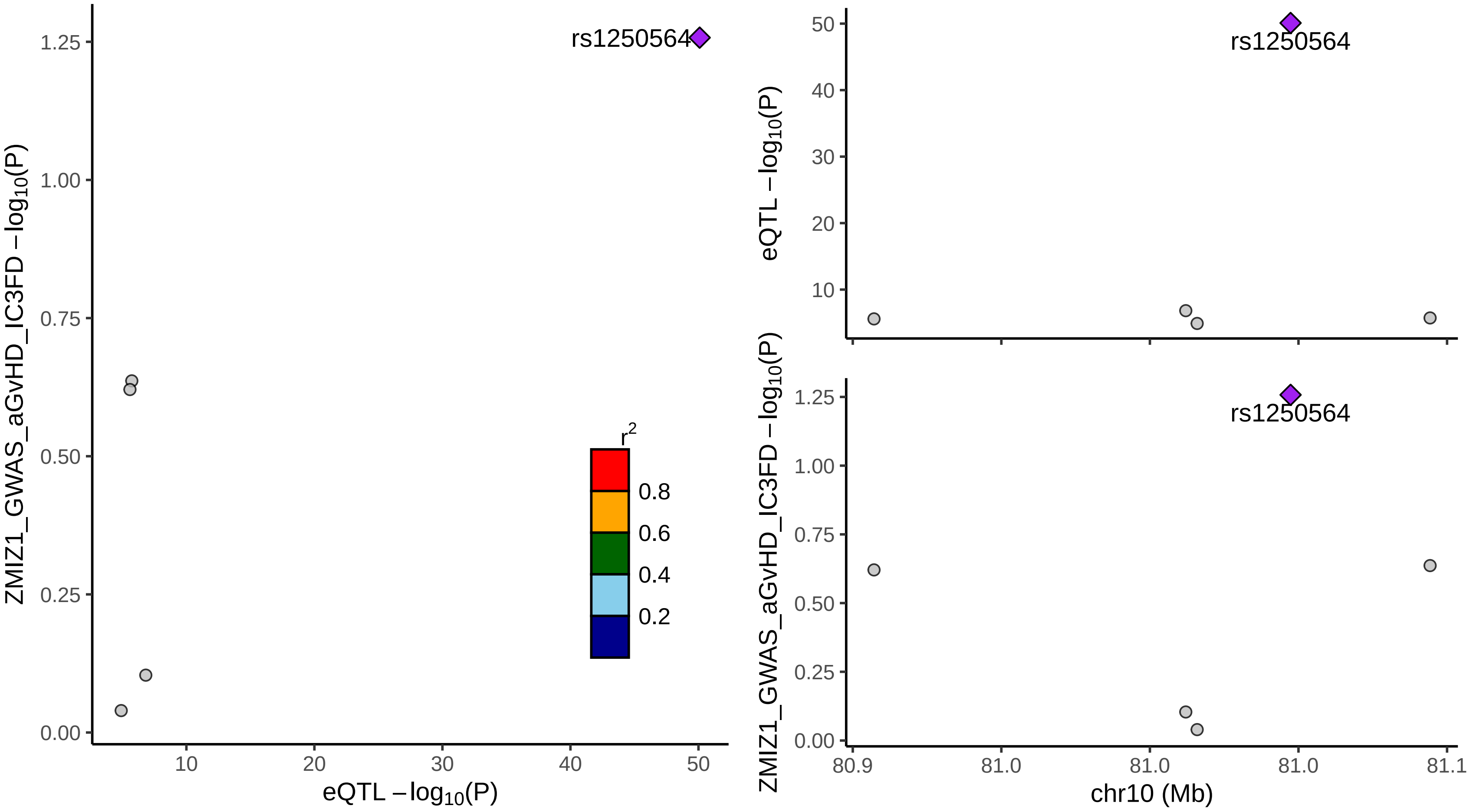
rs1250564
ZMIZ1_GWAS_aGvHD_IC3FD −log10(P)
1.25
1.00
0.75
0.50
0.25
0.00
10
20
30
40
50
eQTL −log10(P)
r2
0.8
0.6
0.4
0.2
eQTL −log10(P)
50
40
30
20
10
rs1250564
ZMIZ1_GWAS_aGvHD_IC3FD −log10(P)
80.9
81.0
81.0
81.0
81.1
chr10 (Mb)
